# miRNAs from Inflamed Gingiva Link Gene Signaling to Increased MET Expression

**DOI:** 10.1177/00220345231197984

**Published:** 2023-10-11

**Authors:** L. Zheng, A. Chopra, J. Weiner, D. Beule, H. Dommisch, A. S. Schaefer

**Affiliations:** 1 Department of Periodontology, Oral Medicine and Oral Surgery, Institute for Dental and Craniofacial Sciences, Charité–University Medicine Berlin, corporate member of Freie Universität Berlin, Humboldt-Universität zu Berlin, and Berlin Institute of Health, Berlin, Germany; 2 Core Unit Bioinformatics, Berlin Institute of Health, Berlin, Germany

**Keywords:** cell cycle, mesenchymal cellular migration, periodontitis, *CPEB1*, *ABCA1*, *ATP6V1C1*

## Abstract

Several array-based microRNA (miRNA) expression studies independently showed increased expression of miRNAs hsa-miR-130a-3p, -142-3p, -144-3p, -144-5p, -223-3p, -17-5p, and -30e-5p in gingiva affected by periodontal inflammation. We aimed to determine direct target genes and signaling pathways regulated by these miRNAs to identify processes relevant to gingival inflammatory responses and tissue homeostasis. We transfected miRNA mimics (mirVana) for each of the 7 miRNAs separately into human primary gingival fibroblasts cultured from 3 different donors. Following RNA sequencing, differential gene expression and second-generation gene set enrichment analyses were performed. miRNA inhibition and upregulation was validated at the transcript and protein levels using quantitative reverse transcriptase polymerase chain reaction, Western blotting, and reporter gene assays. All 7 miRNAs significantly increased expression of the gene *MET* proto-oncogene, receptor tyrosine kinase (*MET*). Expression of known periodontitis risk genes *CPEB1*, *ABCA1*, and *ATP6V1C1* was significantly repressed by hsa-miR-130a-3p, -144-3p, and -144-5p, respectively. The genes *WASL*, *ENPP5*, *ARL6IP1*, and *IDH1* showed the most significant and strongest downregulation after hsa-miR-142-3p, -17-5p, -223-3p, and -30e-5p transfection, respectively. The most significantly regulated gene set of each miRNA related to cell cycle (hsa-miRNA-144-3p and -5p [*P*_adj_ = 4 × 10^−40^ and *P*_adj_ = 4 × 10^−6^], -miR-17-5p [*P*_adj_ = 9.5 × 10^−23^], -miR-30e-5p [*P*_adj_ = 8.2 × 10^−18^], -miR-130a-3p [*P*_adj_ = 5 × 10^−15^]), integrin cell surface interaction (-miR-223-3p [*P*_adj_ = 2.4 × 10^−7^]), and interferon signaling (-miR-142-3p [*P*_adj_ = 5 × 10^−11^]). At the end of acute inflammation, gingival miRNAs bring together complex regulatory networks that lead to increased expression of the gene *MET*. This underscores the importance of mesenchymal cell migration and invasion during gingival tissue remodeling and proliferation in restoring periodontal tissue homeostasis after active inflammation. *MET*, a receptor of the mitogenic hepatocyte growth factor fibroblast secreted, is a core gene of this process.

## Introduction

MicroRNAs (miRNAs) are short single-strand nonprotein coding RNAs with inhibiting functions on gene activity, generally by interacting with the 3′-untranslated regions (3′UTR) of target protein-coding messenger RNAs (mRNAs) (Ha and Kim 2014). Individual miRNAs modulate the expression of distinct genes and, thereby, can affect complex gene networks (Selbach et al. 2008). As a result, miRNAs modulate a range of biological processes. Likewise, miRNAs are implicated in complex diseases, and affected tissues exhibit differential miRNA expression (Paul et al. 2018). To identify miRNAs that are differentially expressed in periodontitis, several studies generated array-based expression profiles of miRNAs in healthy and inflamed periodontal tissues and reported the differential expression of numerous miRNAs in the gingiva (Lee et al. 2011; Xie et al. 2011; Perri et al. 2012; Stoecklin-Wasmer et al. 2012; Ogata et al. 2014) and saliva (Fujimori et al. 2019). However, the target genes and biological processes regulated by these miRNAs in periodontal tissues are still unknown.

We hypothesized that differently expressed miRNAs are suitable targets for the identification of those genes and functional gene networks that are directly involved in the etiology of periodontitis. This is because overall, miRNAs are predicted to target only ~10% to 30% of protein-coding genes, and each miRNA represses on average 200 transcripts (Brennecke et al. 2005; Lall et al. 2006). This considerably reduces the level of complexity and can be a straightforward approach to identify genes and pathways with relevant functions in disease development and restoring health. However, a limitation of studying biological processes in gingival biopsies is that they consist of multiple different tissue types, and in particular, inflamed gingival samples have a share of up to 50% of invaded immune cells (Richter et al. 2021). In addition, fluctuations in the ratio of different cell types can be caused by different sampling times, and differences in analysis design between studies can also affect the results. Therefore, specific observations of individual genomewide expression studies must be interpreted with caution. In contrast, however, it is likely that genes found to be differentially expressed independently in multiple studies of the same disease state do not represent experimental artifacts but are actually involved in the disease process. Therefore, we selected miRNAs that several miRNA expression profiling studies independently showed to be more expressed in gingival biopsies (or saliva) collected immediately prior to periodontal surgery.

In the current study, we aimed to identify genes and gene networks, directly regulated by miRNAs upregulated during gingival inflammation. This would reveal molecular mechanisms with relevant functions for the disease etiology. For this purpose, we selected miRNAs that have been reported to be differentially expressed between healthy and inflamed gingival biopsies. We overexpressed these miRNAs in human primary gingival fibroblasts and performed mRNA sequencing, followed by quantitative reverse transcriptase polymerase chain reaction (qRT-PCR) and Western blotting, to validate the key results.

## Material and Methods

### miRNA Selection

Published array-based miRNA expression studies were identified using the search terms *miRNA*, *periodontitis*, *gingiva*, and *oral inflammation* in the PubMed database. We identified 6 studies (Lee et al. 2011; Xie et al. 2011; Perri et al. 2012; Stoecklin-Wasmer et al. 2012; Ogata et al. 2014; Fujimori et al. 2019). From these studies, we selected miRNAs that were reported by ≥2 studies and showed >2-fold change between affected and healthy gingiva or saliva. These were miRNAs hsa-miR-142-3p, -130a-3p, and -30e-5p (Appendix Table 1). Four studies found increased expression of miRNA-144 without completely indicating strand orientation. In addition, miR-144-3p and -144-5p have very similar expression levels. Therefore, we selected both miR-144-3p and -144-5p. In addition, we selected hsa-miR-223-3p because it was previously found to downregulate the transcription factors (TFs) *MAFB* and *STAT1* (Saadatian et al. 2019). These TFs also regulate the periodontitis risk genes *SIGLEC5* (Mueller et al. 2022) and *CDKN2B-AS1* (Harismendy et al. 2011). Furthermore, we also selected hsa-miR-17-5p, because it downregulates the periodontitis risk gene *ABCA1* (Fan et al. 2020). These 2 miRNAs were found in only 2 of the 6 published array-based miRNA expression studies with a >2-fold change in expression and were added additionally because we considered them interesting candidates.

### Cell Culture and miRNA Transfection

Primary human gingival fibroblasts (phGFs) were cultured from biopsies of the healthy oral masticatory mucosa that were collected from 3 different donors at a defined site of the hard palate adjacent to the fourth and fifth teeth by the use of a tissue puncher with a 3-mm diameter and a depth of approximately 1 mm as previously described (Richter et al. 2019). In brief, cells were cultured in cell growth medium (Dulbecco’s modified Eagle’s medium, 10% fetal bovine serum [FBS], 1% amphotericin B, 1% pen/strep, 1% nonessential amino acids). The cells were sown with a density of 1.6 × 10^5^ cells/well in 6-well tissue culture plates (TPP Techno Plastic Products) 1 d before transfection. We used mirVana mimics miR-130a-3p, -142-3p, -144-3p,-144-5p, -17-5p, -30e-5p, and -223-3p. As a positive control, we used mirVana mimic miR-1, which specifically downregulates the expression of the gene protein tyrosine kinase 9 (*PTK9*), and as a negative control, we used mirVana mimic Negative Control #1 (ThermoFisher Scientific). Each mirVana mimic was separately transfected in a concentration of 30 pmol. The transfections were performed with Lipofectamine RNAiMAX (ThermoFisher Scientific) reagent according to the manufacturer’s protocol. The mimics were transfected into the 3 different phGFs as biological replicates and also into Immortalized Human Gingival Fibroblasts (ihGFs). Twenty-four hours after transfection, the cells were washed twice with phosphate-buffered saline (PBS). Total RNA was extracted using the RNeasy Mini Kit (Qiagen).

### qRT-PCR and RNA Sequencing

Before RNA sequencing (RNA-seq), we determined the transfection efficiency and functionality of the miRNA-positive control mimic by quantifying *PTK9* transcript levels with qRT-PCR (Appendix Fig. 1). Complementary DNA (cDNA) was synthesized from 500 ng DNaseI (Roche) treated total RNA, using the High-Capacity cDNA Reverse Transcription Kit (Applied Biosystems). qRT-PCR was run with the SYBR Select Master Mix (Applied Biosystems) on a CFX Connect System (Bio-Rad). Primer sequences are listed in the Appendix Methods.

RNA-seq was performed at the Berlin Institute of Health, Core Facility Genomics as recently described (Chopra et al. 2022). In brief, 500 to 1,000 ng total RNA of transfected cell cultures was sequenced with 16 million reads (75-bp single end) on a NextSeq 500 using the NextSeq 500/550 High Output Kit v2.5 (75 cycles).

We give a detailed description of the methods used for luciferase reporter gene assays and Western blotting in the Appendix Methods.

## Results

### Periodontitis Risk Genes *CPEB1*, *ABCA1*, and *ATP6V1C1* Are Repressed Direct Targets of the Selected miRNAs

**Figure 1.**
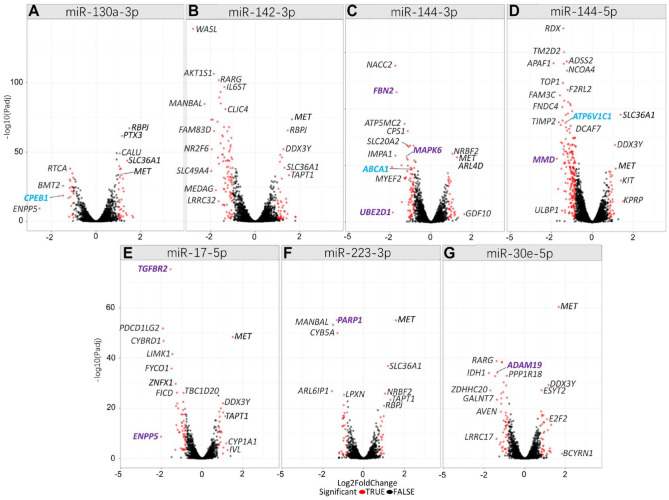
Volcano plots for microRNA (miRNA) regulated genes in primary human gingival fibroblasts (phGFs). (**A**) hsa-miR-130a-3p upregulation affected expression of 1,819 genes with *P*_adj_ < 0.05. In total, 315 genes had a predicted hsa-miR-130a-3p binding site; 272 showed reduced expression, and 48 indicated increased expression. A total of 1,503 differentially expressed genes had no predicted hsa-miR-130a-3p binding site, 433 of which were downregulated and 1,070 upregulated. (**B**) hsa-miR-142-3p upregulation affected 2,203 genes (*P*_adj_ < 0.05). A total of 252 genes had predicted hsa-miR-142-3p binding sites, with 241 showing reduced expression and 11 showing increased expression. (**C**) hsa-miR-144-3p upregulation influenced the expression of 19,647 genes (*P*_adj_ < 0.05), with 449 of them having predicted hsa-miR-144-3p binding sites, 420 of which showed reduced expression and 29 showed increased expression. (**D**) hsa-miR-144-5p upregulation affected 19,647 genes (*P*_adj_ < 0.05). In total, 129 had predicted hsa-miR-144-5p binding sites, 128 of which showed decreased expression and 1 (*DPYD*) increased expression. (**E**) hsa-miR-17-5p upregulation affected 880 genes (*P*_adj_ < 0.05). Of these genes, 358 had predicted hsa-miR-17-5p binding sites, 336 of which showed reduced expression and 22 genes showed increased expression. (**F**) Upregulation of hsa-miR-223-3p resulted in the expression of 852 genes (*P*_adj_ < 0.05), with 131 of them having predicted hsa-miR-223-3p binding sites. All of these genes showed reduced expression. (**G**) hsa-miR-30e-5p upregulation affected 1,113 genes (*P*_adj_ < 0.05). Of 365 genes with predicted hsa-miR-30e-5p binding sites, 358 showed decreased expression, whereas 7 showed increased expression (bold, purple = reported miRNA target gene, blue = reported periodontitis risk gene).

After hsa-miR-130a-3p upregulation, *ENPP5* (ectonucleotide pyrophosphatase/phosphodiesterase family member 5; miRNA target score = 96) was most downregulated with a log_2_ fold change (log_2_FC) of −2.5 (*P*_adj_ = 6.9 × 10^−12^) (Fig. 1A, Appendix Tables 2–8). The second most downregulated gene was periodontitis risk gene *CPEB1* (cytoplasmic polyadenylation element binding protein 1) (Rhodin et al. 2014) (log_2_FC = −1.5, *P*_adj_ = 1.7 × 10^−21^), having the highest target score (100) for hsa-miR-130a-3p binding (Table 1).

**Table 1.** Ten Most Downregulated Genes after miRNA Transfection into Primary Human Gingival Fibroblasts.

miRNA	Gene	*P*_adj_	Fold Change (Down)	Target Score
*hsa-miR-130a-3p*	*ENPP5*	6.9E-12	5.81	96
	***CPEB1***	1.7E-21	2.81	100
	*BMT2*	5.4E-29	2.79	92
	*IMPDH1*	1.3E-33	2.26	94
	*MYBL1*	2.0E-27	2.26	99
	*RTCA*	6.4E-42	2.24	95
	*THOP1*	1.4E-22	2.13	86
	*NACC2*	1.2E-25	2.13	92
	*DICER1*	2.8E-35	2.08	91
	*GAREM1*	2.5E-05	2.08	93
*hsa-miR-142-3p*	*WASL*	7.1E-140	6.87	94
	*SLC49A4*	3.2E-37	3.89	96
	*CFL2*	3.4E-74	3.89	88
	*NR2F6*	6.4E-53	3.85	83
	*TSEN34*	2.8E-74	3.73	94
	*AKT1S1*	2.9E-107	3.62	86
	*LRRC32*	2.5E-15	3.12	88
	*RARG*	8.7E-103	3.10	81
	*INPP5A*	1.8E-42	3.08	86
	*MRFAP1*	2.4E-94	2.94	89
*hsa-miR-144-3p*	*UBE2D1*	3.7E-04	5.03	100
	*NACC2*	4.9E-56	4.47	99
	*IMPA1*	2.9E-24	4.44	90
	*FBN2*	1.2E-46	4.28	99
	*MYEF2*	2.9E-16	3.07	99
	*ATP5MC2*	1.8E-35	3.03	95
	*GPR176*	3.0E-16	2.96	84
	*ARNTL2*	4.3E-16	2.93	83
	*FZD6*	1.3E-16	2.87	99
	*MAPK6*	2.3E-23	2.77	99
*hsa-miR-144-5p*	*MMD*	3.7E-23	4.47	86
	*FAM3C*	1.5E-45	3.92	81
	*RRAGC*	1.2E-43	3.27	92
	***ATP6V1C1***	8.1E-36	3.10	95
	*APBB1*	2.4E-32	2.58	91
	*RAB33A*	2.8E-04	2.58	94
	*TFG*	1.2E-42	2.57	93
	*DRAM2*	3.1E-19	2.55	83
	*REEP3*	7.4E-28	2.51	83
	*ZSCAN31*	4.7E-09	2.45	84
*hsa-miR-30e-5p*	*IDH1*	9.00E-35	2.20	90
	*SEC23A*	1.50E-33	1.82	95
	*LRRC17*	1.70E-08	1.74	99
	*RARG*	2.40E-43	1.73	99
	*GALNT7*	5.40E-27	1.71	98
	*ADAM19*	7.70E-35	1.69	96
	*AVEN*	2.30E-19	1.47	81
	*SKP2*	1.10E-24	1.45	91
	*SCML1*	1.30E-26	1.44	98
	*TMEM181*	3.40E-39	1.42	98
*hsa-miR-17-5p*	*ENPP5*	1.90E-09	2.53	100
	*PDCD1LG2*	1.50E-52	2.41	99
	*CYBRD1*	1.50E-47	2.37	89
	*TGFBR2*	4.10E-76	1.94	86
	*FYCO1*	1.60E-36	1.87	100
	*LIMK1*	2.50E-42	1.83	94
	*KCNB1*	3.7E-04	1.65	99
	*ZNFX1*	1.90E-30	1.63	100
	*SLC40A1*	4.90E-14	1.58	99
	*TMEM64*	1.10E-22	1.53	89
*hsa-miR-223-3p*	*PARP1*	7.10E-56	1.64	89
	*TBC1D17*	1.70E-13	1.30	85
	*SLC35G2*	7.80E-17	1.28	93
	*SLC7A8*	1.30E-16	1.26	91
	*SLC4A4*	9.30E-18	1.24	97
	*CBX5*	1.30E-17	1.18	95
	*FBXO8*	6.40E-14	1.17	94
	*ARMCX1*	7.40E-19	1.16	94
	*RCN2*	1.00E-07	1.02	80
	*NUP210*	4.47E-02	1.00	86

For each microRNA (miRNA), the top 10 most downregulated target genes with a miRNA target score ≥80 are listed. Previously reported periodontitis risk genes are highlighted in bold letters. Note that hsa-miR-144-3p regulates periodontitis risk gene *ABCA1* (fold change downregulation = 2.23, *P*_adj_ = 1.4 × 10^−19^), which is on position 29 (see Appendix Tables 2–8 for the complete list).

After hsa-miR-142-3p upregulation, the gene *WASL* (WASP like actin nucleation promoting factor; *NWASP*) was downregulated the most (log_2_FC = −2.78, *P*_adj_ = 7.1 × 10^−140^, target score = 94) (Fig. 1B).

After hsa-miR-144-3p upregulation, the known target gene *UBE2D1* (ubiquitin conjugating enzyme E2 D1) (Li et al. 2021) showed first downregulation with log_2_FC = −2.3 (*P*_adj_ = 3.7 × 10^−4^) (Fig. 1C).

After hsa-miR-144-5p upregulation, *MMD* (monocyte to macrophage differentiation related) was downregulated most (log2FC = −2.16, *P*_adj_ = 3.7 × 10^−23^) (Fig. 1D), which is a known target of hsa-miR-144-5p (Burgos et al. 2014), The periodontitis risk gene *ATP6V1C1* (Munz et al. 2019) was the fourth most downregulated gene.

After hsa-miR-17-5p upregulation, the strongest downregulated gene was *ENPP5* (ectonucleotide pyrophosphatase/phosphodiesterase family member 5) (Fig. 1E). The fourth top downregulated gene was the known target gene of this miRNA *TGFBR2* (transforming growth factor beta receptor 2).

After upregulation of hsa-miR-223-3p, the gene *ARL6IP1* (ADP ribosylation factor like GTPase 6 interacting protein 1) showed the most significant downregulation (log2FC = −1.938, *P*_adj_ = 1.5 × 10^−27^) (Fig. 1F).

hsa-miR-30e-5p downregulated *IDH1* (isocitrate dehydrogenase NADP(+)) most strongly (log2FC = −2.20, *P*_adj_ = 9.0 × 10^−35^) (Fig. 1G). hsa-miR-30e-5p known target gene *ADAM19* was the eighth most downregulated gene.

### Validation of the Inhibitory Effect of hsa-miR-130a-3p on the 3′UTR Sequence of *CPEB1*

**Figure 2.**
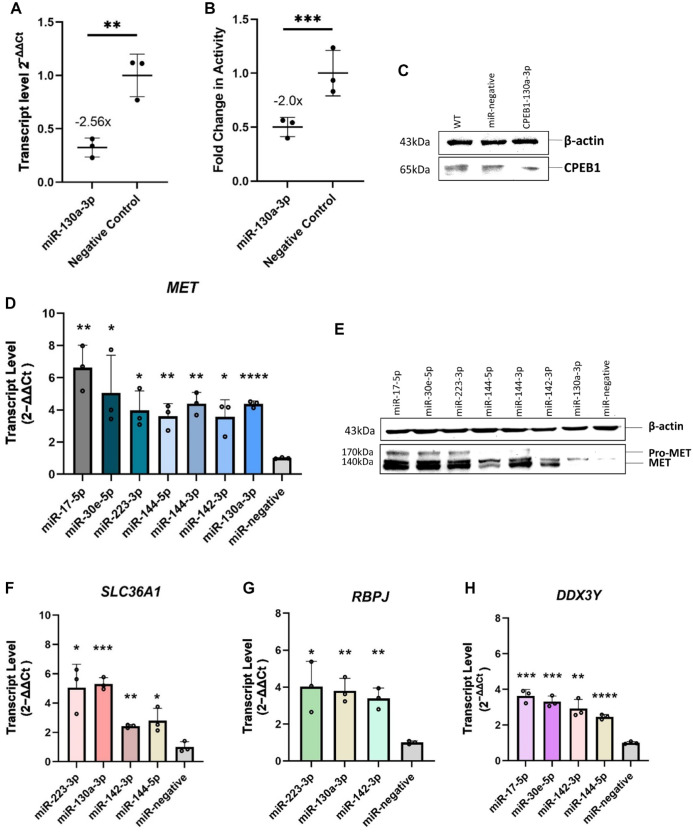
Validation of microRNA (miRNA) effects on the gene and protein levels. Cotransfection of *CPEB1*-3′UTR-Luc reporter gene plasmid with miR-130a-3p mimic significantly reduced Luc gene expression (*P* = 0.0076, fold change [FC] = −2.56; quantitative reverse transcriptase polymerase chain reaction) (**A**) and Luc protein activity (*P* = 0.0001, FC = −2.0) (**B**), as well as *CPEB1* protein concentration in ihGFs (**C**), detected by chemiluminescence. Transfection of miRNA mimics significantly upregulated expression of *MET* (**D**), and protein blotting showed that each of the miRNAs increased *MET* concentration in ihGFs (**E**). β-Actin (43 kDa), pro-MET (170 kDa), and MET (140 kDa). Transfection of miRNA mimics significantly upregulated expression of *SLC36A1* (**F**), *RBPJ* (**G**), and *DDX3Y* (**H**). *p < 0.05, **p < 0.01, ***p < 0.001, ****p < 0.0001.

We aimed to provide mechanistic evidence that *CPEB1* is directly regulated by hsa-miR-130a-3p. For this purpose, we tested whether the *CPEB1* 3′UTR sequence segment containing 3 hsa-miR-130a-3p binding motifs (Appendix Table 9) was sufficient to reduce reporter gene activity at the transcript and protein levels after hsa-miR-130a-3p transfection. ihGFs were transfected with a luciferase reporter gene expressing the *CPEB1* 3′UTR and containing the conserved hsa-miR-130a-3p binding motifs cloned into the 3′UTR of the reporter gene *Luc*. In ihGFs expressing the *Luc* gene with the *CPEB1* 3′UTR sequence, *Luc* messenger RNA (mRNA) transcript levels were significantly reduced, with FC = −2.56 (*P* = 0.0076, Fig. 2A), suggesting that hsa-miR-130a-3p also regulated *CPEB1* on the transcriptional level. In addition, luciferase activity was reduced with FC = −2.0 (*P* = 0.0001, Fig. 2B), and protein blotting result (Fig. 2C) also showed a clear downregulation, suggesting that the regulation of hsa-miR-130a-3p also affected protein activity.

### All miRNAs Significantly Upregulated the Gene *MET*

We found that increased levels of each of the 7 selected miRNAs correlated with increased expression of the MET proto-oncogene, receptor tyrosine kinase (*MET*) gene (Fig. 1).

The 3′UTR of *MET* mRNA contains no miRNA binding site for 5 miRNAs (has-miR-144-5p, -miR-142-3p, -miR-223-3p, -miR-17-5p, -miR-30e-5p). hsa-miR-144-3p had a very poorly conserved predicted *MET* binding site (target score 57), and has-miR-130a-3p had a more conserved *MET* binding site (target score 93). This indicated an indirect regulation by most miRNAs that occurs upstream in the various signaling cascades and would result in increased *MET* expression downstream, as we observed in our experiments.

To add evidence to the observation that all miRNAs had effects on *MET* expression, we validated the RNA-seq data at both the transcript and protein levels. Separate transfection of each of the miRNA mimics for 24 h resulted in a significant increase of *MET* mRNA expression (Fig. 2D, Appendix Table 10) and *MET* protein expression (Fig. 2E). We found that the genes *DDX3Y* and *SLC36A1* were also very strongly upregulated by 4 and *RBPJ* by 3 different miRNAs. We considered such a cluster unusual. However, if it was not an artifact, this cluster would indicate biological importance. We therefore independently validated the expression of these genes again by qRT-PCR (Fig. 2F–H, Appendix Table 10).

### Gene Set Enrichment Analyses

**Figure 3.**
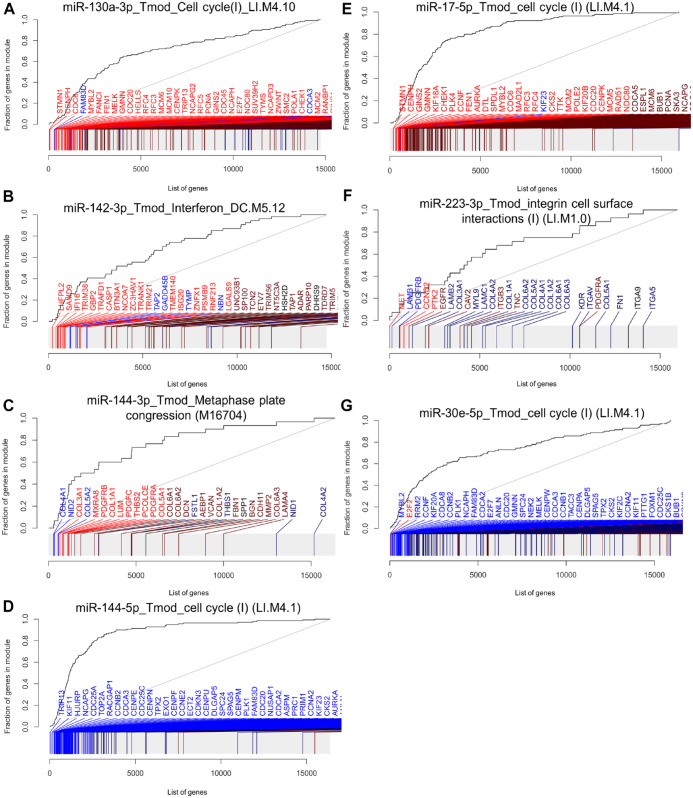
Most enriched gene sets after transfection of each microRNA (miRNA) into primary human gingival fibroblasts (phGFs). Evidence plots of the most enriched gene sets of all analyzed gene set collections are shown. (**A**) The “Cell cycle” gene set is most enriched by miR-130a-3p (area under the curve [AUC] = 0.66, *P*_adj_ = 5.0 × 10^−15^, 190 genes). (**B**) The “Interferon” gene set is most enriched by miR-142-3p (AUC = 0.56, *P*_adj_ = 1.1 × 10^−11^, 1,370 genes). (**C**) The “Metaphase plate congression” gene set is most enriched by miR-144-3p (AUC = 0.72, *P*_adj_ = 3.7 × 10^−7^, 54 genes). (**D**) The “Cell cycle” gene set is most enriched by miR-144-5p (AUC = 0.88, *P*_adj_ = 3.0 × 10^−37^, 140 genes). (**E**) The “Cell cycle” gene set is most enriched by miR-17-5p (AUC = 0.83, *P*_adj_ = 9.5 × 10^−23^, 140 genes). (**F**) The “Integrin” gene set is most enriched by miR-223-3p (AUC = 0.67, *P*_adj_ = 3.2 × 10^−2^, 28 genes). (**G**) The “Cell cycle” gene set is most enriched by miR-30e-5p (AUC = 0.73, *P*_adj_ = 8.2 × 10^−18^, 140 genes). Blue color = downregulation, red color = upregulation (see Appendix Figs. 2–8 for additional information).

We then performed gene set enrichment analysis (GSEA) to identify miRNA regulated gene sets. At first, we tested enrichment of the *MIR* gene sets specific for each of the transfected miRNA. MIR130A_3P (ID: M30650) was enriched with *P* = 9.5 × 10^−69^ (area under the curve [AUC] = 0.73), MIR142_3P (M31116) with *P* = 2.2 × 10^−98^ (AUC = 0.89), MIR144-3p with *P* = 9 × 10^−106^ (AUC = 0.74), MIR144-5p with *P* = 3.2 × 10^−31^ (AUC = 0.9), MIR17-5p(ID:M30516) with *P* = 6.2 × 10^−127^ (AUC = 0.74), MIR223-3p(ID:M31334) with *P* = 4.2 × 10^−27^ (AUC = 0.73), and MIR30e-5p (ID:M30442) with *P* = 1.6 × 10^−103^ (AUC = 0.70). The observed enrichment of the miRNA genes sets specific to each miRNA proved biologically functional effects of the transfected miRNA mimic, adding validity to our findings. Next, we performed GSEA with the coexpression gene set tmod and the Molecular Signatures Database (MSigDB) gene sets reactome, hallmark, KEGG, and GO. The top significantly enriched gene sets found in the different collections are given in Table 2 and Figure 3. The most significantly enriched gene set of each analyzed gene set collection is given for each miRNA in Appendix Figures 2 to 8 and Appendix Tables 2 to 8. The gene set collections for hsa-miR-130a-3p showed significant enrichment in gene sets related to cell cycle regulation and cytokine signaling. Similarly, hsa-miR-142-3p was associated with gene sets involved in cytokine signaling, cell locomotion, and adherens junctions. miRNA-144-3p exhibited enrichment in gene sets associated with extracellular matrix genes (tmod), regulation of cell division (GO), melanoma (Kegg), and more specific involvement in pathways such as “platelet derived growth factor receptor signaling pathway” and “regulation of vascular-associated smooth muscle cell proliferation,” as indicated by GO. miRNA-144-5p was consistently linked to functions in the mitotic cell cycle in all gene set collections. hsa-miR-17-5p and hsa-miR-30e-5p were found to be enriched in gene sets related to cell cycle regulation and transcription. Last, hsa-miR-223-3p was associated with the regulation of integrin cell surface interactions according to the gene set collections.

**Table 2.** Most Significantly Enriched Gene Set for Each Gene Set Collection after miRNA Transfection into Primary Human Gingival Fibroblasts.

miRNA	Gene Set	ID	Collection	Genes	AUC	*P*_adj_
hsa-miR-130a-3p	TNFA signaling via NFKB	M5890	Hallmark	190	0.66	5.0 × 10^−15^
	Cell cycle	M14460	GO	1,598	0.58	5.7 × 10^−15^
	Cell cycle (I)	LI.M4.1	Tmod	140	0.70	9.6 × 10^−9^
	Cytokine signaling in immune system	M1060	Reactome	660	0.58	8.0 × 10^−8^
	Pathways in cancer	M12868	KEGG	271	0.59	5.3 × 10^−5^
hsa-miR-142-3p	Locomotion	M13680	GO	1,370	0.56	1.1 × 10^−11^
	TNFA signaling via NFKB	M5890	Hallmark	190	0.65	4.7 × 10^−11^
	Cytokine signaling in immune system	M1060	Reactome	660	0.57	1.1 × 10^−7^
	Adherens junction	M638	KEGG	67	0.64	1.0 × 10^−5^
	Interferon	DC.M5.12	Tmod	54	0.69	1.3 × 10^−2^
hsa-miR-144-3p	Melanoma	M15798	KEGG	54	0.72	3.7 × 10^−6^
	Metaphase plate congression	M16704	GO	63	0.66	1.5 × 10^−4^
	Extracellular matrix (I)	LI.M2.0	Tmod	30	0.77	2.0 × 10^−3^
hsa-miR-144-5p	Cell cycle (I)	LI.M4.1	Tmod	140	0.88	3.0 × 10^−37^
	G2M checkpoint	M5901	Hallmark	199	0.72	4.8 × 10^−23^
	Mitotic prometaphase	M4217	Reactome	194	0.70	4.6 × 10^−15^
	Sister chromatid segregation	M536	GO	186	0.65	2.5 × 10^−10^
	Cell cycle	M7963	KEGG	122	0.65	2.3 × 10^−5^
hsa-miR-17-5p	Cell cycle (I)	LI.M4.1	Tmod	140	0.83	9.5 × 10^−23^
	Resolution of sister chromatid cohesion	M27181	Reactome	117	0.67	4.2 × 10^−6^
	E2F targets	M5925	Hallmark	200	0.72	8.3 × 10^−16^
	Replication	M16853	Kegg	36	0.74	5.8 × 10^−4^
	Negative regulation of small GTPase-mediated signal transduction	M11863	Go	50	0.66	8.8 × 10^−6^
hsa-miR-223-3p	Integrin cell surface interactions (I)	LI.M1.0	Tmod	28	0.67	3.2 × 10^−2^
	Promotes cell motility	M27778	Reactome	39	0.72	1.6 × 10^−4^
	Renal cell carcinoma	M13266	Kegg	64	0.68	4.8 × 10^−5^
	Regulation of protein localization to nucleus	M15292	Go	115	0.65	6.9 × 10^−5^
hsa-miR-30e-5p	cell cycle (I)	LI.M4.1	Tmod	140	0.73	8.2 × 10^−18^
	Regulation of cholesterol biosynthesis by SREBP (SREBF)	M27001	Reactome	55	0.66	2.7 × 10^−3^
	G2M checkpoint	M5901	Hallmark	198	0.66	2.8 × 10^−12^
	Attachment of spindle microtubules to kinetochor	M10211	Go	35	0.73	2.7 × 10^−3^

For each microRNA (miRNA), the top 1 most significantly enriched gene set collection with AUC ≥ 0.56 and Padj < .05 are listed. AUC, area under the curve; miRNA, microRNA.

## Discussion

In the current study, we identified the target genes and gene sets of 7 miRNAs whose expression is significantly increased in the course of periodontitis. We found that 3 miRNAs each regulated a periodontitis risk gene. These were *CPEB1* (Rhodin et al. 2014), *ABCA1* (Richter et al. 2022), and *ATP6V1C1* (Munz et al. 2019), which were regulated by hsa-miR-130a-3p, hsa-miR-144-3p, and hsa-miR-144-5p, respectively. In addition, *CPEB1* and *ATP6V1C1* were among the 10 most downregulated genes after miRNA upregulation. It was reported before that hsa-miR-144-3p and hsa-miR-144-5p regulate *ABCA1* and *ATP6V1C1* (Capstone Project: Data Science DSC180B; Genetic Overlap between Alzheimer’s, Parkinson’s, and healthy patients; replication project for the paper) (de Aguiar Vallim et al. 2013; Burgos et al. 2014; Wu et al. 2019). Our data confirm this regulation in ihGFs and demonstrated its importance in oral inflammation. The regulation of *CPEB1* by hsa-miR-130a-3p was previously unknown. We validated this interaction by showing that the specific miR-130a-3p binding sites within the 3′UTR of *CPEB1* are sufficient to repress transcript levels and protein activity of a reporter gene. *CPEB1* encodes a member of the cytoplasmic polyadenylation element binding protein family. This highly conserved protein binds to a specific RNA consensus sequence (5′-UUUUUAU-3′) found in the 3′UTR of some mRNAs and directs cytoplasmic polyadenylation and mediates both translational activation and repression (Mendez and Richter 2001; Welk et al. 2001). *CPEB1* and *CPEB4*, another periodontitis risk gene (Freitag-Wolf et al. 2021), are essential for successful mitotic cell division and have sequential nonredundant functions. In particular, *CPEB1* is specifically required for prophase entry and *CPEB4* for cytokinesis (Giangarra et al. 2015). *ABCA1*, *ATP6V1C1*, and *CPEB1* are currently being considered suggestive risk genes of periodontitis, because their associations did not reach genomewide significance (*P* < 5 × 10^−8^) in the explorative genome-wide association study GWAS (Fadista et al. 2016). We believe that the independent discovery of these genes as targets of these miRNAs upregulated in inflamed gingiva provides further evidence for their status as periodontitis susceptibility genes.

After overexpression of hsa-miR-142-3p, the gene *WASL* showed the most significant and strongest downregulation of the target genes of this miRNA. Loss of *WASL* function in mice has previously been reported to induce gingival inflammation and to increase the production of inflammatory cytokines (Wang et al. 2020). Specifically, *WASL* deficiency in hGFs activated the signaling pathways of NF-κB and MAPK, thereby increasing the production and infiltration of inflammatory cytokines and proliferation of keratinocytes (Kalailingam et al. 2017; Wang et al. 2020). *WASL* is involved in actin cytoskeletal reorganization, including signal-dependent regulation of actin dynamics, which is essential, for example, for cell locomotion (Stradal et al. 2004). Correspondingly, we found that the most hsa-miR-142-3p enriched gene sets were ‘TNFa signalling via NF-κB’ (Hallmark), ‘locomotion,’ and ‘cytoskeleton organization’ (GO).

For the other miRNAs, GSEA also indicated specific biological functions. hsa-miR-130a-3p transfection enriched the gene sets ‘cell cycle’ (tmod, GO) and ‘cytokine signaling’ (KEGG, Hallmark, Reactome). The 2 hsa-miR-144 transcripts have a role in the regulation of cell proliferation. hsa-miR-144-3p showed strongest enrichment of the gene sets ‘melanoma’ (KEGG) and cell division (‘metaphase plate congression’; GO), corresponding with recent findings of a role in the regulation of cell proliferation in various cancers (Sun et al. 2020). hsa-miR-144-5p also regulates cell cycle, unanimously shown by all gene set collections in the GSEA. The reliability of our data was enhanced by the discovery of 3 known target genes of hsa-miR-144-3p among the 30 most downregulated genes. These were the periodontitis risk gene *ABCA1* (de Aguiar Vallim et al. 2013), *FBN2* (fibrillin 2) (Mo et al. 2021), and *MAPK6* (mitogen-activated protein kinase 6) (Wu et al. 2019). FBN2 is a component of connective tissue microfibrils. It is supposed to regulate osteoblast maturation by controlling TGF-β bioavailability (inferred from sequence similarity, Uniprot). The function of *MAPK6* is unclear, but it may promote entry in the cell cycle (by similarity, Uniprot). In mice, knockdown of the *ATP6V1C1* ortholog severely impaired osteoclast acidification activity and bone resorption (Feng et al. 2009).

The meaningfulness of the GSEA was limited by the fact that the AUC values were often <0.8. We hypothesize that miRNAs have more subtle regulatory effects compared to TFs. Although miRNAs like TFs can exert a widespread impact on gene expression, miRNAs act hierarchically downstream from TFs because they can only repress an mRNA after it has already been transcribed. Furthermore, while TFs bind abundantly in the genome and can regulate several hundred to thousands of genes (Lopez-Pajares et al. 2015; Goolam et al. 2020), miRNAs repress only 200 transcripts on average (Lewis et al. 2005), which is also consistent with our results.

miRNAs typically inhibit gene activity. However, despite the lack of conserved binding sites for the studied miRNAs, we observed upregulation or altered expression of certain genes after miRNA transfection. This suggests that suppression of inhibitory genes may have activated downstream signaling cascades. Notably, each miRNA significantly increased the expression of the *MET* gene. From this observation, we conclude that at the end of an acute inflammation, the investigated miRNAs bring together complex regulatory networks within the gingiva, leading to an increased expression of the gene *MET*. This suggests a relevant role for *MET* in the disease stage at the time the gingival tissue was harvested. Furthermore, we also found that the genes *DDX3Y* and *SLC36A1* were also very strongly upregulated by 4 and *RBPJ* by 3 different miRNAs. Such a cluster is uncommon and, unless an artifact, indicates biological importance. To exclude an artifact, we validated the RNA-seq data by qRT-PCR as an alternative technical method. In addition, we retransfected each miRNA separately and performed protein blotting. This proved increased *MET* expression after miRNA transfection on the protein level. The validation of significant downregulation of these genes by all or multiple miRNAs indicated biological importance. *MET* was described as the cell-surface receptor for hepatocyte growth factor (HGF) (Bottaro et al. 1991). HGF, secreted by fibroblasts, exhibits angiogenic and mitogenic effects on epithelial and endothelial cells. *MET* may play a role in the transition from active inflammation to tissue regeneration, which coincides with the timing of tissue sampling before periodontal surgery, typically performed after active inflammation subsides. During this transition, granulation tissue forms, characterized by proliferating fibroblasts, extracellular matrix remodeling, and sprouting angiogenesis. GSEA results indicated that the selected miRNAs regulate these processes. Interestingly, the functions of *DDX3Y*, *SLC36A1*, and *RBPJ* are less easily ascribed to a possible disease context.

In conclusion, our study identifies target genes of miRNAs that are upregulated during the course of periodontitis. In particular, our results indicate an important role for the gene *MET*, which may be required in the transition from active inflammation to tissue regeneration. Profiling miRNA expression in gingival tissue at different disease states could reveal additional core genes relevant to specific stages of periodontitis and gingival healing, and therefore offer potential therapeutic targets.

## Author Contributions

L. Zheng, contributed to conception, data acquisition, analysis, and interpretation, drafted and critically revised the manuscript; A. Chopra, contributed to data acquisition and analysis, critically revised the manuscript; J. Weiner 3rd, D. Beule, contributed to data analysis and interpretation, critically revised the manuscript; H. Dommisch, contributed to data interpretation, critically revised the manuscript; A.S. Schaefer, contributed to conception and design, data analysis and interpretation, drafted and critically revised the manuscript. All authors gave final approval and agree to be accountable for all aspects of the work.

## Supplemental Material

sj-docx-1-jdr-10.1177_00220345231197984 – Supplemental material for miRNAs from Inflamed Gingiva Link Gene Signaling to Increased MET Expression Click here for additional data file. Supplemental material, sj-docx-1-jdr-10.1177_00220345231197984 for miRNAs from Inflamed Gingiva Link Gene Signaling to Increased MET Expression by L. Zheng, A. Chopra, J. Weiner, D. Beule, H. Dommisch and A. S. Schaefer in Journal of Dental Research
