# Supplementary material for: miRNAs from Inflamed Gingiva Link Gene Signaling to Increased MET Expression
Source: J Dent Res. 2023 Oct 11;102(13):1488–97. doi: 10.1177/00220345231197984 (PMC10683346; doi:10.1177/00220345231197984)
Supplement: sj-docx-1-jdr-10.1177_00220345231197984 – Supplemental material for miRNAs from Inflamed Gingiva Link Gene Signaling to Increased MET Expression [file sj-docx-1-jdr-10.1177_00220345231197984.docx]

**Supplementary methods**

**Isolation of primary human gingival fibroblasts(phGFs)**

Primary gingival cells were cultured from fresh biopsies immediately. Fresh tissue was transferred into DMEM with 2× Penicillin-Streptomycin for 30 minutes to eliminate bacteria. To separate the epithelial layer from the lamina propria, the biopsies were incubated in 10ml DMEM with Dispase II (5mg/ml) on ice in a 4°C refrigerator overnight. Primary epithelial cells were detached from the epithelial layer of the tissue by 5ml Trypsin/EDTA in a 37°C 300 rpm shaker for 30min and neutralized by 10ml DMEM containing 10% FBS collected by 800 rpm 5min centrifugation. Primary gingival epithelial cells were cultured with Dermalife K keratinocyte Medium complete medium (LL-0007, LIFELINE), with 1× Penicillin-Streptomycin added. Lamina propria part was cut into <1×1 mm pieces by sterile scalpels and cultured with 500ul medium in T 25 cell culture flasks. Primary gingival cells were passaged when reaching 80% confluence. Primary gingival cells were only used before the Sixth passage.

**qRT-PCR primers**

*PTK9* Fwd: AGC TCA ACT ATG TGC AGT TGG AAA, Rev: ACG AGC TGA ATC CTT GGG AA;

GA*PDH*: Fwd: CAA ATT CCA TGG CAC CGT CA, Rev: CCT GCA AAT GAG CCC CAG.

For the quantification of *Luciferase* (*Luc*) activity, RNA was isolated from transfected ihGFs. 500 ng of the total RNA was transcribed into cDNA as described above. Total RNA was treated with DNase I (Roche) followed by RNA precipitation until no traceable band could be detected in a control PCR using primers that annealed to the pGL4.24 plasmid backbone (Fwd: TTC AAC CCA GTC AGC TCC TT, Rev: CAA GAA CTC TGT AGC ACC GC). Differential expression of *Luc*, encoded by the plasmid pGL4.24, was quantified by qRT-PCR with primers that annealed to the *Luc* coding sequence (Fwd: ACG TGC AAA AGA AGC TAC CG, Rev: GGC AAA TGG GAA GTC ACG AA). All qRT-PCR experiments were run with the following settings: initial denaturation at 95℃ for 2 min, 35 cycles of 95℃ for 15 secs and 60℃ for 1 min, and 95℃ for 50 secs of the final extenstion. Ct values of the tested genes were normalized to *GAPDH* Ct values. Fold changes of relative gene expression were calculated by the 2^−ΔΔCt^ method. *PTK9* and *Luc* transcript levels were compared between pGFs that were transfected with the positive and negative control mimics. Differences of transcript levels were calculated with a T-test.

For the quantification of *MET*, *RBPJ, DDX3Y* and *SLC36A1* genes activity, RNA was isolated from transfected phGFs. 500 ng of the total RNA was transcribed into cDNA as described above. Differential expression of *MET*, *RBPJ, DDX3Y* and *SLC36A1* genes, were quantified by qRT-PCR with primers of *MET*, *RBPJ, DDX3Y* and *SLC36A1.*(*MET* Fwd: CCC GAA GTG TAA GCC CAA CT , Rev: TGC ACA ATC AGG CTA CTG GG *, RBPJ* Fwd：CAC TCC TGT GCC TGT GGT AG, Rev: CGG ACC CAT CTC CAA CCT TC ; *DDX3Y* Fwd : GGA CGT GTA GGA AAC CTG GG, Rev: GGC ACC AAA TCC TCC ACT GA ; *SLC36A1* Fwd: GCT GGG ATT CTG CTG TGT CT , Rev : TGG CAG TTA TTG GTG GTC CC). All qRT-PCR experiments were run with the same settings mentioned above. Ct values of the tested genes were normalized to *GAPDH* Ct values. Fold changes of relative gene expression were calculated by the 2^−ΔΔCt^ method. All of these gene transcript levels were compared between phGFs that were transfected with the positive and negative control mimics. Differences of transcript levels were calculated with a T-test.

**RNA-Sequencing (RNA-Seq)**

Reads were aligned to the human genome sequences (build GRCh38.p7) using the STAR aligner v. 2.7.5a (Dobin et al., 2013). Quality control (QC) of the reads was inspected using the multiqc reporting tool (Ewels, Magnusson, Lundin, & Kaller, 2016), including fastqc (available online at http://www.bioinformatics.babraham.ac.uk/projects/fastqc), dupradar (Sayols, Scherzinger, & Klein, 2016), qualimap (Garcia-Alcalde et al., 2012), and RNA-SeqC (DeLuca et al., 2012). Raw counts were extracted using the STAR program. Differential gene expression was analysed as recently described (Chopra et al., 2021). In brief, we used the R package DESeq2 (Love, Huber, & Anders, 2014), version 1.26 and for gene set enrichment analysis we used the CERNO test from the tmod package (Zyla et al., 2019), version 0.46.2, and MSigDB (Liberzon et al., 2015). For the hypergeometric test and the Gene Ontology gene sets, the goseq package, version 1.38 (Young, Wakefield, Smyth, & Oshlack, 2010) was used. The P values of the differently expressed genes were corrected for multiple testing using Benjamini-Hochberg correction. The corrected P-values are given as q values (false discovery rate [FDR]).

To predict target genes of the miRNAs we used the online database miRDB (**version 6.0**) (Chen & Wang, 2020). To identify miRNA binding sites within the 3’UTRs of the predicted miRNA target genes, we used the online program TargetScanHuman (version 7.1). To select regulatory candidate genes, for each miRNA we sorted the set of significantly differentially regulated genes (Padj< 0.05) first by target score > 80. This set was subsequently sorted by ascending log2-FC.

**Cloning of *CPEB1* 3’UTR sequence into the reporter plasmid pGL4.24**

To demonstrate the effect of miRNA regulation on a target gene mechansitically, we selected the periodontitis risk gene *CPEB1* (Cytoplasmic Polyadenylation Element Binding Protein 1) that was downregulated by miR130a-3p. The 3’UTR of *CPEB1* contains 3 conserved 7mer sites that matched the seed region of hsa-miR-130a-3p (**Supplementary Material**). We cloned 920 basepairs (bp) of the *CPEB1* 3'UTR sequence including the miRN binding sites into the reporter vector pGL4.24 (Promega) upstream to the *Luc* polyadenylation site. DNA was amplified by PCR using Phusion High-Fidelity PCR Polymerase (NEB) and genomic DNA (gDNA), which was extracted from pGFs (AllPrep DNA/RNA/miRNA Universal Kit, Qiagen). The sequences were amplified with forward and reverse primers including the XbaI restriction site. *CPEB1-3’UTR*, Fwd: ATT TCT AGA GGC AGG TCA GGC AAG CAG, Rev: CTG TCT AGA CCA CCG AAA AGC AGC CCT (XbaI site underlined). The PCR product was purified using the QIAquick gel extraction kit (QIAGEN) and cloned into pGL4.24 using the XbaI cutting sites. Following *E.coli* amplification, the plasmid was purified using the QIAprep Spin Miniprep Kit (Qiagen).

**Luciferase Reporter gene assays**

The reporter gene assays were analysed using SV40 and hTERT immortalized human gingival fibroblasts (ihGFs; CRL-4061, ATCC). Cells were transfected in 3 biological replicates with modified pGL4.24 and plasmid pRL-SV40 (Promega) as Renilla Luciferase control reporter vector in a ratio of 1:10 using Lipofectamine 2000 transfection reagent (Thermo Fisher Scientific). 4 hours after transfection, the culture medium containing 10% FBS was replaced, and the cells were incubated for further 20 hours at 37°C. Subsequently, cells were transfected in triplicates with the mirVana miRNA mimic miR130A-3p or the mirVana miRNA mimic Negative Control #1 using Lipofectamine RNAiMAX. The ihGFs were washed twice with PBS after additional 24 hours incubation. After cell destruction with Passive Lysis Buffer (Promega), firefly and renilla luciferase activities were quantified using the Dual-Luciferase Stop & Glo Reporter Assay System (Promega) with the Orion II Microplate Luminometer (Berthold Technologies). Relative fold changes (FC) in activities were calculated by the ratio of the average (Firefly/Renilla) from the sample (mirVana miRNA mimic) to the average (Firefly/Renilla) from the control (mirVana miRNA mimic Negative Control #1) to determine relative luciferase activity. Differences of transcript levels were calculated with a T-test using the software GraphPad Prism 6 (GraphPad Software, Inc.).

**Western blotting**

24 hours after plating 3✖10^5^ cells per well in a 6-well plate, the cells were transfected by miRNA mimics for another 24 hours. Once plates were rinsed with cold PBS, RIPA buffer was added for 15 minutes (50 mM Tris-HCl, pH 7.4, 150 mM NaCl, 0.5 mM MgCl_2_, 0.2 mM EGTA, and 1% Triton X-100 with protease inhibitor cocktail (Thermo Scientific). Cells were removed and samples were clarified by centrifugation for 15 minutes at 14,800 g and 4 °C. According to the manufacturer's instructions, a BCA Protein Assay reagent (Pierce) was used to determine protein concentration. On 4-12% gradient gels, 30μg of lysate was separated by SDS-PAGE, and proteins were transferred to PVDF membranes. The membranes were blocked in 5% BSA (Rockland) for 1 hour, and the primary antibodies were added overnight at 4 °C (all at a dilution of 1 : 2,000). Antibodies were used as follows: anti-β-actin (C4) (Santa Cruz, sc-47778), anti-Met (25H2) (CST 3127)，anti-CPEB1(Ab3465), Anti-mouse IgG (CTS 7076P2)，anti-Rabbit IgG (Ab 205718). The membranes were rinsed 3x for 10 minutes with 1x TBST before secondary antibodies were added for one hour at room temperature. After three times of treatment in 1x TBST and exposure to ECL western blotting substrate (Pierce，Thermo Scientific), the membranes were analysed (Touch ECL & Fluorescence Imager (CHEMOSTAR).

**Supplementary References**

Chen, Y., & Wang, X. (2020). miRDB: an online database for prediction of functional microRNA targets. *Nucleic Acids Res, 48*(D1), D127-D131. doi:10.1093/nar/gkz757

Chopra, A., Mueller, R., Weiner, J., 3rd, Rosowski, J., Dommisch, H., Grohmann, E., & Schaefer, A. S. (2021). BACH1 Binding Links the Genetic Risk for Severe Periodontitis with ST8SIA1. *J Dent Res*, 220345211017510. doi:10.1177/00220345211017510

DeLuca, D. S., Levin, J. Z., Sivachenko, A., Fennell, T., Nazaire, M. D., Williams, C., . . . Getz, G. (2012). RNA-SeQC: RNA-seq metrics for quality control and process optimization. *Bioinformatics, 28*(11), 1530-1532. doi:10.1093/bioinformatics/bts196

Dobin, A., Davis, C. A., Schlesinger, F., Drenkow, J., Zaleski, C., Jha, S., . . . Gingeras, T. R. (2013). STAR: ultrafast universal RNA-seq aligner. *Bioinformatics, 29*(1), 15-21. doi:10.1093/bioinformatics/bts635

Ewels, P., Magnusson, M., Lundin, S., & Kaller, M. (2016). MultiQC: summarize analysis results for multiple tools and samples in a single report. *Bioinformatics, 32*(19), 3047-3048. doi:10.1093/bioinformatics/btw354

Freitag-Wolf, S., Munz, M., Wiehe, R., Junge, O., Graetz, C., Jockel-Schneider, Y., . . . Schaefer, A. S. (2019). Smoking Modifies the Genetic Risk for Early-Onset Periodontitis. *J Dent Res, 98*(12), 1332-1339. doi:10.1177/0022034519875443

Garcia-Alcalde, F., Okonechnikov, K., Carbonell, J., Cruz, L. M., Gotz, S., Tarazona, S., . . . Conesa, A. (2012). Qualimap: evaluating next-generation sequencing alignment data. *Bioinformatics, 28*(20), 2678-2679. doi:10.1093/bioinformatics/bts503

Liberzon, A., Birger, C., Thorvaldsdottir, H., Ghandi, M., Mesirov, J. P., & Tamayo, P. (2015). The Molecular Signatures Database (MSigDB) hallmark gene set collection. *Cell Syst, 1*(6), 417-425. doi:10.1016/j.cels.2015.12.004

Love, M. I., Huber, W., & Anders, S. (2014). Moderated estimation of fold change and dispersion for RNA-seq data with DESeq2. *Genome Biol, 15*(12), 550. doi:10.1186/s13059-014-0550-8

Sayols, S., Scherzinger, D., & Klein, H. (2016). dupRadar: a Bioconductor package for the assessment of PCR artifacts in RNA-Seq data. *BMC Bioinformatics, 17*(1), 428. doi:10.1186/s12859-016-1276-2

Young, M. D., Wakefield, M. J., Smyth, G. K., & Oshlack, A. (2010). Gene ontology analysis for RNA-seq: accounting for selection bias. *Genome Biol, 11*(2), R14. doi:10.1186/gb-2010-11-2-r14

Zyla, J., Marczyk, M., Domaszewska, T., Kaufmann, S. H. E., Polanska, J., & Weiner, J. (2019). Gene set enrichment for reproducible science: comparison of CERNO and eight other algorithms. *Bioinformatics, 35*(24), 5146-5154. doi:10.1093/bioinformatics/btz447

**Supplementary Table 1.** Differentially expressed miRNAs in healthy and inflamed gingival biopsies found in the indicated miRNA array-based studies

| **miRNA** | **(Xie, Shu, Jiang, Liu, & Zhang, 2011)** | **(Fujimori et al., 2019)** | **(Lee et al., 2011)** | **(Stoecklin-Wasmer et al., 2012)** | **(Perri R et al. 2012)** | **( Yorimasa Ogata et al. 2014)** |
| --- | --- | --- | --- | --- | --- | --- |
| hsa-let-7a | 2-5 | - | 9.48 | - | - | - |
| **hsa-miR-130a** | **2-5**  **(-3p and -5p)** | **-** | **18.83**  **(-3p)** | **-** | **4.6/6.4**  **(-3p)** | **-** |
| **hsa-miR-142-3p** | **2-5** | **-** | **-** | **-** | **2.4/5.3** | **2.33** |
| **hsa-miR-17-5p** | **2-5** | - | **2.37** | - | - | - |
| hsa-miR-302b | 2-5 | - | 9.5 | - | - | - |
| **hsa-miR-30e** | **2-5**  **(-3p and -5p)** | **-** | **2.4**  **(-5p)** | *0.66*  (-5p) | **4.4/4.9**  **(-5p)** | - |
| **hsa-miR-144** | **-** | **2.16**  **(-3p)** | **9.5**  **(#)** | **-** | - | - |
| **hsa-miR-223-3p** | - | - | - | **2.53** | **-** | **2.73** |
| hsa-miR-210 | - | - | - | *0.47* | 1.4/2.3 | - |
| **hsa-miR-144*** | **2-5** | **-** | **-** | **-** | **-** | **2.08** |
| hsa-miR-126 | 2-5 | - | - | 1.51 | - | - |

^#^ The original study did not add a suffix to the name of the miRNA or added an *.Per definition, an asterisk following the name indicates the mature species found at low levels from the opposite arm of a hairpin. However, according to miRBase, miR-144-3p and -144-5p have very similar expression levels. Therefore, we keep the original miRNA descriptions of the original studies in this table.

Numbers indicate fold change. The strand orientation is given in parentheses if not shown in the first column. Of the 6 studies, 5 studies (Xie, Shu, Jiang, Liu, & Zhang, 2011 ;Lee et al., 2011; Stoecklin-Wasmer et al., 2012; Perri, Nares, Zhang, Barros, & Offenbacher, 2012; Yorimasa Ogata et al. 2014) obtained inflamed gingival tissues from periodontitis patients diagnosed by probing depth (PD) > 4 mm, and attachment loss ≥ 3 mm. 1 study (Fujimori et al., 2019) collected saliva. One study (Stoecklin-Wasmer et al., 2012) collected both inflammed and healthy gingiva from peridontitis patients. This study and the study of Ogata et al., 2014 and Perri et al., 2012 diagnosed bleeding on probing at the day of tissue collection.


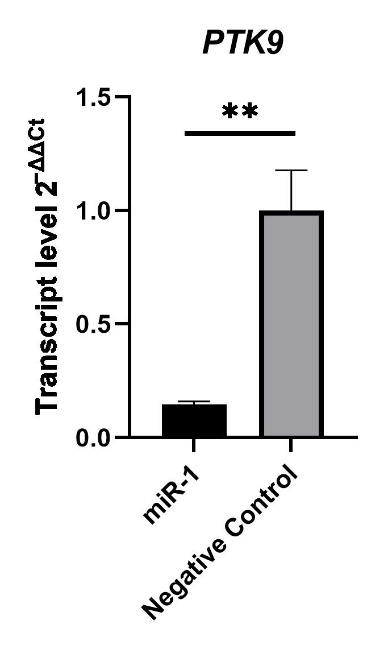


**Supplementary Figure 1**. miR-1 down-regulates PTK9 gene expression in primary human gingival fibroblasts (phGFs). qRT-PCR after transfection of a positive control mimic miR-1 showed reduced *PTK9* expression compared to cells transfected with the negative control mimic (right panels) indicating miRNA function within the transfected cells. (**p < .01)


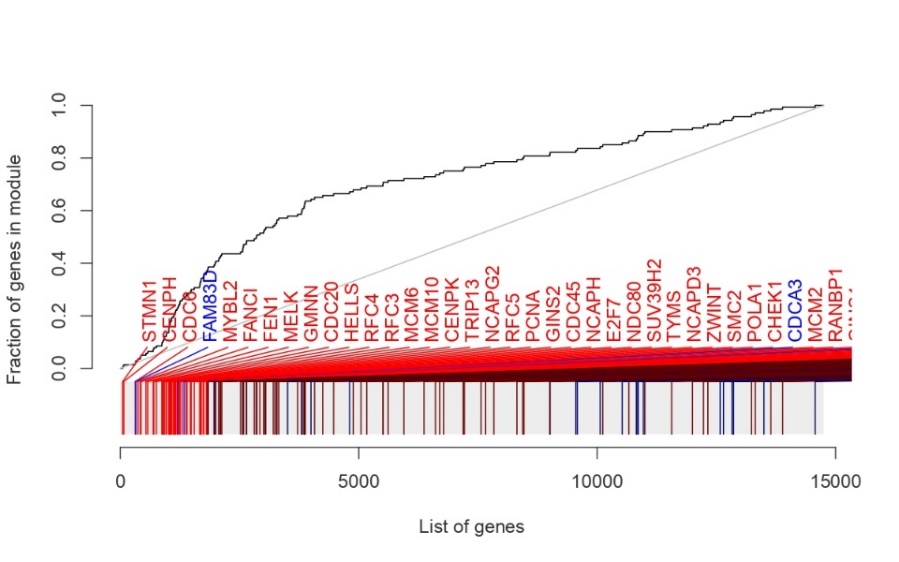
**Supplementary Figure 2 A**. Gene set Cell cycle I (LI.M4.10) was top 1 enriched in the Tmod collection after miR-130a-3p transfection (AUC = 0.7, P_adj_= 9.7 x 10^-9^, 140 genes).


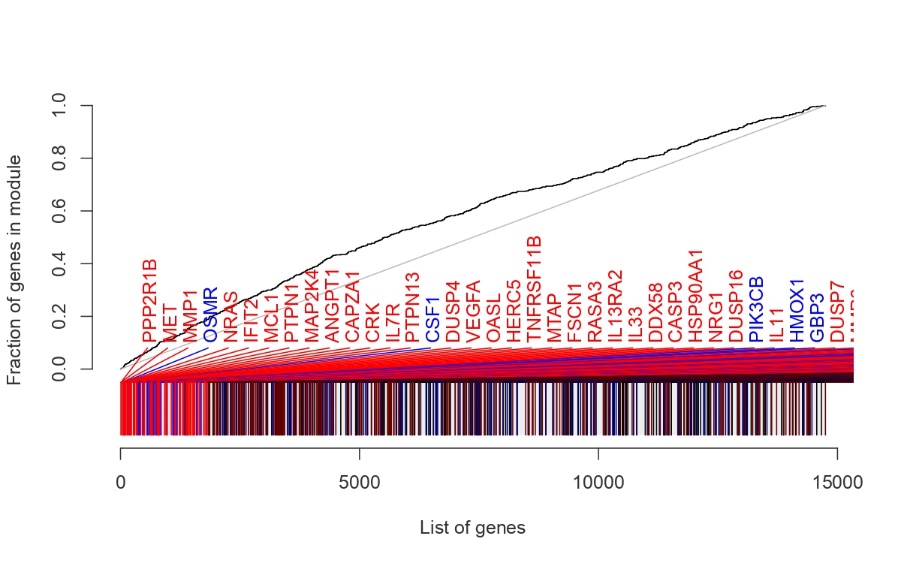


**Supplementary Figure 2 B**. Gene set Cytokine signaling in immune system (M1060) was top 1 enriched in the Reactome collection after miR-130a-3p transfection (AUC = 0.58, P_adj_= 8 x 10^-8^, 660 genes).


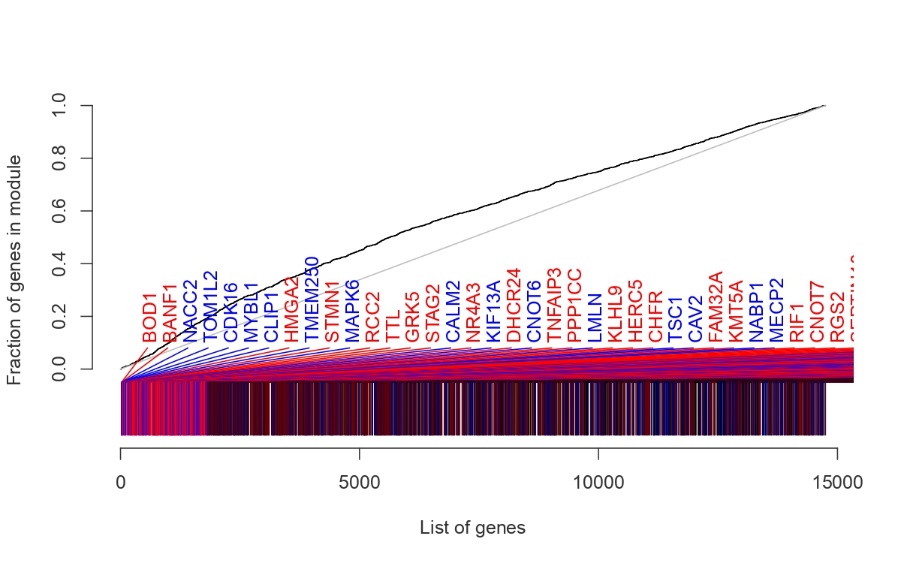


**Supplementary Figure 2 C**. Gene set Cell cycle (M14460) was top 1 enriched in the GO collection after miR-130a-3p transfection (AUC = 0.58, P_adj_= 5.7 x 10^-15^, 1,598 genes).


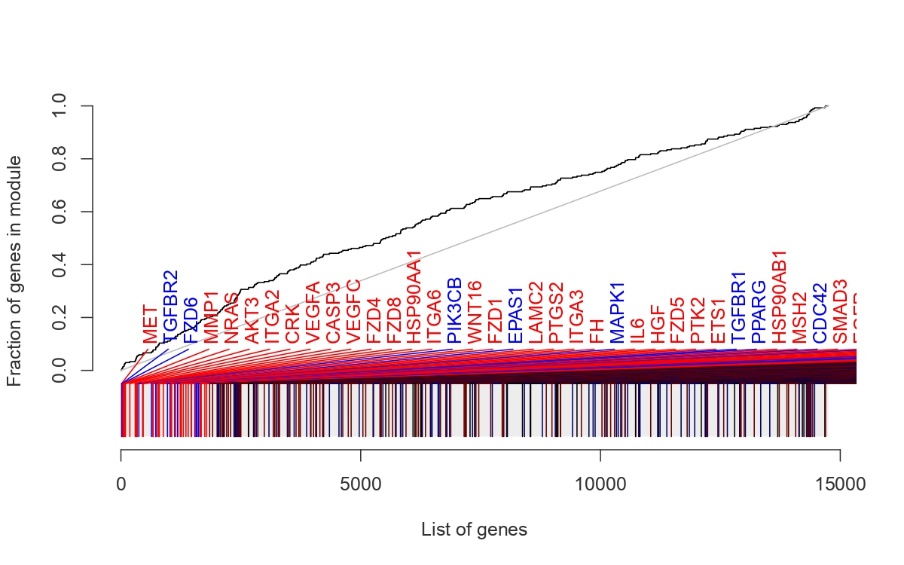


**Supplementary Figure 2 D**. Gene set Pathways in cancer (M12868)) was top 1 enriched in the KEGG collection after miR-130a-3p transfection (AUC = 0.59, P_adj_= 5.3 x 10^-5^, 271 genes).


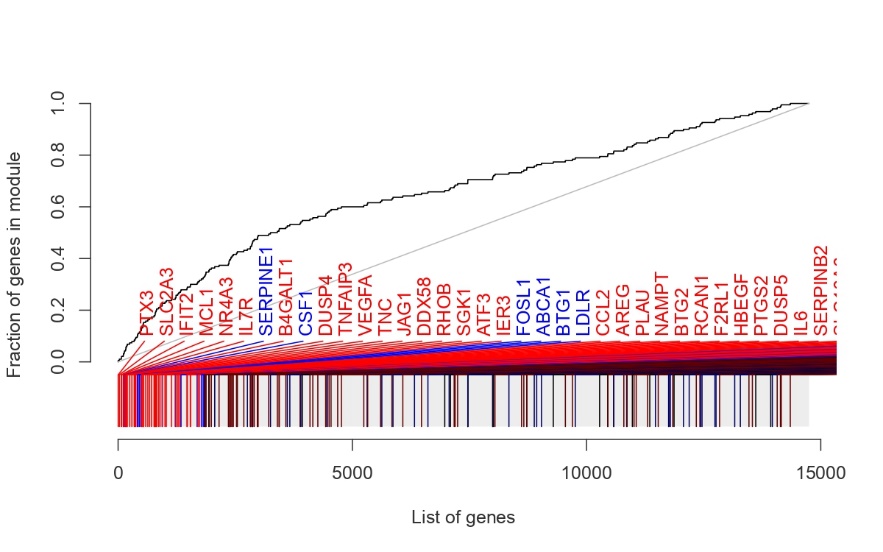
**Supplementary Figure 2 E**. Gene set TNFA signaling via NFKB (M5890) was top 1 enriched in the Hallmark collection after miR-130a-3p transfection (AUC = 0.66, P_adj_= 5.0 x 10^-15^, 190 genes).


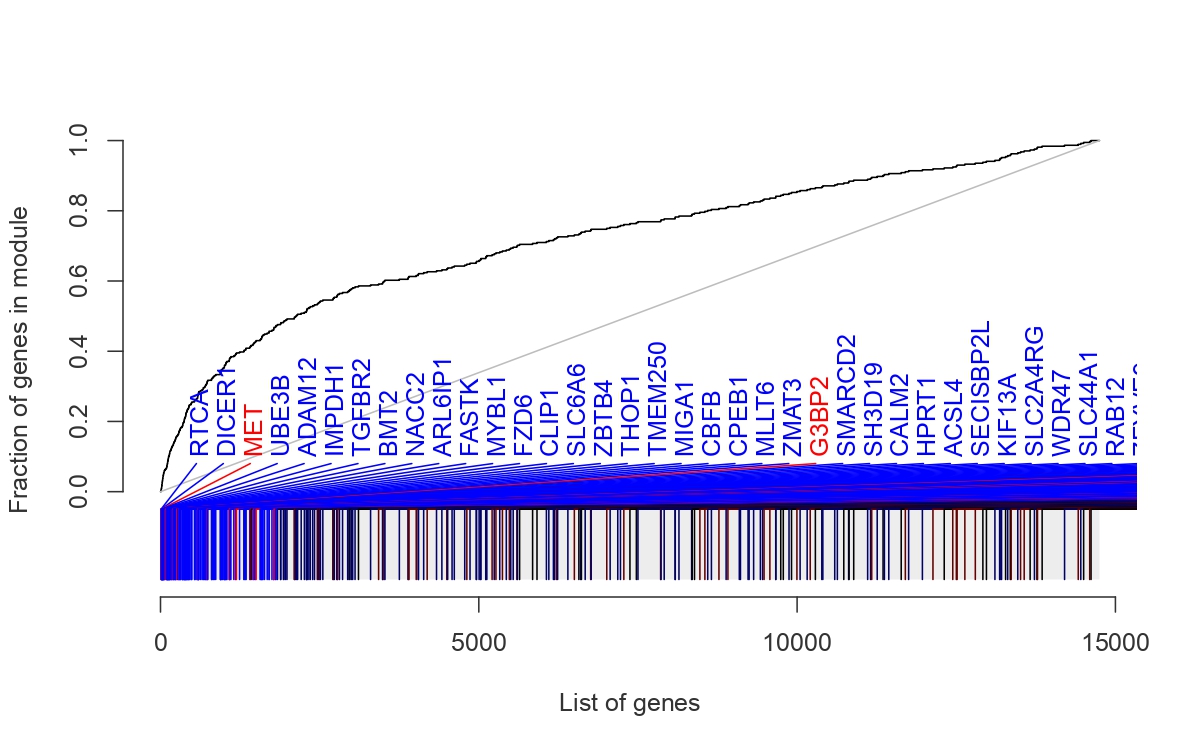


**Supplementary Figure 2 F**. Gene set MIR130A_3P (M30650) was top 1 enriched in the msigdb_mir collection after miR-130a-3p transfection (AUC = 0.73, P_adj_= 9.51 x 10^-69^, 372 genes).


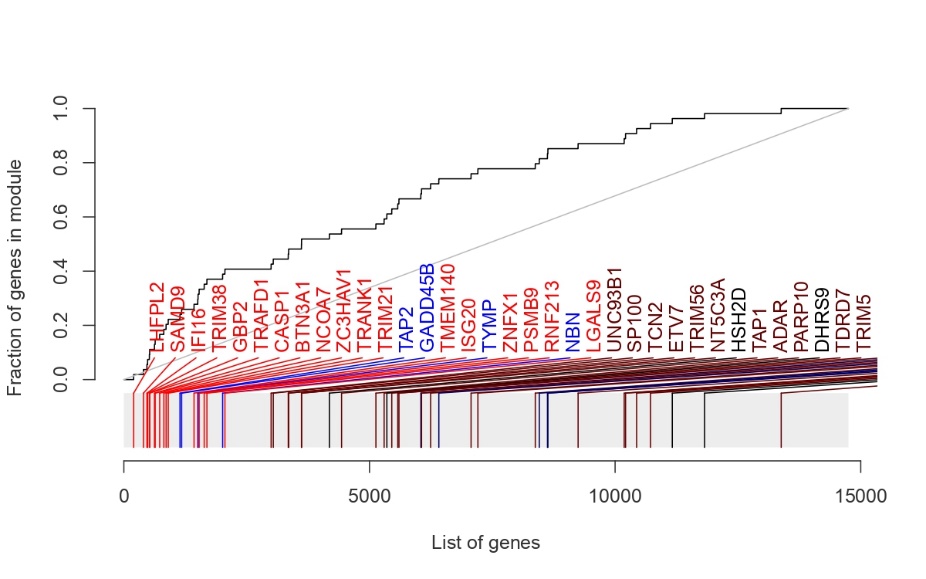
**Supplementary Figure 3 A**. Gene set Interferon (DC.M5.12) was top 1 enriched in the Tmod collection after miR-142-3p transfection (AUC = 0.69, P_adj_= 1.3 x 10^-2^, 54 genes).


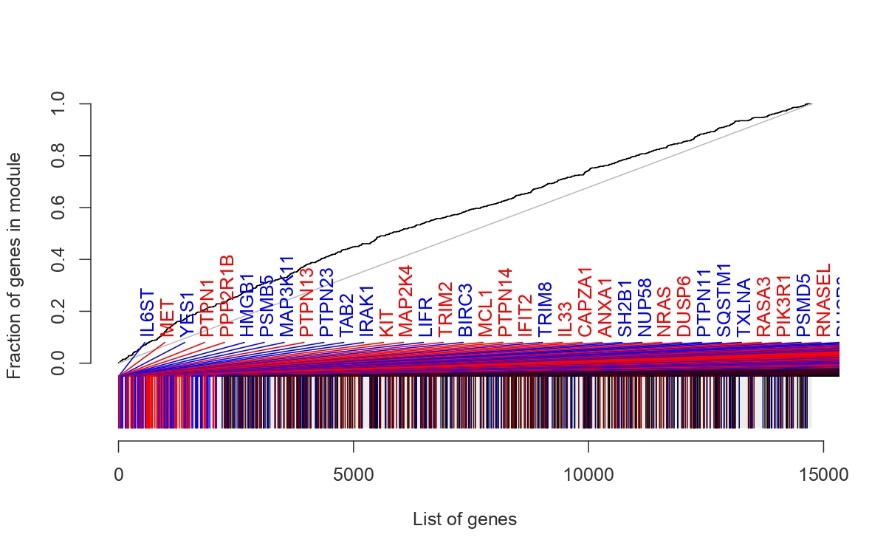


**Supplementary Figure 3 B**. Gene set Cytokine signaling in immune system (M1060) was top 1 enriched in the Reactome collection after miR-142-3p transfection (AUC = 0.57, P_adj_= 1.1 x 10^-7^, 660 genes).


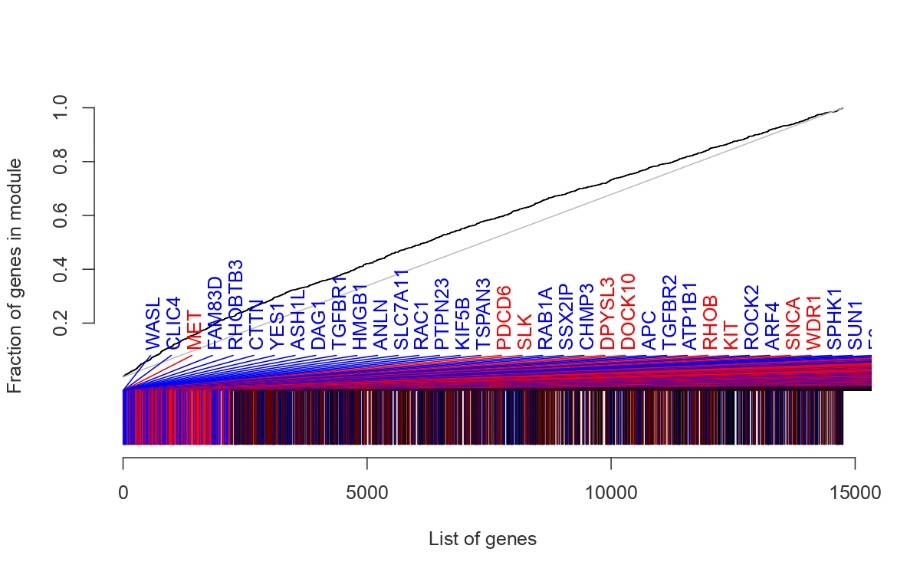


**Supplementary Figure 3 C**. Gene set Locomotion (M13680) was top 1 enriched in the GO collection after miR-142-3p transfection (AUC = 0.56, P_adj_= 1.1 x 10^-11^, 1370 genes).


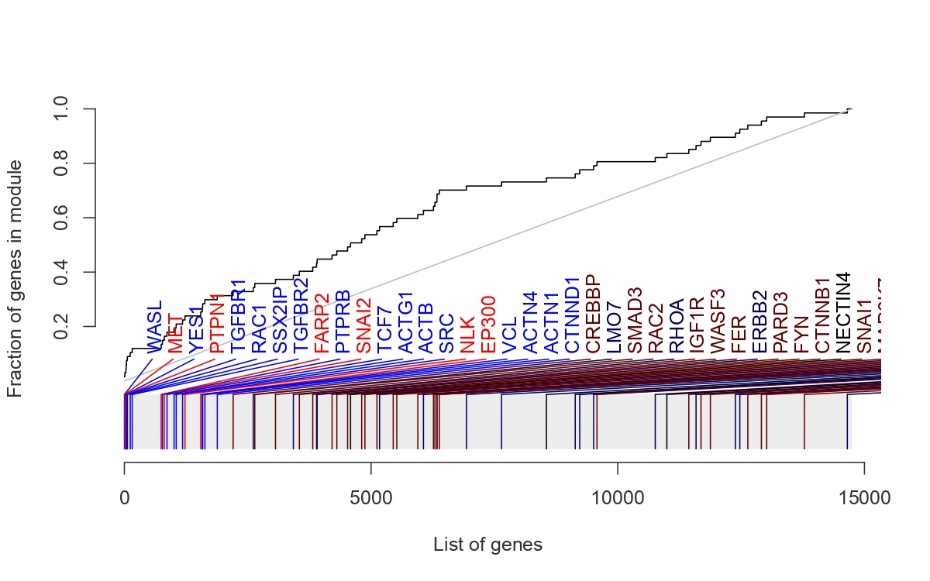


**Supplementary Figure 3 D**. Gene set Adherens Junction (M638) was top 1 enriched in the KEGG collection after miR-142-3p transfection (AUC = 0.64, P_adj_= 1.0 x 10^-5^, 67 genes).


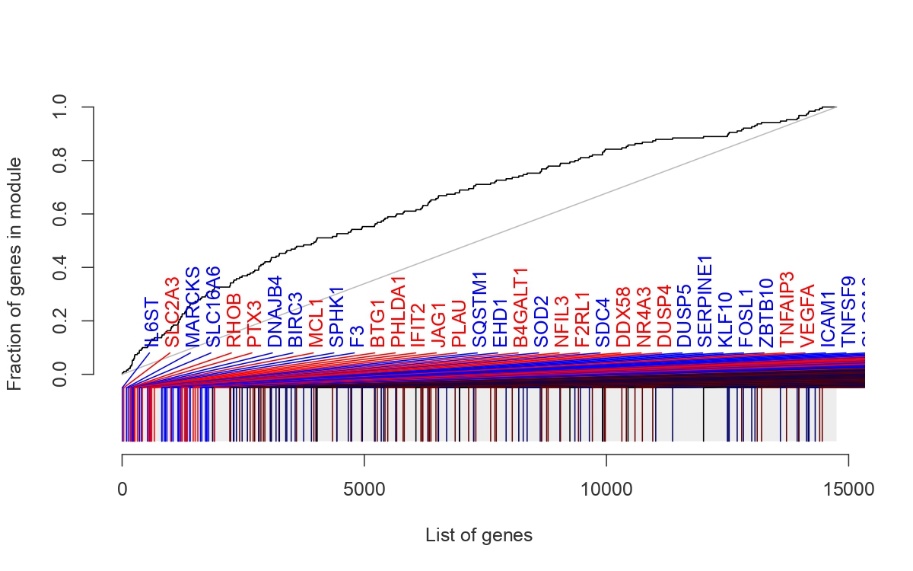


**Supplementary Figure 3 E**. Gene set TNFA signaling via NFKB (M5980) was top 1 enriched in the Hallmark collection after miR-142-3p transfection (AUC = 0.65, P_adj_= 4.7 x 10^-11^, 190 genes).


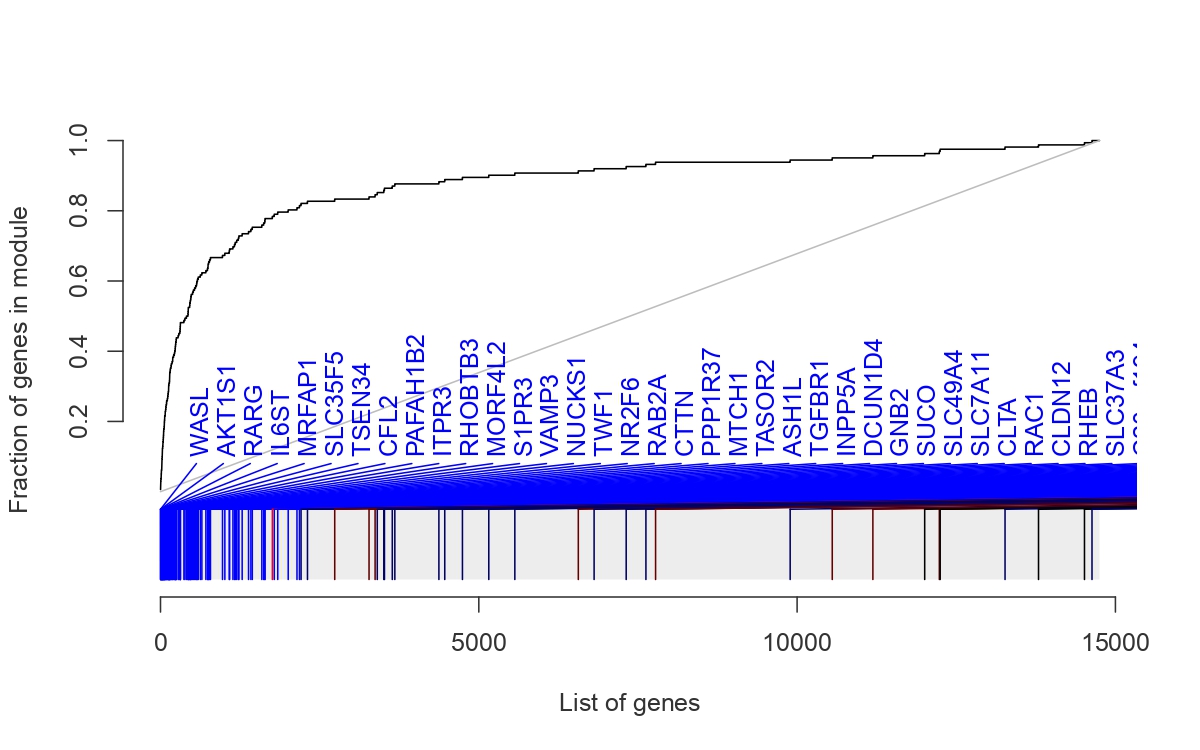


**Supplementary Figure 3 F**. Gene set MIR142-3P (M31116) was top 1 enriched in the msigdb_mir collection after miR-142-3p transfection (AUC = 0.89, P_adj_= 9.4 x 10^-102^, 162 genes).


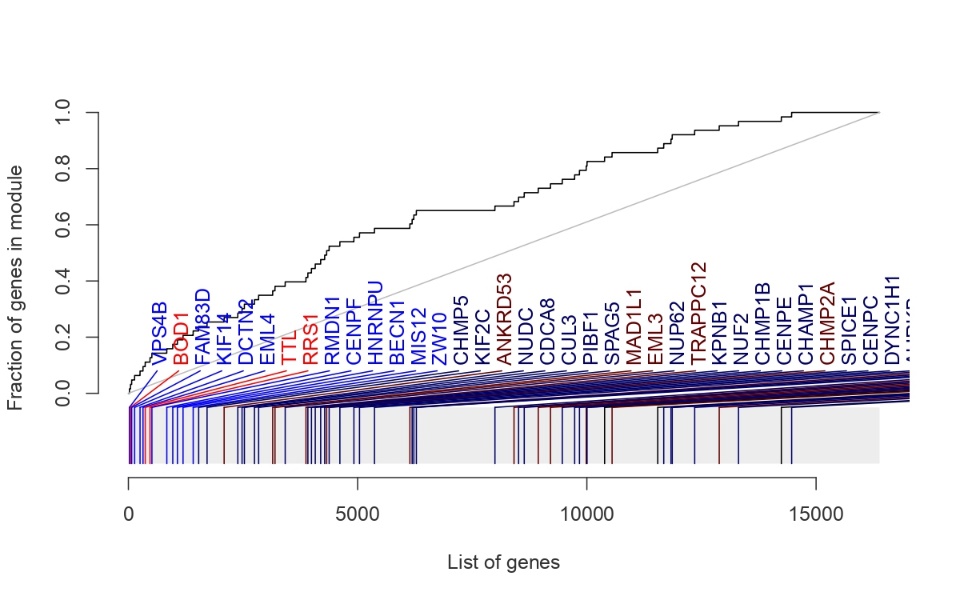


**Supplementary Figure 4 A**. Gene set Metaphase plate congression (M16704) was top 1 enriched in the GO collection after miR-144-3p transfection (AUC = 0.66, P_adj_= 1.5 x 10^-4^, 63 genes).


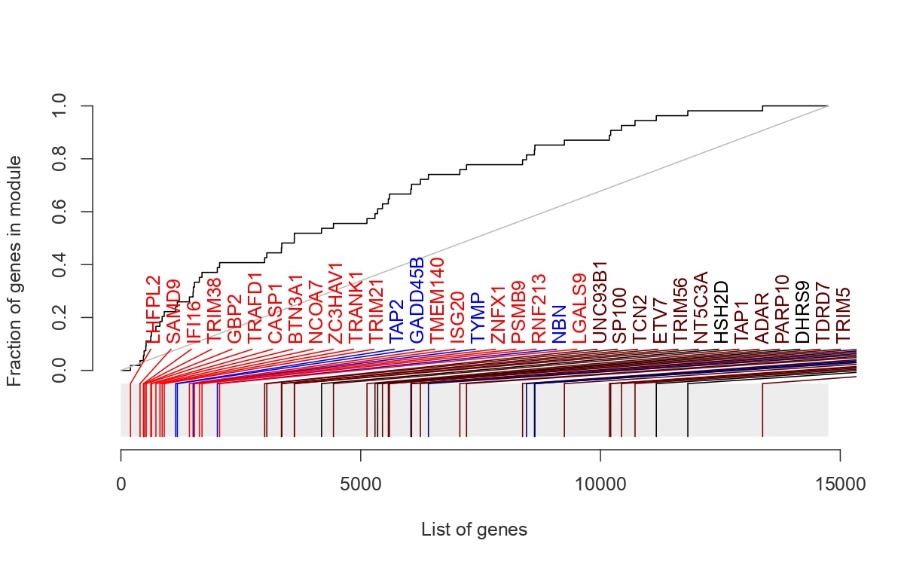


**Supplementary Figure 4 B**. Gene set Extracellular matrix (I) (LI.M2.0) was top 1 enriched in the Tmod collection after miR-144-3p transfection (AUC = 0.77, P_adj_= 2.0 x 10^-3^, 30 genes).


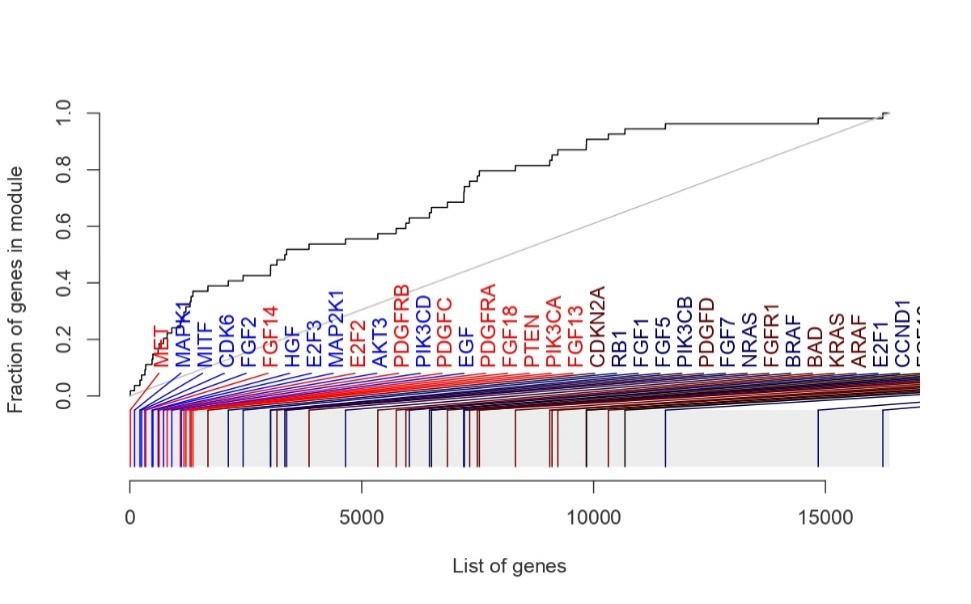


**Supplementary Figure 4 C**. Gene set Melanoma (M15798) was top 1 enriched in the KEGG collection after miR-144-3p transfection (AUC = 0.72, P_adj_= 3.7 x 10^-7^, 54 genes).


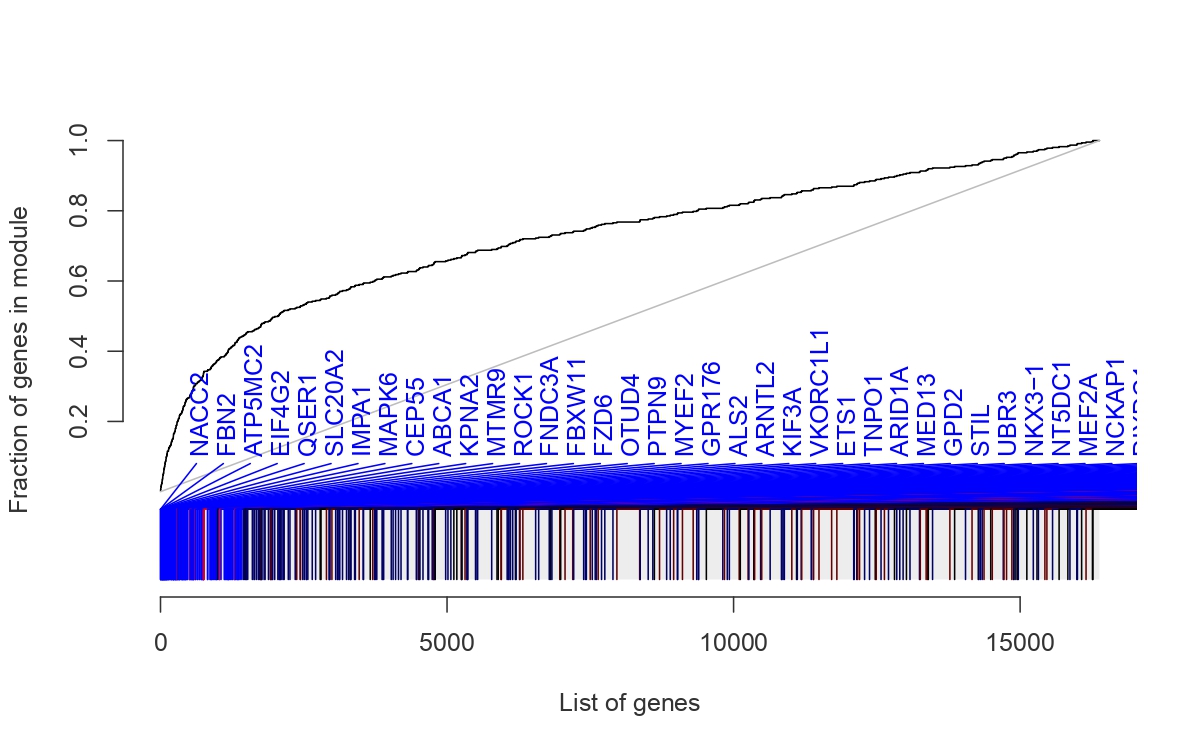


**Supplementary Figure 4 D**. Gene set MIR144-3P (M30504) was top 1 enriched in the msigdb_mir collection after miR-144-3p transfection (AUC = 0.74, P_adj_= 2.1 x 10^-102^, 461 genes).


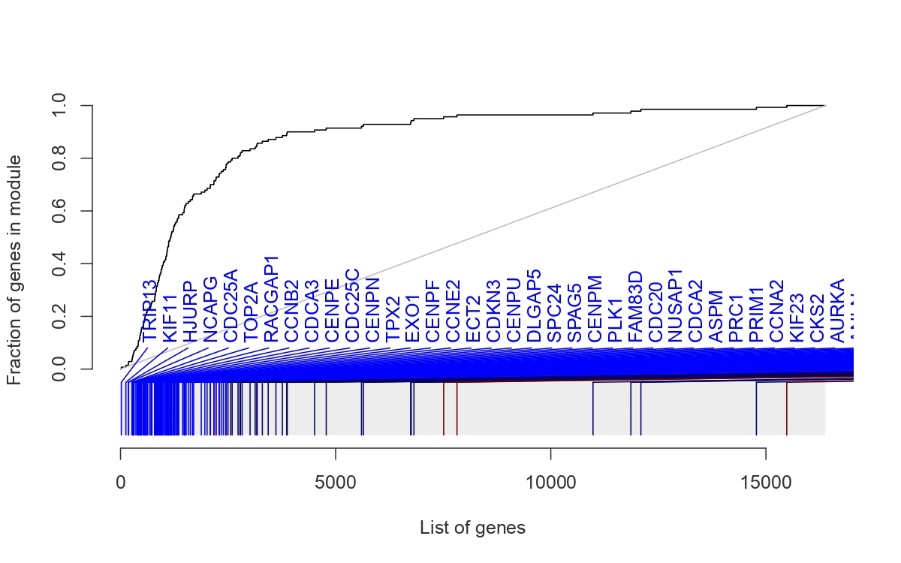


**Supplementary Figure 5 A**. Gene set cell cycle (I) (LI.M4.1) was top 1 enriched in the Tmod collection after miR-144-5p transfection (AUC = 0.88, P_adj_= 3.0 x 10^-37^, 140 genes).


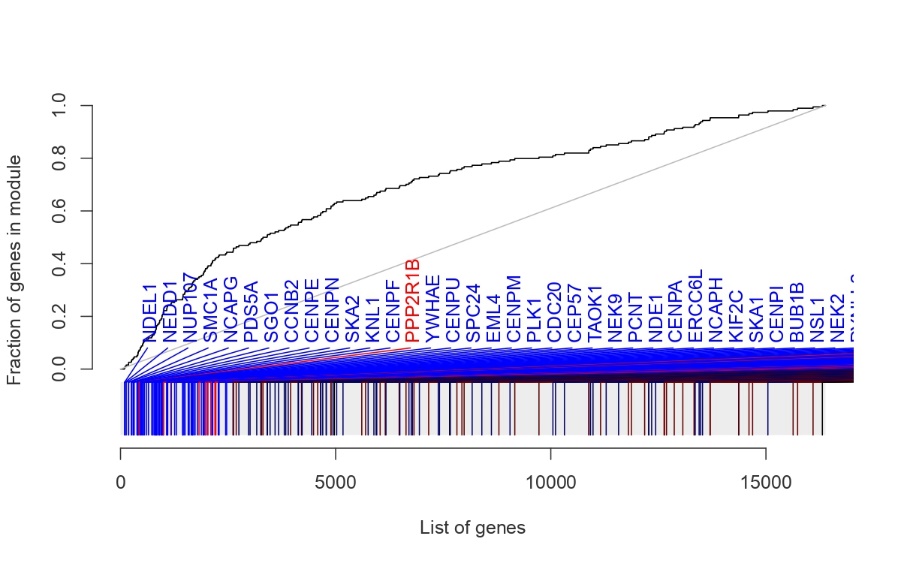


**Supplementary Figure 5 B**. Gene set Mitotic prometaphase (M4217) was top 1 enriched in the Reactome collection after miR-144-5p transfection (AUC = 0.7, P_adj_= 4.6 x 10^-15^, 194 genes).


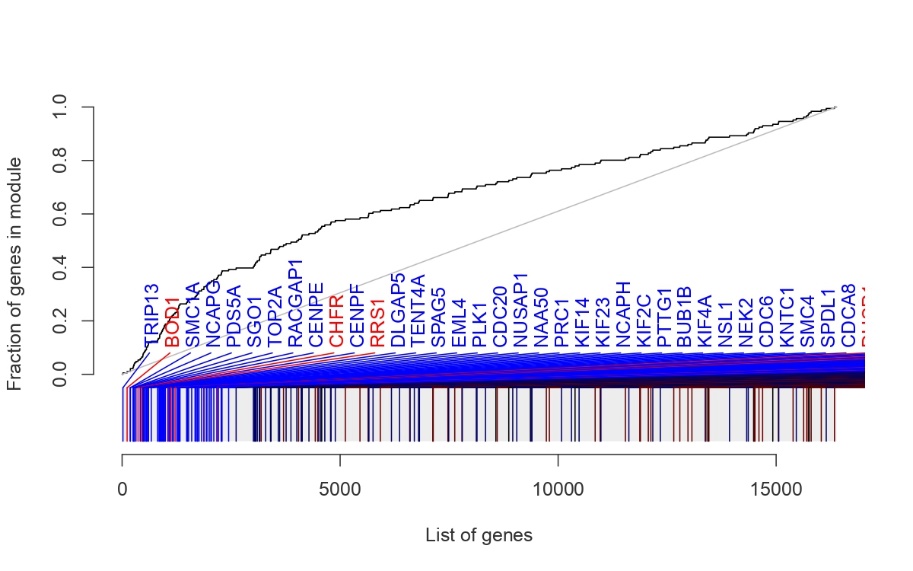


**Supplementary Figure 5 C**. Gene set Sister chromatid segregation (M536) was top 1 enriched in the GO collection after miR-144-5p transfection (AUC = 0.65, P_adj_= 2.5 x 10^-10^, 186 genes).


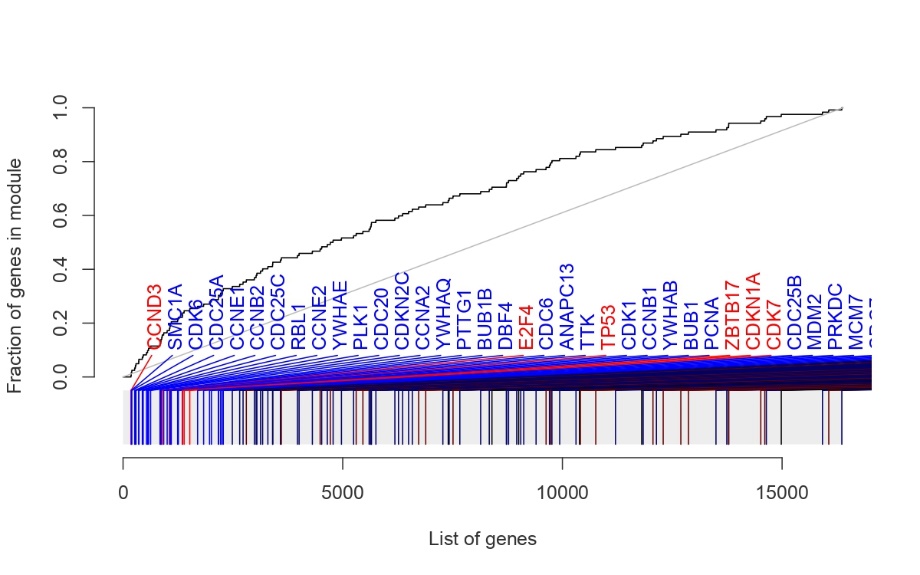


**Supplementary Figure 5 D**. Gene set Cell cycle (M7963) was top 1 enriched in the KEGG collection after miR-144-5p transfection (AUC = 0.65, P_adj_= 2.3x 10^-5^ ,122 genes).


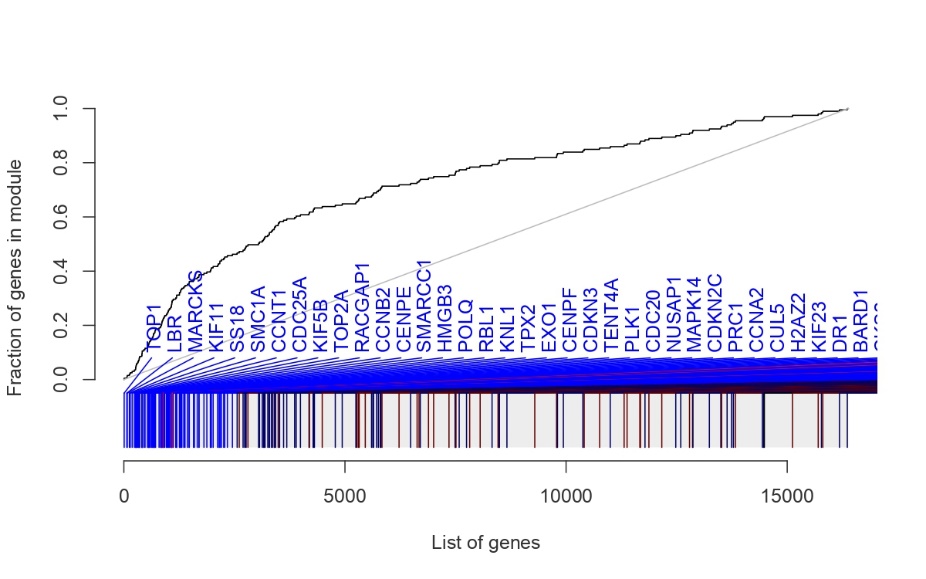


**Supplementary Figure 5 E** Gene set G2M checkpoint (M5901) was top 1 enriched in the KEGG collection after miR-144-5p transfection (AUC = 0.72, P_adj_= 4.8x 10^-23^ ,199 genes).


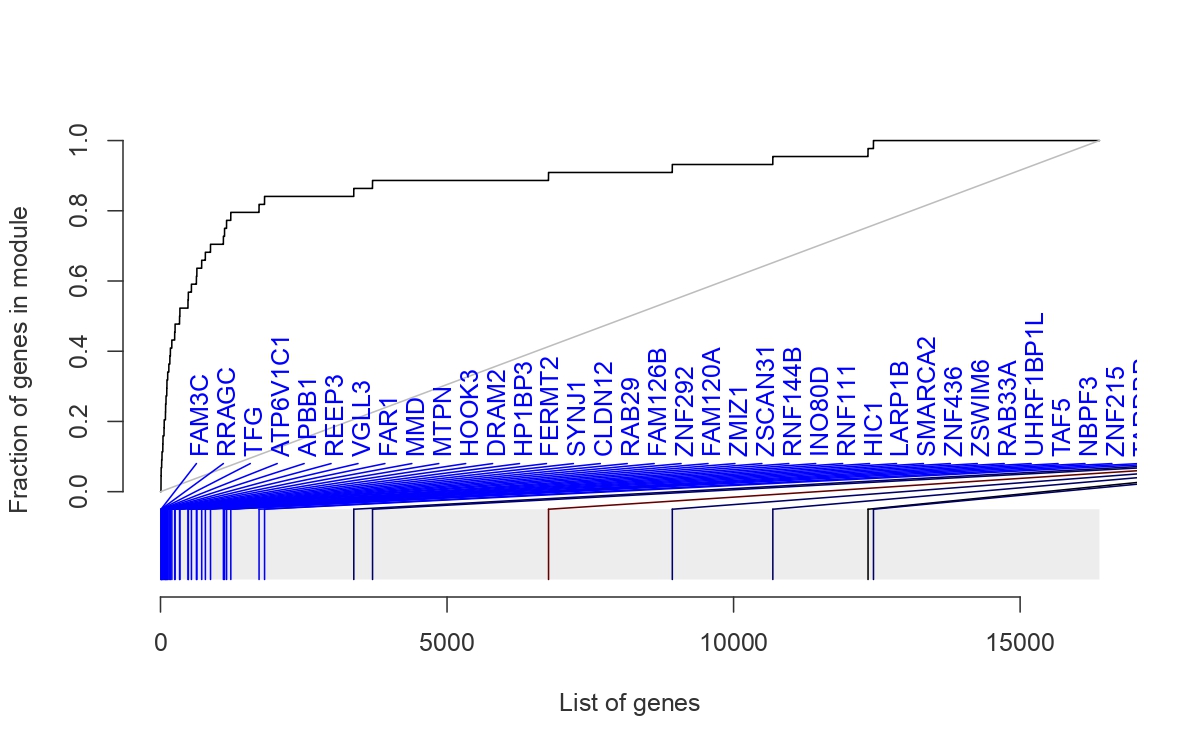


**Supplementary Figure 5 F** Gene set MIR144-5P (M32032) was top 1 enriched in the msigdb_mir collection after miR-144-5p transfection (AUC = 0.9, P_adj_= 7.6x 10^-28^ ,44 genes).


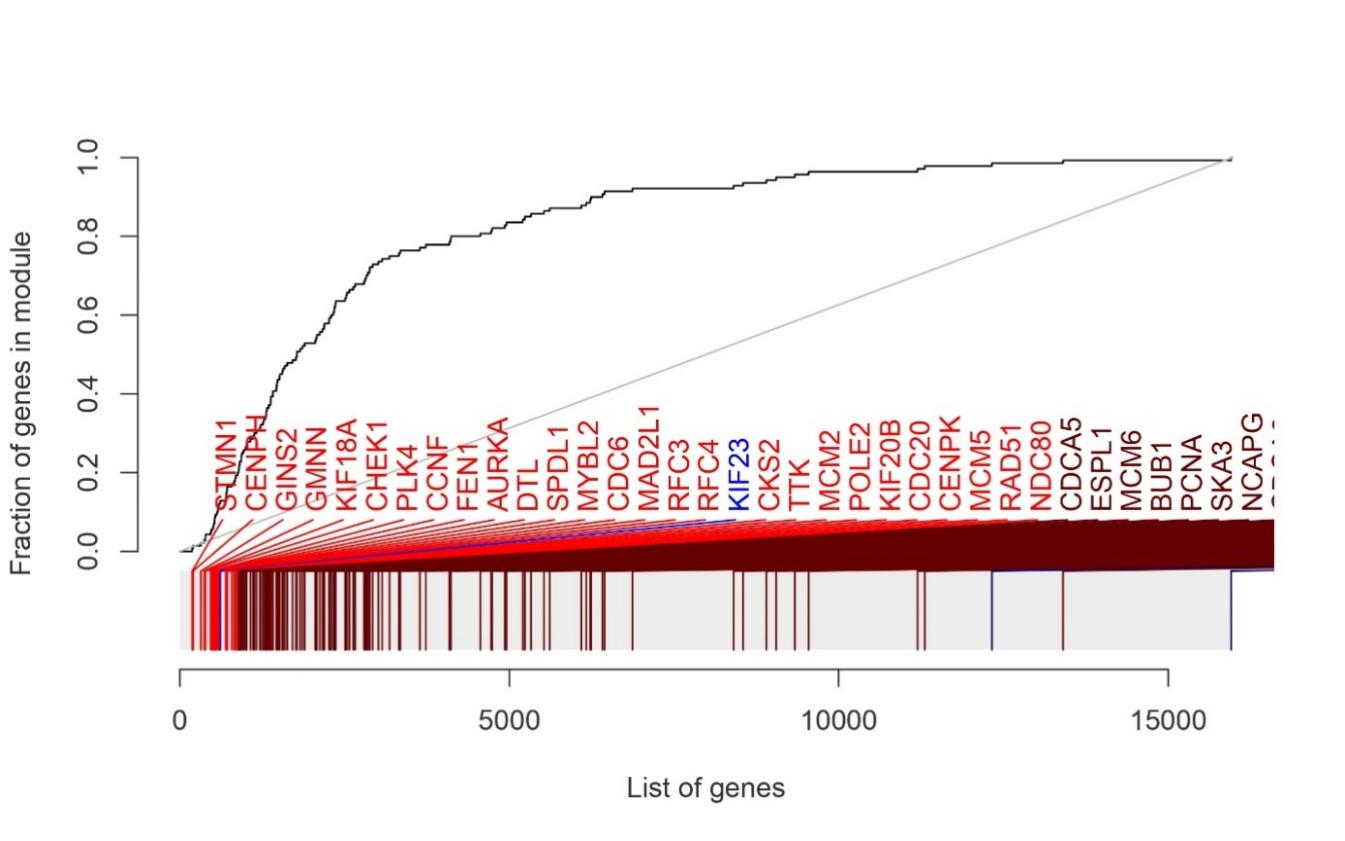


**Supplementary Figure 6 A**. Gene set cell cycle (I) (LI.M4.1) was top 1 enriched in the Tmod collection after miR-17-5p transfection (AUC = 0.83, P_adj_= 9.5 x 10^-23^, 140 genes).


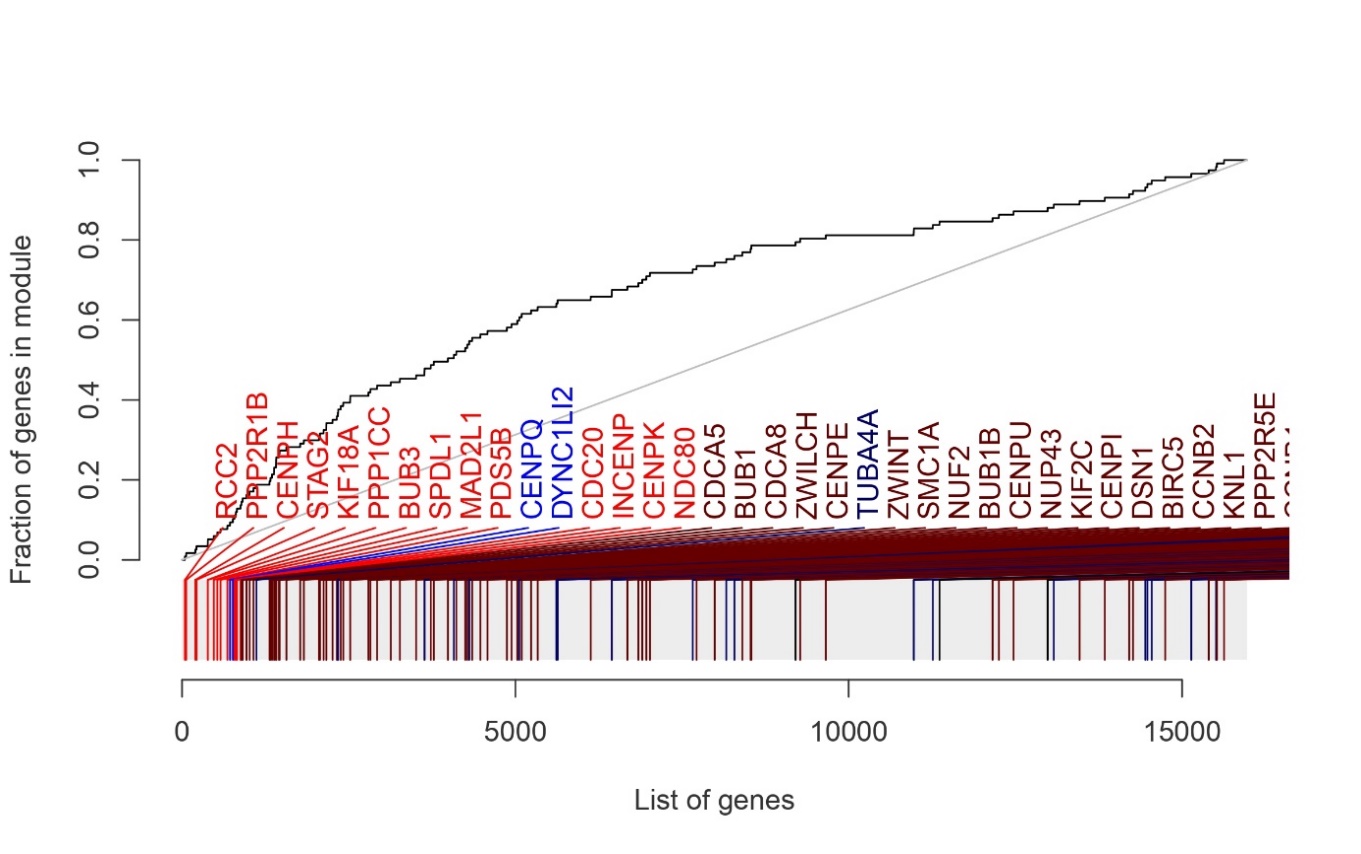


**Supplementary Figure 6 B**. Gene set Resoution of sister chromatid codhsion (M27181) was top 1 enriched in the Reactome collection after miR-17-5p transfection (AUC = 0.67, P_adj_= 4.2 x 10^-6^, 117 genes).


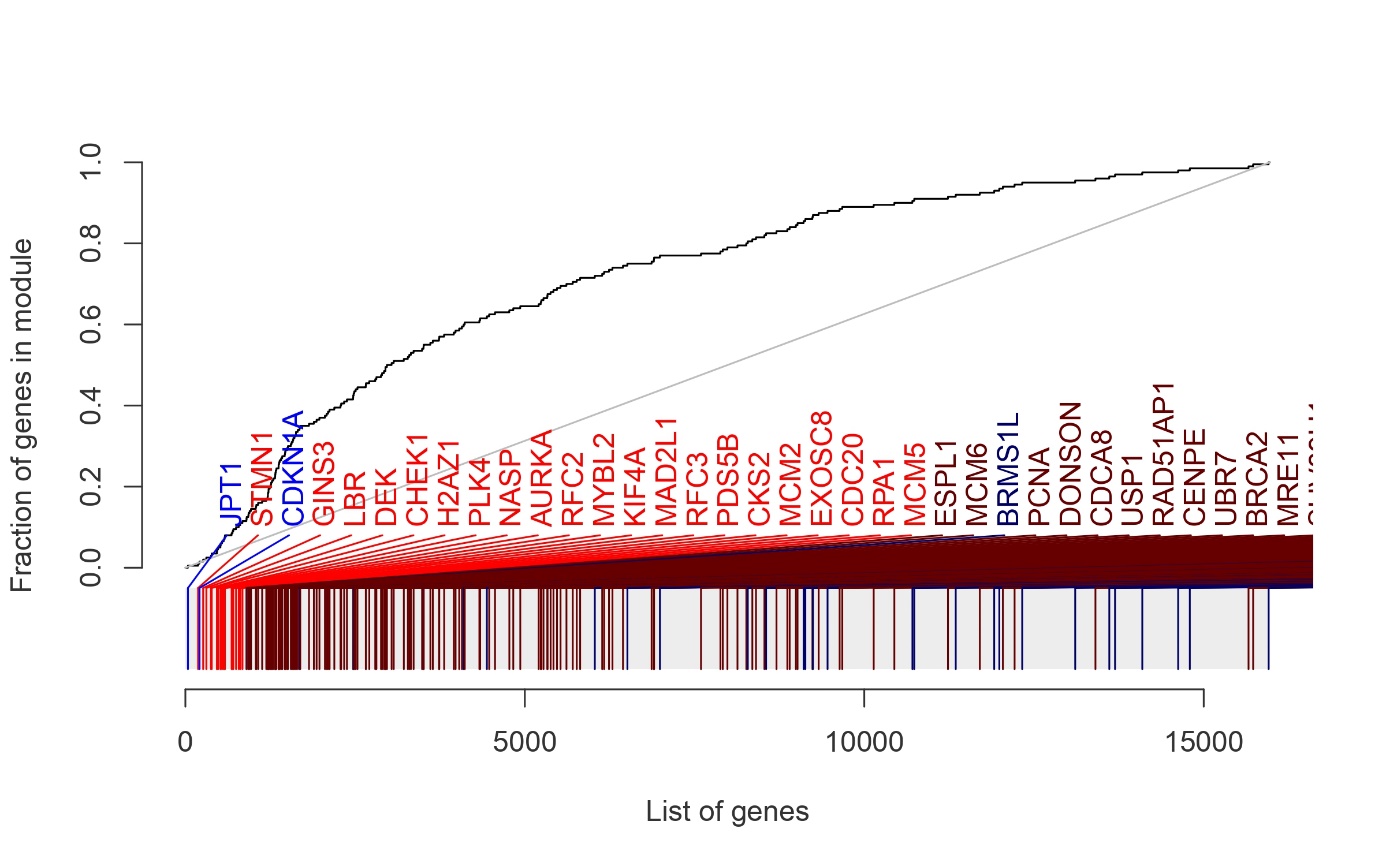


**Supplementary Figure 6 C**. Gene set E2F targets (M5925) was top 1 enriched in the Hallmark collection after miR-17-5p transfection (AUC = 0.72, P_adj_= 8.3 x 10^-16^, 200 genes).


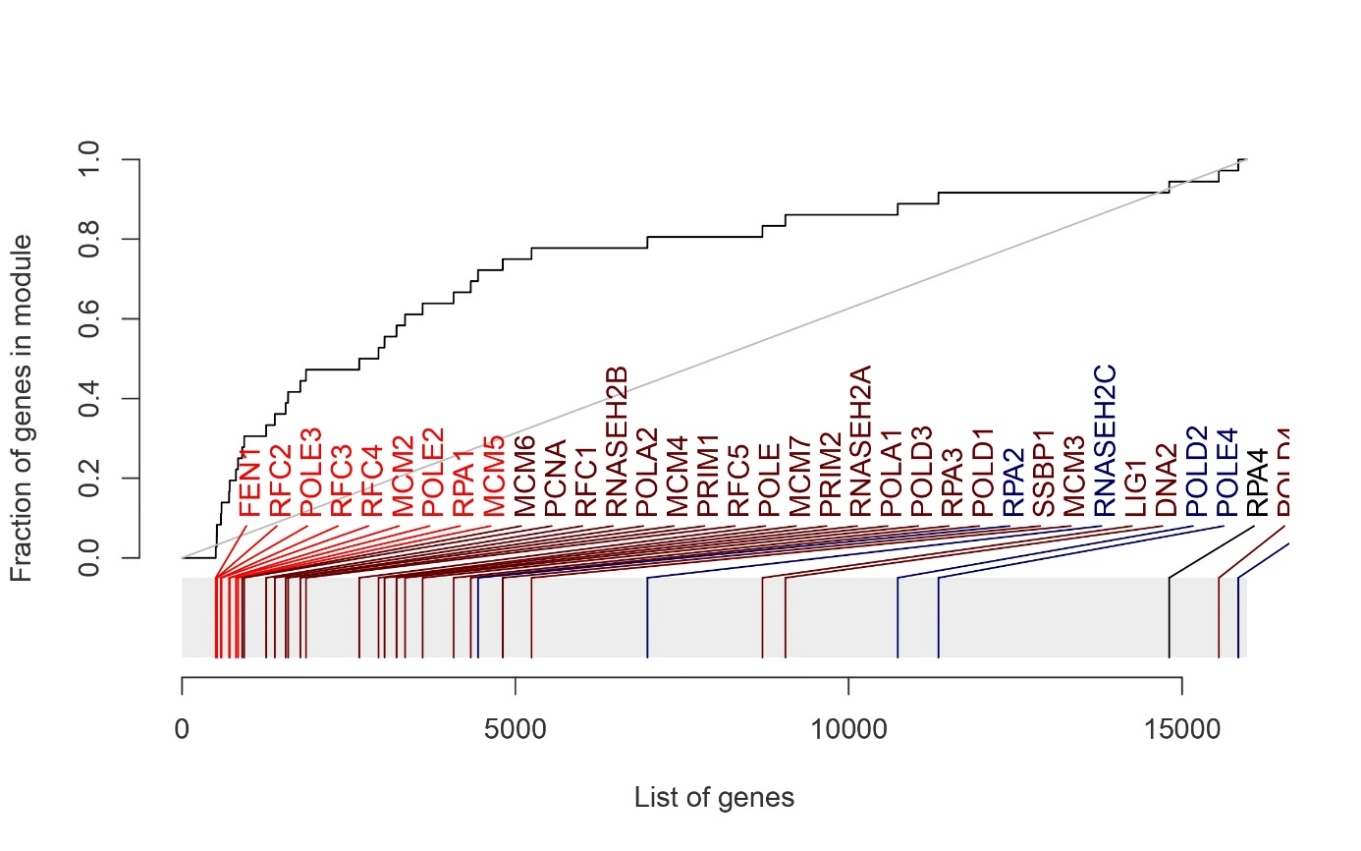


**Supplementary Figure 6 D**. Gene set DNA Replication (M16853) was top 1 enriched in the KEGG collection after miR-17-5p transfection (AUC = 0.74, P_adj_= 5.8 x 10^-4^ ,36 genes).


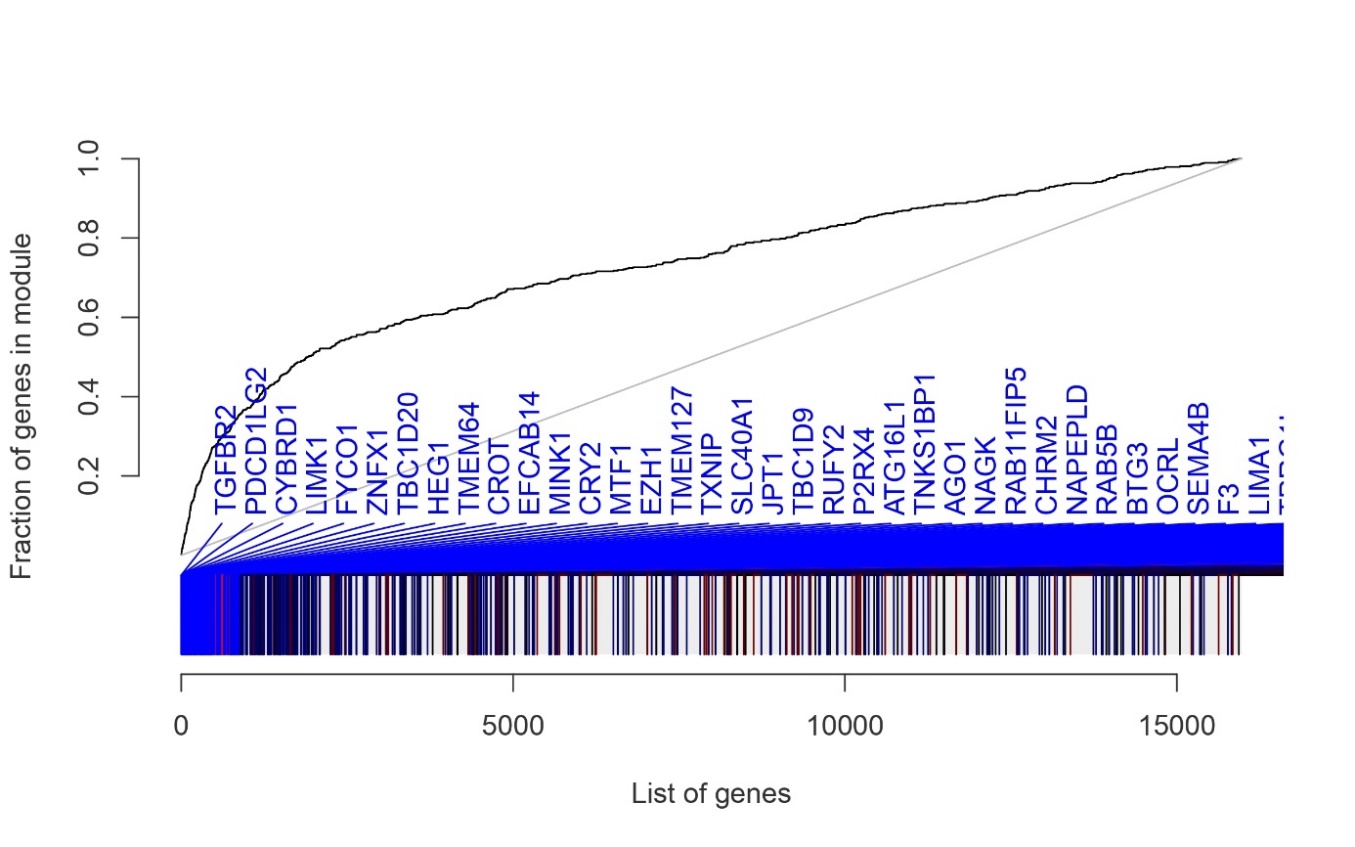


**Supplementary Figure 6 E** Gene set miR-17-5p (M30516) was top 1 enriched in the MIR collection after miR-17-5p transfection (AUC = 0.74, P_adj_= 6.2 x 10^-127^ , 581 genes).


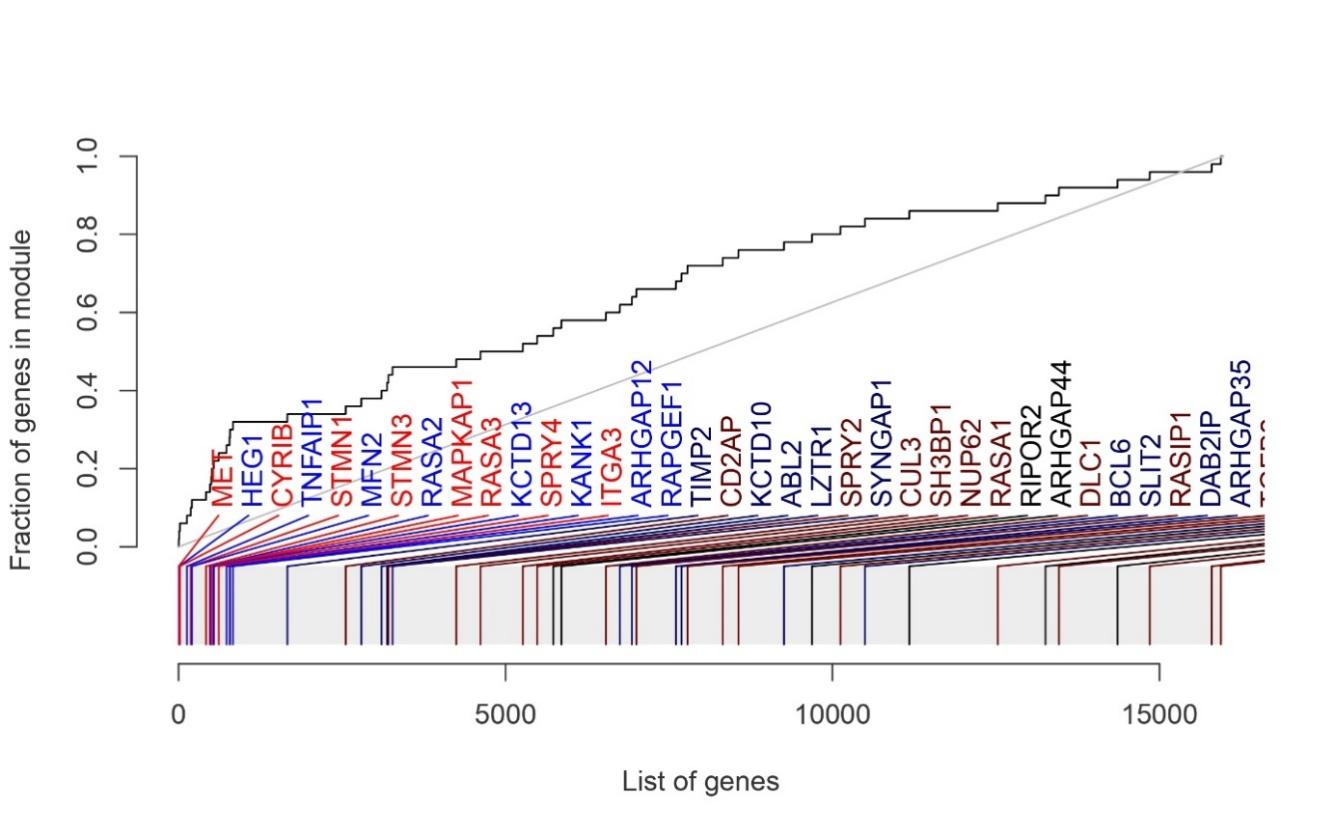


**Supplementary Figure 6 F** Gene set Negative regulation of small GTPASE mediated signal transduction (M11863) was top 1 enriched in the Go collection after miR-17-5p transfection (AUC = 0.66, P_adj_= 8.8 x 10^-6^ , 50 genes).

**
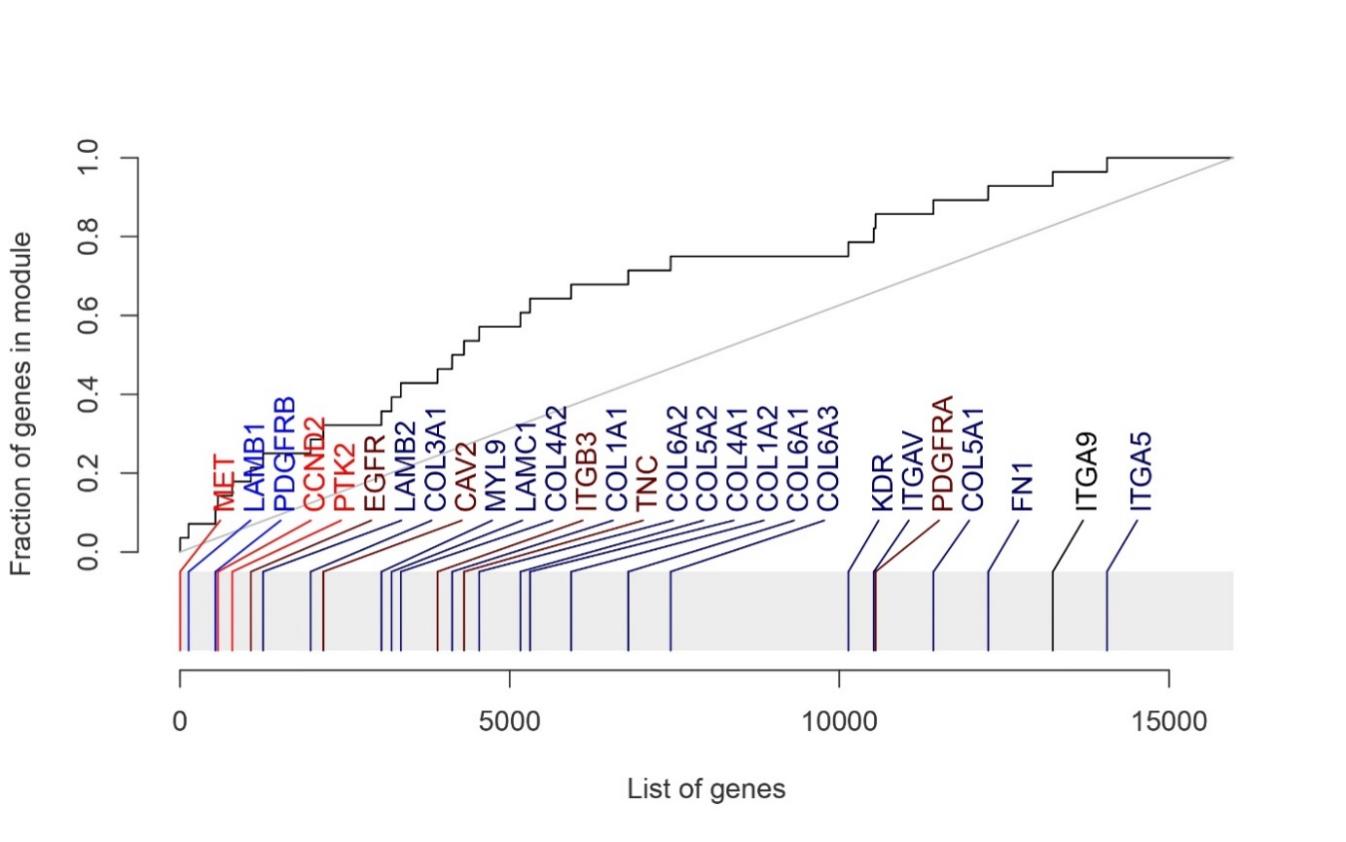
**

**Supplementary Figure 7A**. Gene set integrin cell surface interactions (I) (LI.M1.0) was top 1 enriched in the Tmod collection after miR-223-3p transfection (AUC = 0.67, P_adj_= 3.2 x 10^-2^, 28 genes).


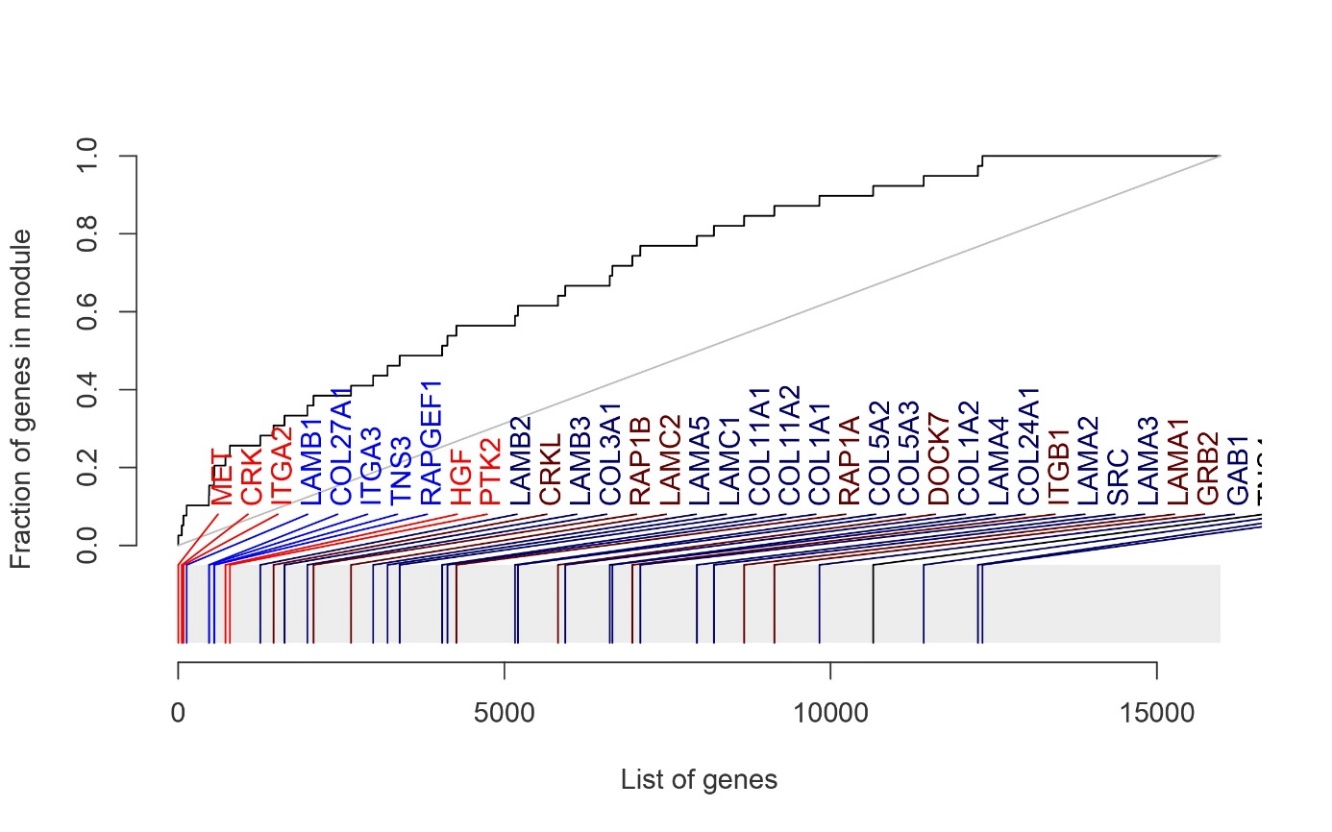


**Supplementary Figure 7B**. Gene set MET promotes cell motility (M27778) was top 1 enriched in the Reactome collection after miR-223-3p transfection (AUC = 0.72, P_adj_= 1.6 x 10^-4^, 39 genes).


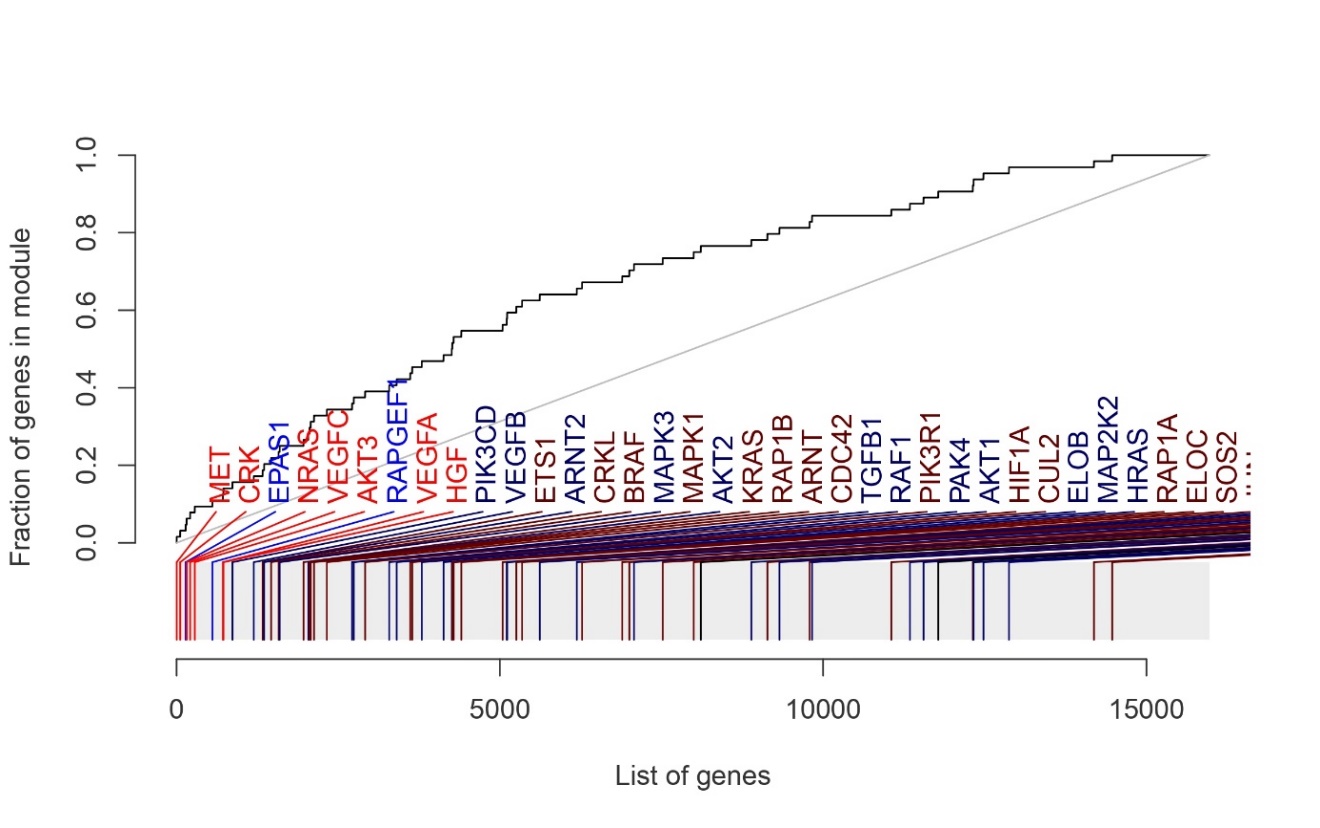


**Supplementary Figure 7C**. Gene set Renal cell carcinoma (M13266) was top 1 enriched in the Kegg collection after miR-223-3p transfection (AUC = 0.68, P_adj_= 4.8 x 10^-5^, 64 genes).


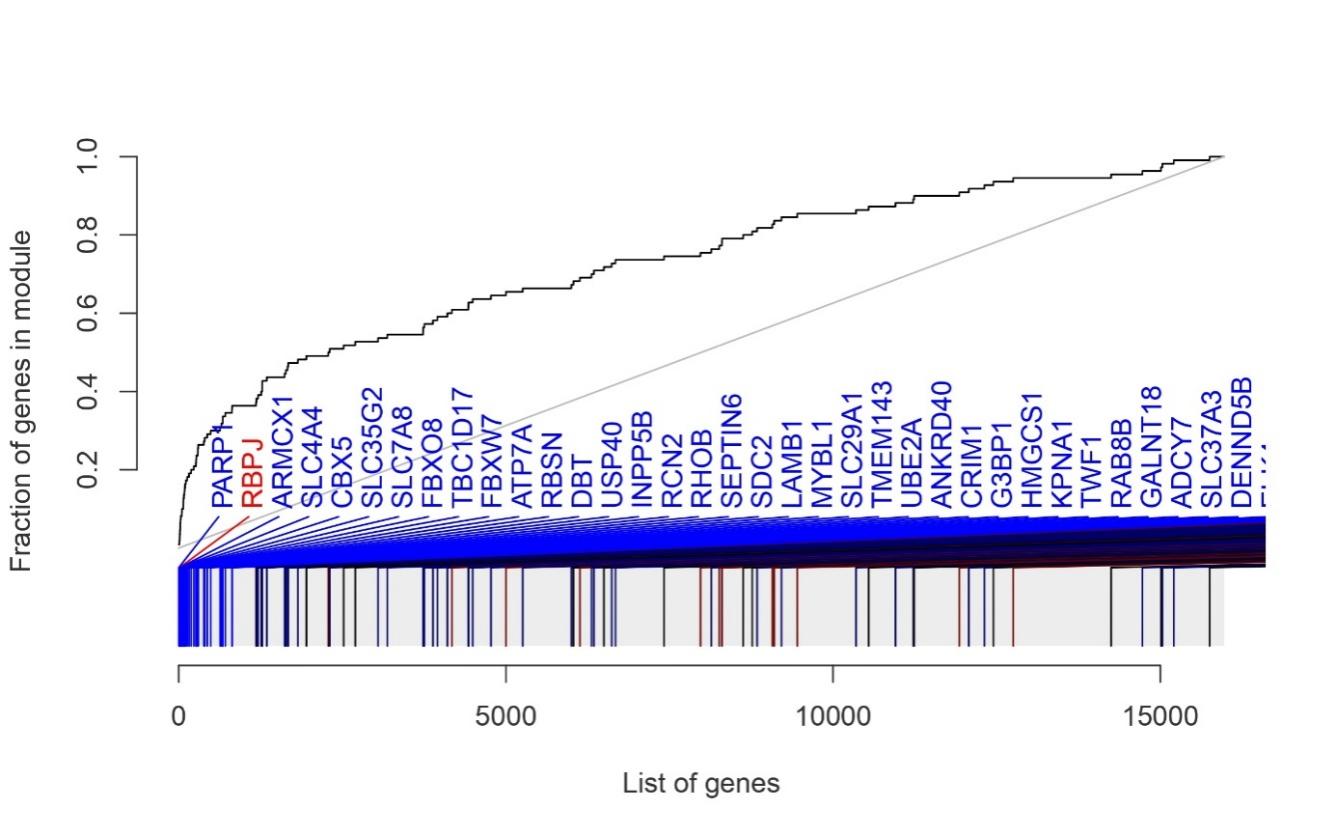


**Supplementary Figure 7D**. Gene set MIR-223-3P (M31334) was top 1 enriched in the MIR collection after miR-223-3p transfection (AUC = 0.73, P_adj_= 4.2 x 10^-27^ ,110 genes).


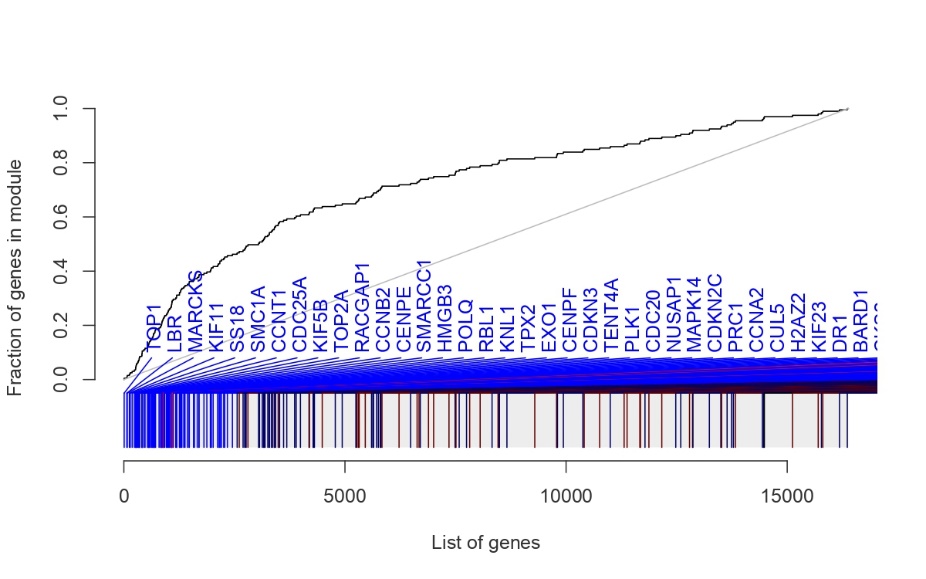


**Supplementary Figure 7 E** Gene set Regulation of protein localization to nucleus(M15292) was top 1 enriched in the GO collection after miR-223-3p transfection (AUC = 0.65, P_adj_= 6.9 x 10^-6^ , 115 genes).


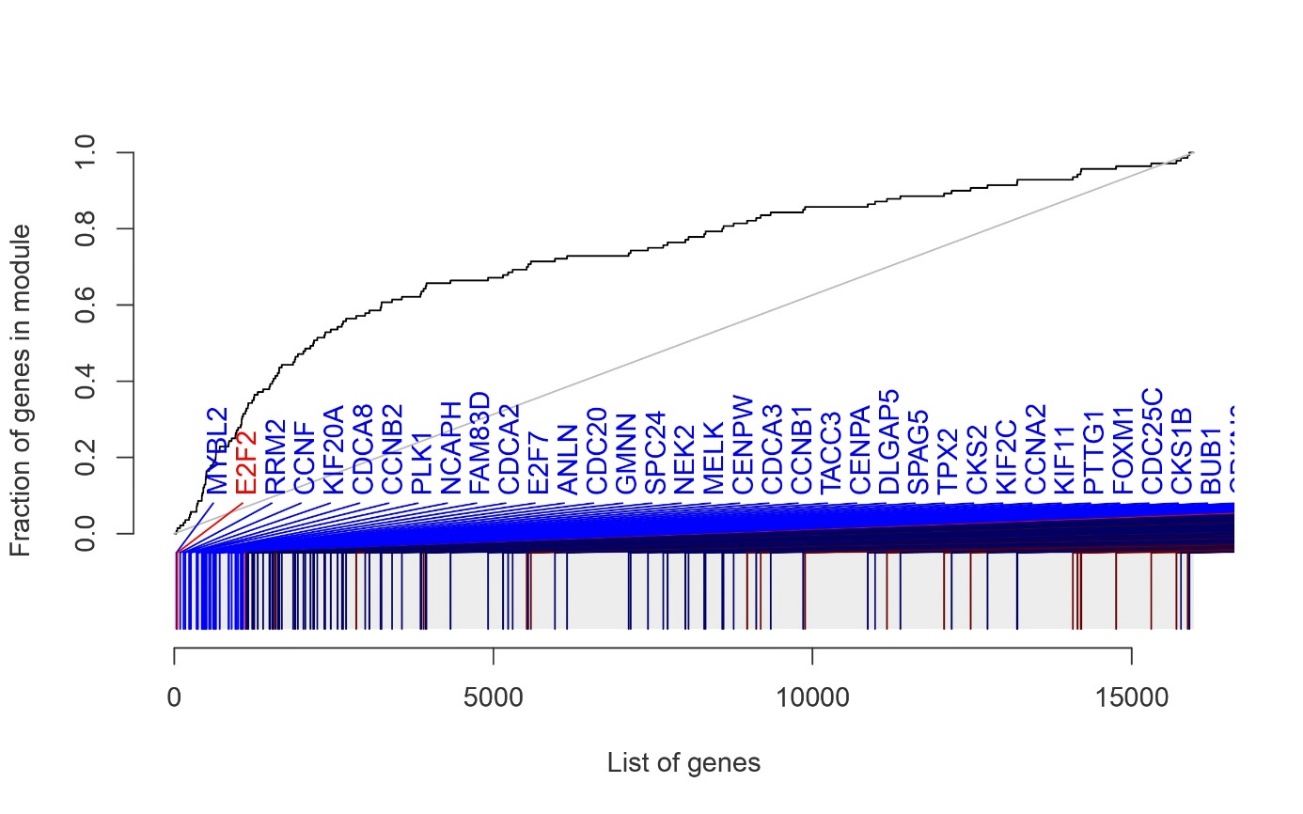


**Supplementary Figure 8A**. Gene set cell cycle (I) (LI.M4.1) was top 1 enriched in the Tmod collection after miR-30e-5p transfection (AUC = 0.73, P_adj_= 8.2 x 10^-18^, 140 genes).


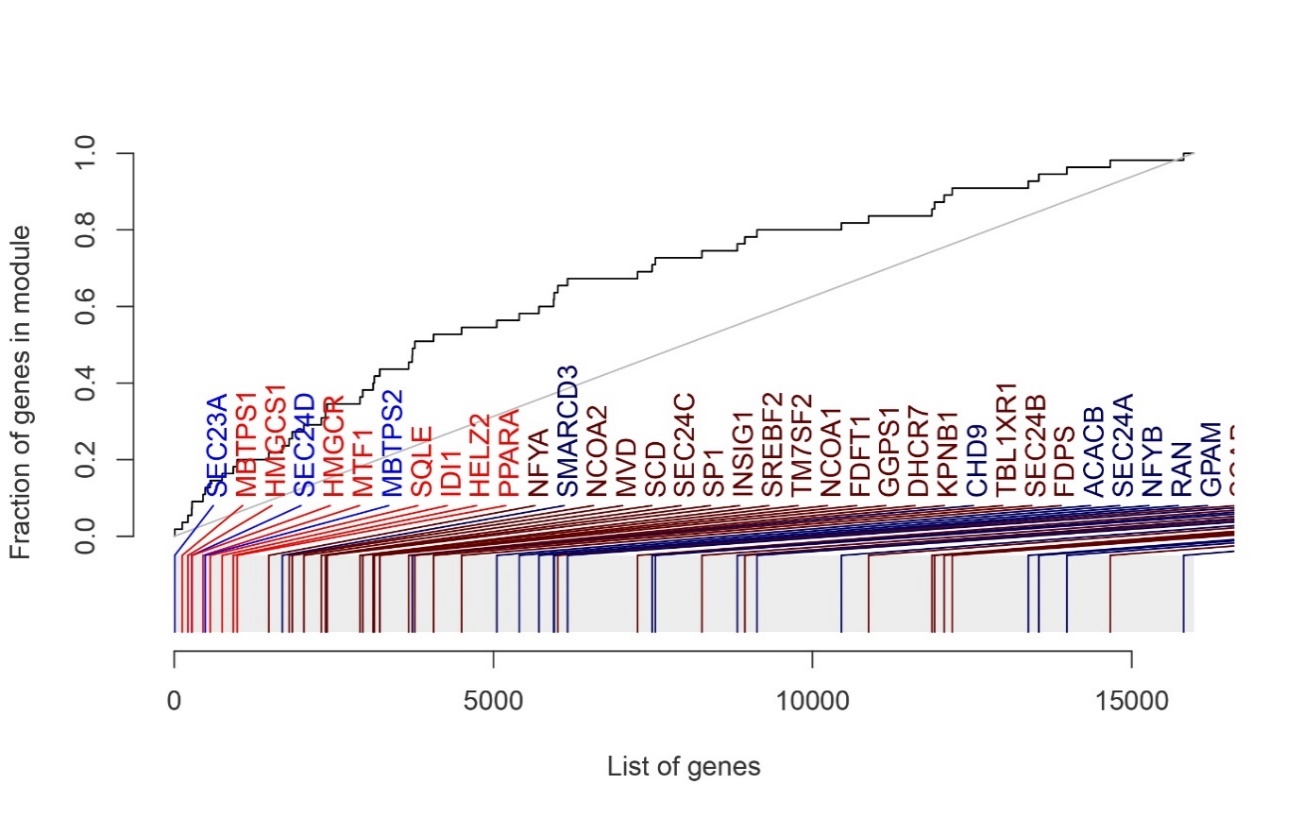


**Supplementary Figure 8 B**. Gene set Regulation of cholesterol biosynthesis by srebp srebf (M27001) was top 1 enriched in the Reactome collection after miR-30e-5p transfection (AUC = 0.66, P_adj_= 2.7 x 10^-3^, 55 genes).


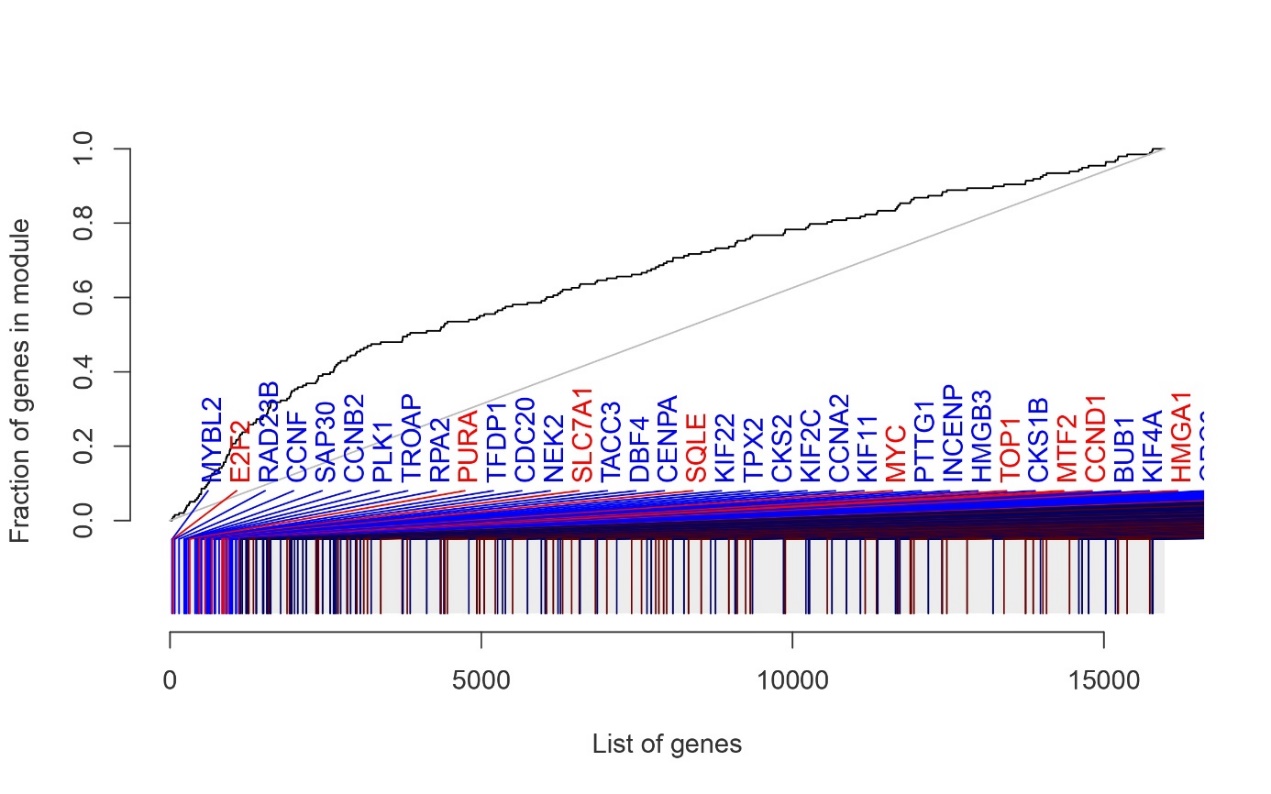


**Supplementary Figure 8 C**. Gene set G2M checkpoint (M5901) was top 1 enriched in the Hallmark collection after miR-17-5p transfection (AUC = 0.66, P_adj_= 2.8 x 10^-12^, 198 genes).


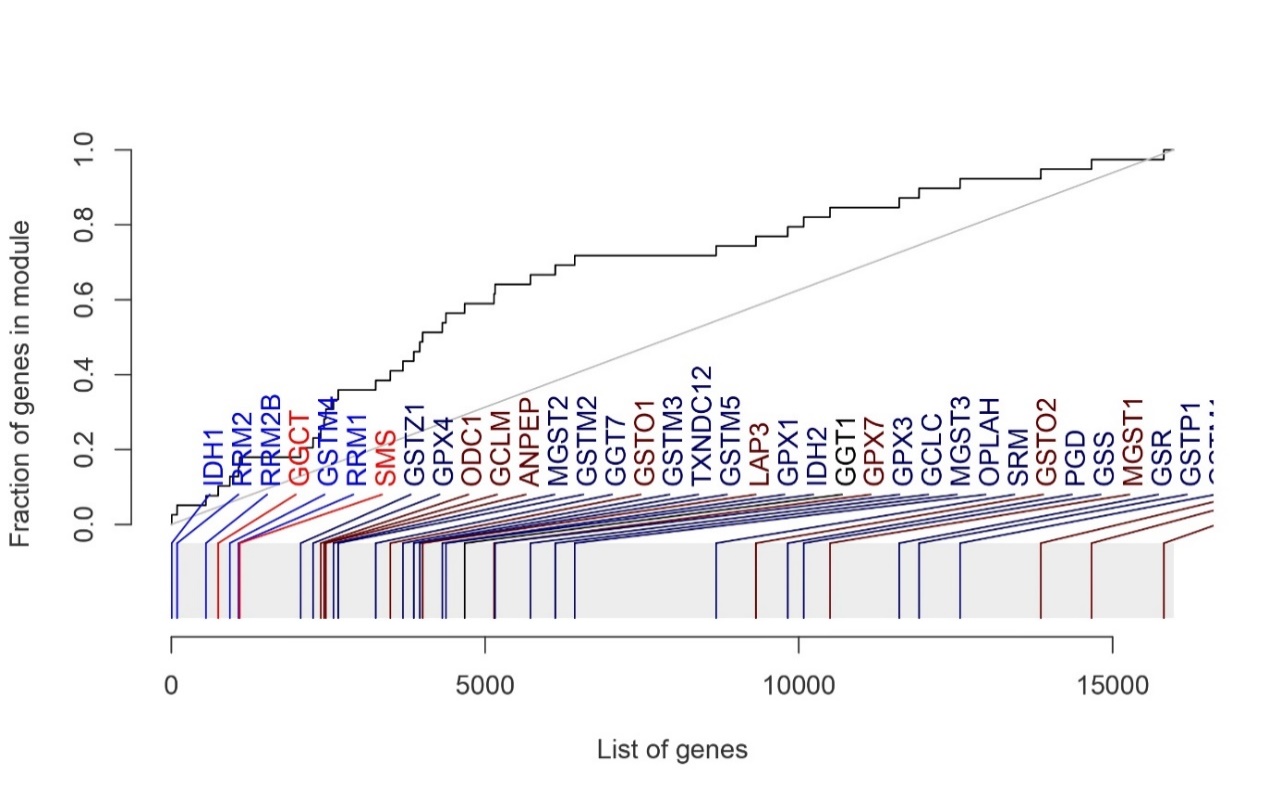


**Supplementary Figure 8 D**. Gene set Glutathione metabolism (M1840) was top 1 enriched in the KEGG collection after miR-30e-5p transfection (AUC = 0.66, P_adj_= 1.3 x 10^-2^ ,39 genes).


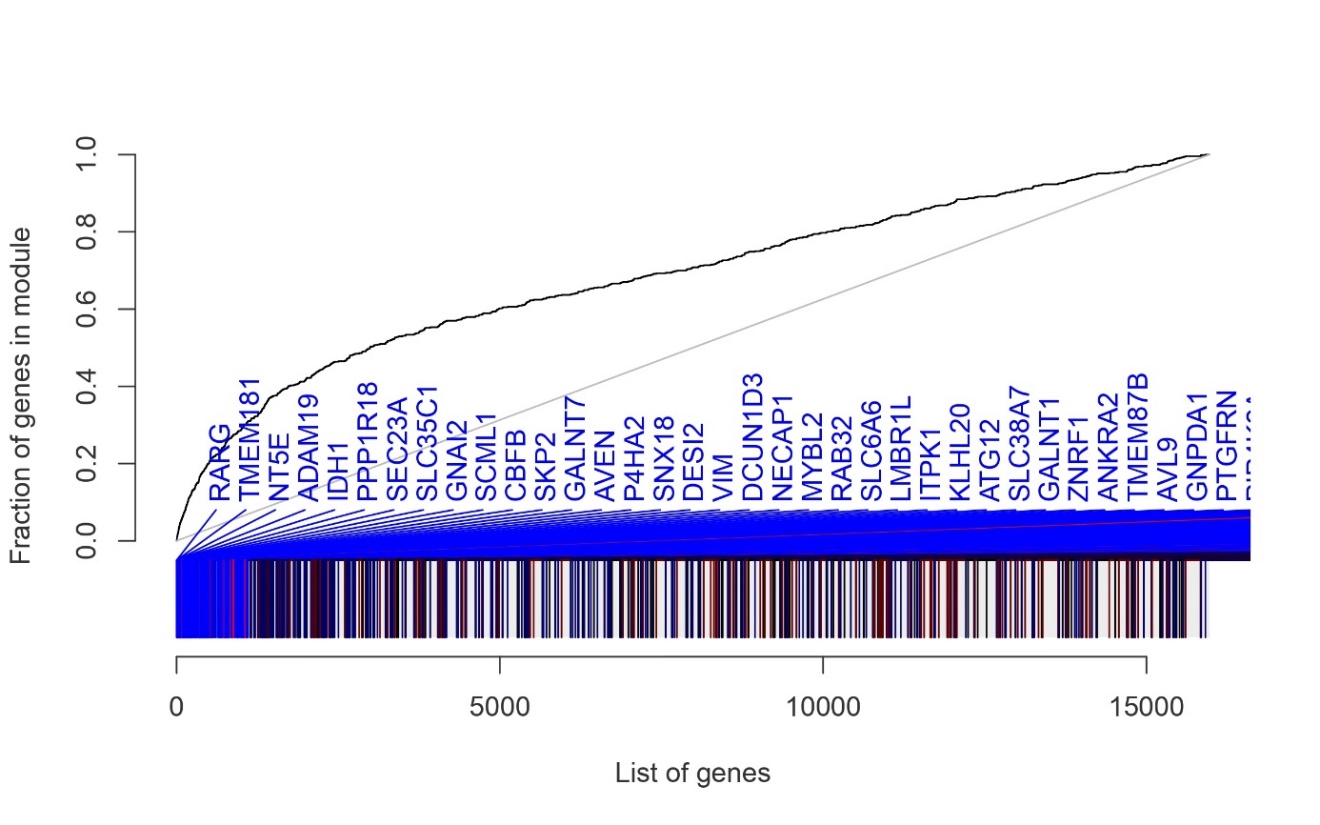


**Supplementary Figure 8 E**. Gene set miR-30e-5p (M30442) was top 1 enriched in the MIR collection after miR-30e-5p transfection (AUC = 0.70, P_adj_= 1.6 x 10^-103^ , 716 genes).


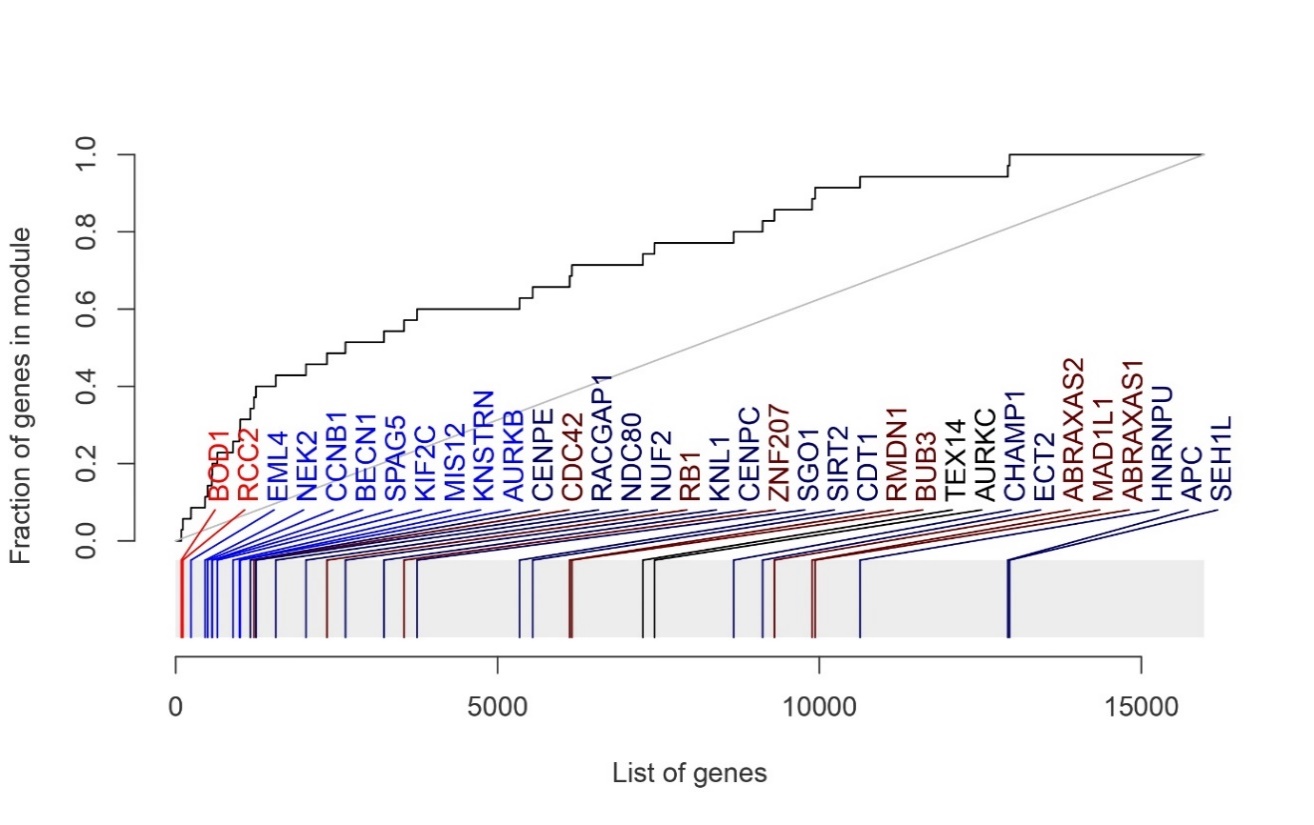


**Supplementary Figure 8 F**. Gene set Attachment of spindle microtubules to kinetochoren (M10211) was top 1 enriched in the Go collection after miR-30e-5p transfection (AUC = 0.73, P_adj_= 2.7 x 10^-4^ , 35 genes).

| Supplementary Table 2. DEGs of miR-130a-3P with target score | | | | | | | | | |
| --- | --- | --- | --- | --- | --- | --- | --- | --- | --- |
| **gene ID** | **symbol** | **entrez** | **baseMean** | **log2Fold Change** | **lfcSE** | **stat** | **pvalue** | **padj** | **Target Score** |
| ENSG00000112796 | ENPP5 | 59084 | 55359.00 | -2,537 | 0.370 | -6,861 | 0.0000 | 0.0000 | 96 |
| ENSG00000214575 | CPEB1 | 64506 | 303043.00 | -1,488 | 0.156 | -9,524 | 0.0000 | 0.0000 | 100 |
| ENSG00000164603 | BMT2 | 154743 | 321627.00 | -1,479 | 0.132 | -11,175 | 0.0000 | 0.0000 | 92 |
| ENSG00000204116 | CHIC1 | 53344 | 493212.00 | -1,232 | 0.135 | -9,115 | 0.0000 | 0.0000 | 77 |
| ENSG00000106348 | IMPDH1 | 3614 | 1438004.00 | -1,178 | 0.097 | -12,086 | 0.0000 | 0.0000 | 94 |
| ENSG00000185697 | MYBL1 | 4603 | 726946.00 | -1,177 | 0.109 | -10,847 | 0.0000 | 0.0000 | 99 |
| ENSG00000137996 | RTCA | 8634 | 1272542.00 | -1,165 | 0.086 | -13,566 | 0.0000 | 0.0000 | 95 |
| ENSG00000103381 | CPPED1 | 55313 | 1020312.00 | -1,155 | 0.097 | -11,890 | 0.0000 | 0.0000 | 73 |
| ENSG00000104643 | MTMR9 | 66036 | 544051.00 | -1,150 | 0.123 | -9,321 | 0.0000 | 0.0000 | 75 |
| ENSG00000154359 | LONRF1 | 91694 | 272344.00 | -1,111 | 0.149 | -7,465 | 0.0000 | 0.0000 | 76 |
| ENSG00000172009 | THOP1 | 7064 | 947293.00 | -1,093 | 0.106 | -10,340 | 0.0000 | 0.0000 | 86 |
| ENSG00000148411 | NACC2 | 138151 | 1023188.00 | -1,087 | 0.099 | -11,036 | 0.0000 | 0.0000 | 92 |
| ENSG00000100697 | DICER1 | 23405 | 1.508.723 | -1,063 | 0.082 | -12,945 | 0.0000 | 0.0000 | 91 |
| ENSG00000141441 | GAREM1 | 64762 | 110632.00 | -1,060 | 0.216 | -4,913 | 0.0000 | 0.0000 | 93 |
| ENSG00000100211 | CBY1 | 25776 | 116397.00 | -1,042 | 0.198 | -5,269 | 0.0000 | 0.0000 | 92 |
| ENSG00000170540 | ARL6IP1 | 23204 | 3.530.996 | -1,034 | 0.094 | -11,015 | 0.0000 | 0.0000 | 86 |
| ENSG00000147642 | SYBU | 55638 | 147059.00 | -1,026 | 0.193 | -5,327 | 0.0000 | 0.0000 | 97 |
| ENSG00000238227 | TMEM250 | 90120 | 908228.00 | -1,021 | 0.100 | -10,211 | 0.0000 | 0.0000 | 90 |
| ENSG00000151148 | UBE3B | 89910 | 1.133.871 | -1,020 | 0.082 | -12,372 | 0.0000 | 0.0000 | 90 |
| ENSG00000198732 | SMOC1 | 64093 | 69840.00 | -1,010 | 0.265 | -3,812 | 0.0001 | 0.0021 | 93 |
| ENSG00000180488 | MIGA1 | 374986 | 641228.00 | -1,008 | 0.102 | -9,887 | 0.0000 | 0.0000 | 81 |
| ENSG00000122126 | OCRL | 4952 | 1.363.648 | -1,004 | 0.098 | -10,273 | 0.0000 | 0.0000 | 67 |
| ENSG00000164930 | FZD6 | 8323 | 1.441.324 | -0.997 | 0.092 | -10,792 | 0.0000 | 0.0000 | 95 |
| ENSG00000174282 | ZBTB4 | 57659 | 2.848.384 | -0.987 | 0.094 | -10,445 | 0.0000 | 0.0000 | 97 |
| ENSG00000165704 | HPRT1 | 3251 | 800638.00 | -0.981 | 0.121 | -8,107 | 0.0000 | 0.0000 | 96 |
| ENSG00000029639 | TFB1M | 51106 | 222261.00 | -0.980 | 0.151 | -6,491 | 0.0000 | 0.0000 | 66 |
| ENSG00000148848 | ADAM12 | 8038 | 3.595.315 | -0.952 | 0.079 | -12,113 | 0.0000 | 0.0000 | 82 |
| ENSG00000185621 | LMLN | 89782 | 294238.00 | -0.921 | 0.137 | -6,730 | 0.0000 | 0.0000 | 92 |
| ENSG00000163513 | TGFBR2 | 7048 | 4.026.621 | -0.911 | 0.077 | -11,804 | 0.0000 | 0.0000 | 89 |
| ENSG00000164896 | FASTK | 10922 | 1.022.683 | -0.905 | 0.083 | -10,945 | 0.0000 | 0.0000 | 94 |
| ENSG00000085433 | WDR47 | 22911 | 717903.00 | -0.889 | 0.114 | -7,786 | 0.0000 | 0.0000 | 94 |
| ENSG00000135823 | STX6 | 10228 | 683770.00 | -0.879 | 0.115 | -7,635 | 0.0000 | 0.0000 | 93 |
| ENSG00000087111 | PIGS | 94005 | 1.283.943 | -0.877 | 0.086 | -10,194 | 0.0000 | 0.0000 | 78 |
| ENSG00000067955 | CBFB | 865 | 1327923.00 | -0.870 | 0.088 | -9,864 | 0.0000 | 0.0000 | 90 |
| ENSG00000001561 | ENPP4 | 22875 | 252516.00 | -0.867 | 0.142 | -6,089 | 0.0000 | 0.0000 | 70 |
| ENSG00000275023 | MLLT6 | 4302 | 819046.00 | -0.851 | 0.090 | -9,427 | 0.0000 | 0.0000 | 96 |
| ENSG00000206418 | RAB12 | 201475 | 531215.00 | -0.842 | 0.110 | -7,668 | 0.0000 | 0.0000 | 88 |
| ENSG00000025039 | RRAGD | 58528 | 246364.00 | -0.835 | 0.193 | -4,323 | 0.0000 | 0.0003 | 90 |
| ENSG00000154222 | CC2D1B | 200014 | 919994.00 | -0.829 | 0.087 | -9,547 | 0.0000 | 0.0000 | 68 |
| ENSG00000118242 | MREG | 55686 | 98722.00 | -0.817 | 0.230 | -3,550 | 0.0004 | 0.0051 | 69 |
| ENSG00000125520 | SLC2A4RG | 56731 | 1170635.00 | -0.811 | 0.102 | -7,929 | 0.0000 | 0.0000 | 87 |
| ENSG00000130779 | CLIP1 | 6249 | 3223200.00 | -0.798 | 0.074 | -10,791 | 0.0000 | 0.0000 | 100 |
| ENSG00000108604 | SMARCD2 | 6603 | 1176445.00 | -0.797 | 0.095 | -8,347 | 0.0000 | 0.0000 | 84 |
| ENSG00000172667 | ZMAT3 | 64393 | 2065670.00 | -0.795 | 0.088 | -9,065 | 0.0000 | 0.0000 | 97 |
| ENSG00000063176 | SPHK2 | 56848 | 238104.00 | -0.792 | 0.140 | -5,664 | 0.0000 | 0.0000 | 83 |
| ENSG00000164023 | SGMS2 | 166929 | 942149.00 | -0.784 | 0.094 | -8,388 | 0.0000 | 0.0000 | 60 |
| ENSG00000188906 | LRRK2 | 120892 | 308093.00 | -0.776 | 0.131 | -5,917 | 0.0000 | 0.0000 | 76 |
| ENSG00000133812 | SBF2 | 81846 | 812754.00 | -0.771 | 0.103 | -7,462 | 0.0000 | 0.0000 | 96 |
| ENSG00000139645 | ANKRD52 | 283373 | 4532895.00 | -0.758 | 0.086 | -8,796 | 0.0000 | 0.0000 | 66 |
| ENSG00000157077 | ZFYVE9 | 9372 | 764854.00 | -0.750 | 0.098 | -7,639 | 0.0000 | 0.0000 | 98 |
| ENSG00000100784 | RPS6KA5 | 9252 | 73103.00 | -0.739 | 0.247 | -2,998 | 0.0027 | 0.0238 | 99 |
| ENSG00000130956 | HABP4 | 22927 | 868186.00 | -0.710 | 0.100 | -7,067 | 0.0000 | 0.0000 | 56 |
| ENSG00000155111 | CDK19 | 23097 | 353806.00 | -0.709 | 0.139 | -5,113 | 0.0000 | 0.0000 | 97 |
| ENSG00000131389 | SLC6A6 | 6533 | 2533656.00 | -0.701 | 0.067 | -10,458 | 0.0000 | 0.0000 | 96 |
| ENSG00000142556 | ZNF614 | 80110 | 263975.00 | -0.700 | 0.140 | -4,983 | 0.0000 | 0.0000 | 74 |
| ENSG00000128973 | CLN6 | 54982 | 312837.00 | -0.700 | 0.132 | -5,313 | 0.0000 | 0.0000 | 69 |
| ENSG00000124225 | PMEPA1 | 56937 | 67203.00 | -0.698 | 0.258 | -2,705 | 0.0068 | 0.0472 | 88 |
| ENSG00000125149 | C16orf70 | 80262 | 339890.00 | -0.697 | 0.126 | -5,556 | 0.0000 | 0.0000 | 88 |
| ENSG00000144566 | RAB5A | 5868 | 1526181.00 | -0.690 | 0.092 | -7,533 | 0.0000 | 0.0000 | 94 |
| ENSG00000123095 | BHLHE41 | 79365 | 134637.00 | -0.684 | 0.187 | -3,657 | 0.0003 | 0.0036 | 83 |
| ENSG00000109686 | SH3D19 | 152503 | 2827655.00 | -0.683 | 0.083 | -8,253 | 0.0000 | 0.0000 | 95 |
| ENSG00000143933 | CALM2 | 805 | 10921119.00 | -0.678 | 0.083 | -8,141 | 0.0000 | 0.0000 | 87 |
| ENSG00000143756 | FBXO28 | 23219 | 1.020.685 | -0.658 | 0.094 | -6,989 | 0.0000 | 0.0000 | 94 |
| ENSG00000134049 | IER3IP1 | 51124 | 641202.00 | -0.658 | 0.103 | -6,375 | 0.0000 | 0.0000 | 77 |
| ENSG00000186310 | NAP1L3 | 4675 | 118365.00 | -0.654 | 0.203 | -3,218 | 0.0013 | 0.0134 | 69 |
| ENSG00000183741 | CBX6 | 23466 | 2.960.703 | -0.642 | 0.086 | -7,446 | 0.0000 | 0.0000 | 83 |
| ENSG00000155099 | PIP4P2 | 55529 | 424005.00 | -0.640 | 0.126 | -5,065 | 0.0000 | 0.0000 | 88 |
| ENSG00000148343 | MIGA2 | 84895 | 491737.00 | -0.638 | 0.113 | -5,648 | 0.0000 | 0.0000 | 91 |
| ENSG00000179981 | TSHZ1 | 10194 | 386560.00 | -0.637 | 0.119 | -5,346 | 0.0000 | 0.0000 | 97 |
| ENSG00000113300 | CNOT6 | 57472 | 831485.00 | -0.614 | 0.086 | -7,120 | 0.0000 | 0.0000 | 98 |
| ENSG00000137075 | RNF38 | 152006 | 585752.00 | -0.611 | 0.100 | -6,096 | 0.0000 | 0.0000 | 97 |
| ENSG00000181072 | CHRM2 | 1129 | 699640.00 | -0.609 | 0.125 | -4,855 | 0.0000 | 0.0000 | 77 |
| ENSG00000163820 | FYCO1 | 79443 | 2681196.00 | -0.609 | 0.091 | -6,715 | 0.0000 | 0.0000 | 70 |
| ENSG00000169446 | MMGT1 | 93380 | 783116.00 | -0.608 | 0.098 | -6,229 | 0.0000 | 0.0000 | 90 |
| ENSG00000155975 | VPS37A | 137492 | 1340973.00 | -0.607 | 0.082 | -7,398 | 0.0000 | 0.0000 | 96 |
| ENSG00000107897 | ACBD5 | 91452 | 1152409.00 | -0.607 | 0.083 | -7,339 | 0.0000 | 0.0000 | 93 |
| ENSG00000106772 | PRUNE2 | 158471 | 521505.00 | -0.603 | 0.124 | -4,869 | 0.0000 | 0.0000 | 92 |
| ENSG00000165029 | ABCA1 | 19 | 601825.00 | -0.598 | 0.120 | -4,982 | 0.0000 | 0.0000 | 67 |
| ENSG00000165699 | TSC1 | 7248 | 1222598.00 | -0.591 | 0.091 | -6,461 | 0.0000 | 0.0000 | 99 |
| ENSG00000138593 | SECISBP2L | 9728 | 1845915.00 | -0.591 | 0.074 | -7,952 | 0.0000 | 0.0000 | 98 |
| ENSG00000184371 | CSF1 | 1435 | 5020887.00 | -0.590 | 0.082 | -7,156 | 0.0000 | 0.0000 | 60 |
| ENSG00000122376 | SHLD2 | 54537 | 1087417.00 | -0.589 | 0.082 | -7,193 | 0.0000 | 0.0000 | 55 |
| ENSG00000007944 | MYLIP | 29116 | 158646.00 | -0.588 | 0.204 | -2,882 | 0.0039 | 0.0315 | 61 |
| ENSG00000070214 | SLC44A1 | 23446 | 1936773.00 | -0.587 | 0.076 | -7,756 | 0.0000 | 0.0000 | 93 |
| ENSG00000120509 | PDZD11 | 51248 | 1006626.00 | -0.584 | 0.091 | -6,380 | 0.0000 | 0.0000 | 82 |
| ENSG00000147650 | LRP12 | 29967 | 942443.00 | -0.580 | 0.099 | -5,832 | 0.0000 | 0.0000 | 84 |
| ENSG00000149218 | ENDOD1 | 23052 | 2078290.00 | -0.575 | 0.095 | -6,056 | 0.0000 | 0.0000 | 72 |
| ENSG00000068366 | ACSL4 | 2182 | 4326455.00 | -0.571 | 0.071 | -8,093 | 0.0000 | 0.0000 | 99 |
| ENSG00000139505 | MTMR6 | 9107 | 1676604.00 | -0.570 | 0.079 | -7,206 | 0.0000 | 0.0000 | 54 |
| ENSG00000185015 | CA13 | 377677 | 93018.00 | -0.568 | 0.214 | -2,659 | 0.0078 | 0.0521 | 64 |
| ENSG00000198718 | TOGARAM1 | 23116 | 436988.00 | -0.567 | 0.116 | -4,885 | 0.0000 | 0.0000 | 93 |
| ENSG00000110888 | CAPRIN2 | 65981 | 695631.00 | -0.559 | 0.105 | -5,343 | 0.0000 | 0.0000 | 96 |
| ENSG00000051825 | MPHOSPH9 | 10198 | 619904.00 | -0.558 | 0.100 | -5,598 | 0.0000 | 0.0000 | 96 |
| ENSG00000083290 | ULK2 | 9706 | 436969.00 | -0.554 | 0.107 | -5,163 | 0.0000 | 0.0000 | 95 |
| ENSG00000178177 | LCORL | 254251 | 291420.00 | -0.553 | 0.136 | -4,065 | 0.0000 | 0.0009 | 96 |
| ENSG00000154856 | APCDD1 | 147495 | 409696.00 | -0.553 | 0.167 | -3,301 | 0.0010 | 0.0107 | 94 |
| ENSG00000142687 | KIAA0319L | 79932 | 1.287.198 | -0.553 | 0.080 | -6,942 | 0.0000 | 0.0000 | 66 |
| ENSG00000024526 | DEPDC1 | 55635 | 1.439.988 | -0.551 | 0.105 | -5,271 | 0.0000 | 0.0000 | 78 |
| ENSG00000215440 | NPEPL1 | 79716 | 78724.00 | -0.548 | 0.229 | -2,392 | 0.0167 | 0.0916 | 83 |
| ENSG00000132716 | DCAF8 | 50717 | 591487.00 | -0.547 | 0.113 | -4,858 | 0.0000 | 0.0000 | 82 |
| ENSG00000132640 | BTBD3 | 22903 | 422908.00 | -0.546 | 0.129 | -4,227 | 0.0000 | 0.0005 | 89 |
| ENSG00000173559 | NABP1 | 64859 | 2.339.790 | -0.540 | 0.089 | -6,057 | 0.0000 | 0.0000 | 93 |
| ENSG00000151690 | MFSD6 | 54842 | 916742.00 | -0.534 | 0.121 | -4,396 | 0.0000 | 0.0002 | 97 |
| ENSG00000082269 | FAM135A | 57579 | 224995.00 | -0.534 | 0.139 | -3,832 | 0.0001 | 0.0020 | 64 |
| ENSG00000168502 | MTCL1 | 23255 | 1.169.828 | -0.529 | 0.099 | -5,358 | 0.0000 | 0.0000 | 84 |
| ENSG00000140022 | STON2 | 85439 | 229625.00 | -0.527 | 0.142 | -3,711 | 0.0002 | 0.0030 | 90 |
| ENSG00000133104 | SPART | 23111 | 3.163.983 | -0.526 | 0.072 | -7,351 | 0.0000 | 0.0000 | 90 |
| ENSG00000137177 | KIF13A | 63971 | 2.881.352 | -0.524 | 0.066 | -7,935 | 0.0000 | 0.0000 | 95 |
| ENSG00000175348 | TMEM9B | 56674 | 1.318.588 | -0.523 | 0.093 | -5,595 | 0.0000 | 0.0000 | 79 |
| ENSG00000105755 | ETHE1 | 23474 | 592256.00 | -0.522 | 0.106 | -4,900 | 0.0000 | 0.0000 | 65 |
| ENSG00000155016 | CYP2U1 | 113612 | 688364.00 | -0.520 | 0.102 | -5,089 | 0.0000 | 0.0000 | 89 |
| ENSG00000235162 | C12orf75 | 387882 | 6.138.966 | -0.520 | 0.078 | -6,638 | 0.0000 | 0.0000 | 70 |
| ENSG00000124788 | ATXN1 | 6310 | 821848.00 | -0.516 | 0.097 | -5,345 | 0.0000 | 0.0000 | 74 |
| ENSG00000146476 | ARMT1 | 79624 | 1.024.864 | -0.511 | 0.088 | -5,829 | 0.0000 | 0.0000 | 54 |
| ENSG00000169057 | MECP2 | 4204 | 1.213.429 | -0.506 | 0.084 | -6,035 | 0.0000 | 0.0000 | 98 |
| ENSG00000116138 | DNAJC16 | 23341 | 714169.00 | -0.505 | 0.094 | -5,387 | 0.0000 | 0.0000 | 97 |
| ENSG00000152520 | PAN3 | 255967 | 543212.00 | -0.505 | 0.107 | -4,733 | 0.0000 | 0.0001 | 96 |
| ENSG00000106366 | SERPINE1 | 5054 | 30.812.815 | -0.505 | 0.067 | -7,587 | 0.0000 | 0.0000 | 65 |
| ENSG00000179456 | ZBTB18 | 10472 | 222421.00 | -0.504 | 0.149 | -3,392 | 0.0007 | 0.0082 | 98 |
| ENSG00000025293 | PHF20 | 51230 | 1.463.067 | -0.498 | 0.076 | -6,558 | 0.0000 | 0.0000 | 97 |
| ENSG00000181751 | C5orf30 | 90355 | 517504.00 | -0.498 | 0.141 | -3,531 | 0.0004 | 0.0054 | 88 |
| ENSG00000178573 | MAF | 4094 | 150751.00 | -0.498 | 0.191 | -2,599 | 0.0093 | 0.0592 | 88 |
| ENSG00000166619 | BLCAP | 10904 | 1.424.993 | -0.494 | 0.089 | -5,546 | 0.0000 | 0.0000 | 94 |
| ENSG00000144724 | PTPRG | 5793 | 1.430.437 | -0.493 | 0.075 | -6,557 | 0.0000 | 0.0000 | 94 |
| ENSG00000020633 | RUNX3 | 864 | 242245.00 | -0.487 | 0.170 | -2,864 | 0.0042 | 0.0329 | 88 |
| ENSG00000131711 | MAP1B | 4131 | 13.510.126 | -0.472 | 0.070 | -6,778 | 0.0000 | 0.0000 | 61 |
| ENSG00000130164 | LDLR | 3949 | 2.105.925 | -0.468 | 0.099 | -4,704 | 0.0000 | 0.0001 | 96 |
| ENSG00000134444 | RELCH | 57614 | 1.204.071 | -0.466 | 0.079 | -5,896 | 0.0000 | 0.0000 | 52 |
| ENSG00000028839 | TBPL1 | 9519 | 502968.00 | -0.463 | 0.115 | -4,034 | 0.0001 | 0.0009 | 66 |
| ENSG00000133193 | FAM104A | 84923 | 555215.00 | -0.462 | 0.107 | -4,331 | 0.0000 | 0.0003 | 82 |
| ENSG00000175087 | PDIK1L | 149420 | 128453.00 | -0.461 | 0.194 | -2,375 | 0.0175 | 0.0947 | 89 |
| ENSG00000137216 | TMEM63B | 55362 | 1.073.482 | -0.460 | 0.089 | -5,148 | 0.0000 | 0.0000 | 67 |
| ENSG00000167930 | FAM234A | 83986 | 712681.00 | -0.460 | 0.098 | -4,696 | 0.0000 | 0.0001 | 64 |
| ENSG00000051382 | PIK3CB | 5291 | 803373.00 | -0.456 | 0.088 | -5,192 | 0.0000 | 0.0000 | 99 |
| ENSG00000115561 | CHMP3 | 51652 | 1.443.631 | -0.453 | 0.097 | -4,667 | 0.0000 | 0.0001 | 78 |
| ENSG00000183864 | TOB2 | 10766 | 1.505.971 | -0.452 | 0.079 | -5,693 | 0.0000 | 0.0000 | 70 |
| ENSG00000196950 | SLC39A10 | 57181 | 611476.00 | -0.452 | 0.112 | -4,037 | 0.0001 | 0.0009 | 58 |
| ENSG00000132824 | SERINC3 | 10955 | 5.186.486 | -0.451 | 0.065 | -6,941 | 0.0000 | 0.0000 | 97 |
| ENSG00000055070 | SZRD1 | 26099 | 2.678.800 | -0.448 | 0.073 | -6,137 | 0.0000 | 0.0000 | 84 |
| ENSG00000175592 | FOSL1 | 8061 | 5.438.584 | -0.444 | 0.088 | -5,015 | 0.0000 | 0.0000 | 84 |
| ENSG00000113504 | SLC12A7 | 10723 | 962957.00 | -0.441 | 0.096 | -4,575 | 0.0000 | 0.0001 | 65 |
| ENSG00000105063 | PPP6R1 | 22870 | 2.187.673 | -0.436 | 0.084 | -5,192 | 0.0000 | 0.0000 | 76 |
| ENSG00000134698 | AGO4 | 192670 | 425863.00 | -0.435 | 0.123 | -3,521 | 0.0004 | 0.0056 | 97 |
| ENSG00000100083 | GGA1 | 26088 | 762043.00 | -0.435 | 0.097 | -4,467 | 0.0000 | 0.0002 | 72 |
| ENSG00000092847 | AGO1 | 26523 | 855501.00 | -0.433 | 0.096 | -4,505 | 0.0000 | 0.0002 | 92 |
| ENSG00000185298 | CCDC137 | 339230 | 719049.00 | -0.432 | 0.109 | -3,981 | 0.0001 | 0.0011 | 55 |
| ENSG00000109572 | CLCN3 | 1182 | 2.059.111 | -0.431 | 0.075 | -5,758 | 0.0000 | 0.0000 | 89 |
| ENSG00000162105 | SHANK2 | 22941 | 231741.00 | -0.430 | 0.160 | -2,681 | 0.0073 | 0.0497 | 93 |
| ENSG00000150995 | ITPR1 | 3708 | 473593.00 | -0.425 | 0.127 | -3,342 | 0.0008 | 0.0094 | 93 |
| ENSG00000139625 | MAP3K12 | 7786 | 700384.00 | -0.425 | 0.094 | -4,540 | 0.0000 | 0.0001 | 93 |
| ENSG00000143458 | GABPB2 | 126626 | 314633.00 | -0.424 | 0.133 | -3,187 | 0.0014 | 0.0146 | 50 |
| ENSG00000028137 | TNFRSF1B | 7133 | 1.094.474 | -0.419 | 0.097 | -4,344 | 0.0000 | 0.0003 | 53 |
| ENSG00000164136 | IL15 | 3600 | 202280.00 | -0.418 | 0.147 | -2,839 | 0.0045 | 0.0349 | 92 |
| ENSG00000142188 | TMEM50B | 757 | 679048.00 | -0.418 | 0.100 | -4,175 | 0.0000 | 0.0006 | 82 |
| ENSG00000066739 | ATG2B | 55102 | 1.102.321 | -0.418 | 0.087 | -4,794 | 0.0000 | 0.0000 | 68 |
| ENSG00000135966 | TGFBRAP1 | 9392 | 1.133.780 | -0.417 | 0.087 | -4,775 | 0.0000 | 0.0000 | 62 |
| ENSG00000165322 | ARHGAP12 | 94134 | 1.543.054 | -0.412 | 0.081 | -5,074 | 0.0000 | 0.0000 | 94 |
| ENSG00000109113 | RAB34 | 83871 | 2.895.484 | -0.409 | 0.074 | -5,545 | 0.0000 | 0.0000 | 60 |
| ENSG00000118263 | KLF7 | 8609 | 744628.00 | -0.407 | 0.103 | -3,965 | 0.0001 | 0.0012 | 99 |
| ENSG00000169083 | AR | 367 | 251628.00 | -0.404 | 0.142 | -2,852 | 0.0043 | 0.0338 | 90 |
| ENSG00000115109 | EPB41L5 | 57669 | 541405.00 | -0.401 | 0.110 | -3,648 | 0.0003 | 0.0037 | 58 |
| ENSG00000173614 | NMNAT1 | 64802 | 379912.00 | -0.398 | 0.119 | -3,333 | 0.0009 | 0.0097 | 57 |
| ENSG00000165782 | PIP4P1 | 90809 | 617819.00 | -0.396 | 0.098 | -4,056 | 0.0000 | 0.0009 | 74 |
| ENSG00000110367 | DDX6 | 1656 | 3.219.689 | -0.394 | 0.072 | -5,511 | 0.0000 | 0.0000 | 98 |
| ENSG00000162409 | PRKAA2 | 5563 | 168647.00 | -0.394 | 0.161 | -2,448 | 0.0143 | 0.0819 | 67 |
| ENSG00000135272 | MDFIC | 29969 | 2.130.173 | -0.393 | 0.076 | -5,135 | 0.0000 | 0.0000 | 96 |
| ENSG00000119906 | SLF2 | 55719 | 1.300.265 | -0.391 | 0.085 | -4,576 | 0.0000 | 0.0001 | 70 |
| ENSG00000151208 | DLG5 | 9231 | 1.417.596 | -0.387 | 0.075 | -5,191 | 0.0000 | 0.0000 | 94 |
| ENSG00000166833 | NAV2 | 89797 | 1.799.138 | -0.387 | 0.084 | -4,586 | 0.0000 | 0.0001 | 57 |
| ENSG00000088356 | PDRG1 | 81572 | 441942.00 | -0.386 | 0.106 | -3,627 | 0.0003 | 0.0040 | 55 |
| ENSG00000171451 | DSEL | 92126 | 3.292.430 | -0.385 | 0.077 | -5,020 | 0.0000 | 0.0000 | 88 |
| ENSG00000172954 | LCLAT1 | 253558 | 350844.00 | -0.385 | 0.116 | -3,332 | 0.0009 | 0.0097 | 71 |
| ENSG00000101199 | ARFGAP1 | 55738 | 2.036.850 | -0.380 | 0.075 | -5,068 | 0.0000 | 0.0000 | 52 |
| ENSG00000141198 | TOM1L1 | 10040 | 344735.00 | -0.378 | 0.115 | -3,275 | 0.0011 | 0.0114 | 55 |
| ENSG00000038274 | MAT2B | 27430 | 1.357.173 | -0.376 | 0.089 | -4,212 | 0.0000 | 0.0005 | 79 |
| ENSG00000100731 | PCNX1 | 22990 | 2.014.396 | -0.375 | 0.072 | -5,199 | 0.0000 | 0.0000 | 85 |
| ENSG00000116747 | RO60 | 6738 | 882086.00 | -0.373 | 0.091 | -4,106 | 0.0000 | 0.0007 | 98 |
| ENSG00000174606 | ANGEL2 | 90806 | 631899.00 | -0.372 | 0.098 | -3,800 | 0.0001 | 0.0022 | 60 |
| ENSG00000110768 | GTF2H1 | 2965 | 2.029.178 | -0.371 | 0.073 | -5,084 | 0.0000 | 0.0000 | 85 |
| ENSG00000137449 | CPEB2 | 132864 | 1.514.363 | -0.371 | 0.088 | -4,235 | 0.0000 | 0.0004 | 77 |
| ENSG00000146833 | TRIM4 | 89122 | 649631.00 | -0.364 | 0.095 | -3,831 | 0.0001 | 0.0020 | 63 |
| ENSG00000114770 | ABCC5 | 10057 | 540047.00 | -0.357 | 0.122 | -2,921 | 0.0035 | 0.0287 | 91 |
| ENSG00000166526 | ZNF3 | 7551 | 487099.00 | -0.356 | 0.111 | -3,196 | 0.0014 | 0.0142 | 80 |
| ENSG00000132356 | PRKAA1 | 5562 | 2.099.876 | -0.355 | 0.080 | -4,449 | 0.0000 | 0.0002 | 85 |
| ENSG00000130508 | PXDN | 7837 | 8.902.474 | -0.354 | 0.082 | -4,291 | 0.0000 | 0.0004 | 79 |
| ENSG00000095574 | IKZF5 | 64376 | 262976.00 | -0.354 | 0.130 | -2,719 | 0.0066 | 0.0459 | 77 |
| ENSG00000101310 | SEC23B | 10483 | 1.400.138 | -0.351 | 0.090 | -3,880 | 0.0001 | 0.0017 | 69 |
| ENSG00000143553 | SNAPIN | 23557 | 594932.00 | -0.351 | 0.103 | -3,397 | 0.0007 | 0.0080 | 65 |
| ENSG00000215114 | UBXN2B | 137886 | 561567.00 | -0.350 | 0.101 | -3,468 | 0.0005 | 0.0065 | 86 |
| ENSG00000171320 | ESCO2 | 157570 | 404822.00 | -0.350 | 0.129 | -2,701 | 0.0069 | 0.0475 | 66 |
| ENSG00000110171 | TRIM3 | 10612 | 485979.00 | -0.350 | 0.100 | -3,484 | 0.0005 | 0.0062 | 58 |
| ENSG00000134717 | BTF3L4 | 91408 | 981342.00 | -0.348 | 0.086 | -4,042 | 0.0001 | 0.0009 | 88 |
| ENSG00000138641 | HERC3 | 8916 | 1.009.379 | -0.345 | 0.082 | -4,197 | 0.0000 | 0.0005 | 53 |
| ENSG00000023516 | AKAP11 | 11215 | 1.871.018 | -0.341 | 0.078 | -4,365 | 0.0000 | 0.0003 | 88 |
| ENSG00000106799 | TGFBR1 | 7046 | 896422.00 | -0.334 | 0.096 | -3,487 | 0.0005 | 0.0062 | 89 |
| ENSG00000011638 | TMEM159 | 57146 | 433837.00 | -0.333 | 0.114 | -2,935 | 0.0033 | 0.0278 | 95 |
| ENSG00000133639 | BTG1 | 694 | 3.060.625 | -0.330 | 0.068 | -4,832 | 0.0000 | 0.0000 | 88 |
| ENSG00000108395 | TRIM37 | 4591 | 1.099.867 | -0.330 | 0.082 | -4,039 | 0.0001 | 0.0009 | 69 |
| ENSG00000132294 | EFR3A | 23167 | 3.380.382 | -0.329 | 0.066 | -4,962 | 0.0000 | 0.0000 | 77 |
| ENSG00000170653 | ATF7 | 11016 | 365143.00 | -0.329 | 0.114 | -2,895 | 0.0038 | 0.0306 | 66 |
| ENSG00000068650 | ATP11A | 23250 | 736251.00 | -0.327 | 0.091 | -3,584 | 0.0003 | 0.0046 | 57 |
| ENSG00000152661 | GJA1 | 2697 | 16.082.928 | -0.326 | 0.070 | -4,677 | 0.0000 | 0.0001 | 100 |
| ENSG00000077684 | JADE1 | 79960 | 623789.00 | -0.325 | 0.101 | -3,214 | 0.0013 | 0.0136 | 91 |
| ENSG00000185112 | FAM43A | 131583 | 587019.00 | -0.324 | 0.105 | -3,075 | 0.0021 | 0.0197 | 69 |
| ENSG00000135720 | DYNC1LI2 | 1783 | 3.069.176 | -0.322 | 0.075 | -4,309 | 0.0000 | 0.0003 | 98 |
| ENSG00000111727 | HCFC2 | 29915 | 708363.00 | -0.322 | 0.094 | -3,443 | 0.0006 | 0.0070 | 96 |
| ENSG00000165175 | MID1IP1 | 58526 | 612806.00 | -0.316 | 0.113 | -2,809 | 0.0050 | 0.0373 | 91 |
| ENSG00000152700 | SAR1B | 51128 | 1.587.010 | -0.315 | 0.078 | -4,025 | 0.0001 | 0.0010 | 95 |
| ENSG00000132170 | PPARG | 5468 | 938502.00 | -0.315 | 0.092 | -3,421 | 0.0006 | 0.0075 | 90 |
| ENSG00000126775 | ATG14 | 22863 | 527412.00 | -0.314 | 0.102 | -3,075 | 0.0021 | 0.0197 | 85 |
| ENSG00000100242 | SUN2 | 25777 | 1.700.069 | -0.314 | 0.080 | -3,928 | 0.0001 | 0.0014 | 76 |
| ENSG00000010818 | HIVEP2 | 3097 | 809012.00 | -0.312 | 0.102 | -3,053 | 0.0023 | 0.0207 | 83 |
| ENSG00000175220 | ARHGAP1 | 392 | 2.497.893 | -0.311 | 0.071 | -4,364 | 0.0000 | 0.0003 | 85 |
| ENSG00000181827 | RFX7 | 64864 | 679736.00 | -0.310 | 0.096 | -3,247 | 0.0012 | 0.0124 | 95 |
| ENSG00000095564 | BTAF1 | 9044 | 1.116.732 | -0.310 | 0.082 | -3,761 | 0.0002 | 0.0025 | 90 |
| ENSG00000115170 | ACVR1 | 90 | 1.619.510 | -0.308 | 0.087 | -3,518 | 0.0004 | 0.0056 | 99 |
| ENSG00000144228 | SPOPL | 339745 | 461311.00 | -0.308 | 0.115 | -2,678 | 0.0074 | 0.0500 | 75 |
| ENSG00000197746 | PSAP | 5660 | 27.760.232 | -0.304 | 0.074 | -4,105 | 0.0000 | 0.0007 | 94 |
| ENSG00000204634 | TBC1D8 | 11138 | 764494.00 | -0.304 | 0.094 | -3,217 | 0.0013 | 0.0134 | 71 |
| ENSG00000138592 | USP8 | 9101 | 1.402.134 | -0.303 | 0.076 | -3,993 | 0.0001 | 0.0011 | 88 |
| ENSG00000163378 | EOGT | 285203 | 867142.00 | -0.300 | 0.087 | -3,446 | 0.0006 | 0.0070 | 81 |
| ENSG00000164163 | ABCE1 | 6059 | 2.554.405 | -0.297 | 0.080 | -3,710 | 0.0002 | 0.0030 | 74 |
| ENSG00000138162 | TACC2 | 10579 | 593398.00 | -0.296 | 0.097 | -3,069 | 0.0021 | 0.0200 | 83 |
| ENSG00000103404 | USP31 | 57478 | 347529.00 | -0.296 | 0.118 | -2,518 | 0.0118 | 0.0705 | 57 |
| ENSG00000169926 | KLF13 | 51621 | 1.731.102 | -0.292 | 0.070 | -4,173 | 0.0000 | 0.0006 | 55 |
| ENSG00000126903 | SLC10A3 | 8273 | 793164.00 | -0.290 | 0.093 | -3,124 | 0.0018 | 0.0172 | 65 |
| ENSG00000205352 | PRR13 | 54458 | 825487.00 | -0.290 | 0.088 | -3,296 | 0.0010 | 0.0108 | 53 |
| ENSG00000092969 | TGFB2 | 7042 | 669270.00 | -0.288 | 0.100 | -2,877 | 0.0040 | 0.0320 | 67 |
| ENSG00000124496 | TRERF1 | 55809 | 675837.00 | -0.286 | 0.098 | -2,915 | 0.0036 | 0.0291 | 72 |
| ENSG00000145391 | SETD7 | 80854 | 3.680.668 | -0.285 | 0.066 | -4,302 | 0.0000 | 0.0003 | 70 |
| ENSG00000166912 | MTMR10 | 54893 | 492302.00 | -0.284 | 0.103 | -2,762 | 0.0057 | 0.0414 | 88 |
| ENSG00000264364 | DYNLL2 | 140735 | 2.692.959 | -0.283 | 0.074 | -3,811 | 0.0001 | 0.0021 | 84 |
| ENSG00000048991 | R3HDM1 | 23518 | 1.098.973 | -0.282 | 0.080 | -3,535 | 0.0004 | 0.0053 | 87 |
| ENSG00000123684 | LPGAT1 | 9926 | 1.604.179 | -0.282 | 0.075 | -3,740 | 0.0002 | 0.0027 | 81 |
| ENSG00000140320 | BAHD1 | 22893 | 456070.00 | -0.279 | 0.118 | -2,364 | 0.0181 | 0.0968 | 82 |
| ENSG00000196914 | ARHGEF12 | 23365 | 5.380.525 | -0.279 | 0.063 | -4,422 | 0.0000 | 0.0002 | 55 |
| ENSG00000176658 | MYO1D | 4642 | 1.542.111 | -0.279 | 0.090 | -3,105 | 0.0019 | 0.0182 | 53 |
| ENSG00000198160 | MIER1 | 57708 | 1.029.849 | -0.275 | 0.080 | -3,422 | 0.0006 | 0.0075 | 96 |
| ENSG00000100030 | MAPK1 | 5594 | 4.960.704 | -0.274 | 0.069 | -3,996 | 0.0001 | 0.0011 | 96 |
| ENSG00000140948 | ZCCHC14 | 23174 | 656205.00 | -0.272 | 0.096 | -2,825 | 0.0047 | 0.0360 | 81 |
| ENSG00000124882 | EREG | 2069 | 854219.00 | -0.269 | 0.114 | -2,368 | 0.0179 | 0.0960 | 97 |
| ENSG00000102910 | LONP2 | 83752 | 2.317.989 | -0.268 | 0.072 | -3,752 | 0.0002 | 0.0026 | 58 |
| ENSG00000112851 | ERBIN | 55914 | 3.117.553 | -0.267 | 0.066 | -4,055 | 0.0001 | 0.0009 | 95 |
| ENSG00000011275 | RNF216 | 54476 | 1.417.629 | -0.265 | 0.078 | -3,413 | 0.0006 | 0.0076 | 95 |
| ENSG00000100625 | SIX4 | 51804 | 515027.00 | -0.265 | 0.101 | -2,631 | 0.0085 | 0.0553 | 85 |
| ENSG00000048707 | VPS13D | 55187 | 1.346.200 | -0.264 | 0.075 | -3,514 | 0.0004 | 0.0057 | 86 |
| ENSG00000072210 | ALDH3A2 | 224 | 1.547.667 | -0.264 | 0.085 | -3,096 | 0.0020 | 0.0186 | 79 |
| ENSG00000152601 | MBNL1 | 4154 | 4.559.953 | -0.262 | 0.060 | -4,339 | 0.0000 | 0.0003 | 95 |
| ENSG00000183530 | PRR14L | 253143 | 875207.00 | -0.262 | 0.088 | -2,979 | 0.0029 | 0.0248 | 57 |
| ENSG00000125347 | IRF1 | 3659 | 1.280.013 | -0.258 | 0.085 | -3,051 | 0.0023 | 0.0208 | 94 |
| ENSG00000137502 | RAB30 | 27314 | 1.359.748 | -0.257 | 0.093 | -2,759 | 0.0058 | 0.0417 | 79 |
| ENSG00000175073 | VCPIP1 | 80124 | 841762.00 | -0.257 | 0.086 | -3,004 | 0.0027 | 0.0234 | 65 |
| ENSG00000144744 | UBA3 | 9039 | 1.203.824 | -0.256 | 0.077 | -3,326 | 0.0009 | 0.0099 | 88 |
| ENSG00000196233 | LCOR | 84458 | 739215.00 | -0.256 | 0.093 | -2,757 | 0.0058 | 0.0420 | 69 |
| ENSG00000113558 | SKP1 | 6500 | 3.716.497 | -0.254 | 0.065 | -3,917 | 0.0001 | 0.0014 | 73 |
| ENSG00000197646 | PDCD1LG2 | 80380 | 846940.00 | -0.254 | 0.087 | -2,926 | 0.0034 | 0.0284 | 71 |
| ENSG00000011114 | BTBD7 | 55727 | 916196.00 | -0.253 | 0.092 | -2,760 | 0.0058 | 0.0416 | 91 |
| ENSG00000157500 | APPL1 | 26060 | 1.111.420 | -0.250 | 0.088 | -2,839 | 0.0045 | 0.0349 | 85 |
| ENSG00000091527 | CDV3 | 55573 | 6.565.729 | -0.248 | 0.062 | -4,028 | 0.0001 | 0.0010 | 52 |
| ENSG00000136295 | TTYH3 | 80727 | 4.686.893 | -0.245 | 0.066 | -3,709 | 0.0002 | 0.0030 | 78 |
| ENSG00000173706 | HEG1 | 57493 | 9.792.829 | -0.243 | 0.071 | -3,448 | 0.0006 | 0.0069 | 86 |
| ENSG00000170242 | USP47 | 55031 | 2.145.290 | -0.239 | 0.069 | -3,460 | 0.0005 | 0.0067 | 57 |
| ENSG00000035862 | TIMP2 | 7077 | 20.620.836 | -0.239 | 0.072 | -3,318 | 0.0009 | 0.0101 | 57 |
| ENSG00000115825 | PRKD3 | 23683 | 1.564.070 | -0.237 | 0.075 | -3,146 | 0.0017 | 0.0163 | 88 |
| ENSG00000100485 | SOS2 | 6655 | 656573.00 | -0.231 | 0.094 | -2,461 | 0.0138 | 0.0797 | 98 |
| ENSG00000168297 | PXK | 54899 | 1.294.250 | -0.230 | 0.082 | -2,817 | 0.0048 | 0.0367 | 79 |
| ENSG00000179119 | SPTY2D1 | 144108 | 1.086.748 | -0.229 | 0.093 | -2,450 | 0.0143 | 0.0816 | 88 |
| ENSG00000134152 | KATNBL1 | 79768 | 656896.00 | -0.229 | 0.091 | -2,526 | 0.0115 | 0.0693 | 73 |
| ENSG00000137145 | DENND4C | 55667 | 1.087.010 | -0.228 | 0.084 | -2,720 | 0.0065 | 0.0458 | 88 |
| ENSG00000164329 | TENT2 | 167153 | 1.395.215 | -0.226 | 0.076 | -2,954 | 0.0031 | 0.0266 | 85 |
| ENSG00000140391 | TSPAN3 | 10099 | 3.005.237 | -0.223 | 0.072 | -3,096 | 0.0020 | 0.0186 | 57 |
| ENSG00000186660 | ZFP91 | 80829 | 2.731.910 | -0.222 | 0.074 | -3,001 | 0.0027 | 0.0236 | 78 |
| ENSG00000058056 | USP13 | 8975 | 1.026.777 | -0.220 | 0.085 | -2,569 | 0.0102 | 0.0631 | 91 |
| ENSG00000113732 | ATP6V0E1 | 8992 | 5.581.475 | -0.217 | 0.058 | -3,737 | 0.0002 | 0.0027 | 76 |
| ENSG00000161800 | RACGAP1 | 29127 | 2.953.014 | -0.213 | 0.082 | -2,583 | 0.0098 | 0.0614 | 94 |
| ENSG00000101109 | STK4 | 6789 | 1.776.274 | -0.211 | 0.075 | -2,817 | 0.0048 | 0.0367 | 67 |
| ENSG00000072803 | FBXW11 | 23291 | 1.677.202 | -0.210 | 0.075 | -2,819 | 0.0048 | 0.0366 | 60 |
| ENSG00000129562 | DAD1 | 1603 | 2.723.639 | -0.206 | 0.068 | -3,006 | 0.0026 | 0.0233 | 72 |
| ENSG00000171246 | NPTX1 | 4884 | 4.960.524 | -0.206 | 0.070 | -2,945 | 0.0032 | 0.0272 | 56 |
| ENSG00000171940 | ZNF217 | 7764 | 1.499.060 | -0.205 | 0.083 | -2,483 | 0.0130 | 0.0765 | 94 |
| ENSG00000136478 | TEX2 | 55852 | 1.337.722 | -0.204 | 0.078 | -2,633 | 0.0085 | 0.0552 | 82 |
| ENSG00000138029 | HADHB | 3032 | 3.725.913 | -0.204 | 0.065 | -3,140 | 0.0017 | 0.0166 | 57 |
| ENSG00000074603 | DPP8 | 54878 | 1.181.163 | -0.203 | 0.081 | -2,489 | 0.0128 | 0.0754 | 63 |
| ENSG00000065526 | SPEN | 23013 | 1.532.585 | -0.202 | 0.073 | -2,781 | 0.0054 | 0.0397 | 82 |
| ENSG00000180628 | PCGF5 | 84333 | 2.224.647 | -0.199 | 0.082 | -2,417 | 0.0156 | 0.0868 | 57 |
| ENSG00000102081 | FMR1 | 2332 | 1.325.383 | -0.198 | 0.080 | -2,471 | 0.0135 | 0.0781 | 98 |
| ENSG00000173482 | PTPRM | 5797 | 1.582.151 | -0.196 | 0.075 | -2,615 | 0.0089 | 0.0573 | 51 |
| ENSG00000077254 | USP33 | 23032 | 2.124.648 | -0.195 | 0.073 | -2,686 | 0.0072 | 0.0492 | 85 |
| ENSG00000176994 | SMCR8 | 140775 | 1.469.288 | -0.194 | 0.082 | -2,365 | 0.0180 | 0.0967 | 62 |
| ENSG00000124222 | STX16 | 8675 | 1.266.367 | -0.190 | 0.077 | -2,481 | 0.0131 | 0.0767 | 65 |
| ENSG00000148516 | ZEB1 | 6935 | 2.599.540 | -0.189 | 0.073 | -2,601 | 0.0093 | 0.0591 | 68 |
| ENSG00000173120 | KDM2A | 22992 | 2.743.548 | -0.183 | 0.068 | -2,704 | 0.0069 | 0.0473 | 93 |
| ENSG00000100239 | PPP6R2 | 9701 | 1.405.136 | -0.183 | 0.077 | -2,371 | 0.0177 | 0.0954 | 81 |
| ENSG00000177565 | TBL1XR1 | 79718 | 1.797.027 | -0.181 | 0.075 | -2,424 | 0.0154 | 0.0858 | 98 |
| ENSG00000147416 | ATP6V1B2 | 526 | 4.658.169 | -0.181 | 0.064 | -2,832 | 0.0046 | 0.0355 | 86 |
| ENSG00000167123 | CERCAM | 51148 | 3.507.933 | -0.181 | 0.075 | -2,426 | 0.0153 | 0.0855 | 61 |
| ENSG00000204217 | BMPR2 | 659 | 2.667.234 | -0.178 | 0.068 | -2,604 | 0.0092 | 0.0587 | 87 |
| ENSG00000134352 | IL6ST | 3572 | 16.596.535 | -0.178 | 0.064 | -2,775 | 0.0055 | 0.0402 | 50 |
| ENSG00000092148 | HECTD1 | 25831 | 4.457.597 | -0.176 | 0.060 | -2,934 | 0.0033 | 0.0279 | 55 |
| ENSG00000066777 | ARFGEF1 | 10565 | 2.188.719 | -0.175 | 0.070 | -2,505 | 0.0122 | 0.0727 | 72 |
| ENSG00000145715 | RASA1 | 5921 | 2.128.971 | -0.174 | 0.070 | -2,497 | 0.0125 | 0.0740 | 77 |
| ENSG00000001629 | ANKIB1 | 54467 | 2.899.589 | -0.173 | 0.065 | -2,667 | 0.0076 | 0.0512 | 94 |
| ENSG00000111540 | RAB5B | 5869 | 3.049.437 | -0.173 | 0.073 | -2,378 | 0.0174 | 0.0943 | 64 |
| ENSG00000017797 | RALBP1 | 10928 | 2.062.376 | -0.171 | 0.068 | -2,528 | 0.0115 | 0.0690 | 74 |
| ENSG00000083799 | CYLD | 1540 | 2.843.434 | -0.169 | 0.067 | -2,530 | 0.0114 | 0.0687 | 70 |
| ENSG00000152291 | TGOLN2 | 10618 | 10.123.192 | -0.164 | 0.058 | -2,839 | 0.0045 | 0.0349 | 52 |
| ENSG00000143387 | CTSK | 1513 | 28.425.157 | -0.161 | 0.067 | -2,396 | 0.0166 | 0.0910 | 70 |
| ENSG00000206560 | ANKRD28 | 23243 | 4.228.631 | -0.160 | 0.063 | -2,547 | 0.0109 | 0.0662 | 55 |
| ENSG00000023318 | ERP44 | 23071 | 2.590.985 | 0.192 | 0.071 | 2,703 | 0.0069 | 0.0473 | 86 |
| ENSG00000142864 | SERBP1 | 26135 | 8.926.302 | 0.202 | 0.061 | 3,337 | 0.0008 | 0.0096 | 80 |
| ENSG00000006451 | RALA | 5898 | 2.908.940 | 0.211 | 0.076 | 2,781 | 0.0054 | 0.0397 | 73 |
| ENSG00000067596 | DHX8 | 1659 | 1.572.959 | 0.219 | 0.072 | 3,048 | 0.0023 | 0.0210 | 72 |
| ENSG00000145860 | RNF145 | 153830 | 1.815.989 | 0.226 | 0.077 | 2,947 | 0.0032 | 0.0270 | 92 |
| ENSG00000057019 | DCBLD2 | 131566 | 9.201.540 | 0.226 | 0.070 | 3,223 | 0.0013 | 0.0133 | 91 |
| ENSG00000112531 | QKI | 9444 | 3.405.412 | 0.235 | 0.078 | 3,000 | 0.0027 | 0.0236 | 73 |
| ENSG00000149289 | ZC3H12C | 85463 | 1.184.689 | 0.236 | 0.089 | 2,654 | 0.0079 | 0.0526 | 68 |
| ENSG00000166483 | WEE1 | 7465 | 1.095.641 | 0.240 | 0.082 | 2,913 | 0.0036 | 0.0293 | 85 |
| ENSG00000134453 | RBM17 | 84991 | 1.475.200 | 0.243 | 0.072 | 3,376 | 0.0007 | 0.0085 | 67 |
| ENSG00000118200 | CAMSAP2 | 23271 | 2.373.079 | 0.249 | 0.078 | 3,207 | 0.0013 | 0.0138 | 86 |
| ENSG00000278053 | DDX52 | 11056 | 1.126.884 | 0.251 | 0.082 | 3,068 | 0.0022 | 0.0200 | 59 |
| ENSG00000180357 | ZNF609 | 23060 | 1.161.616 | 0.270 | 0.081 | 3,338 | 0.0008 | 0.0095 | 81 |
| ENSG00000113441 | LNPEP | 4012 | 2.001.261 | 0.278 | 0.076 | 3,641 | 0.0003 | 0.0038 | 62 |
| ENSG00000204178 | MACO1 | 55219 | 712344.00 | 0.283 | 0.108 | 2,614 | 0.0090 | 0.0575 | 51 |
| ENSG00000114698 | PLSCR4 | 57088 | 600931.00 | 0.291 | 0.104 | 2,804 | 0.0050 | 0.0377 | 56 |
| ENSG00000159140 | SON | 6651 | 6.277.913 | 0.301 | 0.059 | 5,097 | 0.0000 | 0.0000 | 67 |
| ENSG00000165891 | E2F7 | 144455 | 1.513.335 | 0.316 | 0.093 | 3,389 | 0.0007 | 0.0082 | 67 |
| ENSG00000143815 | LBR | 3930 | 1.417.443 | 0.325 | 0.090 | 3,619 | 0.0003 | 0.0041 | 59 |
| ENSG00000139697 | SBNO1 | 55206 | 1.400.827 | 0.329 | 0.083 | 3,985 | 0.0001 | 0.0011 | 82 |
| ENSG00000122966 | CIT | 11113 | 1.629.165 | 0.331 | 0.085 | 3,886 | 0.0001 | 0.0016 | 77 |
| ENSG00000187605 | TET3 | 200424 | 374926.00 | 0.340 | 0.114 | 2,990 | 0.0028 | 0.0242 | 80 |
| ENSG00000184349 | EFNA5 | 1946 | 224757.00 | 0.345 | 0.143 | 2,414 | 0.0158 | 0.0875 | 58 |
| ENSG00000170525 | PFKFB3 | 5209 | 1.544.248 | 0.355 | 0.113 | 3,153 | 0.0016 | 0.0160 | 61 |
| ENSG00000165156 | ZHX1 | 11244 | 2.195.326 | 0.359 | 0.074 | 4,835 | 0.0000 | 0.0000 | 76 |
| ENSG00000125772 | GPCPD1 | 56261 | 619759.00 | 0.399 | 0.119 | 3,356 | 0.0008 | 0.0090 | 61 |
| ENSG00000120539 | MASTL | 84930 | 1.396.688 | 0.403 | 0.102 | 3,942 | 0.0001 | 0.0013 | 77 |
| ENSG00000143702 | CEP170 | 9859 | 2.249.686 | 0.431 | 0.099 | 4,364 | 0.0000 | 0.0003 | 95 |
| ENSG00000161813 | LARP4 | 113251 | 2.093.740 | 0.436 | 0.070 | 6,270 | 0.0000 | 0.0000 | 58 |
| ENSG00000181588 | MEX3D | 399664 | 344747.00 | 0.446 | 0.127 | 3,524 | 0.0004 | 0.0055 | 51 |
| ENSG00000198791 | CNOT7 | 29883 | 2.075.327 | 0.453 | 0.076 | 5,958 | 0.0000 | 0.0000 | 86 |
| ENSG00000100320 | RBFOX2 | 23543 | 5.529.438 | 0.478 | 0.063 | 7,531 | 0.0000 | 0.0000 | 58 |
| ENSG00000152804 | HHEX | 3087 | 208086.00 | 0.481 | 0.156 | 3,075 | 0.0021 | 0.0197 | 57 |
| ENSG00000068489 | PRR11 | 55771 | 1265152.00 | 0.500 | 0.091 | 5,495 | 0.0000 | 0.0000 | 52 |
| ENSG00000186480 | INSIG1 | 3638 | 2796919.00 | 0.524 | 0.211 | 2,481 | 0.0131 | 0.0768 | 87 |
| ENSG00000007968 | E2F2 | 1870 | 113881.00 | 0.563 | 0.225 | 2,503 | 0.0123 | 0.0730 | 62 |
| ENSG00000138757 | G3BP2 | 9908 | 3888223.00 | 0.565 | 0.065 | 8,650 | 0.0000 | 0.0000 | 84 |
| ENSG00000148572 | NRBF2 | 29982 | 506938.00 | 0.579 | 0.102 | 5,658 | 0.0000 | 0.0000 | 81 |
| ENSG00000113448 | PDE4D | 5144 | 245759.00 | 0.612 | 0.150 | 4,069 | 0.0000 | 0.0008 | 59 |
| ENSG00000159167 | STC1 | 6781 | 1682776.00 | 0.653 | 0.113 | 5,789 | 0.0000 | 0.0000 | 54 |
| ENSG00000112576 | CCND3 | 896 | 1740369.00 | 0.665 | 0.242 | 2,746 | 0.0060 | 0.0430 | 58 |
| ENSG00000135378 | PRRG4 | 79056 | 89792.00 | 0.676 | 0.243 | 2,777 | 0.0055 | 0.0400 | 55 |
| ENSG00000196782 | MAML3 | 55534 | 127733.00 | 0.709 | 0.201 | 3,520 | 0.0004 | 0.0056 | 55 |
| ENSG00000165731 | RET | 5979 | 63111.00 | 0.757 | 0.278 | 2,721 | 0.0065 | 0.0457 | 68 |
| ENSG00000106070 | GRB10 | 2887 | 801290.00 | 0.758 | 0.098 | 7,756 | 0.0000 | 0.0000 | 52 |
| ENSG00000079215 | SLC1A3 | 6507 | 63678.00 | 1,054 | 0.311 | 3,391 | 0.0007 | 0.0082 | 66 |
| ENSG00000105976 | MET | 4233 | 2752155.00 | 1,131 | 0.089 | 12,711 | 0.0000 | 0.0000 | 93 |
| ENSG00000112305 | SMAP1 | 60682 | 154413.00 | 1,132 | 0.169 | 6,691 | 0.0000 | 0.0000 | 82 |

| Supplementary Table 3. DEGs of miR-142-3P with target score | | | | | | | | | |
| --- | --- | --- | --- | --- | --- | --- | --- | --- | --- |
| **gene ID** | **symbol** | **entrez** | **baseMean** | **log2Fold**  **Change** | **lfcSE** | **stat** | **pvalue** | **padj** | **Target Score** |
| ENSG00000106299 | WASL | 8976 | 921521.00 | -2,781 | 0.109 | -25,550 | 0.0000 | 0.0000 | 94 |
| ENSG00000101363 | MANBAL | 63905 | 643291.00 | -2,271 | 0.114 | -19,981 | 0.0000 | 0.0000 | 78 |
| ENSG00000138463 | SLC49A4 | 84925 | 266602.00 | -1,962 | 0.149 | -13,170 | 0.0000 | 0.0000 | 96 |
| ENSG00000165410 | CFL2 | 1073 | 2841990 | -1,962 | 0.105 | -18,601 | 0.0000 | 0.0000 | 88 |
| ENSG00000160113 | NR2F6 | 2063 | 418369.00 | -1,944 | 0.124 | -15,706 | 0.0000 | 0.0000 | 83 |
| ENSG00000170892 | TSEN34 | 79042 | 693912.00 | -1,901 | 0.102 | -18,617 | 0.0000 | 0.0000 | 94 |
| ENSG00000204673 | AKT1S1 | 84335 | 1576392.00 | -1,856 | 0.083 | -22,395 | 0.0000 | 0.0000 | 86 |
| ENSG00000137507 | LRRC32 | 2615 | 189024.00 | -1,642 | 0.195 | -8,421 | 0.0000 | 0.0000 | 88 |
| ENSG00000172819 | RARG | 5916 | 2263331.00 | -1,633 | 0.075 | -21,912 | 0.0000 | 0.0000 | 81 |
| ENSG00000068383 | INPP5A | 3632 | 475614.00 | -1,624 | 0.115 | -14,066 | 0.0000 | 0.0000 | 86 |
| ENSG00000197712 | FAM114A1 | 92689 | 3390216.00 | -1,608 | 0.078 | -20,530 | 0.0000 | 0.0000 | 54 |
| ENSG00000179010 | MRFAP1 | 93621 | 6481995.00 | -1,555 | 0.074 | -20,983 | 0.0000 | 0.0000 | 89 |
| ENSG00000049245 | VAMP3 | 9341 | 3448617.00 | -1,554 | 0.095 | -16,329 | 0.0000 | 0.0000 | 86 |
| ENSG00000115084 | SLC35F5 | 80255 | 2053875.00 | -1,544 | 0.077 | -20,035 | 0.0000 | 0.0000 | 92 |
| ENSG00000128591 | FLNC | 2318 | ########## | -1,479 | 0.086 | -17,130 | 0.0000 | 0.0000 | 61 |
| ENSG00000104866 | PPP1R37 | 284352 | 715276.00 | -1,456 | 0.096 | -15,131 | 0.0000 | 0.0000 | 95 |
| ENSG00000164292 | RHOBTB3 | 22836 | 3169285.00 | -1,445 | 0.085 | -17,023 | 0.0000 | 0.0000 | 99 |
| ENSG00000106799 | TGFBR1 | 7046 | 896422.00 | -1,432 | 0.100 | -14,356 | 0.0000 | 0.0000 | 89 |
| ENSG00000151239 | TWF1 | 5756 | 3187692.00 | -1,405 | 0.089 | -15,805 | 0.0000 | 0.0000 | 98 |
| ENSG00000157193 | LRP8 | 7804 | 634666.00 | -1,405 | 0.114 | -12,289 | 0.0000 | 0.0000 | 66 |
| ENSG00000168092 | PAFAH1B2 | 5049 | 1832992.00 | -1,397 | 0.078 | -17,939 | 0.0000 | 0.0000 | 81 |
| ENSG00000107560 | RAB11FIP2 | 22841 | 593303.00 | -1,388 | 0.131 | -10,561 | 0.0000 | 0.0000 | 89 |
| ENSG00000074416 | MGLL | 11343 | 5620511.00 | -1,387 | 0.180 | -7,708 | 0.0000 | 0.0000 | 65 |
| ENSG00000257365 | FNTB | 2342 | 140409.00 | -1,385 | 0.184 | -7,517 | 0.0000 | 0.0000 | 71 |
| ENSG00000128791 | TWSG1 | 57045 | 2812167.00 | -1,383 | 0.093 | -14,811 | 0.0000 | 0.0000 | 80 |
| ENSG00000134352 | IL6ST | 3572 | ########## | -1,380 | 0.065 | -21,369 | 0.0000 | 0.0000 | 86 |
| ENSG00000203950 | RTL8A | 26071 | 619888.00 | -1,336 | 0.110 | -12,145 | 0.0000 | 0.0000 | 60 |
| ENSG00000196950 | SLC39A10 | 57181 | 611476.00 | -1,335 | 0.116 | -11,520 | 0.0000 | 0.0000 | 62 |
| ENSG00000169504 | CLIC4 | 25932 | ########## | -1,331 | 0.068 | -19,549 | 0.0000 | 0.0000 | 80 |
| ENSG00000115540 | MOB4 | 25843 | 482895.00 | -1,317 | 0.130 | -10,170 | 0.0000 | 0.0000 | 92 |
| ENSG00000109184 | DCUN1D4 | 23142 | 926171.00 | -1,273 | 0.093 | -13,636 | 0.0000 | 0.0000 | 87 |
| ENSG00000158402 | CDC25C | 995 | 223714.00 | -1,270 | 0.165 | -7,689 | 0.0000 | 0.0000 | 88 |
| ENSG00000139324 | TMTC3 | 160418 | 1277963.00 | -1,234 | 0.096 | -12,857 | 0.0000 | 0.0000 | 54 |
| ENSG00000136935 | GOLGA1 | 2800 | 490310.00 | -1,233 | 0.115 | -10,763 | 0.0000 | 0.0000 | 72 |
| ENSG00000116539 | ASH1L | 55870 | 1181501.00 | -1,223 | 0.084 | -14,501 | 0.0000 | 0.0000 | 98 |
| ENSG00000157224 | CLDN12 | 9069 | 840992.00 | -1,223 | 0.098 | -12,421 | 0.0000 | 0.0000 | 95 |
| ENSG00000163694 | RBM47 | 54502 | 78438.00 | -1,217 | 0.265 | -4,591 | 0.0000 | 0.0001 | 92 |
| ENSG00000063245 | EPN1 | 29924 | 2,798.658 | -1,212 | 0.088 | -13,730 | 0.0000 | 0.0000 | 78 |
| ENSG00000143771 | CNIH4 | 29097 | 762166.00 | -1,198 | 0.091 | -13,113 | 0.0000 | 0.0000 | 75 |
| ENSG00000213694 | S1PR3 | 1903 | 2,431.007 | -1,197 | 0.072 | -16,580 | 0.0000 | 0.0000 | 92 |
| ENSG00000088854 | C20orf194 | 25943 | 768291.00 | -1,173 | 0.098 | -11,997 | 0.0000 | 0.0000 | 96 |
| ENSG00000096433 | ITPR3 | 3710 | 5,285.923 | -1,168 | 0.065 | -17,926 | 0.0000 | 0.0000 | 81 |
| ENSG00000085733 | CTTN | 2017 | 8,475.783 | -1,166 | 0.075 | -15,454 | 0.0000 | 0.0000 | 83 |
| ENSG00000105855 | ITGB8 | 3696 | 157116.00 | -1,162 | 0.205 | -5,672 | 0.0000 | 0.0000 | 96 |
| ENSG00000129493 | HEATR5A | 25938 | 611787.00 | -1,137 | 0.121 | -9,422 | 0.0000 | 0.0000 | 86 |
| ENSG00000104388 | RAB2A | 5862 | 2,961.051 | -1,136 | 0.073 | -15,658 | 0.0000 | 0.0000 | 94 |
| ENSG00000106615 | RHEB | 6009 | 2,092.798 | -1,134 | 0.092 | -12,356 | 0.0000 | 0.0000 | 87 |
| ENSG00000173327 | MAP3K11 | 4296 | 911761.00 | -1,112 | 0.095 | -11,751 | 0.0000 | 0.0000 | 78 |
| ENSG00000114354 | TFG | 10342 | 3,162.803 | -1,107 | 0.069 | -15,954 | 0.0000 | 0.0000 | 56 |
| ENSG00000094975 | SUCO | 51430 | 1,234.417 | -1,104 | 0.082 | -13,535 | 0.0000 | 0.0000 | 88 |
| ENSG00000157800 | SLC37A3 | 84255 | 977316.00 | -1,098 | 0.089 | -12,347 | 0.0000 | 0.0000 | 96 |
| ENSG00000069275 | NUCKS1 | 64710 | 8,279.550 | -1,098 | 0.068 | -16,149 | 0.0000 | 0.0000 | 86 |
| ENSG00000206418 | RAB12 | 201475 | 531215.00 | -1,079 | 0.111 | -9,718 | 0.0000 | 0.0000 | 87 |
| ENSG00000123562 | MORF4L2 | 9643 | 6,705.566 | -1,061 | 0.063 | -16,884 | 0.0000 | 0.0000 | 91 |
| ENSG00000137409 | MTCH1 | 23787 | 4,550.353 | -1,061 | 0.072 | -14,805 | 0.0000 | 0.0000 | 86 |
| ENSG00000151012 | SLC7A11 | 23657 | 4,327.745 | -1,057 | 0.083 | -12,730 | 0.0000 | 0.0000 | 86 |
| ENSG00000165244 | ZNF367 | 195828 | 610409.00 | -1,057 | 0.115 | -9,198 | 0.0000 | 0.0000 | 79 |
| ENSG00000111652 | COPS7A | 50813 | 1,919.570 | -1,052 | 0.091 | -11,549 | 0.0000 | 0.0000 | 64 |
| ENSG00000100804 | PSMB5 | 5693 | 3,174.963 | -1,046 | 0.088 | -11,851 | 0.0000 | 0.0000 | 94 |
| ENSG00000277443 | MARCKS | 4082 | 5,557.882 | -1,040 | 0.092 | -11,247 | 0.0000 | 0.0000 | 72 |
| ENSG00000177888 | ZBTB41 | 360023 | 546573.00 | -1,038 | 0.105 | -9,916 | 0.0000 | 0.0000 | 96 |
| ENSG00000138448 | ITGAV | 3685 | 4,885.972 | -1,037 | 0.201 | -5,152 | 0.0000 | 0.0000 | 95 |
| ENSG00000108021 | TASOR2 | 54906 | 2,073.533 | -1,031 | 0.071 | -14,589 | 0.0000 | 0.0000 | 100 |
| ENSG00000172354 | GNB2 | 2783 | 3,487.751 | -1,013 | 0.075 | -13,547 | 0.0000 | 0.0000 | 86 |
| ENSG00000180488 | MIGA1 | 374986 | 641228.00 | -0.999 | 0.102 | -9,815 | 0.0000 | 0.0000 | 61 |
| ENSG00000197713 | RPE | 6120 | 1,185.066 | -0.977 | 0.095 | -10,338 | 0.0000 | 0.0000 | 76 |
| ENSG00000156162 | DPY19L4 | 286148 | 674551.00 | -0.959 | 0.101 | -9,514 | 0.0000 | 0.0000 | 51 |
| ENSG00000014824 | SLC30A9 | 10463 | 1,932.663 | -0.958 | 0.089 | -10,771 | 0.0000 | 0.0000 | 74 |
| ENSG00000204388 | HSPA1B | 3304 | 1,412.753 | -0.952 | 0.096 | -9,950 | 0.0000 | 0.0000 | 74 |
| ENSG00000169446 | MMGT1 | 93380 | 783116.00 | -0.952 | 0.099 | -9,623 | 0.0000 | 0.0000 | 67 |
| ENSG00000115561 | CHMP3 | 51652 | 1,443.631 | -0.951 | 0.098 | -9,690 | 0.0000 | 0.0000 | 81 |
| ENSG00000143924 | EML4 | 27436 | 1,720.293 | -0.951 | 0.084 | -11,358 | 0.0000 | 0.0000 | 78 |
| ENSG00000181904 | C5orf24 | 134553 | 1,280.273 | -0.943 | 0.081 | -11,660 | 0.0000 | 0.0000 | 91 |
| ENSG00000105514 | RAB3D | 9545 | 159384.00 | -0.941 | 0.172 | -5,461 | 0.0000 | 0.0000 | 60 |
| ENSG00000167799 | NUDT8 | 254552 | 38184.00 | -0.931 | 0.336 | -2,772 | 0.0056 | 0.0356 | 60 |
| ENSG00000085978 | ATG16L1 | 55054 | 659895.00 | -0.92 | 0.100 | -9,184 | 0.0000 | 0.0000 | 67 |
| ENSG00000131023 | LATS1 | 9113 | 658725.00 | -0.915 | 0.097 | -9,476 | 0.0000 | 0.0000 | 66 |
| ENSG00000175727 | MLXIP | 22877 | 1,052.945 | -0.911 | 0.095 | -9,619 | 0.0000 | 0.0000 | 84 |
| ENSG00000162636 | FAM102B | 284611 | 498710.00 | -0.911 | 0.110 | -8,254 | 0.0000 | 0.0000 | 69 |
| ENSG00000127329 | PTPRB | 5787 | 117404.00 | -0.906 | 0.199 | -4,548 | 0.0000 | 0.0001 | 85 |
| ENSG00000213465 | ARL2 | 402 | 410947.00 | -0.897 | 0.121 | -7,406 | 0.0000 | 0.0000 | 57 |
| ENSG00000139354 | GAS2L3 | 283431 | 528454.00 | -0.893 | 0.117 | -7,629 | 0.0000 | 0.0000 | 92 |
| ENSG00000136238 | RAC1 | 5879 | 6,299.188 | -0.89 | 0.071 | -12,495 | 0.0000 | 0.0000 | 93 |
| ENSG00000189056 | RELN | 5649 | 993033.00 | -0.89 | 0.126 | -7,048 | 0.0000 | 0.0000 | 62 |
| ENSG00000185359 | HGS | 9146 | 1,769.373 | -0.878 | 0.076 | -11,551 | 0.0000 | 0.0000 | 91 |
| ENSG00000137273 | FOXF2 | 2295 | 1,367.175 | -0.878 | 0.082 | -10,721 | 0.0000 | 0.0000 | 52 |
| ENSG00000076201 | PTPN23 | 25930 | 1,377.074 | -0.876 | 0.076 | -11,471 | 0.0000 | 0.0000 | 94 |
| ENSG00000185619 | PCGF3 | 10336 | 781132.00 | -0.863 | 0.092 | -9,381 | 0.0000 | 0.0000 | 86 |
| ENSG00000185722 | ANKFY1 | 51479 | 2,655.003 | -0.86 | 0.073 | -11,829 | 0.0000 | 0.0000 | 59 |
| ENSG00000136738 | STAM | 8027 | 1,052.803 | -0.856 | 0.086 | -10,006 | 0.0000 | 0.0000 | 92 |
| ENSG00000140948 | ZCCHC14 | 23174 | 656205.00 | -0.853 | 0.099 | -8,644 | 0.0000 | 0.0000 | 95 |
| ENSG00000253304 | TMEM200B | 399474 | 366639.00 | -0.844 | 0.136 | -6,208 | 0.0000 | 0.0000 | 95 |
| ENSG00000105649 | RAB3A | 5864 | 67223.00 | -0.835 | 0.251 | -3,334 | 0.0009 | 0.0076 | 82 |
| ENSG00000136240 | KDELR2 | 11014 | 7,933.961 | -0.823 | 0.075 | -10,969 | 0.0000 | 0.0000 | 93 |
| ENSG00000109458 | GAB1 | 2549 | 197388.00 | -0.818 | 0.156 | -5,255 | 0.0000 | 0.0000 | 88 |
| ENSG00000122705 | CLTA | 1211 | 4,211.157 | -0.815 | 0.065 | -12,498 | 0.0000 | 0.0000 | 91 |
| ENSG00000178996 | SNX18 | 112574 | 2,378.144 | -0.798 | 0.078 | -10,284 | 0.0000 | 0.0000 | 64 |
| ENSG00000182621 | PLCB1 | 23236 | 317168.00 | -0.787 | 0.175 | -4,505 | 0.0000 | 0.0001 | 92 |
| ENSG00000108960 | MMD | 23531 | 399877.00 | -0.781 | 0.141 | -5,557 | 0.0000 | 0.0000 | 68 |
| ENSG00000147044 | CASK | 8573 | 803830.00 | -0.774 | 0.108 | -7,139 | 0.0000 | 0.0000 | 67 |
| ENSG00000134318 | ROCK2 | 9475 | 2,558.813 | -0.771 | 0.087 | -8,861 | 0.0000 | 0.0000 | 85 |
| ENSG00000166987 | MBD6 | 114785 | 607678.00 | -0.768 | 0.097 | -7,898 | 0.0000 | 0.0000 | 87 |
| ENSG00000147894 | C9orf72 | 203228 | 102923.00 | -0.766 | 0.200 | -3,840 | 0.0001 | 0.0015 | 86 |
| ENSG00000169213 | RAB3B | 5865 | 6,835.385 | -0.762 | 0.178 | -4,284 | 0.0000 | 0.0003 | 55 |
| ENSG00000204899 | MZT1 | 440145 | 712764.00 | -0.757 | 0.109 | -6,921 | 0.0000 | 0.0000 | 65 |
| ENSG00000119812 | FAM98A | 25940 | 4,218.704 | -0.756 | 0.067 | -11,304 | 0.0000 | 0.0000 | 66 |
| ENSG00000156052 | GNAQ | 2776 | 1,899.656 | -0.752 | 0.079 | -9,466 | 0.0000 | 0.0000 | 80 |
| ENSG00000184203 | PPP1R2 | 5504 | 759580.00 | -0.738 | 0.096 | -7,657 | 0.0000 | 0.0000 | 82 |
| ENSG00000170759 | KIF5B | 3799 | 8,525.711 | -0.736 | 0.067 | -10,931 | 0.0000 | 0.0000 | 86 |
| ENSG00000103051 | COG4 | 25839 | 1,453.870 | -0.735 | 0.076 | -9,703 | 0.0000 | 0.0000 | 87 |
| ENSG00000134982 | APC | 324 | 1,392.383 | -0.733 | 0.078 | -9,430 | 0.0000 | 0.0000 | 82 |
| ENSG00000110429 | FBXO3 | 26273 | 538407.00 | -0.733 | 0.101 | -7,272 | 0.0000 | 0.0000 | 78 |
| ENSG00000061987 | MON2 | 23041 | 1,279.127 | -0.728 | 0.078 | -9,312 | 0.0000 | 0.0000 | 65 |
| ENSG00000138069 | RAB1A | 5861 | 4,906.594 | -0.716 | 0.070 | -10,173 | 0.0000 | 0.0000 | 55 |
| ENSG00000173068 | BNC2 | 54796 | 598435.00 | -0.706 | 0.099 | -7,144 | 0.0000 | 0.0000 | 92 |
| ENSG00000153179 | RASSF3 | 283349 | 882839.00 | -0.706 | 0.102 | -6,898 | 0.0000 | 0.0000 | 80 |
| ENSG00000145919 | BOD1 | 91272 | 856903.00 | -0.704 | 0.094 | -7,456 | 0.0000 | 0.0000 | 96 |
| ENSG00000132507 | EIF5A | 1984 | 6,023.838 | -0.698 | 0.076 | -9,181 | 0.0000 | 0.0000 | 84 |
| ENSG00000070423 | RNF126 | 55658 | 523212.00 | -0.69 | 0.121 | -5,717 | 0.0000 | 0.0000 | 84 |
| ENSG00000132436 | FIGNL1 | 63979 | 516529.00 | -0.69 | 0.108 | -6,383 | 0.0000 | 0.0000 | 53 |
| ENSG00000178188 | SH2B1 | 25970 | 700569.00 | -0.68 | 0.099 | -6,851 | 0.0000 | 0.0000 | 85 |
| ENSG00000104643 | MTMR9 | 66036 | 544051.00 | -0.679 | 0.121 | -5,609 | 0.0000 | 0.0000 | 89 |
| ENSG00000137309 | HMGA1 | 3159 | 12,820.181 | -0.674 | 0.074 | -9,054 | 0.0000 | 0.0000 | 59 |
| ENSG00000137269 | LRRC1 | 55227 | 65580.00 | -0.667 | 0.253 | -2,636 | 0.0084 | 0.0493 | 89 |
| ENSG00000134852 | CLOCK | 9575 | 1,442.382 | -0.666 | 0.085 | -7,816 | 0.0000 | 0.0000 | 98 |
| ENSG00000055208 | TAB2 | 23118 | 1,775.667 | -0.664 | 0.071 | -9,370 | 0.0000 | 0.0000 | 91 |
| ENSG00000108829 | LRRC59 | 55379 | 6,378.630 | -0.659 | 0.060 | -10,976 | 0.0000 | 0.0000 | 62 |
| ENSG00000184216 | IRAK1 | 3654 | 4,802.596 | -0.652 | 0.071 | -9,207 | 0.0000 | 0.0000 | 83 |
| ENSG00000162104 | ADCY9 | 115 | 1,657.936 | -0.651 | 0.085 | -7,672 | 0.0000 | 0.0000 | 89 |
| ENSG00000151693 | ASAP2 | 8853 | 1,198.184 | -0.639 | 0.080 | -7,937 | 0.0000 | 0.0000 | 63 |
| ENSG00000148429 | USP6NL | 9712 | 428519.00 | -0.631 | 0.113 | -5,599 | 0.0000 | 0.0000 | 91 |
| ENSG00000174013 | FBXO45 | 200933 | 498177.00 | -0.619 | 0.111 | -5,602 | 0.0000 | 0.0000 | 73 |
| ENSG00000168374 | ARF4 | 378 | 8,982.209 | -0.618 | 0.074 | -8,337 | 0.0000 | 0.0000 | 82 |
| ENSG00000133794 | ARNTL | 406 | 539854.00 | -0.617 | 0.113 | -5,465 | 0.0000 | 0.0000 | 94 |
| ENSG00000120798 | NR2C1 | 7181 | 359156.00 | -0.616 | 0.115 | -5,341 | 0.0000 | 0.0000 | 64 |
| ENSG00000101639 | CEP192 | 55125 | 876207.00 | -0.615 | 0.090 | -6,820 | 0.0000 | 0.0000 | 91 |
| ENSG00000107897 | ACBD5 | 91452 | 1,152.409 | -0.615 | 0.083 | -7,436 | 0.0000 | 0.0000 | 87 |
| ENSG00000011114 | BTBD7 | 55727 | 916196.00 | -0.611 | 0.093 | -6,583 | 0.0000 | 0.0000 | 70 |
| ENSG00000082805 | ERC1 | 23085 | 1,961.441 | -0.607 | 0.069 | -8,751 | 0.0000 | 0.0000 | 64 |
| ENSG00000065534 | MYLK | 4638 | 5,573.350 | -0.602 | 0.090 | -6,704 | 0.0000 | 0.0000 | 54 |
| ENSG00000196981 | WDR5B | 54554 | 190237.00 | -0.6 | 0.150 | -3,997 | 0.0001 | 0.0008 | 86 |
| ENSG00000168411 | RFWD3 | 55159 | 1,283.440 | -0.599 | 0.089 | -6,723 | 0.0000 | 0.0000 | 72 |
| ENSG00000185480 | PARPBP | 55010 | 358988.00 | -0.598 | 0.119 | -5,033 | 0.0000 | 0.0000 | 68 |
| ENSG00000205189 | ZBTB10 | 65986 | 226738.00 | -0.597 | 0.143 | -4,168 | 0.0000 | 0.0004 | 61 |
| ENSG00000134970 | TMED7 | 51014 | 2,523.345 | -0.596 | 0.077 | -7,746 | 0.0000 | 0.0000 | 66 |
| ENSG00000165868 | HSPA12A | 259217 | 488958.00 | -0.591 | 0.124 | -4,773 | 0.0000 | 0.0000 | 70 |
| ENSG00000073921 | PICALM | 8301 | 6,242.455 | -0.587 | 0.062 | -9,462 | 0.0000 | 0.0000 | 78 |
| ENSG00000167522 | ANKRD11 | 29123 | 2,958.334 | -0.585 | 0.065 | -8,960 | 0.0000 | 0.0000 | 89 |
| ENSG00000156232 | WHAMM | 123720 | 447443.00 | -0.575 | 0.113 | -5,082 | 0.0000 | 0.0000 | 71 |
| ENSG00000237441 | RGL2 | 5863 | 423775.00 | -0.574 | 0.115 | -4,975 | 0.0000 | 0.0000 | 95 |
| ENSG00000135720 | DYNC1LI2 | 1783 | 3,069.176 | -0.574 | 0.075 | -7,667 | 0.0000 | 0.0000 | 66 |
| ENSG00000136504 | KAT7 | 11143 | 834002.00 | -0.549 | 0.094 | -5,839 | 0.0000 | 0.0000 | 70 |
| ENSG00000198964 | SGMS1 | 259230 | 563475.00 | -0.548 | 0.100 | -5,481 | 0.0000 | 0.0000 | 66 |
| ENSG00000134590 | RTL8C | 8933 | 3,522.291 | -0.547 | 0.082 | -6,658 | 0.0000 | 0.0000 | 78 |
| ENSG00000112941 | TENT4A | 11044 | 897110.00 | -0.536 | 0.101 | -5,311 | 0.0000 | 0.0000 | 52 |
| ENSG00000185305 | ARL15 | 54622 | 166745.00 | -0.531 | 0.162 | -3,279 | 0.0010 | 0.0090 | 68 |
| ENSG00000008282 | SYPL1 | 6856 | 3,275.158 | -0.529 | 0.075 | -7,048 | 0.0000 | 0.0000 | 89 |
| ENSG00000154945 | ANKRD40 | 91369 | 1,215.661 | -0.529 | 0.085 | -6,202 | 0.0000 | 0.0000 | 50 |
| ENSG00000149948 | HMGA2 | 8091 | 2,269.066 | -0.524 | 0.098 | -5,358 | 0.0000 | 0.0000 | 81 |
| ENSG00000073417 | PDE8A | 5151 | 696881.00 | -0.522 | 0.092 | -5,686 | 0.0000 | 0.0000 | 52 |
| ENSG00000196792 | STRN3 | 29966 | 773411.00 | -0.518 | 0.091 | -5,700 | 0.0000 | 0.0000 | 92 |
| ENSG00000116209 | TMEM59 | 9528 | 4,244.265 | -0.518 | 0.075 | -6,876 | 0.0000 | 0.0000 | 89 |
| ENSG00000137449 | CPEB2 | 132864 | 1,514.363 | -0.518 | 0.088 | -5,894 | 0.0000 | 0.0000 | 87 |
| ENSG00000126062 | TMEM115 | 11070 | 943299.00 | -0.517 | 0.083 | -6,268 | 0.0000 | 0.0000 | 51 |
| ENSG00000133026 | MYH10 | 4628 | 1,787.551 | -0.513 | 0.075 | -6,838 | 0.0000 | 0.0000 | 83 |
| ENSG00000205339 | IPO7 | 10527 | 7,107.911 | -0.511 | 0.063 | -8,060 | 0.0000 | 0.0000 | 91 |
| ENSG00000171681 | ATF7IP | 55729 | 1,154.070 | -0.494 | 0.084 | -5,902 | 0.0000 | 0.0000 | 90 |
| ENSG00000173273 | TNKS | 8658 | 1,031.139 | -0.494 | 0.084 | -5,871 | 0.0000 | 0.0000 | 88 |
| ENSG00000152242 | C18orf25 | 147339 | 820148.00 | -0.493 | 0.093 | -5,328 | 0.0000 | 0.0000 | 72 |
| ENSG00000198825 | INPP5F | 22876 | 501214.00 | -0.489 | 0.105 | -4,644 | 0.0000 | 0.0001 | 88 |
| ENSG00000101849 | TBL1X | 6907 | 816642.00 | -0.489 | 0.094 | -5,211 | 0.0000 | 0.0000 | 72 |
| ENSG00000164327 | RICTOR | 253260 | 1,066.028 | -0.473 | 0.090 | -5,230 | 0.0000 | 0.0000 | 99 |
| ENSG00000165322 | ARHGAP12 | 94134 | 1,543.054 | -0.471 | 0.081 | -5,784 | 0.0000 | 0.0000 | 70 |
| ENSG00000181789 | COPG1 | 22820 | 5,250.683 | -0.468 | 0.064 | -7,347 | 0.0000 | 0.0000 | 55 |
| ENSG00000198363 | ASPH | 444 | 8,312.864 | -0.467 | 0.065 | -7,146 | 0.0000 | 0.0000 | 56 |
| ENSG00000136478 | TEX2 | 55852 | 1,337.722 | -0.462 | 0.078 | -5,914 | 0.0000 | 0.0000 | 70 |
| ENSG00000153250 | RBMS1 | 5937 | 3,467.467 | -0.453 | 0.079 | -5,727 | 0.0000 | 0.0000 | 55 |
| ENSG00000117758 | STX12 | 23673 | 1,144.413 | -0.446 | 0.081 | -5,492 | 0.0000 | 0.0000 | 89 |
| ENSG00000150347 | ARID5B | 84159 | 1,557.239 | -0.445 | 0.091 | -4,901 | 0.0000 | 0.0000 | 80 |
| ENSG00000169554 | ZEB2 | 9839 | 2,420.393 | -0.444 | 0.094 | -4,727 | 0.0000 | 0.0000 | 100 |
| ENSG00000163820 | FYCO1 | 79443 | 2,681.196 | -0.443 | 0.091 | -4,897 | 0.0000 | 0.0000 | 69 |
| ENSG00000124214 | STAU1 | 6780 | 3,010.776 | -0.439 | 0.074 | -5,910 | 0.0000 | 0.0000 | 97 |
| ENSG00000135540 | NHSL1 | 57224 | 215178.00 | -0.436 | 0.151 | -2,891 | 0.0038 | 0.0264 | 55 |
| ENSG00000155034 | FBXL18 | 80028 | 241603.00 | -0.432 | 0.137 | -3,153 | 0.0016 | 0.0131 | 64 |
| ENSG00000176953 | NFATC2IP | 84901 | 1,147.246 | -0.425 | 0.084 | -5,060 | 0.0000 | 0.0000 | 50 |
| ENSG00000102531 | FNDC3A | 22862 | 3,815.758 | -0.424 | 0.078 | -5,405 | 0.0000 | 0.0000 | 52 |
| ENSG00000131626 | PPFIA1 | 8500 | 1,249.278 | -0.423 | 0.076 | -5,542 | 0.0000 | 0.0000 | 65 |
| ENSG00000125149 | C16orf70 | 80262 | 339890.00 | -0.42 | 0.124 | -3,401 | 0.0007 | 0.0062 | 93 |
| ENSG00000169359 | SLC33A1 | 9197 | 905607.00 | -0.42 | 0.091 | -4,615 | 0.0000 | 0.0001 | 90 |
| ENSG00000165288 | BRWD3 | 254065 | 466821.00 | -0.418 | 0.113 | -3,704 | 0.0002 | 0.0023 | 91 |
| ENSG00000120948 | TARDBP | 23435 | 1,710.029 | -0.418 | 0.079 | -5,311 | 0.0000 | 0.0000 | 91 |
| ENSG00000160305 | DIP2A | 23181 | 833088.00 | -0.416 | 0.088 | -4,745 | 0.0000 | 0.0000 | 57 |
| ENSG00000188647 | PTAR1 | 375743 | 1,603.805 | -0.415 | 0.081 | -5,133 | 0.0000 | 0.0000 | 75 |
| ENSG00000152818 | UTRN | 7402 | 2,748.606 | -0.413 | 0.073 | -5,643 | 0.0000 | 0.0000 | 95 |
| ENSG00000182095 | TNRC18 | 84629 | 2,652.695 | -0.408 | 0.068 | -6,040 | 0.0000 | 0.0000 | 91 |
| ENSG00000124782 | RREB1 | 6239 | 625157.00 | -0.408 | 0.096 | -4,242 | 0.0000 | 0.0003 | 64 |
| ENSG00000092148 | HECTD1 | 25831 | 4,457.597 | -0.382 | 0.060 | -6,337 | 0.0000 | 0.0000 | 93 |
| ENSG00000123739 | PLA2G12A | 81579 | 480432.00 | -0.375 | 0.103 | -3,633 | 0.0003 | 0.0029 | 88 |
| ENSG00000100105 | PATZ1 | 23598 | 331887.00 | -0.368 | 0.118 | -3,121 | 0.0018 | 0.0143 | 75 |
| ENSG00000114166 | KAT2B | 8850 | 938898.00 | -0.353 | 0.096 | -3,668 | 0.0002 | 0.0026 | 92 |
| ENSG00000160551 | TAOK1 | 57551 | 2,909.348 | -0.348 | 0.068 | -5,108 | 0.0000 | 0.0000 | 91 |
| ENSG00000077943 | ITGA8 | 8516 | 2,818.299 | -0.346 | 0.110 | -3,146 | 0.0017 | 0.0134 | 81 |
| ENSG00000196458 | ZNF605 | 1E+08 | 466661.00 | -0.344 | 0.111 | -3,093 | 0.0020 | 0.0154 | 52 |
| ENSG00000077254 | USP33 | 23032 | 2,124.648 | -0.336 | 0.073 | -4,617 | 0.0000 | 0.0001 | 87 |
| ENSG00000072364 | AFF4 | 27125 | 4,695.859 | -0.335 | 0.063 | -5,278 | 0.0000 | 0.0000 | 71 |
| ENSG00000166025 | AMOTL1 | 154810 | 2,283.714 | -0.331 | 0.073 | -4,545 | 0.0000 | 0.0001 | 58 |
| ENSG00000082258 | CCNT2 | 905 | 680598.00 | -0.318 | 0.095 | -3,352 | 0.0008 | 0.0073 | 80 |
| ENSG00000204439 | C6orf47 | 57827 | 414416.00 | -0.317 | 0.114 | -2,773 | 0.0056 | 0.0356 | 86 |
| ENSG00000174738 | NR1D2 | 9975 | 1,470.692 | -0.313 | 0.083 | -3,762 | 0.0002 | 0.0019 | 86 |
| ENSG00000167202 | TBC1D2B | 23102 | 1,517.561 | -0.313 | 0.086 | -3,649 | 0.0003 | 0.0028 | 73 |
| ENSG00000130382 | MLLT1 | 4298 | 1,829.465 | -0.312 | 0.072 | -4,351 | 0.0000 | 0.0002 | 51 |
| ENSG00000088832 | FKBP1A | 2280 | 7,482.594 | -0.303 | 0.068 | -4,453 | 0.0000 | 0.0001 | 64 |
| ENSG00000148516 | ZEB1 | 6935 | 2,599.540 | -0.294 | 0.073 | -4,049 | 0.0001 | 0.0007 | 81 |
| ENSG00000137331 | IER3 | 8870 | 931910.00 | -0.293 | 0.099 | -2,968 | 0.0030 | 0.0215 | 82 |
| ENSG00000117000 | RLF | 6018 | 633157.00 | -0.287 | 0.098 | -2,915 | 0.0036 | 0.0248 | 92 |
| ENSG00000100345 | MYH9 | 4627 | 33,230.804 | -0.287 | 0.079 | -3,651 | 0.0003 | 0.0028 | 51 |
| ENSG00000073969 | NSF | 4905 | 1,576.346 | -0.285 | 0.089 | -3,210 | 0.0013 | 0.0111 | 62 |
| ENSG00000198604 | BAZ1A | 11177 | 1,821.705 | -0.282 | 0.073 | -3,849 | 0.0001 | 0.0014 | 87 |
| ENSG00000106080 | FKBP14 | 55033 | 2,093.354 | -0.281 | 0.083 | -3,372 | 0.0007 | 0.0068 | 51 |
| ENSG00000213463 | SYNJ2BP | 55333 | 816960.00 | -0.278 | 0.096 | -2,898 | 0.0038 | 0.0258 | 73 |
| ENSG00000164494 | PDSS2 | 57107 | 430876.00 | -0.275 | 0.106 | -2,607 | 0.0091 | 0.0527 | 74 |
| ENSG00000070882 | OSBPL3 | 26031 | 2,176.102 | -0.268 | 0.073 | -3,640 | 0.0003 | 0.0029 | 85 |
| ENSG00000108091 | CCDC6 | 8030 | 1,612.703 | -0.267 | 0.072 | -3,722 | 0.0002 | 0.0022 | 88 |
| ENSG00000167193 | CRK | 1398 | 2,696.552 | -0.266 | 0.070 | -3,778 | 0.0002 | 0.0018 | 69 |
| ENSG00000142599 | RERE | 473 | 1,274.513 | -0.264 | 0.085 | -3,118 | 0.0018 | 0.0144 | 81 |
| ENSG00000135164 | DMTF1 | 9988 | 755181.00 | -0.253 | 0.087 | -2,911 | 0.0036 | 0.0250 | 78 |
| ENSG00000134644 | PUM1 | 9698 | 1,656.496 | -0.252 | 0.071 | -3,558 | 0.0004 | 0.0037 | 84 |
| ENSG00000165782 | PIP4P1 | 90809 | 617819.00 | -0.249 | 0.097 | -2,570 | 0.0102 | 0.0571 | 53 |
| ENSG00000196233 | LCOR | 84458 | 739215.00 | -0.247 | 0.093 | -2,662 | 0.0078 | 0.0466 | 87 |
| ENSG00000112531 | QKI | 9444 | 3,405.412 | -0.244 | 0.079 | -3,102 | 0.0019 | 0.0151 | 84 |
| ENSG00000188419 | CHM | 1121 | 1,104.583 | -0.237 | 0.081 | -2,938 | 0.0033 | 0.0234 | 78 |
| ENSG00000168685 | IL7R | 3575 | 6,080.794 | -0.23 | 0.074 | -3,096 | 0.0020 | 0.0153 | 68 |
| ENSG00000110048 | OSBP | 5007 | 1,970.623 | -0.212 | 0.068 | -3,098 | 0.0019 | 0.0152 | 95 |
| ENSG00000011566 | MAP4K3 | 8491 | 1,093.845 | -0.211 | 0.080 | -2,651 | 0.0080 | 0.0478 | 65 |
| ENSG00000184588 | PDE4B | 5142 | 1,061.342 | -0.206 | 0.080 | -2,560 | 0.0105 | 0.0582 | 86 |
| ENSG00000187079 | TEAD1 | 7003 | 5,960.069 | -0.181 | 0.058 | -3,113 | 0.0019 | 0.0146 | 68 |
| ENSG00000128585 | MKLN1 | 4289 | 1,491.668 | -0.172 | 0.074 | -2,315 | 0.0206 | 0.0966 | 60 |
| ENSG00000056097 | ZFR | 51663 | 3,117.767 | -0.167 | 0.067 | -2,471 | 0.0135 | 0.0704 | 68 |
| ENSG00000113580 | NR3C1 | 2908 | 3,505.341 | -0.164 | 0.063 | -2,623 | 0.0087 | 0.0507 | 86 |
| ENSG00000164985 | PSIP1 | 11168 | 1,740.855 | -0.161 | 0.070 | -2,315 | 0.0206 | 0.0967 | 54 |
| ENSG00000029363 | BCLAF1 | 9774 | 3,388.454 | -0.16 | 0.067 | -2,375 | 0.0175 | 0.0862 | 75 |
| ENSG00000163659 | TIPARP | 25976 | 4,185.811 | 0.163 | 0.070 | 2,323 | 0.0202 | 0.0950 | 92 |
| ENSG00000100320 | RBFOX2 | 23543 | 5,529.438 | 0.191 | 0.064 | 2,999 | 0.0027 | 0.0198 | 78 |
| ENSG00000103591 | AAGAB | 79719 | 1,383.512 | 0.196 | 0.078 | 2,521 | 0.0117 | 0.0632 | 58 |
| ENSG00000153147 | SMARCA5 | 8467 | 5,265.914 | 0.233 | 0.077 | 3,017 | 0.0026 | 0.0189 | 64 |
| ENSG00000079739 | PGM1 | 5236 | 1,211.959 | 0.255 | 0.085 | 3,003 | 0.0027 | 0.0195 | 88 |
| ENSG00000118058 | KMT2A | 4297 | 1,345.488 | 0.306 | 0.082 | 3,748 | 0.0002 | 0.0020 | 71 |
| ENSG00000170921 | TANC2 | 26115 | 960309.00 | 0.315 | 0.080 | 3,927 | 0.0001 | 0.0011 | 53 |
| ENSG00000156273 | BACH1 | 571 | 1,493.095 | 0.324 | 0.079 | 4,086 | 0.0000 | 0.0006 | 70 |
| ENSG00000091436 | MAP3K20 | 51776 | 2,738.304 | 0.346 | 0.085 | 4,092 | 0.0000 | 0.0006 | 54 |
| ENSG00000138185 | ENTPD1 | 953 | 74763.00 | 0.566 | 0.243 | 2,330 | 0.0198 | 0.0940 | 74 |
| ENSG00000248905 | FMN1 | 342184 | 1,409.721 | 0.61 | 0.096 | 6,328 | 0.0000 | 0.0000 | 79 |

| Supplementary Table 4. DEGs of miR-144-3P with target score | | | | | | | | | |
| --- | --- | --- | --- | --- | --- | --- | --- | --- | --- |
| **gene ID** | **symbol** | **entrez** | **baseMean** | **log2Fold Change** | **lfcSE** | **stat** | **pvalue** | **padj** | **Target Score** |
| ENSG00000072401 | UBE2D1 | 7321 | 558.921 | -2.327 | 0.535 | -4.353 | 0.000013 | 0.00037 | 100 |
| ENSG00000148411 | NACC2 | 138151 | 2,279.54 | -2.163 | 0.132 | -16.36 | 3.70E-60 | 4.90E-56 | 99 |
| ENSG00000133731 | IMPA1 | 3612 | 851.256 | -2.153 | 0.199 | -10.842 | 2.20E-27 | 2.90E-24 | 90 |
| ENSG00000138829 | FBN2 | 2201 | 18,912.99 | -2.093 | 0.14 | -14.938 | 1.90E-50 | 1.20E-46 | 99 |
| ENSG00000104177 | MYEF2 | 50804 | 718 | -1.615 | 0.182 | -8.851 | 8.70E-19 | 2.90E-16 | 99 |
| ENSG00000135390 | ATP5MC2 | 517 | 3,870.80 | -1.604 | 0.123 | -13.082 | 4.20E-39 | 1.80E-35 | 95 |
| ENSG00000166073 | GPR176 | 11245 | 3,732.36 | -1.568 | 0.177 | -8.843 | 9.30E-19 | 3.00E-16 | 84 |
| ENSG00000029153 | ARNTL2 | 56938 | 2,664.31 | -1.552 | 0.176 | -8.796 | 1.40E-18 | 4.30E-16 | 83 |
| ENSG00000164930 | FZD6 | 8323 | 2,307.26 | -1.516 | 0.169 | -8.958 | 3.30E-19 | 1.30E-16 | 99 |
| ENSG00000069956 | MAPK6 | 5597 | 4,741.01 | -1.47 | 0.138 | -10.632 | 2.10E-26 | 2.30E-23 | 99 |
| ENSG00000060749 | QSER1 | 79832 | 2,477.07 | -1.433 | 0.124 | -11.584 | 5.00E-31 | 9.40E-28 | 95 |
| ENSG00000138180 | CEP55 | 55165 | 2,941.21 | -1.407 | 0.134 | -10.506 | 8.10E-26 | 8.20E-23 | 90 |
| ENSG00000187098 | MITF | 4286 | 357.45 | -1.363 | 0.237 | -5.741 | 9.40E-09 | 5.70E-07 | 80 |
| ENSG00000168575 | SLC20A2 | 6575 | 1,738.22 | -1.345 | 0.117 | -11.493 | 1.40E-30 | 2.40E-27 | 99 |
| ENSG00000179456 | ZBTB18 | 10472 | 646.505 | -1.293 | 0.201 | -6.425 | 0.00E+00 | 0.00E+00 | 96 |
| ENSG00000126821 | SGPP1 | 81537 | 1,146.86 | -1.275 | 0.373 | -3.416 | 6.00E-04 | 9.70E-03 | 92 |
| ENSG00000150764 | DIXDC1 | 85458 | 598.624 | -1.267 | 0.166 | -7.629 | 0.00E+00 | 0.00E+00 | 87 |
| ENSG00000116701 | NCF2 | 4688 | 248.411 | -1.255 | 0.276 | -4.546 | 0.00E+00 | 2.00E-04 | 81 |
| ENSG00000006432 | MAP3K9 | 4293 | 80.748 | -1.252 | 0.347 | -3.609 | 3.00E-04 | 5.30E-03 | 86 |
| ENSG00000106415 | GLCCI1 | 113263 | 215.386 | -1.24 | 0.206 | -6.036 | 0.00E+00 | 0.00E+00 | 82 |
| ENSG00000113356 | POLR3G | 10622 | 892.468 | -1.236 | 0.433 | -2.851 | 4.40E-03 | 4.25E-02 | 82 |
| ENSG00000104643 | MTMR9 | 66036 | 1,219.62 | -1.226 | 0.132 | -9.273 | 0.00E+00 | 0.00E+00 | 85 |
| ENSG00000131437 | KIF3A | 11127 | 711.154 | -1.224 | 0.143 | -8.536 | 0.00E+00 | 0.00E+00 | 98 |
| ENSG00000182481 | KP＃NA2 | 3838 | 12,628.35 | -1.198 | 0.124 | -9.648 | 0.00E+00 | 0.00E+00 | 91 |
| ENSG00000167034 | NKX3-1 | 4824 | 450.067 | -1.197 | 0.152 | -7.853 | 0.00E+00 | 0.00E+00 | 93 |
| ENSG00000143179 | UCK2 | 7371 | 3,123.42 | -1.188 | 0.156 | -7.624 | 0.00E+00 | 0.00E+00 | 90 |
| ENSG00000165895 | ARHGAP42 | 143872 | 317.812 | -1.182 | 0.161 | -7.334 | 0.00E+00 | 0.00E+00 | 89 |
| ENSG00000171208 | NETO2 | 81831 | 431.823 | -1.162 | 0.186 | -6.253 | 0.00E+00 | 0.00E+00 | 93 |
| ENSG00000165029 | ABCA1 | 19 | 1,684.21 | -1.157 | 0.119 | -9.728 | 0.00E+00 | 0.00E+00 | 98 |
| ENSG00000169446 | MMGT1 | 93380 | 1,478.02 | -1.131 | 0.199 | -5.673 | 0.00E+00 | 0.00E+00 | 98 |
| ENSG00000102531 | FNDC3A | 22862 | 6,355.95 | -1.128 | 0.122 | -9.22 | 0.00E+00 | 0.00E+00 | 99 |
| ENSG00000068366 | ACSL4 | 2182 | 7,112.82 | -1.124 | 0.151 | -7.443 | 0.00E+00 | 0.00E+00 | 84 |
| ENSG00000177034 | MTX3 | 345778 | 1,271.18 | -1.118 | 0.162 | -6.913 | 0.00E+00 | 0.00E+00 | 80 |
| ENSG00000117713 | ARID1A | 8289 | 2,583.28 | -1.105 | 0.133 | -8.282 | 0.00E+00 | 0.00E+00 | 100 |
| ENSG00000178425 | NT5DC1 | 221294 | 777.05 | -1.104 | 0.142 | -7.796 | 0.00E+00 | 0.00E+00 | 90 |
| ENSG00000180228 | PRKRA | 8575 | 1,310.87 | -1.091 | 0.144 | -7.591 | 0.00E+00 | 0.00E+00 | 87 |
| ENSG00000115159 | GPD2 | 2820 | 2,867.60 | -1.075 | 0.132 | -8.174 | 0.00E+00 | 0.00E+00 | 94 |
| ENSG00000123473 | STIL | 6491 | 1,491.63 | -1.067 | 0.131 | -8.115 | 0.00E+00 | 0.00E+00 | 95 |
| ENSG00000196715 | VKORC1L1 | 154807 | 2,877.07 | -1.065 | 0.126 | -8.468 | 0.00E+00 | 0.00E+00 | 97 |
| ENSG00000182700 | IGIP | 492311 | 190.577 | -1.062 | 0.216 | -4.911 | 0.00E+00 | 0.00E+00 | 95 |
| ENSG00000170634 | ACYP2 | 98 | 337.196 | -1.057 | 0.186 | -5.676 | 0.00E+00 | 0.00E+00 | 96 |
| ENSG00000110321 | EIF4G2 | 1982 | 47,797.33 | -1.054 | 0.091 | -11.593 | 0.00E+00 | 0.00E+00 | 92 |
| ENSG00000177683 | THAP5 | 168451 | 705.532 | -1.029 | 0.218 | -4.716 | 0.00E+00 | 1.00E-04 | 83 |
| ENSG00000120693 | SMAD9 | 4093 | 294.792 | -1.027 | 0.206 | -4.98 | 0.00E+00 | 0.00E+00 | 88 |
| ENSG00000164164 | OTUD4 | 54726 | 2,922.52 | -1.02 | 0.115 | -8.891 | 0.00E+00 | 0.00E+00 | 90 |
| ENSG00000117620 | SLC35A3 | 23443 | 887.995 | -1.015 | 0.186 | -5.452 | 0.00E+00 | 0.00E+00 | 88 |
| ENSG00000135698 | MPHOSPH6 | 10200 | 1,475.17 | -1.006 | 0.142 | -7.107 | 0.00E+00 | 0.00E+00 | 80 |
| ENSG00000146376 | ARHGAP18 | 93663 | 4,485.32 | -0.991 | 0.141 | -7.03 | 0.00E+00 | 0.00E+00 | 96 |
| ENSG00000185697 | MYBL1 | 4603 | 2,261.48 | -0.989 | 0.178 | -5.545 | 0.00E+00 | 0.00E+00 | 96 |
| ENSG00000072803 | FBXW11 | 23291 | 2,638.30 | -0.989 | 0.108 | -9.168 | 0.00E+00 | 0.00E+00 | 92 |
| ENSG00000110422 | HIPK3 | 10114 | 2,926.27 | -0.982 | 0.143 | -6.852 | 0.00E+00 | 0.00E+00 | 96 |
| ENSG00000132669 | RIN2 | 54453 | 1,345.07 | -0.981 | 0.14 | -6.99 | 0.00E+00 | 0.00E+00 | 100 |
| ENSG00000198843 | SELENOT | 51714 | 4,429.72 | -0.981 | 0.172 | -5.702 | 0.00E+00 | 0.00E+00 | 98 |
| ENSG00000067900 | ROCK1 | 6093 | 5,375.73 | -0.976 | 0.106 | -9.224 | 0.00E+00 | 0.00E+00 | 91 |
| ENSG00000090447 | TFAP4 | 7023 | 184.017 | -0.94 | 0.202 | -4.657 | 0.00E+00 | 1.00E-04 | 94 |
| ENSG00000180263 | FGD6 | 55785 | 297.819 | -0.935 | 0.164 | -5.709 | 0.00E+00 | 0.00E+00 | 97 |
| ENSG00000166483 | WEE1 | 7465 | 1,767.64 | -0.895 | 0.131 | -6.849 | 0.00E+00 | 0.00E+00 | 94 |
| ENSG00000147650 | LRP12 | 29967 | 1,902.21 | -0.893 | 0.122 | -7.313 | 0.00E+00 | 0.00E+00 | 81 |
| ENSG00000061676 | NCKAP1 | 10787 | 11,218.69 | -0.867 | 0.113 | -7.648 | 0.00E+00 | 0.00E+00 | 83 |
| ENSG00000116044 | NFE2L2 | 4780 | 5,630.21 | -0.863 | 0.134 | -6.462 | 0.00E+00 | 0.00E+00 | 100 |
| ENSG00000136754 | ABI1 | 10006 | 2,635.90 | -0.85 | 0.131 | -6.496 | 0.00E+00 | 0.00E+00 | 96 |
| ENSG00000169410 | PTPN9 | 5780 | 2,680.70 | -0.845 | 0.095 | -8.869 | 0.00E+00 | 0.00E+00 | 97 |
| ENSG00000138685 | FGF2 | 2247 | 8,958.15 | -0.845 | 0.168 | -5.024 | 0.00E+00 | 0.00E+00 | 80 |
| ENSG00000155744 | FAM126B | 285172 | 488.336 | -0.843 | 0.13 | -6.487 | 0.00E+00 | 0.00E+00 | 95 |
| ENSG00000196233 | LCOR | 84458 | 1,235.59 | -0.84 | 0.123 | -6.837 | 0.00E+00 | 0.00E+00 | 87 |
| ENSG00000083312 | TNPO1 | 3842 | 16,297.33 | -0.835 | 0.101 | -8.284 | 0.00E+00 | 0.00E+00 | 100 |
| ENSG00000075415 | SLC25A3 | 5250 | 18,290.93 | -0.825 | 0.132 | -6.229 | 0.00E+00 | 0.00E+00 | 87 |
| ENSG00000167220 | HDHD2 | 84064 | 773.841 | -0.825 | 0.142 | -5.815 | 0.00E+00 | 0.00E+00 | 87 |
| ENSG00000119812 | FAM98A | 25940 | 9,355.32 | -0.787 | 0.11 | -7.134 | 0.00E+00 | 0.00E+00 | 81 |
| ENSG00000003393 | ALS2 | 57679 | 2,500.96 | -0.782 | 0.089 | -8.815 | 0.00E+00 | 0.00E+00 | 92 |
| ENSG00000144228 | SPOPL | 339745 | 725.476 | -0.781 | 0.153 | -5.116 | 0.00E+00 | 0.00E+00 | 87 |
| ENSG00000136379 | ABHD17C | 58489 | 455.778 | -0.772 | 0.184 | -4.187 | 0.00E+00 | 7.00E-04 | 90 |
| ENSG00000171827 | ZNF570 | 148268 | 308.717 | -0.768 | 0.153 | -5.012 | 0.00E+00 | 0.00E+00 | 94 |
| ENSG00000165899 | OTOGL | 283310 | 64.993 | -0.768 | 0.291 | -2.637 | 8.40E-03 | 6.86E-02 | 86 |
| ENSG00000120063 | G＃NA13 | 10672 | 3,101.60 | -0.767 | 0.129 | -5.946 | 0.00E+00 | 0.00E+00 | 86 |
| ENSG00000104205 | SGK3 | 23678 | 185.08 | -0.764 | 0.255 | -2.998 | 2.70E-03 | 2.96E-02 | 96 |
| ENSG00000112851 | ERBIN | 55914 | 6,186.76 | -0.76 | 0.125 | -6.057 | 0.00E+00 | 0.00E+00 | 90 |
| ENSG00000144357 | UBR3 | 130507 | 2,413.60 | -0.755 | 0.093 | -8.074 | 0.00E+00 | 0.00E+00 | 100 |
| ENSG00000069998 | HDHD5 | 27440 | 846.22 | -0.755 | 0.146 | -5.163 | 0.00E+00 | 0.00E+00 | 81 |
| ENSG00000168769 | TET2 | 54790 | 593.813 | -0.744 | 0.134 | -5.541 | 0.00E+00 | 0.00E+00 | 99 |
| ENSG00000105866 | SP4 | 6671 | 107.006 | -0.744 | 0.246 | -3.022 | 2.50E-03 | 2.79E-02 | 88 |
| ENSG00000108510 | MED13 | 9969 | 2,850.51 | -0.735 | 0.09 | -8.21 | 0.00E+00 | 0.00E+00 | 84 |
| ENSG00000185115 | NSMCE3 | 56160 | 1,342.80 | -0.734 | 0.122 | -6.026 | 0.00E+00 | 0.00E+00 | 96 |
| ENSG00000130052 | STARD8 | 9754 | 389.583 | -0.731 | 0.167 | -4.368 | 0.00E+00 | 4.00E-04 | 91 |
| ENSG00000102081 | FMR1 | 2332 | 1,833.20 | -0.717 | 0.154 | -4.656 | 0.00E+00 | 1.00E-04 | 87 |
| ENSG00000153094 | BCL2L11 | 10018 | 119.74 | -0.717 | 0.225 | -3.19 | 1.40E-03 | 1.82E-02 | 86 |
| ENSG00000113594 | LIFR | 3977 | 967.05 | -0.703 | 0.138 | -5.08 | 0.00E+00 | 0.00E+00 | 84 |
| ENSG00000151012 | SLC7A11 | 23657 | 10,975.09 | -0.697 | 0.143 | -4.887 | 0.00E+00 | 0.00E+00 | 96 |
| ENSG00000068305 | MEF2A | 4205 | 2,202.21 | -0.694 | 0.089 | -7.761 | 0.00E+00 | 0.00E+00 | 98 |
| ENSG00000134954 | ETS1 | 2113 | 6,690.97 | -0.692 | 0.083 | -8.359 | 0.00E+00 | 0.00E+00 | 92 |
| ENSG00000180182 | MED14 | 9282 | 1,661.36 | -0.69 | 0.092 | -7.524 | 0.00E+00 | 0.00E+00 | 85 |
| ENSG00000134138 | MEIS2 | 4212 | 841.149 | -0.689 | 0.126 | -5.472 | 0.00E+00 | 0.00E+00 | 91 |
| ENSG00000106799 | TGFBR1 | 7046 | 2,454.81 | -0.689 | 0.131 | -5.269 | 0.00E+00 | 0.00E+00 | 81 |
| ENSG00000185129 | PURA | 5813 | 1,560.75 | -0.682 | 0.152 | -4.493 | 0.00E+00 | 2.00E-04 | 91 |
| ENSG00000141380 | SS18 | 6760 | 3,090.80 | -0.673 | 0.119 | -5.68 | 0.00E+00 | 0.00E+00 | 98 |
| ENSG00000067798 | ＃NAV3 | 89795 | 4,388.90 | -0.671 | 0.093 | -7.242 | 0.00E+00 | 0.00E+00 | 86 |
| ENSG00000165490 | DDIAS | 220042 | 919.394 | -0.656 | 0.159 | -4.121 | 0.00E+00 | 9.00E-04 | 95 |
| ENSG00000105971 | CAV2 | 858 | 5,731.47 | -0.636 | 0.144 | -4.424 | 0.00E+00 | 3.00E-04 | 90 |
| ENSG00000100852 | ARHGAP5 | 394 | 3,751.78 | -0.633 | 0.121 | -5.238 | 0.00E+00 | 0.00E+00 | 90 |
| ENSG00000038382 | TRIO | 7204 | 8,957.81 | -0.627 | 0.086 | -7.315 | 0.00E+00 | 0.00E+00 | 83 |
| ENSG00000114480 | GBE1 | 2632 | 8,531.26 | -0.624 | 0.107 | -5.85 | 0.00E+00 | 0.00E+00 | 87 |
| ENSG00000113615 | SEC24A | 10802 | 3,458.28 | -0.623 | 0.089 | -6.963 | 0.00E+00 | 0.00E+00 | 92 |
| ENSG00000116771 | AGMAT | 79814 | 117.696 | -0.623 | 0.22 | -2.834 | 4.60E-03 | 4.39E-02 | 80 |
| ENSG00000151276 | MAGI1 | 9223 | 1,154.64 | -0.621 | 0.114 | -5.458 | 0.00E+00 | 0.00E+00 | 82 |
| ENSG00000174132 | FAM174A | 345757 | 449.37 | -0.62 | 0.171 | -3.63 | 3.00E-04 | 5.00E-03 | 83 |
| ENSG00000145819 | ARHGAP26 | 23092 | 256.617 | -0.614 | 0.216 | -2.842 | 4.50E-03 | 4.33E-02 | 85 |
| ENSG00000206560 | ANKRD28 | 23243 | 7,530.09 | -0.611 | 0.1 | -6.136 | 0.00E+00 | 0.00E+00 | 89 |
| ENSG00000151208 | DLG5 | 9231 | 3,081.06 | -0.611 | 0.101 | -6.054 | 0.00E+00 | 0.00E+00 | 86 |
| ENSG00000116711 | PLA2G4A | 5321 | 1,208.45 | -0.607 | 0.107 | -5.665 | 0.00E+00 | 0.00E+00 | 88 |
| ENSG00000091527 | CDV3 | 55573 | 13,524.89 | -0.605 | 0.108 | -5.606 | 0.00E+00 | 0.00E+00 | 96 |
| ENSG00000115380 | EFEMP1 | 2202 | 2,178.81 | -0.599 | 0.141 | -4.255 | 0.00E+00 | 5.00E-04 | 95 |
| ENSG00000143153 | ATP1B1 | 481 | 679.666 | -0.597 | 0.163 | -3.669 | 2.00E-04 | 4.40E-03 | 99 |
| ENSG00000166747 | AP1G1 | 164 | 5,990.87 | -0.595 | 0.081 | -7.301 | 0.00E+00 | 0.00E+00 | 91 |
| ENSG00000114439 | BBX | 56987 | 3,402.28 | -0.593 | 0.101 | -5.853 | 0.00E+00 | 0.00E+00 | 99 |
| ENSG00000169813 | HNRNPF | 3185 | 9,392.64 | -0.593 | 0.113 | -5.244 | 0.00E+00 | 0.00E+00 | 93 |
| ENSG00000182827 | ACBD3 | 64746 | 3,934.55 | -0.586 | 0.13 | -4.522 | 0.00E+00 | 2.00E-04 | 95 |
| ENSG00000185963 | BICD2 | 23299 | 3,413.46 | -0.57 | 0.104 | -5.479 | 0.00E+00 | 0.00E+00 | 81 |
| ENSG00000106780 | MEGF9 | 1955 | 2,423.78 | -0.569 | 0.1 | -5.715 | 0.00E+00 | 0.00E+00 | 82 |
| ENSG00000139324 | TMTC3 | 160418 | 2,125.17 | -0.564 | 0.12 | -4.699 | 0.00E+00 | 1.00E-04 | 95 |
| ENSG00000106462 | EZH2 | 2146 | 1,308.33 | -0.559 | 0.139 | -4.035 | 1.00E-04 | 1.30E-03 | 90 |
| ENSG00000170027 | YWHAG | 7532 | 15,797.19 | -0.554 | 0.103 | -5.362 | 0.00E+00 | 0.00E+00 | 90 |
| ENSG00000106688 | SLC1A1 | 6505 | 1,370.20 | -0.554 | 0.104 | -5.349 | 0.00E+00 | 0.00E+00 | 82 |
| ENSG00000186260 | MRTFB | 57496 | 784.324 | -0.552 | 0.135 | -4.077 | 0.00E+00 | 1.10E-03 | 98 |
| ENSG00000113851 | CRBN | 51185 | 772.326 | -0.55 | 0.117 | -4.719 | 0.00E+00 | 1.00E-04 | 88 |
| ENSG00000146676 | PURB | 5814 | 2,063.65 | -0.55 | 0.142 | -3.873 | 1.00E-04 | 2.20E-03 | 82 |
| ENSG00000187210 | GCNT1 | 2650 | 2,780.83 | -0.548 | 0.115 | -4.755 | 0.00E+00 | 1.00E-04 | 86 |
| ENSG00000150995 | ITPR1 | 3708 | 792.15 | -0.542 | 0.129 | -4.216 | 0.00E+00 | 6.00E-04 | 90 |
| ENSG00000064651 | SLC12A2 | 6558 | 1,010.27 | -0.54 | 0.121 | -4.47 | 0.00E+00 | 2.00E-04 | 99 |
| ENSG00000152455 | SUV39H2 | 79723 | 508.914 | -0.54 | 0.149 | -3.636 | 3.00E-04 | 4.90E-03 | 91 |
| ENSG00000157450 | RNF111 | 54778 | 1,548.80 | -0.54 | 0.106 | -5.098 | 0.00E+00 | 0.00E+00 | 85 |
| ENSG00000152782 | PANK1 | 53354 | 176.1 | -0.539 | 0.213 | -2.53 | 1.14E-02 | 8.58E-02 | 92 |
| ENSG00000151690 | MFSD6 | 54842 | 1,715.76 | -0.536 | 0.114 | -4.682 | 0.00E+00 | 1.00E-04 | 87 |
| ENSG00000082258 | CCNT2 | 905 | 1,105.21 | -0.534 | 0.099 | -5.395 | 0.00E+00 | 0.00E+00 | 98 |
| ENSG00000198908 | BHLHB9 | 80823 | 486.36 | -0.524 | 0.141 | -3.713 | 2.00E-04 | 3.80E-03 | 86 |
| ENSG00000130164 | LDLR | 3949 | 6,948.67 | -0.521 | 0.092 | -5.635 | 0.00E+00 | 0.00E+00 | 88 |
| ENSG00000189079 | ARID2 | 196528 | 885.96 | -0.518 | 0.125 | -4.143 | 0.00E+00 | 8.00E-04 | 99 |
| ENSG00000159216 | RUNX1 | 861 | 2,148.03 | -0.511 | 0.109 | -4.689 | 0.00E+00 | 1.00E-04 | 91 |
| ENSG00000197930 | ERO1A | 30001 | 6,533.54 | -0.511 | 0.139 | -3.685 | 2.00E-04 | 4.20E-03 | 89 |
| ENSG00000005889 | ZFX | 7543 | 1,206.91 | -0.506 | 0.097 | -5.198 | 0.00E+00 | 0.00E+00 | 98 |
| ENSG00000087303 | NID2 | 22795 | 12,058.69 | -0.5 | 0.1 | -4.98 | 0.00E+00 | 0.00E+00 | 92 |
| ENSG00000213516 | RBMXL1 | 494115 | 843.614 | -0.498 | 0.135 | -3.684 | 2.00E-04 | 4.20E-03 | 95 |
| ENSG00000104067 | TJP1 | 7082 | 5,098.51 | -0.497 | 0.1 | -4.992 | 0.00E+00 | 0.00E+00 | 97 |
| ENSG00000151233 | GXYLT1 | 283464 | 2,156.48 | -0.493 | 0.166 | -2.976 | 2.90E-03 | 3.12E-02 | 86 |
| ENSG00000100426 | ZBED4 | 9889 | 1,090.42 | -0.49 | 0.124 | -3.959 | 1.00E-04 | 1.60E-03 | 80 |
| ENSG00000173218 | VANGL1 | 81839 | 2,608.62 | -0.489 | 0.102 | -4.781 | 0.00E+00 | 1.00E-04 | 94 |
| ENSG00000077458 | FAM76B | 143684 | 312.739 | -0.479 | 0.15 | -3.201 | 1.40E-03 | 1.76E-02 | 88 |
| ENSG00000064042 | LIMCH1 | 22998 | 442.492 | -0.478 | 0.172 | -2.783 | 5.40E-03 | 4.99E-02 | 94 |
| ENSG00000119408 | NEK6 | 10783 | 5,278.56 | -0.478 | 0.089 | -5.372 | 0.00E+00 | 0.00E+00 | 80 |
| ENSG00000095015 | MAP3K1 | 4214 | 254.57 | -0.474 | 0.167 | -2.842 | 4.50E-03 | 4.33E-02 | 87 |
| ENSG00000198793 | MTOR | 2475 | 3,758.96 | -0.472 | 0.107 | -4.42 | 0.00E+00 | 3.00E-04 | 86 |
| ENSG00000123684 | LPGAT1 | 9926 | 3,326.67 | -0.466 | 0.105 | -4.453 | 0.00E+00 | 3.00E-04 | 83 |
| ENSG00000196159 | FAT4 | 79633 | 2,568.17 | -0.461 | 0.153 | -3.017 | 2.60E-03 | 2.81E-02 | 98 |
| ENSG00000139163 | ETNK1 | 55500 | 1,189.99 | -0.459 | 0.152 | -3.023 | 2.50E-03 | 2.78E-02 | 92 |
| ENSG00000010244 | ZNF207 | 7756 | 6,283.60 | -0.458 | 0.112 | -4.095 | 0.00E+00 | 1.00E-03 | 92 |
| ENSG00000167081 | PBX3 | 5090 | 3,154.00 | -0.457 | 0.126 | -3.617 | 3.00E-04 | 5.20E-03 | 83 |
| ENSG00000107779 | BMPR1A | 657 | 2,077.16 | -0.448 | 0.109 | -4.121 | 0.00E+00 | 9.00E-04 | 83 |
| ENSG00000169991 | IFFO2 | 126917 | 490.727 | -0.443 | 0.131 | -3.373 | 7.00E-04 | 1.09E-02 | 89 |
| ENSG00000131508 | UBE2D2 | 7322 | 3,580.42 | -0.442 | 0.112 | -3.941 | 1.00E-04 | 1.70E-03 | 93 |
| ENSG00000257103 | LSM14A | 26065 | 3,333.76 | -0.44 | 0.104 | -4.219 | 0.00E+00 | 6.00E-04 | 88 |
| ENSG00000077721 | UBE2A | 7319 | 5,168.53 | -0.44 | 0.11 | -4.001 | 1.00E-04 | 1.40E-03 | 87 |
| ENSG00000163577 | EIF5A2 | 56648 | 941.307 | -0.44 | 0.144 | -3.044 | 2.30E-03 | 2.65E-02 | 84 |
| ENSG00000170801 | HTRA3 | 94031 | 164.416 | -0.439 | 0.176 | -2.499 | 1.25E-02 | 9.09E-02 | 98 |
| ENSG00000188419 | CHM | 1121 | 1,993.17 | -0.438 | 0.093 | -4.705 | 0.00E+00 | 1.00E-04 | 80 |
| ENSG00000198718 | TOGARAM1 | 23116 | 990.28 | -0.433 | 0.108 | -4.021 | 1.00E-04 | 1.30E-03 | 99 |
| ENSG00000168137 | SETD5 | 55209 | 3,772.12 | -0.432 | 0.093 | -4.627 | 0.00E+00 | 1.00E-04 | 97 |
| ENSG00000106346 | USP42 | 84132 | 760.484 | -0.432 | 0.117 | -3.698 | 2.00E-04 | 4.00E-03 | 95 |
| ENSG00000151914 | DST | 667 | 15,197.53 | -0.431 | 0.148 | -2.911 | 3.60E-03 | 3.66E-02 | 91 |
| ENSG00000177125 | ZBTB34 | 403341 | 438.069 | -0.428 | 0.144 | -2.968 | 3.00E-03 | 3.19E-02 | 98 |
| ENSG00000137449 | CPEB2 | 132864 | 3,068.14 | -0.428 | 0.126 | -3.388 | 7.00E-04 | 1.04E-02 | 81 |
| ENSG00000175066 | GK5 | 256356 | 829.089 | -0.427 | 0.149 | -2.863 | 4.20E-03 | 4.13E-02 | 88 |
| ENSG00000091656 | ZFHX4 | 79776 | 1,066.46 | -0.423 | 0.166 | -2.55 | 1.08E-02 | 8.21E-02 | 90 |
| ENSG00000095564 | BTAF1 | 9044 | 1,760.20 | -0.422 | 0.095 | -4.43 | 0.00E+00 | 3.00E-04 | 97 |
| ENSG00000122483 | CCDC18 | 343099 | 804.6 | -0.413 | 0.141 | -2.92 | 3.50E-03 | 3.59E-02 | 89 |
| ENSG00000221914 | PPP2R2A | 5520 | 3,167.20 | -0.412 | 0.109 | -3.794 | 1.00E-04 | 2.90E-03 | 92 |
| ENSG00000254004 | ZNF260 | 339324 | 933.825 | -0.403 | 0.123 | -3.28 | 1.00E-03 | 1.42E-02 | 96 |
| ENSG00000114933 | INO80D | 54891 | 482.694 | -0.397 | 0.131 | -3.036 | 2.40E-03 | 2.70E-02 | 90 |
| ENSG00000157483 | MYO1E | 4643 | 3,189.16 | -0.395 | 0.102 | -3.867 | 1.00E-04 | 2.20E-03 | 93 |
| ENSG00000065183 | WDR3 | 10885 | 2,296.94 | -0.391 | 0.087 | -4.495 | 0.00E+00 | 2.00E-04 | 86 |
| ENSG00000156804 | FBXO32 | 114907 | 4,532.70 | -0.389 | 0.145 | -2.691 | 7.10E-03 | 6.15E-02 | 89 |
| ENSG00000107201 | DDX58 | 23586 | 11,516.59 | -0.386 | 0.126 | -3.065 | 2.20E-03 | 2.51E-02 | 90 |
| ENSG00000164463 | CREBRF | 153222 | 1,018.49 | -0.38 | 0.145 | -2.617 | 8.90E-03 | 7.14E-02 | 90 |
| ENSG00000090989 | EXOC1 | 55763 | 3,204.46 | -0.38 | 0.097 | -3.939 | 1.00E-04 | 1.70E-03 | 85 |
| ENSG00000147533 | GOLGA7 | 51125 | 2,417.98 | -0.38 | 0.122 | -3.102 | 1.90E-03 | 2.27E-02 | 82 |
| ENSG00000183354 | KIAA2026 | 158358 | 1,170.13 | -0.375 | 0.112 | -3.344 | 8.00E-04 | 1.18E-02 | 89 |
| ENSG00000185551 | NR2F2 | 7026 | 1,571.36 | -0.371 | 0.098 | -3.773 | 2.00E-04 | 3.10E-03 | 91 |
| ENSG00000086189 | DIMT1 | 27292 | 1,689.72 | -0.366 | 0.128 | -2.859 | 4.30E-03 | 4.17E-02 | 88 |
| ENSG00000148516 | ZEB1 | 6935 | 5,669.73 | -0.362 | 0.107 | -3.378 | 7.00E-04 | 1.08E-02 | 94 |
| ENSG00000070961 | ATP2B1 | 490 | 8,145.68 | -0.36 | 0.111 | -3.257 | 1.10E-03 | 1.51E-02 | 88 |
| ENSG00000057019 | DCBLD2 | 131566 | 17,181.24 | -0.354 | 0.129 | -2.75 | 6.00E-03 | 5.41E-02 | 87 |
| ENSG00000174804 | FZD4 | 8322 | 1,091.10 | -0.348 | 0.11 | -3.166 | 1.50E-03 | 1.92E-02 | 86 |
| ENSG00000064999 | ANKS1A | 23294 | 1,050.84 | -0.341 | 0.125 | -2.736 | 6.20E-03 | 5.59E-02 | 86 |
| ENSG00000134363 | FST | 10468 | 25,321.73 | -0.34 | 0.126 | -2.708 | 6.80E-03 | 5.92E-02 | 93 |
| ENSG00000007168 | PAFAH1B1 | 5048 | 8,338.43 | -0.337 | 0.097 | -3.477 | 5.00E-04 | 8.00E-03 | 93 |
| ENSG00000144747 | TMF1 | 7110 | 3,256.48 | -0.337 | 0.084 | -4.033 | 1.00E-04 | 1.30E-03 | 89 |
| ENSG00000149289 | ZC3H12C | 85463 | 1,723.81 | -0.334 | 0.098 | -3.395 | 7.00E-04 | 1.02E-02 | 85 |
| ENSG00000100664 | EIF5 | 1983 | 16,654.00 | -0.328 | 0.086 | -3.813 | 1.00E-04 | 2.70E-03 | 84 |
| ENSG00000196792 | STRN3 | 29966 | 1,554.45 | -0.327 | 0.107 | -3.068 | 2.20E-03 | 2.49E-02 | 92 |
| ENSG00000184254 | ALDH1A3 | 220 | 1,103.62 | -0.32 | 0.127 | -2.518 | 1.18E-02 | 8.77E-02 | 94 |
| ENSG00000173276 | ZBTB21 | 49854 | 1,141.06 | -0.318 | 0.108 | -2.949 | 3.20E-03 | 3.34E-02 | 95 |
| ENSG00000136143 | SUCLA2 | 8803 | 2,503.17 | -0.317 | 0.108 | -2.941 | 3.30E-03 | 3.40E-02 | 88 |
| ENSG00000115020 | PIKFYVE | 200576 | 2,523.24 | -0.31 | 0.1 | -3.094 | 2.00E-03 | 2.31E-02 | 84 |
| ENSG00000115808 | STRN | 6801 | 2,289.15 | -0.307 | 0.095 | -3.225 | 1.30E-03 | 1.65E-02 | 92 |
| ENSG00000079819 | EPB41L2 | 2037 | 4,739.29 | -0.296 | 0.085 | -3.485 | 5.00E-04 | 7.80E-03 | 84 |
| ENSG00000132964 | CDK8 | 1024 | 1,018.19 | -0.295 | 0.102 | -2.9 | 3.70E-03 | 3.77E-02 | 94 |
| ENSG00000102038 | SMARCA1 | 6594 | 4,115.80 | -0.294 | 0.09 | -3.258 | 1.10E-03 | 1.51E-02 | 84 |
| ENSG00000173065 | FAM222B | 55731 | 927.164 | -0.285 | 0.114 | -2.489 | 1.28E-02 | 9.27E-02 | 91 |
| ENSG00000141664 | ZCCHC2 | 54877 | 1,120.46 | -0.28 | 0.108 | -2.604 | 9.20E-03 | 7.36E-02 | 96 |
| ENSG00000107562 | CXCL12 | 6387 | 7,272.35 | -0.278 | 0.096 | -2.893 | 3.80E-03 | 3.83E-02 | 92 |
| ENSG00000163848 | ZNF148 | 7707 | 2,020.02 | -0.275 | 0.097 | -2.839 | 4.50E-03 | 4.36E-02 | 90 |
| ENSG00000118007 | STAG1 | 10274 | 3,269.44 | -0.275 | 0.091 | -3.02 | 2.50E-03 | 2.80E-02 | 81 |
| ENSG00000166326 | TRIM44 | 54765 | 6,101.99 | -0.272 | 0.082 | -3.3 | 1.00E-03 | 1.35E-02 | 91 |
| ENSG00000153187 | HNRNPU | 3192 | 20,008.61 | -0.271 | 0.083 | -3.267 | 1.10E-03 | 1.47E-02 | 82 |
| ENSG00000136810 | TXN | 7295 | 22,533.49 | -0.251 | 0.094 | -2.661 | 7.80E-03 | 6.51E-02 | 98 |
| ENSG00000169967 | MAP3K2 | 10746 | 2,276.74 | -0.247 | 0.096 | -2.58 | 9.90E-03 | 7.75E-02 | 85 |
| ENSG00000135316 | SYNCRIP | 10492 | 11,346.31 | -0.24 | 0.088 | -2.716 | 6.60E-03 | 5.81E-02 | 85 |
| ENSG00000155816 | FMN2 | 56776 | 3,422.93 | -0.205 | 0.084 | -2.452 | 1.42E-02 | 9.96E-02 | 84 |
| ENSG00000143702 | CEP170 | 9859 | 4,627.39 | 0.307 | 0.121 | 2.528 | 1.15E-02 | 8.60E-02 | 83 |
| ENSG00000116132 | PRRX1 | 5396 | 42,090.98 | 0.314 | 0.087 | 3.618 | 3.00E-04 | 5.20E-03 | 93 |
| ENSG00000152661 | GJA1 | 2697 | 33,298.92 | 0.345 | 0.108 | 3.185 | 1.40E-03 | 1.84E-02 | 85 |
| ENSG00000171862 | PTEN | 5728 | 5,196.62 | 0.349 | 0.12 | 2.899 | 3.70E-03 | 3.78E-02 | 88 |
| ENSG00000005812 | FBXL3 | 26224 | 1,124.88 | 0.369 | 0.146 | 2.53 | 1.14E-02 | 8.57E-02 | 90 |
| ENSG00000123094 | RASSF8 | 11228 | 6,604.24 | 0.529 | 0.128 | 4.125 | 0.00E+00 | 9.00E-04 | 89 |
| ENSG00000116128 | BCL9 | 607 | 1,015.78 | 0.566 | 0.158 | 3.595 | 3.00E-04 | 5.60E-03 | 92 |
| ENSG00000018408 | WWTR1 | 25937 | 4,608.47 | 0.647 | 0.124 | 5.238 | 0.00E+00 | 0.00E+00 | 82 |
| ENSG00000137872 | SEMA6D | 80031 | 816.905 | 0.679 | 0.187 | 3.631 | 3.00E-04 | 4.90E-03 | 95 |
| ENSG00000169851 | PCDH7 | 5099 | 1,541.62 | 0.77 | 0.184 | 4.196 | 0.00E+00 | 7.00E-04 | 90 |
| ENSG00000266524 | GDF10 | 2662 | 63.41 | 2.035 | 0.514 | 3.963 | 1.00E-04 | 1.60E-03 | 94 |
| ENSG00000197226 | TBC1D9B | 23061 | 6,994.39 | -0.267 | 0.095 | -2.81 | 5.00E-03 | 4.66E-02 | 80 |
| ENSG00000143815 | LBR | 3930 | 1,466.12 | -1.248 | 0.141 | -8.851 | 0.00E+00 | 0.00E+00 | 79 |
| ENSG00000115468 | EFHD1 | 80303 | 55.005 | -1.246 | 0.471 | -2.647 | 8.10E-03 | 6.73E-02 | 79 |
| ENSG00000120137 | PANK3 | 79646 | 2,757.99 | -0.885 | 0.162 | -5.455 | 0.00E+00 | 0.00E+00 | 79 |
| ENSG00000205730 | ITPRIPL2 | 162073 | 2,973.26 | -0.871 | 0.133 | -6.527 | 0.00E+00 | 0.00E+00 | 79 |
| ENSG00000157741 | UBN2 | 254048 | 632.858 | -0.5 | 0.125 | -3.992 | 1.00E-04 | 1.50E-03 | 79 |
| ENSG00000146223 | RPL7L1 | 285855 | 7,137.79 | -0.286 | 0.084 | -3.424 | 6.00E-04 | 9.40E-03 | 79 |
| ENSG00000070413 | DGCR2 | 9993 | 3,167.36 | -0.929 | 0.09 | -10.313 | 0.00E+00 | 0.00E+00 | 78 |
| ENSG00000177426 | TGIF1 | 7050 | 2,338.86 | -0.643 | 0.127 | -5.063 | 0.00E+00 | 0.00E+00 | 78 |
| ENSG00000116675 | D＃NAJC6 | 9829 | 2,381.36 | -0.559 | 0.127 | -4.391 | 0.00E+00 | 3.00E-04 | 78 |
| ENSG00000101189 | MRGBP | 55257 | 1,065.19 | -0.521 | 0.106 | -4.915 | 0.00E+00 | 0.00E+00 | 78 |
| ENSG00000119900 | OGFRL1 | 79627 | 2,627.05 | -0.464 | 0.11 | -4.213 | 0.00E+00 | 6.00E-04 | 78 |
| ENSG00000197763 | TXNRD3 | 114112 | 433.511 | -0.447 | 0.139 | -3.213 | 1.30E-03 | 1.70E-02 | 78 |
| ENSG00000150347 | ARID5B | 84159 | 3,104.41 | -0.408 | 0.129 | -3.175 | 1.50E-03 | 1.88E-02 | 78 |
| ENSG00000058729 | RIOK2 | 55781 | 1,938.64 | -0.285 | 0.091 | -3.116 | 1.80E-03 | 2.18E-02 | 78 |
| ENSG00000155850 | SLC26A2 | 1836 | 1,706.51 | -1.065 | 0.143 | -7.434 | 0.00E+00 | 0.00E+00 | 77 |
| ENSG00000089057 | SLC23A2 | 9962 | 1,783.85 | -0.925 | 0.115 | -8.046 | 0.00E+00 | 0.00E+00 | 77 |
| ENSG00000113161 | HMGCR | 3156 | 3,002.17 | -0.85 | 0.113 | -7.503 | 0.00E+00 | 0.00E+00 | 77 |
| ENSG00000136928 | GABBR2 | 9568 | 984.729 | -0.842 | 0.179 | -4.707 | 0.00E+00 | 1.00E-04 | 77 |
| ENSG00000107929 | LARP4B | 23185 | 3,393.01 | -0.79 | 0.083 | -9.511 | 0.00E+00 | 0.00E+00 | 77 |
| ENSG00000087053 | MTMR2 | 8898 | 3,804.32 | -0.74 | 0.115 | -6.414 | 0.00E+00 | 0.00E+00 | 77 |
| ENSG00000117479 | SLC19A2 | 10560 | 326.528 | -0.46 | 0.169 | -2.72 | 6.50E-03 | 5.77E-02 | 77 |
| ENSG00000011258 | MBTD1 | 54799 | 329.695 | -0.393 | 0.137 | -2.874 | 4.10E-03 | 4.02E-02 | 77 |
| ENSG00000144724 | PTPRG | 5793 | 4,516.13 | -0.347 | 0.086 | -4.05 | 1.00E-04 | 1.20E-03 | 77 |
| ENSG00000044524 | EPHA3 | 2042 | 384.553 | 1.598 | 0.583 | 2.74 | 6.10E-03 | 5.53E-02 | 77 |
| ENSG00000101447 | FAM83D | 81610 | 1,944.33 | -0.96 | 0.123 | -7.796 | 0.00E+00 | 0.00E+00 | 76 |
| ENSG00000048740 | CELF2 | 10659 | 1,533.39 | -0.797 | 0.172 | -4.641 | 0.00E+00 | 1.00E-04 | 76 |
| ENSG00000118200 | CAMSAP2 | 23271 | 3,713.94 | -0.566 | 0.103 | -5.52 | 0.00E+00 | 0.00E+00 | 76 |
| ENSG00000131374 | TBC1D5 | 9779 | 3,239.19 | -0.236 | 0.089 | -2.645 | 8.20E-03 | 6.75E-02 | 76 |
| ENSG00000132718 | SYT11 | 23208 | 3,640.90 | 0.288 | 0.108 | 2.68 | 7.40E-03 | 6.28E-02 | 76 |
| ENSG00000119541 | VPS4B | 9525 | 2,236.36 | -1.431 | 0.15 | -9.523 | 1.70E-21 | 9.60E-19 | 75 |
| ENSG00000105879 | CBLL1 | 79872 | 940.487 | -0.553 | 0.131 | -4.222 | 0.00E+00 | 6.00E-04 | 75 |
| ENSG00000176623 | RMDN1 | 51115 | 2,565.47 | -0.405 | 0.096 | -4.215 | 0.00E+00 | 6.00E-04 | 75 |
| ENSG00000054267 | ARID4B | 51742 | 1,679.79 | -0.394 | 0.094 | -4.168 | 0.00E+00 | 8.00E-04 | 75 |
| ENSG00000139793 | MBNL2 | 10150 | 1,705.41 | -0.393 | 0.116 | -3.378 | 7.00E-04 | 1.08E-02 | 75 |
| ENSG00000153317 | ASAP1 | 50807 | 7,637.91 | -0.332 | 0.086 | -3.861 | 1.00E-04 | 2.30E-03 | 75 |
| ENSG00000156639 | ZFAND3 | 60685 | 5,116.14 | 0.357 | 0.1 | 3.56 | 4.00E-04 | 6.30E-03 | 75 |
| ENSG00000100697 | DICER1 | 23405 | 2,773.09 | -0.571 | 0.096 | -5.933 | 0.00E+00 | 0.00E+00 | 74 |
| ENSG00000275835 | TUBGCP5 | 114791 | 1,260.29 | -0.432 | 0.108 | -4.014 | 1.00E-04 | 1.30E-03 | 74 |
| ENSG00000134851 | TMEM165 | 55858 | 4,260.87 | -0.912 | 0.109 | -8.396 | 0.00E+00 | 0.00E+00 | 73 |
| ENSG00000116747 | RO60 | 6738 | 1,365.93 | -0.701 | 0.155 | -4.522 | 0.00E+00 | 2.00E-04 | 73 |
| ENSG00000167005 | NUDT21 | 11051 | 4,133.42 | -0.637 | 0.159 | -4.009 | 1.00E-04 | 1.40E-03 | 73 |
| ENSG00000108468 | CBX1 | 10951 | 4,513.41 | -0.549 | 0.087 | -6.321 | 0.00E+00 | 0.00E+00 | 73 |
| ENSG00000213853 | EMP2 | 2013 | 1,254.61 | -0.518 | 0.129 | -4.027 | 1.00E-04 | 1.30E-03 | 73 |
| ENSG00000136485 | DCAF7 | 10238 | 4,435.03 | -0.456 | 0.102 | -4.457 | 0.00E+00 | 3.00E-04 | 73 |
| ENSG00000162105 | SHANK2 | 22941 | 512.52 | -0.437 | 0.147 | -2.97 | 3.00E-03 | 3.17E-02 | 73 |
| ENSG00000181827 | RFX7 | 64864 | 1,006.42 | -0.405 | 0.107 | -3.772 | 2.00E-04 | 3.10E-03 | 73 |
| ENSG00000091409 | ITGA6 | 3655 | 2,863.51 | -1.786 | 0.178 | -10.039 | 1.00E-23 | 8.50E-21 | 72 |
| ENSG00000105176 | URI1 | 8725 | 2,353.47 | -1.204 | 0.18 | -6.697 | 0.00E+00 | 0.00E+00 | 72 |
| ENSG00000155111 | CDK19 | 23097 | 883.561 | -0.642 | 0.122 | -5.273 | 0.00E+00 | 0.00E+00 | 72 |
| ENSG00000169914 | OTUD3 | 23252 | 622.337 | -0.626 | 0.129 | -4.844 | 0.00E+00 | 0.00E+00 | 72 |
| ENSG00000272886 | DCP1A | 55802 | 1,468.71 | -0.488 | 0.102 | -4.783 | 0.00E+00 | 1.00E-04 | 72 |
| ENSG00000100722 | ZC3H14 | 79882 | 2,578.28 | -0.455 | 0.091 | -5.022 | 0.00E+00 | 0.00E+00 | 72 |
| ENSG00000123342 | MMP19 | 4327 | 1,011.12 | -1.119 | 0.121 | -9.247 | 0.00E+00 | 0.00E+00 | 71 |
| ENSG00000111846 | GCNT2 | 2651 | 184.138 | -0.901 | 0.227 | -3.968 | 1.00E-04 | 1.60E-03 | 71 |
| ENSG00000133059 | DSTYK | 25778 | 2,728.93 | -0.487 | 0.099 | -4.934 | 0.00E+00 | 0.00E+00 | 71 |
| ENSG00000114450 | GNB4 | 59345 | 2,820.47 | -0.433 | 0.124 | -3.491 | 5.00E-04 | 7.70E-03 | 71 |
| ENSG00000197128 | ZNF772 | 400720 | 428.335 | -0.408 | 0.126 | -3.239 | 1.20E-03 | 1.58E-02 | 71 |
| ENSG00000139291 | TMEM19 | 55266 | 1,028.57 | -0.396 | 0.151 | -2.617 | 8.90E-03 | 7.14E-02 | 71 |
| ENSG00000106034 | CPED1 | 79974 | 3,241.76 | -0.274 | 0.09 | -3.029 | 2.50E-03 | 2.75E-02 | 71 |
| ENSG00000122884 | P4HA1 | 5033 | 6,608.17 | -1.004 | 0.179 | -5.598 | 0.00E+00 | 0.00E+00 | 70 |
| ENSG00000160877 | ＃NACC1 | 112939 | 4,380.89 | -0.929 | 0.103 | -9.027 | 0.00E+00 | 0.00E+00 | 70 |
| ENSG00000065989 | PDE4A | 5141 | 598.421 | -0.591 | 0.151 | -3.905 | 1.00E-04 | 2.00E-03 | 70 |
| ENSG00000156531 | PHF6 | 84295 | 1,602.99 | -0.58 | 0.15 | -3.867 | 1.00E-04 | 2.20E-03 | 70 |
| ENSG00000175305 | CCNE2 | 9134 | 296.366 | -0.575 | 0.224 | -2.564 | 1.03E-02 | 8.03E-02 | 70 |
| ENSG00000188807 | TMEM201 | 199953 | 765.427 | -0.465 | 0.171 | -2.718 | 6.60E-03 | 5.80E-02 | 70 |
| ENSG00000111817 | DSE | 29940 | 3,384.83 | -0.378 | 0.113 | -3.355 | 8.00E-04 | 1.15E-02 | 70 |
| ENSG00000164576 | SAP30L | 79685 | 930.862 | -0.337 | 0.127 | -2.65 | 8.10E-03 | 6.69E-02 | 70 |
| ENSG00000065882 | TBC1D1 | 23216 | 1,806.73 | -0.323 | 0.111 | -2.901 | 3.70E-03 | 3.76E-02 | 70 |
| ENSG00000121741 | ZMYM2 | 7750 | 1,881.14 | -0.308 | 0.099 | -3.097 | 2.00E-03 | 2.30E-02 | 70 |
| ENSG00000185728 | YTHDF3 | 253943 | 4,676.84 | 0.423 | 0.113 | 3.728 | 2.00E-04 | 3.60E-03 | 70 |
| ENSG00000181690 | PLAG1 | 5324 | 319.262 | 0.594 | 0.196 | 3.028 | 2.50E-03 | 2.75E-02 | 70 |
| ENSG00000080493 | SLC4A4 | 8671 | 2,003.19 | -1.009 | 0.103 | -9.814 | 0.00E+00 | 0.00E+00 | 69 |
| ENSG00000129518 | EAPP | 55837 | 1,023.98 | -0.682 | 0.135 | -5.07 | 0.00E+00 | 0.00E+00 | 69 |
| ENSG00000184602 | SNN | 8303 | 1,923.12 | -0.668 | 0.111 | -6.031 | 0.00E+00 | 0.00E+00 | 69 |
| ENSG00000137770 | CTDSPL2 | 51496 | 1,071.93 | -0.624 | 0.124 | -5.026 | 0.00E+00 | 0.00E+00 | 69 |
| ENSG00000168958 | MFF | 56947 | 4,032.17 | -0.583 | 0.115 | -5.074 | 0.00E+00 | 0.00E+00 | 69 |
| ENSG00000078902 | TOLLIP | 54472 | 3,286.55 | -0.476 | 0.101 | -4.714 | 0.00E+00 | 1.00E-04 | 69 |
| ENSG00000119396 | RAB14 | 51552 | 5,419.78 | -0.463 | 0.146 | -3.179 | 1.50E-03 | 1.86E-02 | 69 |
| ENSG00000139977 | ＃NAA30 | 122830 | 1,239.79 | -0.456 | 0.163 | -2.8 | 5.10E-03 | 4.77E-02 | 69 |
| ENSG00000198492 | YTHDF2 | 51441 | 3,819.27 | -0.421 | 0.095 | -4.444 | 0.00E+00 | 3.00E-04 | 69 |
| ENSG00000134318 | ROCK2 | 9475 | 4,688.53 | -0.396 | 0.097 | -4.088 | 0.00E+00 | 1.00E-03 | 69 |
| ENSG00000138669 | PRKG2 | 5593 | 980.078 | -0.752 | 0.134 | -5.596 | 0.00E+00 | 0.00E+00 | 68 |
| ENSG00000170365 | SMAD1 | 4086 | 393.317 | -0.69 | 0.135 | -5.099 | 0.00E+00 | 0.00E+00 | 68 |
| ENSG00000163939 | PBRM1 | 55193 | 3,834.64 | -0.616 | 0.102 | -6.041 | 0.00E+00 | 0.00E+00 | 68 |
| ENSG00000157657 | ZNF618 | 114991 | 1,727.52 | -0.553 | 0.12 | -4.592 | 0.00E+00 | 1.00E-04 | 68 |
| ENSG00000109184 | DCUN1D4 | 23142 | 1,790.18 | -0.471 | 0.127 | -3.71 | 2.00E-04 | 3.80E-03 | 68 |
| ENSG00000091640 | SPAG7 | 9552 | 1,998.62 | -0.465 | 0.132 | -3.526 | 4.00E-04 | 7.00E-03 | 68 |
| ENSG00000189308 | LIN54 | 132660 | 1,246.43 | -0.462 | 0.106 | -4.347 | 0.00E+00 | 4.00E-04 | 68 |
| ENSG00000120129 | DUSP1 | 1843 | 4,958.07 | -0.413 | 0.095 | -4.36 | 0.00E+00 | 4.00E-04 | 68 |
| ENSG00000170759 | KIF5B | 3799 | 15,934.50 | -0.385 | 0.092 | -4.199 | 0.00E+00 | 7.00E-04 | 68 |
| ENSG00000109332 | UBE2D3 | 7323 | 12,644.74 | -0.362 | 0.14 | -2.59 | 9.60E-03 | 7.61E-02 | 68 |
| ENSG00000069329 | VPS35 | 55737 | 8,727.94 | -0.346 | 0.083 | -4.171 | 0.00E+00 | 8.00E-04 | 68 |
| ENSG00000159131 | GART | 2618 | 3,978.72 | -0.229 | 0.083 | -2.764 | 5.70E-03 | 5.21E-02 | 68 |
| ENSG00000113369 | ARRDC3 | 57561 | 3,106.98 | 0.296 | 0.1 | 2.958 | 3.10E-03 | 3.26E-02 | 68 |
| ENSG00000158985 | CDC42SE2 | 56990 | 1,190.57 | -0.866 | 0.168 | -5.155 | 0.00E+00 | 0.00E+00 | 67 |
| ENSG00000168813 | ZNF507 | 22847 | 1,134.04 | -0.735 | 0.097 | -7.604 | 0.00E+00 | 0.00E+00 | 67 |
| ENSG00000153147 | SMARCA5 | 8467 | 7,261.71 | -0.672 | 0.077 | -8.766 | 0.00E+00 | 0.00E+00 | 67 |
| ENSG00000164219 | PGGT1B | 5229 | 605.551 | -1.173 | 0.167 | -7.007 | 0.00E+00 | 0.00E+00 | 66 |
| ENSG00000120526 | NUDCD1 | 84955 | 2,226.29 | -0.821 | 0.123 | -6.691 | 0.00E+00 | 0.00E+00 | 66 |
| ENSG00000113583 | C5orf15 | 56951 | 4,176.87 | -0.53 | 0.142 | -3.726 | 2.00E-04 | 3.60E-03 | 66 |
| ENSG00000145715 | RASA1 | 5921 | 3,418.69 | -0.359 | 0.108 | -3.324 | 9.00E-04 | 1.26E-02 | 66 |
| ENSG00000177311 | ZBTB38 | 253461 | 7,076.61 | -0.8 | 0.094 | -8.529 | 0.00E+00 | 0.00E+00 | 65 |
| ENSG00000197121 | PGAP1 | 80055 | 742.713 | -0.526 | 0.159 | -3.301 | 1.00E-03 | 1.35E-02 | 65 |
| ENSG00000001084 | GCLC | 2729 | 1,260.44 | -0.337 | 0.137 | -2.464 | 1.37E-02 | 9.71E-02 | 65 |
| ENSG00000114395 | CYB561D2 | 11068 | 615.84 | -0.333 | 0.115 | -2.886 | 3.90E-03 | 3.89E-02 | 65 |
| ENSG00000078237 | TIGAR | 57103 | 1,529.87 | -0.31 | 0.107 | -2.887 | 3.90E-03 | 3.89E-02 | 65 |
| ENSG00000163629 | PTPN13 | 5783 | 3,768.45 | 0.806 | 0.151 | 5.34 | 0.00E+00 | 0.00E+00 | 65 |
| ENSG00000196396 | PTPN1 | 5770 | 2,936.25 | 0.968 | 0.12 | 8.061 | 0.00E+00 | 0.00E+00 | 65 |
| ENSG00000156675 | RAB11FIP1 | 80223 | 164.51 | -2.372 | 0.24 | -9.893 | 4.40E-23 | 3.30E-20 | 64 |
| ENSG00000115339 | GALNT3 | 2591 | 312.709 | -1.297 | 0.271 | -4.792 | 0.00E+00 | 1.00E-04 | 64 |
| ENSG00000091317 | CMTM6 | 54918 | 4,529.26 | -1.18 | 0.297 | -3.977 | 1.00E-04 | 1.50E-03 | 64 |
| ENSG00000124788 | ATXN1 | 6310 | 1,388.80 | -0.855 | 0.134 | -6.382 | 0.00E+00 | 0.00E+00 | 64 |
| ENSG00000163644 | PPM1K | 152926 | 1,368.90 | -0.745 | 0.141 | -5.293 | 0.00E+00 | 0.00E+00 | 64 |
| ENSG00000170448 | NFXL1 | 152518 | 370.544 | -0.519 | 0.158 | -3.285 | 1.00E-03 | 1.41E-02 | 64 |
| ENSG00000025039 | RRAGD | 58528 | 381.628 | -0.496 | 0.198 | -2.497 | 1.25E-02 | 9.13E-02 | 64 |
| ENSG00000224470 | ATXN1L | 342371 | 2,354.36 | -0.486 | 0.132 | -3.695 | 2.00E-04 | 4.00E-03 | 64 |
| ENSG00000101190 | TCFL5 | 10732 | 605.293 | -0.426 | 0.126 | -3.375 | 7.00E-04 | 1.08E-02 | 64 |
| ENSG00000165671 | NSD1 | 64324 | 3,296.90 | -0.677 | 0.12 | -5.635 | 0.00E+00 | 0.00E+00 | 63 |
| ENSG00000072210 | ALDH3A2 | 224 | 3,214.63 | -0.53 | 0.111 | -4.786 | 0.00E+00 | 1.00E-04 | 63 |
| ENSG00000145050 | MANF | 7873 | 3,357.73 | -0.505 | 0.114 | -4.418 | 0.00E+00 | 3.00E-04 | 63 |
| ENSG00000163625 | WDFY3 | 23001 | 2,698.01 | -0.424 | 0.107 | -3.95 | 1.00E-04 | 1.70E-03 | 63 |
| ENSG00000172466 | ZNF24 | 7572 | 2,262.03 | -0.417 | 0.11 | -3.786 | 2.00E-04 | 3.00E-03 | 63 |
| ENSG00000196950 | SLC39A10 | 57181 | 1,415.32 | -0.371 | 0.133 | -2.784 | 5.40E-03 | 4.98E-02 | 63 |
| ENSG00000159658 | EFCAB14 | 9813 | 6,815.92 | -0.338 | 0.082 | -4.142 | 0.00E+00 | 8.00E-04 | 63 |
| ENSG00000107186 | MPDZ | 8777 | 1,496.00 | -0.333 | 0.119 | -2.788 | 5.30E-03 | 4.92E-02 | 63 |
| ENSG00000111912 | NCOA7 | 135112 | 1,863.55 | -0.318 | 0.126 | -2.517 | 1.18E-02 | 8.78E-02 | 63 |
| ENSG00000065243 | PKN2 | 5586 | 2,548.98 | -0.276 | 0.103 | -2.681 | 7.30E-03 | 6.28E-02 | 63 |
| ENSG00000152495 | CAMK4 | 814 | 339.45 | 0.499 | 0.186 | 2.689 | 7.20E-03 | 6.17E-02 | 63 |
| ENSG00000164548 | TRA2A | 29896 | 2,002.08 | -0.686 | 0.149 | -4.603 | 0.00E+00 | 1.00E-04 | 62 |
| ENSG00000156261 | CCT8 | 10694 | 10,188.80 | -0.28 | 0.097 | -2.897 | 3.80E-03 | 3.79E-02 | 62 |
| ENSG00000168214 | RBPJ | 3516 | 5,146.15 | 0.589 | 0.16 | 3.685 | 2.00E-04 | 4.20E-03 | 62 |
| ENSG00000147894 | C9orf72 | 203228 | 186.115 | -1.07 | 0.18 | -5.929 | 0.00E+00 | 0.00E+00 | 61 |
| ENSG00000138166 | DUSP5 | 1847 | 3,613.69 | -0.795 | 0.126 | -6.335 | 0.00E+00 | 0.00E+00 | 61 |
| ENSG00000175203 | DCTN2 | 10540 | 5,878.92 | -0.516 | 0.094 | -5.501 | 0.00E+00 | 0.00E+00 | 61 |
| ENSG00000123836 | PFKFB2 | 5208 | 474.727 | -0.397 | 0.146 | -2.729 | 6.30E-03 | 5.67E-02 | 61 |
| ENSG00000025796 | SEC63 | 11231 | 6,967.70 | -0.318 | 0.097 | -3.266 | 1.10E-03 | 1.48E-02 | 61 |
| ENSG00000006576 | PHTF2 | 57157 | 2,828.25 | -0.297 | 0.117 | -2.529 | 1.14E-02 | 8.59E-02 | 61 |
| ENSG00000075539 | FRYL | 285527 | 2,270.19 | -0.288 | 0.105 | -2.739 | 6.20E-03 | 5.55E-02 | 61 |
| ENSG00000105810 | CDK6 | 1021 | 7,878.01 | -0.745 | 0.135 | -5.502 | 0.00E+00 | 0.00E+00 | 60 |
| ENSG00000101290 | CDS2 | 8760 | 2,344.86 | -0.665 | 0.106 | -6.277 | 0.00E+00 | 0.00E+00 | 60 |
| ENSG00000106261 | ZKSCAN1 | 7586 | 3,576.19 | -0.599 | 0.088 | -6.783 | 0.00E+00 | 0.00E+00 | 60 |
| ENSG00000103404 | USP31 | 57478 | 705.51 | -0.528 | 0.137 | -3.864 | 1.00E-04 | 2.30E-03 | 60 |
| ENSG00000122512 | PMS2 | 5395 | 533.937 | -0.316 | 0.123 | -2.581 | 9.80E-03 | 7.74E-02 | 60 |
| ENSG00000186448 | ZNF197 | 10168 | 987.138 | -0.258 | 0.102 | -2.528 | 1.15E-02 | 8.60E-02 | 60 |
| ENSG00000152402 | GUCY1A2 | 2977 | 587.395 | 0.455 | 0.172 | 2.649 | 8.10E-03 | 6.69E-02 | 60 |
| ENSG00000178502 | KLHL11 | 55175 | 133.097 | -1.064 | 0.204 | -5.219 | 0.00E+00 | 0.00E+00 | 59 |
| ENSG00000145331 | TRMT10A | 93587 | 164.933 | -0.915 | 0.19 | -4.81 | 0.00E+00 | 1.00E-04 | 59 |
| ENSG00000010072 | SPRTN | 83932 | 612.883 | -0.598 | 0.133 | -4.504 | 0.00E+00 | 2.00E-04 | 59 |
| ENSG00000125484 | GTF3C4 | 9329 | 2,045.26 | -0.572 | 0.094 | -6.079 | 0.00E+00 | 0.00E+00 | 59 |
| ENSG00000196793 | ZNF239 | 8187 | 277.093 | -0.474 | 0.151 | -3.143 | 1.70E-03 | 2.04E-02 | 59 |
| ENSG00000019991 | HGF | 3082 | 3,096.93 | -0.399 | 0.092 | -4.328 | 0.00E+00 | 4.00E-04 | 59 |
| ENSG00000137502 | RAB30 | 27314 | 2,556.64 | -0.363 | 0.095 | -3.824 | 1.00E-04 | 2.60E-03 | 59 |
| ENSG00000101745 | ANKRD12 | 23253 | 2,721.22 | -0.305 | 0.106 | -2.873 | 4.10E-03 | 4.02E-02 | 59 |
| ENSG00000134077 | THUMPD3 | 25917 | 1,841.42 | -0.301 | 0.106 | -2.838 | 4.50E-03 | 4.36E-02 | 59 |
| ENSG00000157181 | ODR4 | 54953 | 1,378.26 | -0.301 | 0.113 | -2.662 | 7.80E-03 | 6.51E-02 | 59 |
| ENSG00000153214 | TMEM87B | 84910 | 3,849.82 | -0.248 | 0.1 | -2.475 | 1.33E-02 | 9.53E-02 | 59 |
| ENSG00000185008 | ROBO2 | 6092 | 2,777.79 | 0.442 | 0.137 | 3.223 | 1.30E-03 | 1.66E-02 | 59 |
| ENSG00000021826 | CPS1 | 1373 | 824.491 | -1.409 | 0.112 | -12.601 | 2.10E-36 | 6.90E-33 | 58 |
| ENSG00000136205 | TNS3 | 64759 | 6,262.54 | -0.745 | 0.103 | -7.242 | 0.00E+00 | 0.00E+00 | 58 |
| ENSG00000133026 | MYH10 | 4628 | 3,044.52 | -0.47 | 0.093 | -5.034 | 0.00E+00 | 0.00E+00 | 58 |
| ENSG00000204388 | HSPA1B | 3304 | 1,324.29 | -0.439 | 0.162 | -2.7 | 6.90E-03 | 6.02E-02 | 58 |
| ENSG00000186298 | PPP1CC | 5501 | 6,107.16 | -0.348 | 0.135 | -2.585 | 9.70E-03 | 7.70E-02 | 58 |
| ENSG00000067082 | KLF6 | 1316 | 11,310.78 | -0.254 | 0.087 | -2.916 | 3.50E-03 | 3.62E-02 | 58 |
| ENSG00000057252 | SOAT1 | 6646 | 6,144.66 | -0.248 | 0.088 | -2.833 | 4.60E-03 | 4.40E-02 | 58 |
| ENSG00000167193 | CRK | 1398 | 5,745.93 | 0.363 | 0.109 | 3.338 | 8.00E-04 | 1.20E-02 | 58 |
| ENSG00000071537 | SEL1L | 6400 | 9,667.26 | 0.488 | 0.09 | 5.398 | 0.00E+00 | 0.00E+00 | 58 |
| ENSG00000116670 | MAD2L2 | 10459 | 895.638 | -0.966 | 0.142 | -6.783 | 0.00E+00 | 0.00E+00 | 57 |
| ENSG00000181722 | ZBTB20 | 26137 | 298.411 | -0.688 | 0.241 | -2.85 | 4.40E-03 | 4.26E-02 | 57 |
| ENSG00000163694 | RBM47 | 54502 | 210.084 | -0.634 | 0.223 | -2.837 | 4.60E-03 | 4.36E-02 | 57 |
| ENSG00000074657 | ZNF532 | 55205 | 3,632.66 | -0.612 | 0.095 | -6.456 | 0.00E+00 | 0.00E+00 | 57 |
| ENSG00000164291 | ARSK | 153642 | 813.194 | -0.448 | 0.14 | -3.2 | 1.40E-03 | 1.76E-02 | 57 |
| ENSG00000101856 | PGRMC1 | 10857 | 12,624.54 | -0.329 | 0.119 | -2.772 | 5.60E-03 | 5.10E-02 | 57 |
| ENSG00000184408 | KCND2 | 3751 | 73.452 | 1.402 | 0.325 | 4.309 | 0.00E+00 | 4.00E-04 | 57 |
| ENSG00000105976 | MET | 4233 | 6,368.97 | 1.585 | 0.148 | 10.697 | 0.00E+00 | 0.00E+00 | 57 |
| ENSG00000119314 | PTBP3 | 9991 | 2,474.99 | -0.758 | 0.128 | -5.9 | 0.00E+00 | 0.00E+00 | 56 |
| ENSG00000181450 | ZNF678 | 339500 | 188.736 | -0.749 | 0.179 | -4.185 | 0.00E+00 | 7.00E-04 | 56 |
| ENSG00000138443 | ABI2 | 10152 | 2,145.57 | -0.636 | 0.127 | -5.027 | 0.00E+00 | 0.00E+00 | 56 |
| ENSG00000131507 | NDFIP1 | 80762 | 5,398.30 | -0.605 | 0.107 | -5.647 | 0.00E+00 | 0.00E+00 | 56 |
| ENSG00000102390 | PBDC1 | 51260 | 1,230.83 | -0.495 | 0.102 | -4.873 | 0.00E+00 | 0.00E+00 | 56 |
| ENSG00000105186 | ANKRD27 | 84079 | 1,572.27 | -0.479 | 0.101 | -4.741 | 0.00E+00 | 1.00E-04 | 56 |
| ENSG00000168724 | D＃NAJC21 | 134218 | 2,401.25 | -0.367 | 0.09 | -4.069 | 0.00E+00 | 1.10E-03 | 56 |
| ENSG00000105821 | D＃NAJC2 | 27000 | 1,558.27 | -0.335 | 0.098 | -3.401 | 7.00E-04 | 1.01E-02 | 56 |
| ENSG00000109046 | WSB1 | 26118 | 2,873.76 | 0.499 | 0.124 | 4.029 | 1.00E-04 | 1.30E-03 | 56 |
| ENSG00000118985 | ELL2 | 22936 | 8,781.84 | -0.918 | 0.158 | -5.81 | 0.00E+00 | 0.00E+00 | 55 |
| ENSG00000113300 | CNOT6 | 57472 | 1,434.30 | -0.831 | 0.123 | -6.735 | 0.00E+00 | 0.00E+00 | 55 |
| ENSG00000110925 | CSRNP2 | 81566 | 1,877.97 | -0.673 | 0.096 | -7.04 | 0.00E+00 | 0.00E+00 | 55 |
| ENSG00000137177 | KIF13A | 63971 | 5,591.60 | -0.607 | 0.102 | -5.946 | 0.00E+00 | 0.00E+00 | 55 |
| ENSG00000124782 | RREB1 | 6239 | 1,047.42 | -0.335 | 0.122 | -2.748 | 6.00E-03 | 5.43E-02 | 55 |
| ENSG00000137193 | PIM1 | 5292 | 386.29 | -0.956 | 0.157 | -6.078 | 0.00E+00 | 0.00E+00 | 54 |
| ENSG00000115415 | STAT1 | 6772 | 60,813.42 | -0.864 | 0.139 | -6.211 | 0.00E+00 | 0.00E+00 | 54 |
| ENSG00000118193 | KIF14 | 9928 | 1,581.55 | -0.83 | 0.127 | -6.515 | 0.00E+00 | 0.00E+00 | 54 |
| ENSG00000127980 | PEX1 | 5189 | 752.687 | -0.434 | 0.116 | -3.757 | 2.00E-04 | 3.30E-03 | 54 |
| ENSG00000121057 | AKAP1 | 8165 | 941.284 | -0.331 | 0.105 | -3.143 | 1.70E-03 | 2.04E-02 | 54 |
| ENSG00000063587 | ZNF275 | 10838 | 1,468.22 | -0.312 | 0.102 | -3.063 | 2.20E-03 | 2.51E-02 | 54 |
| ENSG00000170017 | ALCAM | 214 | 17,116.53 | -0.276 | 0.098 | -2.809 | 5.00E-03 | 4.68E-02 | 54 |
| ENSG00000137075 | RNF38 | 152006 | 1,093.21 | -0.528 | 0.129 | -4.103 | 0.00E+00 | 1.00E-03 | 53 |
| ENSG00000168502 | MTCL1 | 23255 | 2,726.82 | -0.237 | 0.093 | -2.547 | 1.09E-02 | 8.26E-02 | 53 |
| ENSG00000198807 | PAX9 | 5083 | 6,061.61 | 0.808 | 0.139 | 5.804 | 0.00E+00 | 0.00E+00 | 53 |
| ENSG00000171033 | PKIA | 5569 | 441.168 | -1.058 | 0.25 | -4.236 | 0.00E+00 | 6.00E-04 | 52 |
| ENSG00000176542 | USF3 | 205717 | 699.582 | -0.541 | 0.124 | -4.377 | 0.00E+00 | 3.00E-04 | 52 |
| ENSG00000103196 | CRISPLD2 | 83716 | 1,642.30 | -0.42 | 0.107 | -3.925 | 1.00E-04 | 1.80E-03 | 52 |
| ENSG00000160551 | TAOK1 | 57551 | 6,085.40 | -0.256 | 0.101 | -2.527 | 1.15E-02 | 8.60E-02 | 52 |
| ENSG00000144674 | GOLGA4 | 2803 | 10,768.00 | -0.25 | 0.081 | -3.102 | 1.90E-03 | 2.27E-02 | 52 |
| ENSG00000127022 | CANX | 821 | 61,257.55 | -0.226 | 0.084 | -2.7 | 6.90E-03 | 6.02E-02 | 52 |
| ENSG00000107643 | MAPK8 | 5599 | 931.31 | -1.008 | 0.15 | -6.729 | 0.00E+00 | 0.00E+00 | 51 |
| ENSG00000143190 | POU2F1 | 5451 | 307.024 | -0.979 | 0.156 | -6.271 | 0.00E+00 | 0.00E+00 | 51 |
| ENSG00000198399 | ITSN2 | 50618 | 1,744.92 | -0.883 | 0.1 | -8.863 | 0.00E+00 | 0.00E+00 | 51 |
| ENSG00000160352 | ZNF714 | 148206 | 382.497 | -0.678 | 0.143 | -4.74 | 0.00E+00 | 1.00E-04 | 51 |
| ENSG00000174839 | DENND6A | 201627 | 475.902 | -0.464 | 0.153 | -3.039 | 2.40E-03 | 2.68E-02 | 51 |
| ENSG00000111450 | STX2 | 2054 | 2,096.18 | -0.444 | 0.103 | -4.32 | 0.00E+00 | 4.00E-04 | 51 |
| ENSG00000156273 | BACH1 | 571 | 2,221.89 | -0.431 | 0.101 | -4.266 | 0.00E+00 | 5.00E-04 | 51 |
| ENSG00000179151 | EDC3 | 80153 | 1,533.34 | -0.374 | 0.12 | -3.125 | 1.80E-03 | 2.14E-02 | 51 |
| ENSG00000187605 | TET3 | 200424 | 577.522 | -0.372 | 0.139 | -2.666 | 7.70E-03 | 6.46E-02 | 51 |
| ENSG00000145555 | MYO10 | 4651 | 4,912.81 | -0.312 | 0.106 | -2.935 | 3.30E-03 | 3.45E-02 | 51 |
| ENSG00000171490 | RSL1D1 | 26156 | 6,641.10 | -0.219 | 0.081 | -2.702 | 6.90E-03 | 6.00E-02 | 51 |
| ENSG00000128872 | TMOD2 | 29767 | 1,341.23 | -0.512 | 0.137 | -3.721 | 2.00E-04 | 3.70E-03 | 50 |
| ENSG00000169032 | MAP2K1 | 5604 | 2,153.94 | -0.437 | 0.11 | -3.952 | 1.00E-04 | 1.70E-03 | 50 |
| ENSG00000124177 | CHD6 | 84181 | 1,643.61 | -0.431 | 0.125 | -3.464 | 5.00E-04 | 8.40E-03 | 50 |
| ENSG00000164951 | PDP1 | 54704 | 1,682.18 | -0.389 | 0.131 | -2.961 | 3.10E-03 | 3.24E-02 | 50 |
| ENSG00000137073 | UBAP2 | 55833 | 2,286.26 | -0.339 | 0.124 | -2.723 | 6.50E-03 | 5.73E-02 | 50 |
| ENSG00000136381 | IREB2 | 3658 | 2,639.00 | -0.247 | 0.091 | -2.718 | 6.60E-03 | 5.80E-02 | 50 |

| Supplementary Table 5. DEGs of miR-144-5P with target score | | | | | | | | | |
| --- | --- | --- | --- | --- | --- | --- | --- | --- | --- |
| **gene ID** | **symbol** | **entrez** | **baseMean** | **log2Fold Change** | **lfcSE** | **stat** | **pvalue** | **padj** | **Target Score** |
| ENSG00000108960 | MMD | 23531 | 578.304 | -2.162 | 0.207 | -10.429 | 1.80E-25 | 3.70E-23 | 86 |
| ENSG00000196937 | FAM3C | 10447 | 3,227.57 | -1.969 | 0.134 | -14.669 | 1.00E-48 | 1.50E-45 | 81 |
| ENSG00000116954 | RRAGC | 64121 | 2,498.21 | -1.706 | 0.119 | -14.361 | 9.10E-47 | 1.20E-43 | 92 |
| ENSG00000155097 | ATP6V1C1 | 528 | 4,327.88 | -1.629 | 0.125 | -12.996 | 1.30E-38 | 8.10E-36 | 95 |
| ENSG00000166313 | APBB1 | 322 | 1,531.70 | -1.371 | 0.111 | -12.349 | 4.90E-35 | 2.40E-32 | 91 |
| ENSG00000134594 | RAB33A | 9363 | 62.974 | -1.367 | 0.319 | -4.283 | 1.80E-05 | 2.80E-04 | 94 |
| ENSG00000114354 | TFG | 10342 | 6,489.57 | -1.359 | 0.096 | -14.188 | 1.10E-45 | 1.20E-42 | 93 |
| ENSG00000156171 | DRAM2 | 128338 | 684.098 | -1.35 | 0.142 | -9.492 | 2.30E-21 | 3.10E-19 | 83 |
| ENSG00000165476 | REEP3 | 221035 | 3,326.46 | -1.334 | 0.117 | -11.452 | 2.30E-30 | 7.40E-28 | 83 |
| ENSG00000235109 | ZSCAN31 | 64288 | 168.895 | -1.292 | 0.201 | -6.439 | 1.20E-10 | 4.70E-09 | 84 |
| ENSG00000159082 | SYNJ1 | 8867 | 974.775 | -1.232 | 0.141 | -8.76 | 1.90E-18 | 2.00E-16 | 88 |
| ENSG00000197601 | FAR1 | 84188 | 2,560.37 | -1.181 | 0.112 | -10.566 | 0.00E+00 | 0.00E+00 | 80 |
| ENSG00000168172 | HOOK3 | 84376 | 2,446.30 | -1.14 | 0.119 | -9.62 | 0.00E+00 | 0.00E+00 | 82 |
| ENSG00000157224 | CLDN12 | 9069 | 1,348.51 | -1.066 | 0.126 | -8.446 | 0.00E+00 | 0.00E+00 | 81 |
| ENSG00000155744 | FAM126B | 285172 | 488.336 | -1.063 | 0.132 | -8.069 | 0.00E+00 | 0.00E+00 | 81 |
| ENSG00000206538 | VGLL3 | 389136 | 5,775.68 | -1.02 | 0.09 | -11.352 | 7.30E-30 | 2.20E-27 | 86 |
| ENSG00000073712 | FERMT2 | 10979 | 6,211.39 | -0.973 | 0.107 | -9.133 | 0.00E+00 | 0.00E+00 | 83 |
| ENSG00000108175 | ZMIZ1 | 57178 | 4,009.89 | -0.949 | 0.133 | -7.113 | 1.10E-12 | 5.80E-11 | 93 |
| ENSG00000117280 | RAB29 | 8934 | 2,898.01 | -0.882 | 0.108 | -8.2 | 0.00E+00 | 0.00E+00 | 83 |
| ENSG00000137393 | RNF144B | 255488 | 384.608 | -0.868 | 0.135 | -6.409 | 0.00E+00 | 0.00E+00 | 84 |
| ENSG00000127483 | HP1BP3 | 50809 | 8,420.41 | -0.835 | 0.091 | -9.2 | 0.00E+00 | 0.00E+00 | 88 |
| ENSG00000149054 | ZNF215 | 7762 | 167.676 | -0.788 | 0.213 | -3.706 | 2.00E-04 | 2.20E-03 | 89 |
| ENSG00000114933 | INO80D | 54891 | 482.694 | -0.75 | 0.133 | -5.642 | 0.00E+00 | 0.00E+00 | 88 |
| ENSG00000048828 | FAM120A | 23196 | 8,676.60 | -0.679 | 0.094 | -7.195 | 0.00E+00 | 0.00E+00 | 85 |
| ENSG00000177374 | HIC1 | 3090 | 682.151 | -0.659 | 0.123 | -5.348 | 5.60E-14 | 3.50E-12 | 94 |
| ENSG00000125945 | ZNF436 | 80818 | 545.72 | -0.609 | 0.129 | -4.725 | 2.30E-06 | 4.20E-05 | 90 |
| ENSG00000148835 | TAF5 | 6877 | 356.496 | -0.602 | 0.155 | -3.876 | 1.00E-04 | 1.20E-03 | 94 |
| ENSG00000157450 | RNF111 | 54778 | 1,548.80 | -0.597 | 0.106 | -5.61 | 0.00E+00 | 0.00E+00 | 86 |
| ENSG00000138709 | LARP1B | 55132 | 782.718 | -0.596 | 0.119 | -5.023 | 0.00E+00 | 0.00E+00 | 86 |
| ENSG00000142794 | NBPF3 | 84224 | 800.874 | -0.513 | 0.134 | -3.835 | 1.00E-04 | 1.40E-03 | 94 |
| ENSG00000080503 | SMARCA2 | 6595 | 3,696.48 | -0.501 | 0.1 | -5.01 | 5.40E-07 | 1.10E-05 | 93 |
| ENSG00000130449 | ZSWIM6 | 57688 | 1,373.75 | -0.458 | 0.101 | -4.522 | 0.00E+00 | 1.00E-04 | 85 |
| ENSG00000081386 | ZNF510 | 22869 | 470.027 | -0.404 | 0.131 | -3.076 | 2.10E-03 | 1.51E-02 | 82 |
| ENSG00000120948 | TARDBP | 23435 | 3,352.33 | -0.379 | 0.119 | -3.171 | 1.50E-03 | 1.16E-02 | 86 |
| ENSG00000111647 | UHRF1BP1L | 23074 | 2,381.94 | -0.369 | 0.095 | -3.907 | 1.00E-04 | 1.10E-03 | 86 |
| ENSG00000110344 | UBE4A | 9354 | 2,158.86 | -1.434 | 0.124 | -11.565 | 6.20E-31 | 2.30E-28 | 79 |
| ENSG00000145780 | FEM1C | 56929 | 1,963.72 | -0.882 | 0.119 | -7.392 | 0.00E+00 | 0.00E+00 | 79 |
| ENSG00000162819 | BROX | 148362 | 2,298.62 | -0.544 | 0.115 | -4.734 | 0.00E+00 | 0.00E+00 | 79 |
| ENSG00000188641 | DPYD | 1806 | 2,430.76 | 0.296 | 0.107 | 2.775 | 5.50E-03 | 3.33E-02 | 79 |
| ENSG00000169490 | TM2D2 | 83877 | 2,316.98 | -1.723 | 0.101 | -16.984 | 1.10E-64 | 7.10E-61 | 78 |
| ENSG00000164220 | F2RL2 | 2151 | 4,346.32 | -1.501 | 0.1 | -14.964 | 1.30E-50 | 2.10E-47 | 78 |
| ENSG00000196782 | MAML3 | 55534 | 155.392 | -1.023 | 0.234 | -4.371 | 0.00E+00 | 2.00E-04 | 78 |
| ENSG00000119616 | FCF1 | 51077 | 2,147.38 | -1.229 | 0.107 | -11.489 | 0.00E+00 | 0.00E+00 | 77 |
| ENSG00000017260 | ATP2C1 | 27032 | 6,951.28 | -0.814 | 0.081 | -10.019 | 0.00E+00 | 0.00E+00 | 77 |
| ENSG00000106483 | SFRP4 | 6424 | 795.372 | -0.58 | 0.2 | -2.897 | 3.80E-03 | 2.47E-02 | 77 |
| ENSG00000121542 | SEC22A | 26984 | 630.479 | -0.281 | 0.117 | -2.403 | 1.63E-02 | 7.61E-02 | 77 |
| ENSG00000001629 | ANKIB1 | 54467 | 3,977.27 | -1.295 | 0.141 | -9.205 | 3.40E-20 | 4.10E-18 | 76 |
| ENSG00000047410 | TPR | 7175 | 7,569.00 | -0.211 | 0.092 | -2.296 | 2.17E-02 | 9.41E-02 | 76 |
| ENSG00000175322 | ZNF519 | 162655 | 108.637 | -0.621 | 0.239 | -2.594 | 9.50E-03 | 5.05E-02 | 75 |
| ENSG00000143776 | CDC42BPA | 8476 | 4,629.43 | -0.609 | 0.121 | -5.048 | 0.00E+00 | 0.00E+00 | 75 |
| ENSG00000132849 | PATJ | 10207 | 347.85 | -0.386 | 0.145 | -2.664 | 7.70E-03 | 4.34E-02 | 75 |
| ENSG00000197063 | MAFG | 4097 | 3,058.74 | -0.739 | 0.11 | -6.747 | 0.00E+00 | 0.00E+00 | 74 |
| ENSG00000111224 | PARP11 | 57097 | 357.699 | -1.832 | 0.158 | -11.624 | 3.10E-31 | 1.20E-28 | 73 |
| ENSG00000165389 | SPTSSA | 171546 | 740.192 | -1.724 | 0.172 | -10.04 | 1.00E-23 | 1.70E-21 | 73 |
| ENSG00000166913 | YWHAB | 7529 | 20,265.79 | -0.291 | 0.079 | -3.683 | 2.00E-04 | 2.40E-03 | 73 |
| ENSG00000133121 | STARD13 | 90627 | 2,136.57 | -0.227 | 0.091 | -2.505 | 1.23E-02 | 6.16E-02 | 73 |
| ENSG00000165572 | KBTBD6 | 89890 | 389.376 | -1.595 | 0.145 | -11.012 | 3.30E-28 | 9.30E-26 | 72 |
| ENSG00000136937 | NCBP1 | 4686 | 3,215.94 | -0.817 | 0.092 | -8.93 | 0.00E+00 | 0.00E+00 | 72 |
| ENSG00000132670 | PTPRA | 5786 | 3,883.38 | -0.679 | 0.106 | -6.437 | 0.00E+00 | 0.00E+00 | 72 |
| ENSG00000196233 | LCOR | 84458 | 1,235.59 | -0.389 | 0.122 | -3.185 | 1.40E-03 | 1.11E-02 | 72 |
| ENSG00000170365 | SMAD1 | 4086 | 393.317 | -1.078 | 0.139 | -7.772 | 0.00E+00 | 0.00E+00 | 71 |
| ENSG00000072657 | TRHDE | 29953 | 659.13 | -0.9 | 0.195 | -4.625 | 0.00E+00 | 1.00E-04 | 71 |
| ENSG00000196757 | ZNF700 | 90592 | 315.816 | -0.875 | 0.153 | -5.727 | 0.00E+00 | 0.00E+00 | 71 |
| ENSG00000117593 | DARS2 | 55157 | 1,453.76 | -0.786 | 0.113 | -6.951 | 0.00E+00 | 0.00E+00 | 71 |
| ENSG00000145476 | CYP4V2 | 285440 | 1,327.64 | -0.778 | 0.114 | -6.814 | 0.00E+00 | 0.00E+00 | 71 |
| ENSG00000118655 | DCLRE1B | 64858 | 658.619 | -0.497 | 0.136 | -3.651 | 3.00E-04 | 2.70E-03 | 71 |
| ENSG00000143633 | C1orf131 | 128061 | 324.119 | -1.204 | 0.144 | -8.375 | 0.00E+00 | 0.00E+00 | 70 |
| ENSG00000188868 | ZNF563 | 147837 | 123.313 | -1.078 | 0.23 | -4.683 | 0.00E+00 | 1.00E-04 | 70 |
| ENSG00000005194 | CIAPIN1 | 57019 | 2,005.16 | -0.692 | 0.095 | -7.318 | 0.00E+00 | 0.00E+00 | 70 |
| ENSG00000085231 | AK6 | 102157402 | 842.01 | -1.688 | 0.153 | -11.006 | 3.60E-28 | 9.60E-26 | 69 |
| ENSG00000085719 | CPNE3 | 8895 | 5,120.69 | -1.097 | 0.096 | -11.465 | 0.00E+00 | 0.00E+00 | 69 |
| ENSG00000104415 | CCN4 | 8840 | 222.439 | -0.909 | 0.261 | -3.486 | 5.00E-04 | 4.50E-03 | 69 |
| ENSG00000115966 | ATF2 | 1386 | 1,526.65 | -0.811 | 0.099 | -8.226 | 0.00E+00 | 0.00E+00 | 69 |
| ENSG00000138756 | BMP2K | 55589 | 1,506.17 | -0.47 | 0.121 | -3.904 | 1.00E-04 | 1.10E-03 | 69 |
| ENSG00000173674 | EIF1AX | 1964 | 4,354.60 | -1.239 | 0.136 | -9.089 | 0.00E+00 | 0.00E+00 | 68 |
| ENSG00000176834 | VSIG10 | 54621 | 864.034 | -0.456 | 0.111 | -4.106 | 0.00E+00 | 6.00E-04 | 67 |
| ENSG00000114098 | ARMC8 | 25852 | 1,819.99 | -0.369 | 0.096 | -3.846 | 1.00E-04 | 1.40E-03 | 67 |
| ENSG00000198826 | ARHGAP11A | 9824 | 5,268.34 | -0.595 | 0.132 | -4.492 | 0.00E+00 | 1.00E-04 | 66 |
| ENSG00000097033 | SH3GLB1 | 51100 | 7,600.60 | -0.264 | 0.083 | -3.195 | 1.40E-03 | 1.08E-02 | 66 |
| ENSG00000205268 | PDE7A | 5150 | 441.342 | -0.964 | 0.148 | -6.503 | 0.00E+00 | 0.00E+00 | 65 |
| ENSG00000141380 | SS18 | 6760 | 3,090.80 | -1.012 | 0.119 | -8.511 | 0.00E+00 | 0.00E+00 | 64 |
| ENSG00000164040 | PGRMC2 | 10424 | 6,476.50 | -0.832 | 0.137 | -6.057 | 0.00E+00 | 0.00E+00 | 64 |
| ENSG00000095574 | IKZF5 | 64376 | 452.937 | -0.602 | 0.127 | -4.735 | 0.00E+00 | 0.00E+00 | 64 |
| ENSG00000104517 | UBR5 | 51366 | 5,763.54 | -0.479 | 0.141 | -3.407 | 7.00E-04 | 5.80E-03 | 64 |
| ENSG00000163577 | EIF5A2 | 56648 | 941.307 | -1.922 | 0.149 | -12.866 | 7.00E-38 | 4.20E-35 | 63 |
| ENSG00000105887 | MTPN | 136319 | 11,191.94 | -1.126 | 0.117 | -9.647 | 0.00E+00 | 0.00E+00 | 63 |
| ENSG00000100523 | DDHD1 | 80821 | 1,305.42 | -0.357 | 0.096 | -3.712 | 2.00E-04 | 2.20E-03 | 63 |
| ENSG00000170035 | UBE2E3 | 10477 | 3,200.13 | -1.753 | 0.163 | -10.778 | 4.40E-27 | 9.80E-25 | 62 |
| ENSG00000172403 | SYNPO2 | 171024 | 4,892.57 | -1.157 | 0.125 | -9.268 | 0.00E+00 | 0.00E+00 | 62 |
| ENSG00000036549 | ZZZ3 | 26009 | 2,216.24 | -0.845 | 0.101 | -8.386 | 0.00E+00 | 0.00E+00 | 62 |
| ENSG00000197937 | ZNF347 | 84671 | 617.072 | -0.448 | 0.134 | -3.341 | 8.00E-04 | 7.10E-03 | 62 |
| ENSG00000078043 | PIAS2 | 9063 | 1,009.84 | -0.429 | 0.105 | -4.092 | 0.00E+00 | 6.00E-04 | 62 |
| ENSG00000174437 | ATP2A2 | 488 | 18,015.15 | -1.318 | 0.094 | -14.086 | 4.60E-45 | 4.70E-42 | 61 |
| ENSG00000072422 | RHOBTB1 | 9886 | 163.842 | -1.147 | 0.212 | -5.412 | 0.00E+00 | 0.00E+00 | 61 |
| ENSG00000164741 | DLC1 | 10395 | 7,104.92 | -0.997 | 0.117 | -8.525 | 0.00E+00 | 0.00E+00 | 61 |
| ENSG00000128923 | MINDY2 | 54629 | 2,142.37 | -0.935 | 0.137 | -6.83 | 0.00E+00 | 0.00E+00 | 61 |
| ENSG00000135686 | KLHL36 | 79786 | 1,613.94 | -0.489 | 0.107 | -4.569 | 0.00E+00 | 1.00E-04 | 61 |
| ENSG00000218739 | CEBPZOS | 100505876 | 1,513.67 | -0.811 | 0.142 | -5.691 | 0.00E+00 | 0.00E+00 | 60 |
| ENSG00000139946 | PELI2 | 57161 | 526.27 | -0.692 | 0.129 | -5.357 | 0.00E+00 | 0.00E+00 | 60 |
| ENSG00000072415 | MPP5 | 64398 | 2,390.42 | -0.819 | 0.127 | -6.431 | 0.00E+00 | 0.00E+00 | 59 |
| ENSG00000117543 | DPH5 | 51611 | 994.674 | -0.306 | 0.108 | -2.848 | 4.40E-03 | 2.78E-02 | 59 |
| ENSG00000135838 | NPL | 80896 | 95.342 | -1.259 | 0.239 | -5.277 | 0.00E+00 | 0.00E+00 | 58 |
| ENSG00000146676 | PURB | 5814 | 2,063.65 | -1.106 | 0.143 | -7.75 | 0.00E+00 | 0.00E+00 | 58 |
| ENSG00000139793 | MBNL2 | 10150 | 1,705.41 | -0.85 | 0.117 | -7.246 | 0.00E+00 | 0.00E+00 | 58 |
| ENSG00000197535 | MYO5A | 4644 | 6,086.33 | -0.596 | 0.12 | -4.962 | 0.00E+00 | 0.00E+00 | 58 |
| ENSG00000187605 | TET3 | 200424 | 577.522 | -0.539 | 0.141 | -3.833 | 1.00E-04 | 1.40E-03 | 58 |
| ENSG00000154642 | C21orf91 | 54149 | 972.625 | -0.514 | 0.135 | -3.804 | 1.00E-04 | 1.60E-03 | 58 |
| ENSG00000116903 | EXOC8 | 149371 | 858.255 | -0.415 | 0.108 | -3.854 | 1.00E-04 | 1.30E-03 | 58 |
| ENSG00000104043 | ATP8B4 | 79895 | 111.807 | -0.775 | 0.283 | -2.735 | 6.20E-03 | 3.68E-02 | 57 |
| ENSG00000143390 | RFX5 | 5993 | 1,698.09 | -0.769 | 0.109 | -7.042 | 0.00E+00 | 0.00E+00 | 57 |
| ENSG00000163638 | ADAMTS9 | 56999 | 114.866 | -0.804 | 0.302 | -2.665 | 7.70E-03 | 4.33E-02 | 56 |
| ENSG00000172667 | ZMAT3 | 64393 | 5,244.26 | -0.526 | 0.109 | -4.812 | 0.00E+00 | 0.00E+00 | 56 |
| ENSG00000136026 | CKAP4 | 10970 | 35,313.84 | -0.957 | 0.13 | -7.368 | 0.00E+00 | 0.00E+00 | 55 |
| ENSG00000181163 | NPM1 | 4869 | 39,963.99 | -0.704 | 0.085 | -8.282 | 0.00E+00 | 0.00E+00 | 55 |
| ENSG00000204406 | MBD5 | 55777 | 901.975 | -0.389 | 0.146 | -2.663 | 7.80E-03 | 4.35E-02 | 55 |
| ENSG00000153814 | JAZF1 | 221895 | 822.804 | -0.357 | 0.148 | -2.412 | 1.59E-02 | 7.46E-02 | 55 |
| ENSG00000115226 | FNDC4 | 64838 | 675.177 | -1.826 | 0.131 | -13.984 | 2.00E-44 | 1.80E-41 | 54 |
| ENSG00000163923 | RPL39L | 116832 | 500.602 | -1.721 | 0.151 | -11.38 | 5.30E-30 | 1.60E-27 | 54 |
| ENSG00000154813 | DPH3 | 285381 | 1,928.14 | -1.208 | 0.105 | -11.519 | 0.00E+00 | 0.00E+00 | 54 |
| ENSG00000124532 | MRS2 | 57380 | 1,237.45 | -0.424 | 0.106 | -3.984 | 1.00E-04 | 9.00E-04 | 54 |
| ENSG00000129315 | CCNT1 | 904 | 1,513.37 | -0.789 | 0.109 | -7.264 | 0.00E+00 | 0.00E+00 | 53 |
| ENSG00000146410 | MTFR2 | 113115 | 434.11 | -0.552 | 0.174 | -3.178 | 1.50E-03 | 1.14E-02 | 53 |
| ENSG00000071539 | TRIP13 | 9319 | 2,093.31 | -1.81 | 0.138 | -13.147 | 1.80E-39 | 1.20E-36 | 52 |
| ENSG00000092847 | AGO1 | 26523 | 1,501.53 | -1.178 | 0.137 | -8.6 | 0.00E+00 | 0.00E+00 | 52 |
| ENSG00000091656 | ZFHX4 | 79776 | 1,066.46 | -1.133 | 0.167 | -6.765 | 0.00E+00 | 0.00E+00 | 52 |
| ENSG00000140961 | OSGIN1 | 29948 | 2,416.03 | -0.697 | 0.139 | -5.023 | 0.00E+00 | 0.00E+00 | 52 |
| ENSG00000110328 | GALNT18 | 374378 | 252.445 | -0.806 | 0.192 | -4.208 | 0.00E+00 | 4.00E-04 | 50 |
| ENSG00000165355 | FBXO33 | 254170 | 1,004.25 | -0.779 | 0.131 | -5.967 | 0.00E+00 | 0.00E+00 | 50 |
| ENSG00000116750 | UCHL5 | 51377 | 1,377.05 | -0.451 | 0.125 | -3.613 | 3.00E-04 | 3.00E-03 | 50 |

| Supplementary Table 6. DEGs of miR-30e-5P with target score | | | | | | | | | |
| --- | --- | --- | --- | --- | --- | --- | --- | --- | --- |
|  | symbol | entrez | baseMean | log2Fold Change | lfcSE | stat | pvalue | padj | Target sore |
| ENSG00000138413 | IDH1 | 3417 | 3,270.001 | -2,201 | 0.171 | -12,905 | 4.20E-38 | 9.00E-35 | 90 |
| ENSG00000180776 | ZDHHC20 | 253832 | 1,441.699 | -2,113 | 0.183 | -11,549 | 7.50E-31 | 7.40E-28 | 61 |
| ENSG00000100934 | SEC23A | 10484 | 4,717.726 | -1,821 | 0.144 | -12,665 | 9.20E-37 | 1.50E-33 | 95 |
| ENSG00000128606 | LRRC17 | 10234 | 158,678 | -1,742 | 0.273 | -6,379 | 1.80E-10 | 1.70E-08 | 99 |
| ENSG00000172819 | RARG | 5916 | 4,902.402 | -1,733 | 0.125 | -13,806 | 2.40E-43 | 1.50E-39 | 99 |
| ENSG00000109586 | GALNT7 | 51809 | 1,113.339 | -1,705 | 0.158 | -10,759 | 5.40E-27 | 3.90E-24 | 98 |
| ENSG00000135074 | ADAM19 | 8728 | 665,985 | -1,679 | 0.130 | -12,932 | 3.00E-38 | 7.70E-35 | 96 |
| ENSG00000184524 | CEND1 | 51286 | 99,461 | -1,538 | 0.280 | -5,501 | 3.80E-08 | 2.3E-06 | NA |
| ENSG00000099260 | PALMD | 54873 | 210,018 | -1,475 | 0.252 | -5,859 | 4.70E-09 | 3.40E-07 | NA |
| ENSG00000169857 | AVEN | 57099 | 715,398 | -1,469 | 0.152 | -9,671 | 4.00E-22 | 2.30E-19 | 81 |
| ENSG00000178202 | POGLUT3 | 143888 | 3,368.231 | -1,448 | 0.139 | -10,403 | 2.40E-25 | 1.60E-22 | NA |
| ENSG00000145604 | SKP2 | 6502 | 660,091 | -1,446 | 0.133 | -10,880 | 1.40E-27 | 1.10E-24 | 91 |
| ENSG00000047634 | SCML1 | 6322 | 866,405 | -1,444 | 0.128 | -11,289 | 1.50E-29 | 1.30E-26 | 98 |
| ENSG00000146433 | TMEM181 | 57583 | 2,280.495 | -1,421 | 0.104 | -13,719 | 7.80E-43 | 3.40E-39 | 98 |
| ENSG00000254300 | LINC01111 | 1E+08 | 109,075 | -1,415 | 0.293 | -4,831 | 1.4E-06 | 0.000056 | NA |
| ENSG00000135318 | NT5E | 4907 | 37,696.324 | -1,394 | 0.102 | -13,669 | 1.60E-42 | 5.00E-39 | 99 |
| ENSG00000105866 | SP4 | 6671 | 61,991 | -1,355 | 0.331 | -4,090 | 4.3E-05 | 0.0012 | 83 |
| ENSG00000074047 | GLI2 | 2736 | 174,305 | -1,350 | 0.268 | -5,037 | 4.70E-07 | 0.000022 | 88 |
| ENSG00000166592 | RRAD | 6236 | 1,005.416 | -1,332 | 0.182 | -7,313 | 2.60E-13 | 4.20E-11 | 95 |
| ENSG00000114353 | GNAI2 | 2771 | 18,826.036 | -1,324 | 0.116 | -11,403 | 4.00E-30 | 3.70E-27 | 90 |
| ENSG00000102524 | TNFSF13B | 10673 | 397,765 | -1,312 | 0.220 | -5,953 | 2.60E-09 | 2.10E-07 | NA |
| ENSG00000181830 | SLC35C1 | 55343 | 2,541.671 | -1,297 | 0.106 | -12,195 | 3.30E-34 | 4.80E-31 | 96 |
| ENSG00000101057 | MYBL2 | 4605 | 1,434.556 | -1,295 | 0.148 | -8,764 | 1.90E-18 | 7.10E-16 | 93 |
| ENSG00000242265 | PEG10 | 23089 | 2,367.302 | -1,248 | 0.162 | -7,711 | 0.0000 | 0.0000 | 78 |
| ENSG00000124875 | CXCL6 | 6372 | 99,968 | -1,231 | 0.272 | -4,527 | 0.0000 | 0.0002 | NA |
| ENSG00000168916 | ZNF608 | 57507 | 346,960 | -1,213 | 0.449 | -2,701 | 0.0069 | 0.0712 | 94 |
| ENSG00000100628 | ASB2 | 51676 | 184,637 | -1,204 | 0.259 | -4,653 | 0.0000 | 0.0001 | 83 |
| ENSG00000139636 | LMBR1L | 55716 | 715,871 | -1,192 | 0.138 | -8,619 | 0.0000 | 0.0000 | 98 |
| ENSG00000164331 | ANKRA2 | 57763 | 306,288 | -1,178 | 0.145 | -8,118 | 0.0000 | 0.0000 | 100 |
| ENSG00000162496 | DHRS3 | 9249 | 1,098.594 | -1,177 | 0.145 | -8,127 | 0.0000 | 0.0000 | NA |
| ENSG00000131459 | GFPT2 | 9945 | 219,889 | -1,169 | 0.176 | -6,639 | 0.0000 | 0.0000 | 90 |
| ENSG00000100605 | ITPK1 | 3705 | 1,385.009 | -1,165 | 0.136 | -8,583 | 0.0000 | 0.0000 | 97 |
| ENSG00000072682 | P4HA2 | 8974 | 8,820.915 | -1,162 | 0.121 | -9,573 | 0.0000 | 0.0000 | 94 |
| ENSG00000108551 | RASD1 | 51655 | 114,721 | -1,159 | 0.256 | -4,533 | 0.0000 | 0.0002 | 90 |
| ENSG00000134247 | PTGFRN | 5738 | 1,006.274 | -1,157 | 0.147 | -7,893 | 0.0000 | 0.0000 | 100 |
| ENSG00000138311 | ZNF365 | 22891 | 54,629 | -1,153 | 0.314 | -3,675 | 0.0002 | 0.0049 | NA |
| ENSG00000121644 | DESI2 | 51029 | 1,526.351 | -1,153 | 0.124 | -9,288 | 0.0000 | 0.0000 | 98 |
| ENSG00000121578 | B4GALT4 | 8702 | 1,797.284 | -1,140 | 0.095 | -11,943 | 0.0000 | 0.0000 | 50 |
| ENSG00000151239 | TWF1 | 5756 | 2,748.727 | -1,127 | 0.161 | -6,976 | 0.0000 | 0.0000 | 100 |
| ENSG00000186187 | ZNRF1 | 84937 | 499,791 | -1,122 | 0.137 | -8,212 | 0.0000 | 0.0000 | 98 |
| ENSG00000146112 | PPP1R18 | 170954 | 8,230.750 | -1,107 | 0.087 | -12,695 | 0.0000 | 0.0000 | 86 |
| ENSG00000067955 | CBFB | 865 | 1,110.557 | -1,099 | 0.101 | -10,932 | 0.0000 | 0.0000 | 83 |
| ENSG00000188215 | DCUN1D3 | 123879 | 696,579 | -1,090 | 0.122 | -8,921 | 0.0000 | 0.0000 | 100 |
| ENSG00000144339 | TMEFF2 | 23671 | 69,174 | -1,073 | 0.391 | -2,741 | 0.0061 | 0.0649 | NA |
| ENSG00000141429 | GALNT1 | 2589 | 1,842.088 | -1,063 | 0.129 | -8,261 | 0.0000 | 0.0000 | 91 |
| ENSG00000026025 | VIM | 7431 | 125,472.201 | -1,057 | 0.116 | -9,115 | 0.0000 | 0.0000 | 97 |
| ENSG00000172954 | LCLAT1 | 253558 | 398,322 | -1,054 | 0.173 | -6,106 | 0.0000 | 0.0000 | 99 |
| ENSG00000171848 | RRM2 | 6241 | 6,342.611 | -1,050 | 0.148 | -7,084 | 0.0000 | 0.0000 | NA |
| ENSG00000269958 |  |  | 288,738 | -1,041 | 0.153 | -6,822 | 0.0000 | 0.0000 | NA |
| ENSG00000174013 | FBXO45 | 200933 | 438,830 | -1,035 | 0.169 | -6,130 | 0.0000 | 0.0000 | 96 |
| ENSG00000196743 | GM2A | 2760 | 4,032.169 | -1,033 | 0.121 | -8,531 | 0.0000 | 0.0000 | 66 |
| ENSG00000258900 |  |  | 45,889 | -1,031 | 0.311 | -3,319 | 0.0009 | 0.0145 | NA |
| ENSG00000197872 | CYRIA | 81553 | 60,628 | -1,019 | 0.310 | -3,283 | 0.0010 | 0.0160 | NA |
| ENSG00000102098 | SCML2 | 10389 | 230,400 | -1,014 | 0.210 | -4,831 | 0.0000 | 0.0001 | 69 |
| ENSG00000028839 | TBPL1 | 9519 | 479,356 | -1,010 | 0.155 | -6,533 | 0.0000 | 0.0000 | 53 |
| ENSG00000154040 | CABYR | 26256 | 121,018 | -0.991 | 0.232 | -4,281 | 0.0000 | 0.0006 | NA |
| ENSG00000172137 | CALB2 | 794 | 319,222 | -0.990 | 0.244 | -4,063 | 0.0000 | 0.0013 | 63 |
| ENSG00000006576 | PHTF2 | 57157 | 952,922 | -0.984 | 0.167 | -5,888 | 0.0000 | 0.0000 | 97 |
| ENSG00000099953 | MMP11 | 4320 | 46,258 | -0.982 | 0.355 | -2,766 | 0.0057 | 0.0610 | NA |
| ENSG00000151208 | DLG5 | 9231 | 2,535.452 | -0.980 | 0.137 | -7,154 | 0.0000 | 0.0000 | 95 |
| ENSG00000145782 | ATG12 | 9140 | 1,572.087 | -0.974 | 0.115 | -8,490 | 0.0000 | 0.0000 | 97 |
| ENSG00000124574 | ABCC10 | 89845 | 1,366.686 | -0.972 | 0.130 | -7,495 | 0.0000 | 0.0000 | NA |
| ENSG00000128872 | TMOD2 | 29767 | 606,689 | -0.972 | 0.138 | -7,022 | 0.0000 | 0.0000 | 94 |
| ENSG00000131389 | SLC6A6 | 6533 | 3,603.266 | -0.971 | 0.112 | -8,680 | 0.0000 | 0.0000 | 81 |
| ENSG00000177879 | AP3S1 | 1176 | 2,515.689 | -0.967 | 0.119 | -8,098 | 0.0000 | 0.0000 | 78 |
| ENSG00000154359 | LONRF1 | 91694 | 279,601 | -0.959 | 0.163 | -5,888 | 0.0000 | 0.0000 | 69 |
| ENSG00000116962 | NID1 | 4811 | 4,214.208 | -0.959 | 0.092 | -10,412 | 0.0000 | 0.0000 | 65 |
| ENSG00000117600 | PLPPR4 | 9890 | 194,167 | -0.956 | 0.218 | -4,382 | 0.0000 | 0.0004 | 98 |
| ENSG00000240344 | PPIL3 | 53938 | 407,525 | -0.955 | 0.144 | -6,628 | 0.0000 | 0.0000 | 67 |
| ENSG00000167550 | RHEBL1 | 121268 | 79,433 | -0.954 | 0.280 | -3,413 | 0.0006 | 0.0109 | 90 |
| ENSG00000140044 | JDP2 | 122953 | 889,804 | -0.952 | 0.149 | -6,378 | 0.0000 | 0.0000 | 88 |
| ENSG00000163939 | PBRM1 | 55193 | 2,013.086 | -0.952 | 0.128 | -7,458 | 0.0000 | 0.0000 | 65 |
| ENSG00000076321 | KLHL20 | 27252 | 911,315 | -0.951 | 0.111 | -8,547 | 0.0000 | 0.0000 | 100 |
| ENSG00000119686 | FLVCR2 | 55640 | 146,801 | -0.951 | 0.205 | -4,636 | 0.0000 | 0.0001 | 94 |
| ENSG00000185112 | FAM43A | 131583 | 1,646.780 | -0.945 | 0.168 | -5,613 | 0.0000 | 0.0000 | 94 |
| ENSG00000204682 | MIR1915HG | 399726 | 105,037 | -0.941 | 0.238 | -3,957 | 0.0001 | 0.0019 | NA |
| ENSG00000008300 | CELSR3 | 1951 | 260,888 | -0.932 | 0.188 | -4,947 | 0.0000 | 0.0000 | 100 |
| ENSG00000164932 | CTHRC1 | 115908 | 2,833.098 | -0.931 | 0.124 | -7,512 | 0.0000 | 0.0000 | 87 |
| ENSG00000105778 | AVL9 | 23080 | 2,421.240 | -0.930 | 0.116 | -8,006 | 0.0000 | 0.0000 | 86 |
| ENSG00000146826 | MAP11 | 55262 | 714,591 | -0.923 | 0.159 | -5,795 | 0.0000 | 0.0000 | NA |
| ENSG00000076258 | FMO4 | 2329 | 61,617 | -0.923 | 0.298 | -3,093 | 0.0020 | 0.0273 | NA |
| ENSG00000152229 | PSTPIP2 | 9050 | 327,033 | -0.922 | 0.174 | -5,292 | 0.0000 | 0.0000 | NA |
| ENSG00000180660 | MAB21L1 | 4081 | 745,049 | -0.919 | 0.159 | -5,778 | 0.0000 | 0.0000 | 94 |
| ENSG00000164465 | DCBLD1 | 285761 | 1,490.340 | -0.916 | 0.120 | -7,627 | 0.0000 | 0.0000 | NA |
| ENSG00000162624 | LHX8 | 431707 | 830,046 | -0.906 | 0.122 | -7,398 | 0.0000 | 0.0000 | 99 |
| ENSG00000120889 | TNFRSF10B | 8795 | 5,868.297 | -0.905 | 0.112 | -8,109 | 0.0000 | 0.0000 | 62 |
| ENSG00000100241 | SBF1 | 6305 | 4,691.357 | -0.904 | 0.136 | -6,643 | 0.0000 | 0.0000 | 80 |
| ENSG00000233695 |  |  | 121,179 | -0.903 | 0.233 | -3,883 | 0.0001 | 0.0024 | NA |
| ENSG00000108381 | ASPA | 443 | 47,320 | -0.903 | 0.318 | -2,841 | 0.0045 | 0.0514 | NA |
| ENSG00000168309 | FAM107A | 11170 | 1,033.020 | -0.901 | 0.340 | -2,648 | 0.0081 | 0.0795 | NA |
| ENSG00000089818 | NECAP1 | 25977 | 1,527.031 | -0.898 | 0.101 | -8,876 | 0.0000 | 0.0000 | 97 |
| ENSG00000153214 | TMEM87B | 84910 | 1,926.698 | -0.898 | 0.111 | -8,069 | 0.0000 | 0.0000 | 93 |
| ENSG00000147894 | C9orf72 | 203228 | 120,927 | -0.891 | 0.231 | -3,855 | 0.0001 | 0.0027 | 99 |
| ENSG00000277586 | NEFL | 4747 | 86,878 | -0.891 | 0.260 | -3,424 | 0.0006 | 0.0106 | 84 |
| ENSG00000122547 | EEPD1 | 80820 | 183,191 | -0.887 | 0.213 | -4,156 | 0.0000 | 0.0009 | 60 |
| ENSG00000113552 | GNPDA1 | 10007 | 2,928.362 | -0.886 | 0.111 | -7,981 | 0.0000 | 0.0000 | 84 |
| ENSG00000146250 | PRSS35 | 167681 | 101,294 | -0.879 | 0.329 | -2,668 | 0.0076 | 0.0764 | NA |
| ENSG00000244459 |  |  | 46,309 | -0.873 | 0.334 | -2,610 | 0.0091 | 0.0854 | NA |
| ENSG00000049239 | H6PD | 9563 | 3,499.500 | -0.872 | 0.118 | -7,369 | 0.0000 | 0.0000 | 60 |
| ENSG00000138650 | PCDH10 | 57575 | 544,077 | -0.870 | 0.135 | -6,424 | 0.0000 | 0.0000 | 77 |
| ENSG00000111450 | STX2 | 2054 | 1,515.102 | -0.868 | 0.124 | -7,007 | 0.0000 | 0.0000 | 95 |
| ENSG00000116704 | SLC35D1 | 23169 | 1,135.685 | -0.867 | 0.131 | -6,640 | 0.0000 | 0.0000 | NA |
| ENSG00000136531 | SCN2A | 6326 | 172,422 | -0.867 | 0.201 | -4,305 | 0.0000 | 0.0005 | 100 |
| ENSG00000150867 | PIP4K2A | 5305 | 1,442.169 | -0.867 | 0.112 | -7,737 | 0.0000 | 0.0000 | 99 |
| ENSG00000164091 | WDR82 | 80335 | 3,717.188 | -0.864 | 0.118 | -7,336 | 0.0000 | 0.0000 | 95 |
| ENSG00000178996 | SNX18 | 112574 | 2,438.275 | -0.863 | 0.092 | -9,422 | 0.0000 | 0.0000 | 98 |
| ENSG00000164105 | SAP30 | 8819 | 441,094 | -0.862 | 0.161 | -5,363 | 0.0000 | 0.0000 | 61 |
| ENSG00000280798 | LINC00294 | 283267 | 202,506 | -0.856 | 0.170 | -5,038 | 0.0000 | 0.0000 | NA |
| ENSG00000198324 | PHETA1 | 144717 | 661,878 | -0.856 | 0.140 | -6,110 | 0.0000 | 0.0000 | 58 |
| ENSG00000152689 | RASGRP3 | 25780 | 742,656 | -0.852 | 0.170 | -5,002 | 0.0000 | 0.0000 | NA |
| ENSG00000197506 | SLC28A3 | 64078 | 124,947 | -0.849 | 0.213 | -3,982 | 0.0001 | 0.0017 | 55 |
| ENSG00000162063 | CCNF | 899 | 1,027.505 | -0.848 | 0.135 | -6,272 | 0.0000 | 0.0000 | NA |
| ENSG00000153904 | DDAH1 | 23576 | 3,989.525 | -0.843 | 0.121 | -6,953 | 0.0000 | 0.0000 | 96 |
| ENSG00000049245 | VAMP3 | 9341 | 5,579.512 | -0.842 | 0.105 | -8,008 | 0.0000 | 0.0000 | NA |
| ENSG00000196961 | AP2A1 | 160 | 8,378.087 | -0.840 | 0.115 | -7,282 | 0.0000 | 0.0000 | 79 |
| ENSG00000101134 | DOK5 | 55816 | 539,965 | -0.831 | 0.131 | -6,325 | 0.0000 | 0.0000 | 93 |
| ENSG00000134690 | CDCA8 | 55143 | 1,696.698 | -0.829 | 0.156 | -5,325 | 0.0000 | 0.0000 | NA |
| ENSG00000138641 | HERC3 | 8916 | 1,519.901 | -0.828 | 0.125 | -6,635 | 0.0000 | 0.0000 | 76 |
| ENSG00000144827 | ABHD10 | 55347 | 689,215 | -0.825 | 0.141 | -5,833 | 0.0000 | 0.0000 | 83 |
| ENSG00000086289 | EPDR1 | 54749 | 2,941.182 | -0.820 | 0.095 | -8,638 | 0.0000 | 0.0000 | 64 |
| ENSG00000164604 | GPR85 | 54329 | 139,558 | -0.819 | 0.205 | -3,999 | 0.0001 | 0.0016 | NA |
| ENSG00000107566 | ERLIN1 | 10613 | 3,756.260 | -0.819 | 0.119 | -6,894 | 0.0000 | 0.0000 | 94 |
| ENSG00000103042 | SLC38A7 | 55238 | 2,836.750 | -0.816 | 0.098 | -8,311 | 0.0000 | 0.0000 | 93 |
| ENSG00000178904 | DPY19L3 | 147991 | 985,938 | -0.815 | 0.140 | -5,822 | 0.0000 | 0.0000 | 86 |
| ENSG00000006634 | DBF4 | 10926 | 516,918 | -0.814 | 0.203 | -4,006 | 0.0001 | 0.0016 | 79 |
| ENSG00000092931 | MFSD11 | 79157 | 994,236 | -0.812 | 0.126 | -6,449 | 0.0000 | 0.0000 | NA |
| ENSG00000149294 | NCAM1 | 4684 | 126,224 | -0.812 | 0.311 | -2,614 | 0.0089 | 0.0847 | 99 |
| ENSG00000184661 | CDCA2 | 157313 | 1,268.916 | -0.811 | 0.179 | -4,519 | 0.0000 | 0.0002 | NA |
| ENSG00000149260 | CAPN5 | 726 | 1,899.690 | -0.809 | 0.125 | -6,458 | 0.0000 | 0.0000 | 86 |
| ENSG00000185619 | PCGF3 | 10336 | 1,460.908 | -0.809 | 0.101 | -7,981 | 0.0000 | 0.0000 | 69 |
| ENSG00000197265 | GTF2E2 | 2961 | 1,366.763 | -0.807 | 0.101 | -8,031 | 0.0000 | 0.0000 | NA |
| ENSG00000162384 | CZIB | 54987 | 847,325 | -0.807 | 0.115 | -7,023 | 0.0000 | 0.0000 | NA |
| ENSG00000121039 | RDH10 | 157506 | 3,960.387 | -0.806 | 0.161 | -5,017 | 0.0000 | 0.0000 | NA |
| ENSG00000069869 | NEDD4 | 4734 | 4,146.130 | -0.806 | 0.136 | -5,941 | 0.0000 | 0.0000 | 98 |
| ENSG00000107789 | MINPP1 | 9562 | 762,645 | -0.802 | 0.112 | -7,145 | 0.0000 | 0.0000 | 56 |
| ENSG00000069020 | MAST4 | 375449 | 526,924 | -0.797 | 0.156 | -5,122 | 0.0000 | 0.0000 | 98 |
| ENSG00000091157 | WDR7 | 23335 | 679,940 | -0.796 | 0.134 | -5,955 | 0.0000 | 0.0000 | 100 |
| ENSG00000169139 | UBE2V2 | 7336 | 1,209.601 | -0.792 | 0.127 | -6,223 | 0.0000 | 0.0000 | 95 |
| ENSG00000103174 | NAGPA | 51172 | 984,151 | -0.787 | 0.140 | -5,622 | 0.0000 | 0.0000 | 71 |
| ENSG00000086848 | ALG9 | 79796 | 225,350 | -0.786 | 0.179 | -4,384 | 0.0000 | 0.0004 | NA |
| ENSG00000164305 | CASP3 | 836 | 2,705.042 | -0.784 | 0.108 | -7,274 | 0.0000 | 0.0000 | NA |
| ENSG00000144681 | STAC | 6769 | 343,667 | -0.780 | 0.170 | -4,585 | 0.0000 | 0.0002 | 95 |
| ENSG00000138604 | GLCE | 26035 | 674,394 | -0.780 | 0.175 | -4,449 | 0.0000 | 0.0003 | 78 |
| ENSG00000113356 | POLR3G | 10622 | 207,680 | -0.775 | 0.180 | -4,310 | 0.0000 | 0.0005 | 61 |
| ENSG00000180957 | PITPNB | 23760 | 2,113.139 | -0.772 | 0.114 | -6,766 | 0.0000 | 0.0000 | 57 |
| ENSG00000100744 | GSKIP | 51527 | 698,535 | -0.771 | 0.144 | -5,363 | 0.0000 | 0.0000 | 85 |
| ENSG00000126003 | PLAGL2 | 5326 | 760,524 | -0.770 | 0.106 | -7,248 | 0.0000 | 0.0000 | 98 |
| ENSG00000143515 | ATP8B2 | 57198 | 9,170.526 | -0.768 | 0.125 | -6,168 | 0.0000 | 0.0000 | 57 |
| ENSG00000139998 | RAB15 | 376267 | 805,601 | -0.767 | 0.121 | -6,342 | 0.0000 | 0.0000 | 82 |
| ENSG00000103034 | NDRG4 | 65009 | 170,261 | -0.765 | 0.232 | -3,304 | 0.0010 | 0.0151 | NA |
| ENSG00000117650 | NEK2 | 4751 | 603,340 | -0.762 | 0.184 | -4,138 | 0.0000 | 0.0010 | NA |
| ENSG00000105255 | FSD1 | 79187 | 58,013 | -0.762 | 0.284 | -2,685 | 0.0073 | 0.0738 | NA |
| ENSG00000157456 | CCNB2 | 9133 | 1,225.517 | -0.759 | 0.145 | -5,244 | 0.0000 | 0.0000 | NA |
| ENSG00000139625 | MAP3K12 | 7786 | 1,463.140 | -0.756 | 0.141 | -5,369 | 0.0000 | 0.0000 | 78 |
| ENSG00000101447 | FAM83D | 81610 | 1,148.098 | -0.754 | 0.166 | -4,551 | 0.0000 | 0.0002 | NA |
| ENSG00000164125 | GASK1B | 51313 | 405,635 | -0.748 | 0.208 | -3,595 | 0.0003 | 0.0063 | NA |
| ENSG00000112984 | KIF20A | 10112 | 2,563.834 | -0.741 | 0.126 | -5,873 | 0.0000 | 0.0000 | NA |
| ENSG00000116497 | S100PBP | 64766 | 662,186 | -0.741 | 0.141 | -5,259 | 0.0000 | 0.0000 | 96 |
| ENSG00000166851 | PLK1 | 5347 | 2,208.487 | -0.739 | 0.144 | -5,144 | 0.0000 | 0.0000 | NA |
| ENSG00000178033 | CALHM5 | 254228 | 1,037.418 | -0.733 | 0.178 | -4,111 | 0.0000 | 0.0011 | NA |
| ENSG00000181826 | RELL1 | 768211 | 183,078 | -0.733 | 0.181 | -4,060 | 0.0000 | 0.0013 | 78 |
| ENSG00000103978 | TMEM87A | 25963 | 1,233.020 | -0.732 | 0.113 | -6,498 | 0.0000 | 0.0000 | 90 |
| ENSG00000138459 | SLC35A5 | 55032 | 1,079.127 | -0.731 | 0.124 | -5,911 | 0.0000 | 0.0000 | 71 |
| ENSG00000236393 | LOC101927476 | 1E+08 | 129,848 | -0.729 | 0.219 | -3,332 | 0.0009 | 0.0140 | NA |
| ENSG00000131386 | GALNT15 | 117248 | 293,901 | -0.727 | 0.174 | -4,173 | 0.0000 | 0.0009 | NA |
| ENSG00000175931 | UBE2O | 63893 | 1,233.658 | -0.726 | 0.118 | -6,178 | 0.0000 | 0.0000 | NA |
| ENSG00000156671 | SAMD8 | 142891 | 1,347.433 | -0.725 | 0.109 | -6,619 | 0.0000 | 0.0000 | 96 |
| ENSG00000160145 | KALRN | 8997 | 125,259 | -0.724 | 0.243 | -2,984 | 0.0028 | 0.0360 | NA |
| ENSG00000175414 | ARL10 | 285598 | 482,153 | -0.724 | 0.133 | -5,428 | 0.0000 | 0.0000 | 55 |
| ENSG00000140451 | PIF1 | 80119 | 377,140 | -0.723 | 0.153 | -4,717 | 0.0000 | 0.0001 | NA |
| ENSG00000161888 | SPC24 | 147841 | 519,601 | -0.723 | 0.175 | -4,138 | 0.0000 | 0.0010 | NA |
| ENSG00000171502 | COL24A1 | 255631 | 68,151 | -0.723 | 0.265 | -2,732 | 0.0063 | 0.0660 | NA |
| ENSG00000186193 | SAPCD2 | 89958 | 426,369 | -0.720 | 0.171 | -4,205 | 0.0000 | 0.0008 | NA |
| ENSG00000145687 | SSBP2 | 23635 | 485,195 | -0.720 | 0.145 | -4,951 | 0.0000 | 0.0000 | 76 |
| ENSG00000141449 | GREB1L | 80000 | 240,732 | -0.717 | 0.215 | -3,328 | 0.0009 | 0.0141 | NA |
| ENSG00000167130 | DOLPP1 | 57171 | 605,632 | -0.715 | 0.124 | -5,775 | 0.0000 | 0.0000 | 97 |
| ENSG00000180694 | TMEM64 | 169200 | 474,162 | -0.714 | 0.142 | -5,034 | 0.0000 | 0.0000 | NA |
| ENSG00000205356 | TECPR1 | 25851 | 1,200.798 | -0.714 | 0.120 | -5,948 | 0.0000 | 0.0000 | NA |
| ENSG00000140297 | GCNT3 | 9245 | 409,593 | -0.714 | 0.178 | -4,018 | 0.0001 | 0.0015 | NA |
| ENSG00000158402 | CDC25C | 995 | 238,766 | -0.714 | 0.221 | -3,233 | 0.0012 | 0.0186 | NA |
| ENSG00000162694 | EXTL2 | 2135 | 518,888 | -0.713 | 0.133 | -5,367 | 0.0000 | 0.0000 | 97 |
| ENSG00000118508 | RAB32 | 10981 | 2,930.260 | -0.713 | 0.082 | -8,706 | 0.0000 | 0.0000 | 94 |
| ENSG00000175575 | PAAF1 | 80227 | 587,087 | -0.713 | 0.134 | -5,312 | 0.0000 | 0.0000 | 91 |
| ENSG00000120693 | SMAD9 | 4093 | 178,209 | -0.712 | 0.183 | -3,886 | 0.0001 | 0.0024 | NA |
| ENSG00000155100 | OTUD6B | 51633 | 401,826 | -0.712 | 0.169 | -4,222 | 0.0000 | 0.0007 | 96 |
| ENSG00000129195 | PIMREG | 54478 | 1,078.348 | -0.711 | 0.128 | -5,545 | 0.0000 | 0.0000 | NA |
| ENSG00000075213 | SEMA3A | 10371 | 2,690.211 | -0.710 | 0.103 | -6,908 | 0.0000 | 0.0000 | 83 |
| ENSG00000151617 | EDNRA | 1909 | 287,874 | -0.708 | 0.171 | -4,133 | 0.0000 | 0.0010 | 92 |
| ENSG00000119698 | PPP4R4 | 57718 | 510,192 | -0.708 | 0.128 | -5,515 | 0.0000 | 0.0000 | 90 |
| ENSG00000179387 | ELMOD2 | 255520 | 605,172 | -0.708 | 0.133 | -5,336 | 0.0000 | 0.0000 | 84 |
| ENSG00000112245 | PTP4A1 | 7803 | 4,632.717 | -0.707 | 0.128 | -5,543 | 0.0000 | 0.0000 | 97 |
| ENSG00000021574 | SPAST | 6683 | 373,577 | -0.705 | 0.140 | -5,045 | 0.0000 | 0.0000 | 93 |
| ENSG00000064655 | EYA2 | 2139 | 365,013 | -0.704 | 0.135 | -5,224 | 0.0000 | 0.0000 | 75 |
| ENSG00000121152 | NCAPH | 23397 | 831,801 | -0.703 | 0.154 | -4,553 | 0.0000 | 0.0002 | NA |
| ENSG00000065057 | NTHL1 | 4913 | 188,644 | -0.703 | 0.175 | -4,027 | 0.0001 | 0.0015 | NA |
| ENSG00000126787 | DLGAP5 | 9787 | 1,416.891 | -0.699 | 0.180 | -3,890 | 0.0001 | 0.0024 | NA |
| ENSG00000182372 | CLN8 | 2055 | 632,079 | -0.698 | 0.116 | -6,010 | 0.0000 | 0.0000 | NA |
| ENSG00000144580 | CNOT9 | 9125 | 2,265.694 | -0.698 | 0.114 | -6,103 | 0.0000 | 0.0000 | 96 |
| ENSG00000155096 | AZIN1 | 51582 | 5,123.096 | -0.695 | 0.115 | -6,070 | 0.0000 | 0.0000 | 96 |
| ENSG00000218336 | TENM3 | 55714 | 271,093 | -0.695 | 0.168 | -4,130 | 0.0000 | 0.0010 | 92 |
| ENSG00000011426 | ANLN | 54443 | 7,515.621 | -0.695 | 0.165 | -4,208 | 0.0000 | 0.0008 | 54 |
| ENSG00000169221 | TBC1D10B | 26000 | 3,054.288 | -0.693 | 0.141 | -4,906 | 0.0000 | 0.0000 | 95 |
| ENSG00000115232 | ITGA4 | 3676 | 1,382.990 | -0.692 | 0.135 | -5,139 | 0.0000 | 0.0000 | 53 |
| ENSG00000066629 | EML1 | 2009 | 4,012.721 | -0.686 | 0.118 | -5,831 | 0.0000 | 0.0000 | 97 |
| ENSG00000080819 | CPOX | 1371 | 839,214 | -0.685 | 0.119 | -5,772 | 0.0000 | 0.0000 | NA |
| ENSG00000165891 | E2F7 | 144455 | 1,204.701 | -0.685 | 0.154 | -4,461 | 0.0000 | 0.0003 | 97 |
| ENSG00000176909 | MAMSTR | 284358 | 107,297 | -0.684 | 0.240 | -2,848 | 0.0044 | 0.0507 | NA |
| ENSG00000092068 | SLC7A8 | 23428 | 1,389.143 | -0.680 | 0.139 | -4,875 | 0.0000 | 0.0000 | NA |
| ENSG00000132326 | PER2 | 8864 | 152,401 | -0.680 | 0.199 | -3,410 | 0.0007 | 0.0110 | 93 |
| ENSG00000173852 | DPY19L1 | 23333 | 1,138.960 | -0.678 | 0.137 | -4,952 | 0.0000 | 0.0000 | 86 |
| ENSG00000107282 | APBA1 | 320 | 394,569 | -0.677 | 0.166 | -4,077 | 0.0000 | 0.0012 | 91 |
| ENSG00000090905 | TNRC6A | 27327 | 3,257.272 | -0.675 | 0.100 | -6,774 | 0.0000 | 0.0000 | 99 |
| ENSG00000147642 | SYBU | 55638 | 257,279 | -0.672 | 0.182 | -3,687 | 0.0002 | 0.0047 | NA |
| ENSG00000135451 | TROAP | 10024 | 825,737 | -0.670 | 0.133 | -5,050 | 0.0000 | 0.0000 | NA |
| ENSG00000148848 | ADAM12 | 8038 | 4,684.912 | -0.667 | 0.099 | -6,769 | 0.0000 | 0.0000 | 84 |
| ENSG00000213551 | DNAJC9 | 23234 | 711,617 | -0.666 | 0.121 | -5,519 | 0.0000 | 0.0000 | NA |
| ENSG00000119318 | RAD23B | 5887 | 7,734.423 | -0.666 | 0.090 | -7,359 | 0.0000 | 0.0000 | 91 |
| ENSG00000115163 | CENPA | 1058 | 387,343 | -0.665 | 0.166 | -4,004 | 0.0001 | 0.0016 | NA |
| ENSG00000172296 | SPTLC3 | 55304 | 371,881 | -0.661 | 0.194 | -3,404 | 0.0007 | 0.0112 | 72 |
| ENSG00000165507 | DEPP1 | 11067 | 222,930 | -0.660 | 0.228 | -2,892 | 0.0038 | 0.0454 | NA |
| ENSG00000173530 | TNFRSF10D | 8793 | 1,405.190 | -0.659 | 0.133 | -4,967 | 0.0000 | 0.0000 | NA |
| ENSG00000168228 | ZCCHC4 | 29063 | 332,497 | -0.658 | 0.151 | -4,349 | 0.0000 | 0.0004 | NA |
| ENSG00000130270 | ATP8B3 | 148229 | 209,215 | -0.655 | 0.185 | -3,538 | 0.0004 | 0.0075 | NA |
| ENSG00000198483 | ANKRD35 | 148741 | 126,872 | -0.655 | 0.232 | -2,823 | 0.0048 | 0.0536 | NA |
| ENSG00000186364 | NUDT17 | 200035 | 66,491 | -0.655 | 0.258 | -2,536 | 0.0112 | 0.0988 | NA |
| ENSG00000083937 | CHMP2B | 25978 | 1,351.034 | -0.654 | 0.122 | -5,378 | 0.0000 | 0.0000 | 87 |
| ENSG00000185361 | TNFAIP8L1 | 126282 | 231,714 | -0.653 | 0.175 | -3,730 | 0.0002 | 0.0041 | NA |
| ENSG00000124813 | RUNX2 | 860 | 544,868 | -0.653 | 0.149 | -4,385 | 0.0000 | 0.0004 | 96 |
| ENSG00000169122 | FAM110B | 90362 | 271,414 | -0.653 | 0.147 | -4,452 | 0.0000 | 0.0003 | 93 |
| ENSG00000089123 | TASP1 | 55617 | 141,137 | -0.652 | 0.191 | -3,417 | 0.0006 | 0.0108 | 88 |
| ENSG00000112078 | KCTD20 | 222658 | 3,881.396 | -0.652 | 0.108 | -6,026 | 0.0000 | 0.0000 | 76 |
| ENSG00000130560 | UBAC1 | 10422 | 1,077.353 | -0.642 | 0.116 | -5,553 | 0.0000 | 0.0000 | 89 |
| ENSG00000179151 | EDC3 | 80153 | 1,058.843 | -0.642 | 0.101 | -6,359 | 0.0000 | 0.0000 | 86 |
| ENSG00000158470 | B4GALT5 | 9334 | 2,938.327 | -0.642 | 0.109 | -5,918 | 0.0000 | 0.0000 | 74 |
| ENSG00000124659 | TBCC | 6903 | 937,534 | -0.635 | 0.141 | -4,506 | 0.0000 | 0.0002 | NA |
| ENSG00000136108 | CKAP2 | 26586 | 2,264.245 | -0.634 | 0.134 | -4,747 | 0.0000 | 0.0001 | NA |
| ENSG00000138395 | CDK15 | 65061 | 226,413 | -0.633 | 0.178 | -3,555 | 0.0004 | 0.0071 | NA |
| ENSG00000067221 | STOML1 | 9399 | 1,122.732 | -0.632 | 0.167 | -3,782 | 0.0002 | 0.0034 | NA |
| ENSG00000205268 | PDE7A | 5150 | 248,924 | -0.631 | 0.170 | -3,708 | 0.0002 | 0.0044 | 99 |
| ENSG00000153922 | CHD1 | 1105 | 1,028.004 | -0.631 | 0.139 | -4,555 | 0.0000 | 0.0002 | 98 |
| ENSG00000163462 | TRIM46 | 80128 | 167,714 | -0.630 | 0.245 | -2,567 | 0.0103 | 0.0926 | NA |
| ENSG00000186310 | NAP1L3 | 4675 | 284,872 | -0.630 | 0.171 | -3,690 | 0.0002 | 0.0047 | 61 |
| ENSG00000103196 | CRISPLD2 | 83716 | 1,067.792 | -0.628 | 0.124 | -5,049 | 0.0000 | 0.0000 | NA |
| ENSG00000230417 | LINC00595 | 414243 | 496,757 | -0.627 | 0.154 | -4,057 | 0.0000 | 0.0013 | NA |
| ENSG00000184182 | UBE2F | 140739 | 307,635 | -0.626 | 0.155 | -4,026 | 0.0001 | 0.0015 | 62 |
| ENSG00000079257 | LXN | 56925 | 178,637 | -0.624 | 0.223 | -2,793 | 0.0052 | 0.0575 | NA |
| ENSG00000106993 | CDC37L1 | 55664 | 352,542 | -0.622 | 0.148 | -4,213 | 0.0000 | 0.0008 | NA |
| ENSG00000111665 | CDCA3 | 83461 | 1,238.485 | -0.622 | 0.154 | -4,044 | 0.0001 | 0.0014 | NA |
| ENSG00000150764 | DIXDC1 | 85458 | 399,015 | -0.622 | 0.188 | -3,310 | 0.0009 | 0.0149 | 61 |
| ENSG00000131558 | EXOC4 | 60412 | 2,460.064 | -0.622 | 0.116 | -5,346 | 0.0000 | 0.0000 | 50 |
| ENSG00000143924 | EML4 | 27436 | 1,408.373 | -0.620 | 0.117 | -5,291 | 0.0000 | 0.0000 | 96 |
| ENSG00000160208 | RRP1B | 23076 | 1,827.922 | -0.620 | 0.119 | -5,224 | 0.0000 | 0.0000 | 66 |
| ENSG00000175182 | FAM131A | 131408 | 310,372 | -0.619 | 0.171 | -3,612 | 0.0003 | 0.0059 | NA |
| ENSG00000100304 | TTLL12 | 23170 | 1,762.960 | -0.618 | 0.122 | -5,070 | 0.0000 | 0.0000 | NA |
| ENSG00000163808 | KIF15 | 56992 | 417,267 | -0.618 | 0.210 | -2,936 | 0.0033 | 0.0406 | NA |
| ENSG00000167191 | GPRC5B | 51704 | 472,503 | -0.617 | 0.144 | -4,276 | 0.0000 | 0.0006 | NA |
| ENSG00000240370 | RPL13P5 | 283345 | 78,385 | -0.617 | 0.230 | -2,677 | 0.0074 | 0.0750 | NA |
| ENSG00000169760 | NLGN1 | 22871 | 134,459 | -0.617 | 0.194 | -3,185 | 0.0014 | 0.0213 | 88 |
| ENSG00000138316 | ADAMTS14 | 140766 | 1,226.814 | -0.615 | 0.164 | -3,755 | 0.0002 | 0.0038 | NA |
| ENSG00000118985 | ELL2 | 22936 | 5,248.936 | -0.614 | 0.127 | -4,851 | 0.0000 | 0.0001 | 98 |
| ENSG00000172197 | MBOAT1 | 154141 | 121,641 | -0.614 | 0.240 | -2,563 | 0.0104 | 0.0934 | 86 |
| ENSG00000161513 | FDXR | 2232 | 1,248.047 | -0.613 | 0.142 | -4,328 | 0.0000 | 0.0005 | NA |
| ENSG00000174332 | GLIS1 | 148979 | 204,674 | -0.613 | 0.197 | -3,114 | 0.0018 | 0.0257 | NA |
| ENSG00000013375 | PGM3 | 5238 | 2,090.127 | -0.613 | 0.126 | -4,868 | 0.0000 | 0.0000 | 87 |
| ENSG00000100100 | PIK3IP1 | 113791 | 914,638 | -0.612 | 0.168 | -3,651 | 0.0003 | 0.0053 | NA |
| ENSG00000144746 | ARL6IP5 | 10550 | 5,290.021 | -0.612 | 0.177 | -3,446 | 0.0006 | 0.0100 | NA |
| ENSG00000176597 | B3GNT5 | 84002 | 131,446 | -0.612 | 0.232 | -2,645 | 0.0082 | 0.0799 | 100 |
| ENSG00000164850 | GPER1 | 2852 | 237,687 | -0.609 | 0.192 | -3,164 | 0.0016 | 0.0226 | NA |
| ENSG00000113595 | TRIM23 | 373 | 429,073 | -0.608 | 0.148 | -4,103 | 0.0000 | 0.0011 | 68 |
| ENSG00000134057 | CCNB1 | 891 | 3,413.501 | -0.606 | 0.151 | -4,025 | 0.0001 | 0.0015 | NA |
| ENSG00000183496 | MEX3B | 84206 | 155,928 | -0.606 | 0.201 | -3,014 | 0.0026 | 0.0333 | 92 |
| ENSG00000113721 | PDGFRB | 5159 | 4,102.121 | -0.606 | 0.152 | -3,992 | 0.0001 | 0.0017 | 84 |
| ENSG00000166579 | NDEL1 | 81565 | 2,766.359 | -0.603 | 0.094 | -6,401 | 0.0000 | 0.0000 | 91 |
| ENSG00000112312 | GMNN | 51053 | 691,236 | -0.602 | 0.144 | -4,182 | 0.0000 | 0.0008 | NA |
| ENSG00000180758 | GPR157 | 80045 | 316,137 | -0.601 | 0.146 | -4,126 | 0.0000 | 0.0010 | NA |
| ENSG00000006638 | TBXA2R | 6915 | 98,862 | -0.601 | 0.230 | -2,610 | 0.0091 | 0.0854 | NA |
| ENSG00000104497 | SNX16 | 64089 | 176,842 | -0.601 | 0.194 | -3,102 | 0.0019 | 0.0265 | 99 |
| ENSG00000171928 | TVP23B | 51030 | 971,380 | -0.600 | 0.137 | -4,388 | 0.0000 | 0.0004 | 72 |
| ENSG00000182107 | TMEM30B | 161291 | 132,628 | -0.599 | 0.193 | -3,110 | 0.0019 | 0.0260 | NA |
| ENSG00000213064 | SFT2D2 | 375035 | 1,142.079 | -0.598 | 0.115 | -5,195 | 0.0000 | 0.0000 | NA |
| ENSG00000117748 | RPA2 | 6118 | 1,412.433 | -0.596 | 0.122 | -4,889 | 0.0000 | 0.0000 | NA |
| ENSG00000138160 | KIF11 | 3832 | 1,759.443 | -0.593 | 0.164 | -3,622 | 0.0003 | 0.0058 | 84 |
| ENSG00000004777 | ARHGAP33 | 115703 | 321,410 | -0.592 | 0.155 | -3,825 | 0.0001 | 0.0030 | NA |
| ENSG00000133169 | BEX1 | 55859 | 1,788.350 | -0.591 | 0.117 | -5,049 | 0.0000 | 0.0000 | NA |
| ENSG00000134253 | TRIM45 | 80263 | 110,541 | -0.590 | 0.213 | -2,765 | 0.0057 | 0.0611 | NA |
| ENSG00000165476 | REEP3 | 221035 | 1,687.389 | -0.589 | 0.130 | -4,528 | 0.0000 | 0.0002 | 97 |
| ENSG00000259207 | ITGB3 | 3690 | 809,428 | -0.588 | 0.147 | -4,001 | 0.0001 | 0.0016 | 87 |
| ENSG00000106477 | CEP41 | 95681 | 491,906 | -0.587 | 0.139 | -4,214 | 0.0000 | 0.0008 | 94 |
| ENSG00000136982 | DSCC1 | 79075 | 267,094 | -0.587 | 0.209 | -2,811 | 0.0049 | 0.0553 | 61 |
| ENSG00000171492 | LRRC8D | 55144 | 978,678 | -0.586 | 0.122 | -4,815 | 0.0000 | 0.0001 | 92 |
| ENSG00000142945 | KIF2C | 11004 | 1,633.605 | -0.585 | 0.161 | -3,635 | 0.0003 | 0.0055 | NA |
| ENSG00000168874 | ATOH8 | 84913 | 1,044.899 | -0.585 | 0.163 | -3,584 | 0.0003 | 0.0065 | NA |
| ENSG00000144959 | NCEH1 | 57552 | 4,618.704 | -0.585 | 0.127 | -4,616 | 0.0000 | 0.0001 | 70 |
| ENSG00000168092 | PAFAH1B2 | 5049 | 2,895.291 | -0.583 | 0.104 | -5,620 | 0.0000 | 0.0000 | 62 |
| ENSG00000165526 | RPUSD4 | 84881 | 653,541 | -0.580 | 0.109 | -5,335 | 0.0000 | 0.0000 | 69 |
| ENSG00000048392 | RRM2B | 50484 | 1,905.729 | -0.579 | 0.150 | -3,856 | 0.0001 | 0.0027 | NA |
| ENSG00000165304 | MELK | 9833 | 1,804.357 | -0.577 | 0.141 | -4,093 | 0.0000 | 0.0012 | NA |
| ENSG00000156140 | ADAMTS3 | 9508 | 158,925 | -0.574 | 0.199 | -2,883 | 0.0039 | 0.0465 | 97 |
| ENSG00000147416 | ATP6V1B2 | 526 | 7,861.254 | -0.571 | 0.111 | -5,138 | 0.0000 | 0.0000 | NA |
| ENSG00000147649 | MTDH | 92140 | 6,323.815 | -0.571 | 0.115 | -4,944 | 0.0000 | 0.0000 | 98 |
| ENSG00000175305 | CCNE2 | 9134 | 175,036 | -0.570 | 0.194 | -2,937 | 0.0033 | 0.0405 | 99 |
| ENSG00000149657 | LSM14B | 149986 | 1,731.426 | -0.569 | 0.103 | -5,535 | 0.0000 | 0.0000 | 71 |
| ENSG00000114423 | CBLB | 868 | 392,901 | -0.567 | 0.141 | -4,013 | 0.0001 | 0.0015 | 92 |
| ENSG00000197818 | SLC9A8 | 23315 | 1,317.031 | -0.567 | 0.133 | -4,256 | 0.0000 | 0.0006 | 87 |
| ENSG00000168917 | SLC35G2 | 80723 | 798,830 | -0.567 | 0.139 | -4,076 | 0.0000 | 0.0012 | 54 |
| ENSG00000198919 | DZIP3 | 9666 | 481,876 | -0.566 | 0.169 | -3,351 | 0.0008 | 0.0133 | NA |
| ENSG00000099957 | P2RX6 | 9127 | 151,221 | -0.566 | 0.209 | -2,710 | 0.0067 | 0.0694 | NA |
| ENSG00000119397 | CNTRL | 11064 | 321,376 | -0.565 | 0.162 | -3,488 | 0.0005 | 0.0087 | NA |
| ENSG00000035499 | DEPDC1B | 55789 | 269,371 | -0.564 | 0.203 | -2,773 | 0.0056 | 0.0602 | NA |
| ENSG00000124098 | FAM210B | 116151 | 2,646.170 | -0.564 | 0.099 | -5,701 | 0.0000 | 0.0000 | 93 |
| ENSG00000019549 | SNAI2 | 6591 | 1,105.687 | -0.564 | 0.124 | -4,544 | 0.0000 | 0.0002 | 89 |
| ENSG00000081320 | STK17B | 9262 | 1,652.700 | -0.563 | 0.124 | -4,546 | 0.0000 | 0.0002 | 90 |
| ENSG00000183856 | IQGAP3 | 128239 | 1,926.709 | -0.561 | 0.155 | -3,619 | 0.0003 | 0.0058 | NA |
| ENSG00000161021 | MAML1 | 9794 | 1,537.252 | -0.561 | 0.113 | -4,973 | 0.0000 | 0.0000 | 89 |
| ENSG00000117399 | CDC20 | 991 | 3,420.385 | -0.560 | 0.133 | -4,206 | 0.0000 | 0.0008 | NA |
| ENSG00000164329 | TENT2 | 167153 | 1,422.292 | -0.559 | 0.123 | -4,530 | 0.0000 | 0.0002 | 97 |
| ENSG00000198246 | SLC29A3 | 55315 | 205,626 | -0.559 | 0.181 | -3,088 | 0.0020 | 0.0276 | 60 |
| ENSG00000132718 | SYT11 | 23208 | 1,882.615 | -0.556 | 0.113 | -4,907 | 0.0000 | 0.0000 | NA |
| ENSG00000130962 | PRRG1 | 5638 | 623,760 | -0.553 | 0.152 | -3,631 | 0.0003 | 0.0056 | NA |
| ENSG00000133424 | LARGE1 | 9215 | 900,154 | -0.553 | 0.111 | -4,983 | 0.0000 | 0.0000 | 94 |
| ENSG00000145391 | SETD7 | 80854 | 5,780.919 | -0.552 | 0.105 | -5,241 | 0.0000 | 0.0000 | 92 |
| ENSG00000154328 | NEIL2 | 252969 | 521,341 | -0.552 | 0.141 | -3,907 | 0.0001 | 0.0022 | 60 |
| ENSG00000138778 | CENPE | 1062 | 1,366.685 | -0.548 | 0.195 | -2,806 | 0.0050 | 0.0559 | NA |
| ENSG00000109265 | CRACD | 57482 | 130,670 | -0.548 | 0.204 | -2,691 | 0.0071 | 0.0725 | NA |
| ENSG00000151233 | GXYLT1 | 283464 | 866,635 | -0.548 | 0.169 | -3,250 | 0.0012 | 0.0177 | 74 |
| ENSG00000171223 | JUNB | 3726 | 1,474.617 | -0.548 | 0.192 | -2,853 | 0.0043 | 0.0500 | 70 |
| ENSG00000137338 | PGBD1 | 84547 | 274,913 | -0.547 | 0.160 | -3,412 | 0.0006 | 0.0109 | NA |
| ENSG00000214357 | NEURL1B | 54492 | 347,397 | -0.547 | 0.182 | -3,007 | 0.0026 | 0.0339 | 95 |
| ENSG00000075218 | GTSE1 | 51512 | 1,521.229 | -0.545 | 0.147 | -3,707 | 0.0002 | 0.0044 | NA |
| ENSG00000163431 | LMOD1 | 25802 | 254,224 | -0.545 | 0.194 | -2,811 | 0.0049 | 0.0553 | NA |
| ENSG00000156011 | PSD3 | 23362 | 2,678.390 | -0.545 | 0.103 | -5,306 | 0.0000 | 0.0000 | 85 |
| ENSG00000136295 | TTYH3 | 80727 | 10,664.212 | -0.544 | 0.121 | -4,495 | 0.0000 | 0.0002 | NA |
| ENSG00000049759 | NEDD4L | 23327 | 1,842.309 | -0.544 | 0.104 | -5,211 | 0.0000 | 0.0000 | 80 |
| ENSG00000179152 | TCAIM | 285343 | 791,515 | -0.543 | 0.139 | -3,905 | 0.0001 | 0.0022 | NA |
| ENSG00000149639 | SOGA1 | 140710 | 3,596.430 | -0.542 | 0.122 | -4,435 | 0.0000 | 0.0003 | 84 |
| ENSG00000102096 | PIM2 | 11040 | 359,393 | -0.540 | 0.149 | -3,620 | 0.0003 | 0.0058 | NA |
| ENSG00000148343 | MIGA2 | 84895 | 1,157.545 | -0.540 | 0.127 | -4,261 | 0.0000 | 0.0006 | 76 |
| ENSG00000079950 | STX7 | 8417 | 1,769.111 | -0.539 | 0.126 | -4,273 | 0.0000 | 0.0006 | NA |
| ENSG00000007237 | GAS7 | 8522 | 168,257 | -0.539 | 0.194 | -2,773 | 0.0055 | 0.0602 | NA |
| ENSG00000164938 | TP53INP1 | 94241 | 859,718 | -0.538 | 0.174 | -3,088 | 0.0020 | 0.0276 | 95 |
| ENSG00000081014 | AP4E1 | 23431 | 796,847 | -0.538 | 0.130 | -4,142 | 0.0000 | 0.0010 | 86 |
| ENSG00000162769 | FLVCR1 | 28982 | 132,755 | -0.537 | 0.194 | -2,763 | 0.0057 | 0.0614 | 90 |
| ENSG00000079435 | LIPE | 3991 | 125,380 | -0.536 | 0.207 | -2,587 | 0.0097 | 0.0889 | NA |
| ENSG00000145386 | CCNA2 | 890 | 1,827.958 | -0.534 | 0.147 | -3,630 | 0.0003 | 0.0056 | NA |
| ENSG00000158023 | CFAP251 | 144406 | 244,809 | -0.534 | 0.174 | -3,071 | 0.0021 | 0.0287 | NA |
| ENSG00000181804 | SLC9A9 | 285195 | 317,797 | -0.534 | 0.175 | -3,049 | 0.0023 | 0.0305 | NA |
| ENSG00000151502 | VPS26B | 112936 | 1,536.189 | -0.533 | 0.090 | -5,923 | 0.0000 | 0.0000 | 93 |
| ENSG00000048052 | HDAC9 | 9734 | 1,239.714 | -0.532 | 0.140 | -3,805 | 0.0001 | 0.0032 | 98 |
| ENSG00000164924 | YWHAZ | 7534 | 27,397.870 | -0.530 | 0.114 | -4,667 | 0.0000 | 0.0001 | NA |
| ENSG00000076382 | SPAG5 | 10615 | 1,659.358 | -0.530 | 0.139 | -3,819 | 0.0001 | 0.0030 | NA |
| ENSG00000159214 | CCDC24 | 149473 | 139,818 | -0.529 | 0.205 | -2,583 | 0.0098 | 0.0895 | NA |
| ENSG00000170348 | TMED10 | 10972 | 8,016.129 | -0.528 | 0.092 | -5,727 | 0.0000 | 0.0000 | NA |
| ENSG00000120063 | GNA13 | 10672 | 1,733.095 | -0.528 | 0.126 | -4,184 | 0.0000 | 0.0008 | 91 |
| ENSG00000188158 | NHS | 4810 | 210,137 | -0.528 | 0.171 | -3,084 | 0.0020 | 0.0279 | 84 |
| ENSG00000105849 | POLR1F | 221830 | 578,166 | -0.527 | 0.145 | -3,630 | 0.0003 | 0.0056 | NA |
| ENSG00000163704 | PRRT3 | 285368 | 423,860 | -0.526 | 0.165 | -3,187 | 0.0014 | 0.0213 | NA |
| ENSG00000148019 | CEP78 | 84131 | 607,301 | -0.524 | 0.166 | -3,163 | 0.0016 | 0.0226 | NA |
| ENSG00000141576 | RNF157 | 114804 | 580,832 | -0.524 | 0.148 | -3,529 | 0.0004 | 0.0077 | 82 |
| ENSG00000267106 |  |  | 123,766 | -0.523 | 0.201 | -2,607 | 0.0091 | 0.0858 | NA |
| ENSG00000177732 | SOX12 | 6666 | 1,369.384 | -0.523 | 0.137 | -3,808 | 0.0001 | 0.0032 | 68 |
| ENSG00000168297 | PXK | 54899 | 1,159.765 | -0.521 | 0.143 | -3,633 | 0.0003 | 0.0056 | NA |
| ENSG00000213694 | S1PR3 | 1903 | 3,367.106 | -0.519 | 0.123 | -4,232 | 0.0000 | 0.0007 | NA |
| ENSG00000134769 | DTNA | 1837 | 427,178 | -0.519 | 0.149 | -3,496 | 0.0005 | 0.0085 | NA |
| ENSG00000167100 | SAMD14 | 201191 | 296,477 | -0.519 | 0.163 | -3,187 | 0.0014 | 0.0213 | NA |
| ENSG00000106366 | SERPINE1 | 5054 | 42,872.221 | -0.518 | 0.105 | -4,920 | 0.0000 | 0.0000 | NA |
| ENSG00000115556 | PLCD4 | 84812 | 217,707 | -0.518 | 0.175 | -2,956 | 0.0031 | 0.0388 | NA |
| ENSG00000142039 | CCDC97 | 90324 | 1,378.386 | -0.518 | 0.113 | -4,582 | 0.0000 | 0.0002 | 99 |
| ENSG00000123892 | RAB38 | 23682 | 217,492 | -0.518 | 0.200 | -2,591 | 0.0096 | 0.0884 | 92 |
| ENSG00000165195 | PIGA | 5277 | 176,818 | -0.518 | 0.204 | -2,537 | 0.0112 | 0.0988 | 81 |
| ENSG00000203760 | CENPW | 387103 | 568,208 | -0.517 | 0.128 | -4,044 | 0.0001 | 0.0014 | NA |
| ENSG00000088325 | TPX2 | 22974 | 4,667.916 | -0.517 | 0.139 | -3,728 | 0.0002 | 0.0041 | NA |
| ENSG00000176903 | PNMA1 | 9240 | 2,519.800 | -0.516 | 0.100 | -5,152 | 0.0000 | 0.0000 | NA |
| ENSG00000107854 | TNKS2 | 80351 | 2,139.277 | -0.516 | 0.109 | -4,730 | 0.0000 | 0.0001 | NA |
| ENSG00000175324 | LSM1 | 27257 | 529,690 | -0.515 | 0.132 | -3,913 | 0.0001 | 0.0022 | NA |
| ENSG00000105750 | ZNF85 | 7639 | 207,666 | -0.515 | 0.194 | -2,659 | 0.0078 | 0.0775 | NA |
| ENSG00000119787 | ATL2 | 64225 | 798,887 | -0.515 | 0.155 | -3,328 | 0.0009 | 0.0141 | 82 |
| ENSG00000134575 | ACP2 | 53 | 2,058.362 | -0.514 | 0.129 | -3,974 | 0.0001 | 0.0018 | NA |
| ENSG00000117724 | CENPF | 1063 | 3,185.448 | -0.514 | 0.181 | -2,843 | 0.0045 | 0.0511 | NA |
| ENSG00000075702 | WDR62 | 284403 | 1,152.399 | -0.513 | 0.130 | -3,928 | 0.0001 | 0.0021 | NA |
| ENSG00000129675 | ARHGEF6 | 9459 | 393,784 | -0.513 | 0.153 | -3,358 | 0.0008 | 0.0130 | 92 |
| ENSG00000138735 | PDE5A | 8654 | 984,843 | -0.512 | 0.127 | -4,020 | 0.0001 | 0.0015 | 72 |
| ENSG00000134222 | PSRC1 | 84722 | 872,699 | -0.511 | 0.164 | -3,106 | 0.0019 | 0.0262 | NA |
| ENSG00000013810 | TACC3 | 10460 | 3,646.262 | -0.508 | 0.127 | -4,007 | 0.0001 | 0.0016 | NA |
| ENSG00000150347 | ARID5B | 84159 | 1,620.569 | -0.508 | 0.111 | -4,578 | 0.0000 | 0.0002 | 87 |
| ENSG00000184743 | ATL3 | 25923 | 3,504.194 | -0.507 | 0.136 | -3,730 | 0.0002 | 0.0041 | NA |
| ENSG00000147955 | SIGMAR1 | 10280 | 4,911.211 | -0.506 | 0.100 | -5,067 | 0.0000 | 0.0000 | NA |
| ENSG00000169169 | CPT1C | 126129 | 613,205 | -0.505 | 0.161 | -3,142 | 0.0017 | 0.0239 | NA |
| ENSG00000112742 | TTK | 7272 | 702,349 | -0.503 | 0.184 | -2,740 | 0.0061 | 0.0650 | NA |
| ENSG00000198162 | MAN1A2 | 10905 | 1,948.577 | -0.501 | 0.139 | -3,603 | 0.0003 | 0.0061 | 93 |
| ENSG00000112419 | PHACTR2 | 9749 | 1,484.575 | -0.501 | 0.114 | -4,389 | 0.0000 | 0.0004 | 89 |
| ENSG00000134574 | DDB2 | 1643 | 1,480.875 | -0.500 | 0.119 | -4,189 | 0.0000 | 0.0008 | NA |
| ENSG00000163734 | CXCL3 | 2921 | 879,047 | -0.499 | 0.196 | -2,543 | 0.0110 | 0.0978 | NA |
| ENSG00000276600 | RAB7B | 338382 | 890,433 | -0.497 | 0.105 | -4,727 | 0.0000 | 0.0001 | NA |
| ENSG00000127824 | TUBA4A | 7277 | 1,819.090 | -0.497 | 0.115 | -4,312 | 0.0000 | 0.0005 | NA |
| ENSG00000188312 | CENPP | 401541 | 178,424 | -0.496 | 0.192 | -2,580 | 0.0099 | 0.0899 | NA |
| ENSG00000205181 | LINC00654 | 149837 | 293,335 | -0.495 | 0.139 | -3,553 | 0.0004 | 0.0071 | NA |
| ENSG00000187266 | EPOR | 2057 | 336,407 | -0.495 | 0.158 | -3,129 | 0.0018 | 0.0247 | NA |
| ENSG00000074590 | NUAK1 | 9891 | 304,081 | -0.493 | 0.141 | -3,494 | 0.0005 | 0.0085 | NA |
| ENSG00000102385 | DRP2 | 1821 | 219,276 | -0.493 | 0.167 | -2,960 | 0.0031 | 0.0385 | NA |
| ENSG00000261730 |  |  | 158,723 | -0.493 | 0.181 | -2,720 | 0.0065 | 0.0677 | NA |
| ENSG00000158859 | ADAMTS4 | 9507 | 443,290 | -0.491 | 0.133 | -3,677 | 0.0002 | 0.0049 | NA |
| ENSG00000176148 | TCP11L1 | 55346 | 859,211 | -0.490 | 0.122 | -3,998 | 0.0001 | 0.0016 | NA |
| ENSG00000143633 | C1orf131 | 128061 | 252,231 | -0.490 | 0.158 | -3,109 | 0.0019 | 0.0260 | NA |
| ENSG00000131943 | C19orf12 | 83636 | 1,139.931 | -0.489 | 0.120 | -4,083 | 0.0000 | 0.0012 | NA |
| ENSG00000198826 | ARHGAP11A | 9824 | 2,315.179 | -0.488 | 0.133 | -3,664 | 0.0002 | 0.0051 | NA |
| ENSG00000100023 | PPIL2 | 23759 | 1,608.697 | -0.487 | 0.095 | -5,115 | 0.0000 | 0.0000 | NA |
| ENSG00000103275 | UBE2I | 7329 | 2,078.285 | -0.485 | 0.082 | -5,888 | 0.0000 | 0.0000 | 88 |
| ENSG00000162745 | OLFML2B | 25903 | 3,276.823 | -0.484 | 0.121 | -3,984 | 0.0001 | 0.0017 | NA |
| ENSG00000164023 | SGMS2 | 166929 | 1,136.029 | -0.483 | 0.108 | -4,487 | 0.0000 | 0.0003 | NA |
| ENSG00000120899 | PTK2B | 2185 | 559,218 | -0.482 | 0.178 | -2,716 | 0.0066 | 0.0684 | NA |
| ENSG00000133193 | FAM104A | 84923 | 643,515 | -0.482 | 0.110 | -4,371 | 0.0000 | 0.0004 | 54 |
| ENSG00000179163 | FUCA1 | 2517 | 2,176.895 | -0.482 | 0.114 | -4,249 | 0.0000 | 0.0007 | 54 |
| ENSG00000108604 | SMARCD2 | 6603 | 2,180.251 | -0.482 | 0.110 | -4,386 | 0.0000 | 0.0004 | 50 |
| ENSG00000198876 | DCAF12 | 25853 | 1,087.705 | -0.481 | 0.115 | -4,198 | 0.0000 | 0.0008 | NA |
| ENSG00000167703 | SLC43A2 | 124935 | 915,278 | -0.481 | 0.141 | -3,414 | 0.0006 | 0.0109 | NA |
| ENSG00000186340 | THBS2 | 7058 | 36,721.952 | -0.481 | 0.130 | -3,691 | 0.0002 | 0.0047 | 65 |
| ENSG00000112144 | CILK1 | 22858 | 679,565 | -0.480 | 0.139 | -3,448 | 0.0006 | 0.0099 | NA |
| ENSG00000123975 | CKS2 | 1164 | 2,255.166 | -0.479 | 0.131 | -3,670 | 0.0002 | 0.0050 | NA |
| ENSG00000010292 | NCAPD2 | 9918 | 3,446.058 | -0.478 | 0.125 | -3,825 | 0.0001 | 0.0030 | NA |
| ENSG00000171241 | SHCBP1 | 79801 | 2,251.559 | -0.478 | 0.141 | -3,382 | 0.0007 | 0.0120 | NA |
| ENSG00000100629 | CEP128 | 145508 | 221,004 | -0.477 | 0.178 | -2,675 | 0.0075 | 0.0752 | NA |
| ENSG00000165675 | ENOX2 | 10495 | 492,729 | -0.477 | 0.170 | -2,799 | 0.0051 | 0.0567 | 63 |
| ENSG00000089041 | P2RX7 | 5027 | 377,417 | -0.476 | 0.152 | -3,136 | 0.0017 | 0.0243 | NA |
| ENSG00000067191 | CACNB1 | 782 | 253,034 | -0.476 | 0.173 | -2,756 | 0.0059 | 0.0627 | NA |
| ENSG00000123268 | ATF1 | 466 | 520,241 | -0.476 | 0.153 | -3,119 | 0.0018 | 0.0254 | 82 |
| ENSG00000118418 | HMGN3 | 9324 | 501,240 | -0.475 | 0.132 | -3,613 | 0.0003 | 0.0059 | NA |
| ENSG00000198795 | ZNF521 | 25925 | 1,282.517 | -0.475 | 0.119 | -3,999 | 0.0001 | 0.0016 | 91 |
| ENSG00000164603 | BMT2 | 154743 | 276,558 | -0.475 | 0.186 | -2,554 | 0.0106 | 0.0954 | 86 |
| ENSG00000105556 | MIER2 | 54531 | 1,097.102 | -0.473 | 0.116 | -4,087 | 0.0000 | 0.0012 | 82 |
| ENSG00000183044 | ABAT | 18 | 197,353 | -0.472 | 0.170 | -2,770 | 0.0056 | 0.0606 | NA |
| ENSG00000133997 | MED6 | 10001 | 496,947 | -0.471 | 0.140 | -3,355 | 0.0008 | 0.0131 | NA |
| ENSG00000104765 | BNIP3L | 665 | 2,474.451 | -0.471 | 0.121 | -3,903 | 0.0001 | 0.0023 | 94 |
| ENSG00000109670 | FBXW7 | 55294 | 524,884 | -0.469 | 0.123 | -3,806 | 0.0001 | 0.0032 | NA |
| ENSG00000109805 | NCAPG | 64151 | 1,454.375 | -0.469 | 0.162 | -2,901 | 0.0037 | 0.0445 | NA |
| ENSG00000174370 | C11orf45 | 219833 | 217,703 | -0.468 | 0.168 | -2,791 | 0.0052 | 0.0577 | NA |
| ENSG00000187951 | LOC100288637 | 1E+08 | 339,242 | -0.468 | 0.171 | -2,730 | 0.0063 | 0.0663 | NA |
| ENSG00000173599 | PC | 5091 | 607,239 | -0.467 | 0.128 | -3,641 | 0.0003 | 0.0054 | NA |
| ENSG00000012174 | MBTPS2 | 51360 | 1,294.959 | -0.467 | 0.115 | -4,061 | 0.0000 | 0.0013 | 91 |
| ENSG00000090238 | YPEL3 | 83719 | 452,663 | -0.466 | 0.128 | -3,654 | 0.0003 | 0.0052 | NA |
| ENSG00000144524 | COPS7B | 64708 | 1,088.096 | -0.465 | 0.119 | -3,905 | 0.0001 | 0.0022 | NA |
| ENSG00000188042 | ARL4C | 10123 | 6,111.917 | -0.465 | 0.119 | -3,902 | 0.0001 | 0.0023 | NA |
| ENSG00000011332 | DPF1 | 8193 | 147,318 | -0.465 | 0.181 | -2,574 | 0.0101 | 0.0910 | NA |
| ENSG00000108306 | FBXL20 | 84961 | 362,974 | -0.465 | 0.157 | -2,961 | 0.0031 | 0.0384 | 91 |
| ENSG00000068912 | ERLEC1 | 27248 | 3,075.514 | -0.464 | 0.106 | -4,395 | 0.0000 | 0.0004 | NA |
| ENSG00000100526 | CDKN3 | 1033 | 804,401 | -0.463 | 0.151 | -3,072 | 0.0021 | 0.0286 | NA |
| ENSG00000198833 | UBE2J1 | 51465 | 2,186.857 | -0.463 | 0.102 | -4,558 | 0.0000 | 0.0002 | 98 |
| ENSG00000167992 | VWCE | 220001 | 506,345 | -0.462 | 0.126 | -3,665 | 0.0002 | 0.0050 | NA |
| ENSG00000156970 | BUB1B | 701 | 1,178.492 | -0.461 | 0.178 | -2,596 | 0.0094 | 0.0878 | NA |
| ENSG00000143801 | PSEN2 | 5664 | 1,605.666 | -0.460 | 0.109 | -4,214 | 0.0000 | 0.0008 | NA |
| ENSG00000198301 | SDAD1 | 55153 | 1,016.208 | -0.460 | 0.126 | -3,644 | 0.0003 | 0.0054 | 85 |
| ENSG00000140543 | DET1 | 55070 | 238,968 | -0.459 | 0.150 | -3,049 | 0.0023 | 0.0305 | 74 |
| ENSG00000020922 | MRE11 | 4361 | 530,971 | -0.458 | 0.163 | -2,806 | 0.0050 | 0.0559 | NA |
| ENSG00000149091 | DGKZ | 8525 | 3,130.924 | -0.458 | 0.114 | -4,032 | 0.0001 | 0.0014 | 65 |
| ENSG00000121691 | CAT | 847 | 1,880.800 | -0.457 | 0.102 | -4,483 | 0.0000 | 0.0003 | NA |
| ENSG00000152601 | MBNL1 | 4154 | 4,603.337 | -0.457 | 0.130 | -3,517 | 0.0004 | 0.0080 | 77 |
| ENSG00000087274 | ADD1 | 118 | 11,095.544 | -0.456 | 0.078 | -5,879 | 0.0000 | 0.0000 | NA |
| ENSG00000178999 | AURKB | 9212 | 1,092.432 | -0.456 | 0.151 | -3,013 | 0.0026 | 0.0333 | NA |
| ENSG00000138640 | FAM13A | 10144 | 552,851 | -0.456 | 0.145 | -3,140 | 0.0017 | 0.0240 | 82 |
| ENSG00000099814 | CEP170B | 283638 | 4,309.348 | -0.456 | 0.133 | -3,438 | 0.0006 | 0.0102 | 64 |
| ENSG00000057663 | ATG5 | 9474 | 409,361 | -0.455 | 0.149 | -3,060 | 0.0022 | 0.0296 | 89 |
| ENSG00000172765 | TMCC1 | 23023 | 522,175 | -0.455 | 0.126 | -3,617 | 0.0003 | 0.0058 | 81 |
| ENSG00000167604 | NFKBID | 84807 | 202,057 | -0.454 | 0.168 | -2,693 | 0.0071 | 0.0723 | NA |
| ENSG00000183763 | TRAIP | 10293 | 272,236 | -0.454 | 0.174 | -2,602 | 0.0093 | 0.0867 | NA |
| ENSG00000169679 | BUB1 | 699 | 2,058.860 | -0.453 | 0.144 | -3,150 | 0.0016 | 0.0234 | NA |
| ENSG00000090975 | PITPNM2 | 57605 | 1,085.600 | -0.453 | 0.132 | -3,445 | 0.0006 | 0.0100 | 68 |
| ENSG00000196781 | TLE1 | 7088 | 1,193.134 | -0.450 | 0.120 | -3,763 | 0.0002 | 0.0037 | NA |
| ENSG00000198176 | TFDP1 | 7027 | 5,432.901 | -0.450 | 0.103 | -4,356 | 0.0000 | 0.0004 | 90 |
| ENSG00000144320 | LNPK | 80856 | 1,651.561 | -0.450 | 0.118 | -3,821 | 0.0001 | 0.0030 | 75 |
| ENSG00000168765 | GSTM4 | 2948 | 519,515 | -0.449 | 0.145 | -3,107 | 0.0019 | 0.0261 | NA |
| ENSG00000087903 | RFX2 | 5990 | 278,329 | -0.449 | 0.158 | -2,846 | 0.0044 | 0.0508 | 65 |
| ENSG00000167842 | MIS12 | 79003 | 353,927 | -0.449 | 0.142 | -3,163 | 0.0016 | 0.0226 | 54 |
| ENSG00000258289 | CHURC1 | 91612 | 1,425.163 | -0.448 | 0.117 | -3,831 | 0.0001 | 0.0029 | NA |
| ENSG00000170734 | POLH | 5429 | 1,196.786 | -0.448 | 0.129 | -3,489 | 0.0005 | 0.0087 | NA |
| ENSG00000048991 | R3HDM1 | 23518 | 1,237.060 | -0.448 | 0.130 | -3,436 | 0.0006 | 0.0103 | 94 |
| ENSG00000029993 | HMGB3 | 3149 | 1,135.220 | -0.448 | 0.134 | -3,336 | 0.0008 | 0.0139 | 62 |
| ENSG00000128944 | KNSTRN | 90417 | 1,217.057 | -0.447 | 0.148 | -3,029 | 0.0025 | 0.0321 | NA |
| ENSG00000167202 | TBC1D2B | 23102 | 2,189.784 | -0.447 | 0.085 | -5,267 | 0.0000 | 0.0000 | 83 |
| ENSG00000139719 | VPS33A | 65082 | 473,648 | -0.446 | 0.137 | -3,261 | 0.0011 | 0.0171 | NA |
| ENSG00000204219 | TCEA3 | 6920 | 249,204 | -0.446 | 0.143 | -3,113 | 0.0019 | 0.0258 | NA |
| ENSG00000133069 | TMCC2 | 9911 | 151,880 | -0.446 | 0.171 | -2,611 | 0.0090 | 0.0851 | NA |
| ENSG00000152223 | EPG5 | 57724 | 4,598.231 | -0.446 | 0.124 | -3,589 | 0.0003 | 0.0064 | 91 |
| ENSG00000108448 | TRIM16L | 147166 | 660,826 | -0.445 | 0.119 | -3,743 | 0.0002 | 0.0039 | NA |
| ENSG00000116991 | SIPA1L2 | 57568 | 1,515.588 | -0.444 | 0.115 | -3,850 | 0.0001 | 0.0027 | NA |
| ENSG00000135775 | COG2 | 22796 | 658,576 | -0.444 | 0.117 | -3,803 | 0.0001 | 0.0032 | NA |
| ENSG00000164611 | PTTG1 | 9232 | 1,911.357 | -0.444 | 0.127 | -3,506 | 0.0005 | 0.0083 | NA |
| ENSG00000077943 | ITGA8 | 8516 | 1,985.264 | -0.444 | 0.150 | -2,958 | 0.0031 | 0.0386 | 85 |
| ENSG00000124181 | PLCG1 | 5335 | 3,229.977 | -0.444 | 0.095 | -4,672 | 0.0000 | 0.0001 | 64 |
| ENSG00000214941 | ZSWIM7 | 125150 | 260,926 | -0.443 | 0.153 | -2,899 | 0.0037 | 0.0446 | NA |
| ENSG00000064687 | ABCA7 | 10347 | 192,443 | -0.443 | 0.169 | -2,627 | 0.0086 | 0.0825 | NA |
| ENSG00000110218 | PANX1 | 24145 | 3,306.603 | -0.442 | 0.140 | -3,150 | 0.0016 | 0.0234 | NA |
| ENSG00000104313 | EYA1 | 2138 | 639,259 | -0.442 | 0.171 | -2,587 | 0.0097 | 0.0889 | NA |
| ENSG00000159388 | BTG2 | 7832 | 775,011 | -0.441 | 0.145 | -3,039 | 0.0024 | 0.0312 | NA |
| ENSG00000163923 | RPL39L | 116832 | 366,318 | -0.439 | 0.144 | -3,045 | 0.0023 | 0.0307 | NA |
| ENSG00000100221 | JOSD1 | 9929 | 2,606.771 | -0.439 | 0.089 | -4,915 | 0.0000 | 0.0000 | 88 |
| ENSG00000198954 | KIFBP | 26128 | 1,571.203 | -0.438 | 0.106 | -4,113 | 0.0000 | 0.0011 | NA |
| ENSG00000023445 | BIRC3 | 330 | 2,438.803 | -0.438 | 0.154 | -2,852 | 0.0043 | 0.0501 | NA |
| ENSG00000138107 | ACTR1A | 10121 | 7,620.947 | -0.438 | 0.077 | -5,667 | 0.0000 | 0.0000 | 92 |
| ENSG00000183337 | BCOR | 54880 | 1,087.355 | -0.436 | 0.105 | -4,160 | 0.0000 | 0.0009 | 89 |
| ENSG00000176624 | MEX3C | 51320 | 1,122.015 | -0.436 | 0.109 | -3,986 | 0.0001 | 0.0017 | 84 |
| ENSG00000164087 | POC1A | 25886 | 841,692 | -0.434 | 0.113 | -3,837 | 0.0001 | 0.0029 | NA |
| ENSG00000124216 | SNAI1 | 6615 | 246,204 | -0.434 | 0.166 | -2,622 | 0.0087 | 0.0836 | 88 |
| ENSG00000237649 | KIFC1 | 3833 | 2,158.671 | -0.433 | 0.163 | -2,659 | 0.0078 | 0.0777 | NA |
| ENSG00000164466 | SFXN1 | 94081 | 2,265.091 | -0.432 | 0.112 | -3,874 | 0.0001 | 0.0025 | 51 |
| ENSG00000186185 | KIF18B | 146909 | 1,222.444 | -0.431 | 0.146 | -2,947 | 0.0032 | 0.0398 | NA |
| ENSG00000156265 | MAP3K7CL | 56911 | 258,011 | -0.430 | 0.165 | -2,603 | 0.0092 | 0.0864 | NA |
| ENSG00000126581 | BECN1 | 8678 | 2,010.264 | -0.430 | 0.112 | -3,828 | 0.0001 | 0.0029 | 85 |
| ENSG00000109689 | STIM2 | 57620 | 795,237 | -0.429 | 0.135 | -3,180 | 0.0015 | 0.0216 | 97 |
| ENSG00000150961 | SEC24D | 9871 | 6,869.923 | -0.429 | 0.085 | -5,042 | 0.0000 | 0.0000 | 60 |
| ENSG00000009954 | BAZ1B | 9031 | 4,808.768 | -0.428 | 0.094 | -4,565 | 0.0000 | 0.0002 | NA |
| ENSG00000175040 | CHST2 | 9435 | 3,123.240 | -0.428 | 0.122 | -3,525 | 0.0004 | 0.0078 | 96 |
| ENSG00000181220 | ZNF746 | 155061 | 1,158.724 | -0.428 | 0.115 | -3,713 | 0.0002 | 0.0043 | 66 |
| ENSG00000116001 | TIA1 | 7072 | 870,230 | -0.428 | 0.120 | -3,569 | 0.0004 | 0.0068 | 53 |
| ENSG00000091651 | ORC6 | 23594 | 767,315 | -0.426 | 0.138 | -3,076 | 0.0021 | 0.0283 | NA |
| ENSG00000140199 | SLC12A6 | 9990 | 1,240.269 | -0.426 | 0.129 | -3,297 | 0.0010 | 0.0154 | 96 |
| ENSG00000163900 | TMEM41A | 90407 | 692,384 | -0.424 | 0.102 | -4,163 | 0.0000 | 0.0009 | NA |
| ENSG00000164615 | CAMLG | 819 | 882,949 | -0.424 | 0.104 | -4,080 | 0.0000 | 0.0012 | NA |
| ENSG00000148300 | REXO4 | 57109 | 1,121.097 | -0.424 | 0.114 | -3,738 | 0.0002 | 0.0040 | NA |
| ENSG00000165533 | TTC8 | 123016 | 824,813 | -0.424 | 0.117 | -3,614 | 0.0003 | 0.0059 | NA |
| ENSG00000131370 | SH3BP5 | 9467 | 297,830 | -0.424 | 0.163 | -2,610 | 0.0091 | 0.0854 | NA |
| ENSG00000143416 | SELENBP1 | 8991 | 569,341 | -0.423 | 0.133 | -3,186 | 0.0014 | 0.0213 | NA |
| ENSG00000004660 | CAMKK1 | 84254 | 396,381 | -0.422 | 0.129 | -3,283 | 0.0010 | 0.0160 | NA |
| ENSG00000172731 | LRRC20 | 55222 | 326,493 | -0.419 | 0.139 | -3,016 | 0.0026 | 0.0331 | NA |
| ENSG00000011143 | MKS1 | 54903 | 232,322 | -0.419 | 0.151 | -2,767 | 0.0056 | 0.0609 | NA |
| ENSG00000171992 | SYNPO | 11346 | 4,642.155 | -0.417 | 0.134 | -3,106 | 0.0019 | 0.0262 | NA |
| ENSG00000140022 | STON2 | 85439 | 429,490 | -0.417 | 0.144 | -2,889 | 0.0039 | 0.0457 | NA |
| ENSG00000133110 | POSTN | 10631 | 8,949.345 | -0.416 | 0.113 | -3,677 | 0.0002 | 0.0049 | NA |
| ENSG00000108395 | TRIM37 | 4591 | 1,347.773 | -0.416 | 0.114 | -3,651 | 0.0003 | 0.0053 | NA |
| ENSG00000156374 | PCGF6 | 84108 | 291,987 | -0.416 | 0.155 | -2,683 | 0.0073 | 0.0741 | NA |
| ENSG00000273015 |  |  | 192,005 | -0.415 | 0.156 | -2,663 | 0.0077 | 0.0770 | NA |
| ENSG00000182093 | GET1 | 7485 | 676,330 | -0.415 | 0.159 | -2,609 | 0.0091 | 0.0855 | NA |
| ENSG00000171604 | CXXC5 | 51523 | 1,412.438 | -0.414 | 0.116 | -3,578 | 0.0003 | 0.0066 | NA |
| ENSG00000064419 | TNPO3 | 23534 | 3,439.337 | -0.414 | 0.098 | -4,219 | 0.0000 | 0.0007 | 79 |
| ENSG00000090889 | KIF4A | 24137 | 1,395.334 | -0.413 | 0.132 | -3,140 | 0.0017 | 0.0240 | NA |
| ENSG00000111727 | HCFC2 | 29915 | 599,523 | -0.413 | 0.116 | -3,567 | 0.0004 | 0.0068 | 98 |
| ENSG00000141985 | SH3GL1 | 6455 | 4,781.178 | -0.412 | 0.121 | -3,415 | 0.0006 | 0.0109 | NA |
| ENSG00000136280 | CCM2 | 83605 | 2,340.496 | -0.412 | 0.124 | -3,331 | 0.0009 | 0.0140 | NA |
| ENSG00000135698 | MPHOSPH6 | 10200 | 1,054.478 | -0.412 | 0.145 | -2,836 | 0.0046 | 0.0519 | NA |
| ENSG00000169118 | CSNK1G1 | 53944 | 955,466 | -0.411 | 0.128 | -3,209 | 0.0013 | 0.0200 | 88 |
| ENSG00000105700 | KXD1 | 79036 | 1,788.980 | -0.411 | 0.104 | -3,936 | 0.0001 | 0.0020 | 83 |
| ENSG00000126216 | TUBGCP3 | 10426 | 831,342 | -0.411 | 0.105 | -3,910 | 0.0001 | 0.0022 | 50 |
| ENSG00000088543 | C3orf18 | 51161 | 1,680.918 | -0.409 | 0.117 | -3,486 | 0.0005 | 0.0088 | NA |
| ENSG00000149503 | INCENP | 3619 | 1,680.145 | -0.409 | 0.118 | -3,466 | 0.0005 | 0.0094 | NA |
| ENSG00000185298 | CCDC137 | 339230 | 1,510.287 | -0.408 | 0.112 | -3,655 | 0.0003 | 0.0052 | NA |
| ENSG00000058272 | PPP1R12A | 4659 | 2,560.173 | -0.408 | 0.121 | -3,359 | 0.0008 | 0.0129 | 90 |
| ENSG00000114019 | AMOTL2 | 51421 | 5,672.489 | -0.408 | 0.149 | -2,735 | 0.0062 | 0.0656 | 76 |
| ENSG00000079616 | KIF22 | 3835 | 1,490.752 | -0.406 | 0.108 | -3,769 | 0.0002 | 0.0036 | NA |
| ENSG00000099308 | MAST3 | 23031 | 450,381 | -0.406 | 0.136 | -2,972 | 0.0030 | 0.0372 | 93 |
| ENSG00000163904 | SENP2 | 59343 | 1,327.219 | -0.404 | 0.105 | -3,864 | 0.0001 | 0.0026 | NA |
| ENSG00000204116 | CHIC1 | 53344 | 392,517 | -0.404 | 0.159 | -2,536 | 0.0112 | 0.0989 | 96 |
| ENSG00000138175 | ARL3 | 403 | 722,806 | -0.403 | 0.112 | -3,584 | 0.0003 | 0.0065 | NA |
| ENSG00000004700 | RECQL | 5965 | 1,652.081 | -0.403 | 0.140 | -2,877 | 0.0040 | 0.0471 | NA |
| ENSG00000276293 | PIP4K2B | 8396 | 3,873.027 | -0.403 | 0.090 | -4,468 | 0.0000 | 0.0003 | 89 |
| ENSG00000131748 | STARD3 | 10948 | 1,717.524 | -0.402 | 0.122 | -3,292 | 0.0010 | 0.0156 | NA |
| ENSG00000089685 | BIRC5 | 332 | 2,606.109 | -0.402 | 0.132 | -3,045 | 0.0023 | 0.0307 | NA |
| ENSG00000155545 | MIER3 | 166968 | 350,918 | -0.402 | 0.153 | -2,618 | 0.0089 | 0.0843 | 100 |
| ENSG00000124217 | MOCS3 | 27304 | 408,082 | -0.402 | 0.146 | -2,747 | 0.0060 | 0.0639 | 58 |
| ENSG00000137166 | FOXP4 | 116113 | 1,205.720 | -0.402 | 0.151 | -2,662 | 0.0078 | 0.0771 | 54 |
| ENSG00000163812 | ZDHHC3 | 51304 | 2,570.245 | -0.400 | 0.084 | -4,751 | 0.0000 | 0.0001 | NA |
| ENSG00000071051 | NCK2 | 8440 | 637,059 | -0.400 | 0.126 | -3,164 | 0.0016 | 0.0226 | 69 |
| ENSG00000162144 | CYB561A3 | 220002 | 2,780.253 | -0.399 | 0.105 | -3,801 | 0.0001 | 0.0032 | NA |
| ENSG00000185621 | LMLN | 89782 | 480,887 | -0.399 | 0.151 | -2,640 | 0.0083 | 0.0809 | 90 |
| ENSG00000116273 | PHF13 | 148479 | 950,335 | -0.397 | 0.101 | -3,923 | 0.0001 | 0.0021 | 92 |
| ENSG00000181744 | DIPK2A | 205428 | 645,849 | -0.397 | 0.143 | -2,769 | 0.0056 | 0.0607 | 85 |
| ENSG00000167552 | TUBA1A | 7846 | 15,956.692 | -0.396 | 0.076 | -5,198 | 0.0000 | 0.0000 | NA |
| ENSG00000171823 | FBXL14 | 144699 | 250,013 | -0.396 | 0.145 | -2,728 | 0.0064 | 0.0664 | 53 |
| ENSG00000186417 | GLDN | 342035 | 427,035 | -0.395 | 0.136 | -2,893 | 0.0038 | 0.0454 | NA |
| ENSG00000058056 | USP13 | 8975 | 1,139.923 | -0.393 | 0.138 | -2,849 | 0.0044 | 0.0505 | NA |
| ENSG00000092871 | RFFL | 117584 | 426,158 | -0.392 | 0.120 | -3,272 | 0.0011 | 0.0166 | NA |
| ENSG00000164330 | EBF1 | 1879 | 385,008 | -0.392 | 0.150 | -2,617 | 0.0089 | 0.0844 | NA |
| ENSG00000196155 | PLEKHG4 | 25894 | 2,229.358 | -0.391 | 0.133 | -2,939 | 0.0033 | 0.0404 | NA |
| ENSG00000139496 | NUP58 | 9818 | 1,918.871 | -0.390 | 0.127 | -3,072 | 0.0021 | 0.0286 | NA |
| ENSG00000165406 | MARCHF8 | 220972 | 1,165.657 | -0.388 | 0.102 | -3,789 | 0.0002 | 0.0033 | NA |
| ENSG00000160803 | UBQLN4 | 56893 | 1,724.517 | -0.388 | 0.105 | -3,687 | 0.0002 | 0.0047 | NA |
| ENSG00000221955 | SLC12A8 | 84561 | 1,438.541 | -0.388 | 0.131 | -2,956 | 0.0031 | 0.0388 | NA |
| ENSG00000198901 | PRC1 | 9055 | 3,367.657 | -0.388 | 0.132 | -2,927 | 0.0034 | 0.0415 | NA |
| ENSG00000134265 | NAPG | 8774 | 1,160.992 | -0.388 | 0.135 | -2,876 | 0.0040 | 0.0472 | 80 |
| ENSG00000117697 | NSL1 | 25936 | 521,396 | -0.388 | 0.126 | -3,078 | 0.0021 | 0.0283 | 61 |
| ENSG00000135622 | SEMA4F | 10505 | 1,048.196 | -0.387 | 0.094 | -4,112 | 0.0000 | 0.0011 | NA |
| ENSG00000133195 | SLC39A11 | 201266 | 819,940 | -0.387 | 0.105 | -3,676 | 0.0002 | 0.0049 | NA |
| ENSG00000148229 | POLE3 | 54107 | 1,660.656 | -0.387 | 0.127 | -3,048 | 0.0023 | 0.0305 | NA |
| ENSG00000224149 |  |  | 472,657 | -0.387 | 0.142 | -2,733 | 0.0063 | 0.0659 | NA |
| ENSG00000119139 | TJP2 | 9414 | 1,069.355 | -0.386 | 0.103 | -3,752 | 0.0002 | 0.0038 | NA |
| ENSG00000107643 | MAPK8 | 5599 | 639,953 | -0.386 | 0.146 | -2,643 | 0.0082 | 0.0803 | 83 |
| ENSG00000173207 | CKS1B | 1163 | 1,124.180 | -0.385 | 0.120 | -3,210 | 0.0013 | 0.0199 | NA |
| ENSG00000145882 | PCYOX1L | 78991 | 406,209 | -0.385 | 0.128 | -3,003 | 0.0027 | 0.0343 | NA |
| ENSG00000164535 | DAGLB | 221955 | 1,421.486 | -0.384 | 0.105 | -3,668 | 0.0002 | 0.0050 | NA |
| ENSG00000221926 | TRIM16 | 10626 | 685,734 | -0.384 | 0.137 | -2,806 | 0.0050 | 0.0559 | NA |
| ENSG00000161638 | ITGA5 | 3678 | 34,624.650 | -0.383 | 0.101 | -3,794 | 0.0001 | 0.0033 | NA |
| ENSG00000196642 | RABL6 | 55684 | 3,746.449 | -0.383 | 0.131 | -2,927 | 0.0034 | 0.0415 | NA |
| ENSG00000120318 | ARAP3 | 64411 | 297,591 | -0.382 | 0.151 | -2,530 | 0.0114 | 0.0998 | NA |
| ENSG00000184203 | PPP1R2 | 5504 | 863,516 | -0.382 | 0.112 | -3,417 | 0.0006 | 0.0108 | 97 |
| ENSG00000136636 | KCTD3 | 51133 | 1,585.540 | -0.382 | 0.114 | -3,346 | 0.0008 | 0.0134 | 71 |
| ENSG00000108106 | UBE2S | 27338 | 5,408.564 | -0.381 | 0.141 | -2,705 | 0.0068 | 0.0704 | NA |
| ENSG00000138162 | TACC2 | 10579 | 1,189.565 | -0.381 | 0.143 | -2,667 | 0.0076 | 0.0764 | NA |
| ENSG00000142230 | SAE1 | 10055 | 7,105.712 | -0.380 | 0.086 | -4,401 | 0.0000 | 0.0004 | NA |
| ENSG00000197302 | ZNF720 | 124411 | 377,811 | -0.380 | 0.132 | -2,871 | 0.0041 | 0.0477 | NA |
| ENSG00000081087 | OSTM1 | 28962 | 3,062.185 | -0.380 | 0.131 | -2,902 | 0.0037 | 0.0444 | 91 |
| ENSG00000151067 | CACNA1C | 775 | 1,761.750 | -0.380 | 0.103 | -3,681 | 0.0002 | 0.0048 | 59 |
| ENSG00000106460 | TMEM106B | 54664 | 1,126.680 | -0.379 | 0.121 | -3,137 | 0.0017 | 0.0242 | NA |
| ENSG00000143553 | SNAPIN | 23557 | 780,050 | -0.379 | 0.109 | -3,480 | 0.0005 | 0.0089 | 69 |
| ENSG00000127946 | HIP1 | 3092 | 990,202 | -0.378 | 0.119 | -3,184 | 0.0015 | 0.0213 | NA |
| ENSG00000156735 | BAG4 | 9530 | 571,433 | -0.378 | 0.135 | -2,793 | 0.0052 | 0.0575 | NA |
| ENSG00000118900 | UBN1 | 29855 | 3,192.460 | -0.378 | 0.092 | -4,126 | 0.0000 | 0.0010 | 93 |
| ENSG00000149679 | CABLES2 | 81928 | 534,726 | -0.377 | 0.126 | -2,993 | 0.0028 | 0.0352 | NA |
| ENSG00000166575 | TMEM135 | 65084 | 396,648 | -0.377 | 0.142 | -2,654 | 0.0080 | 0.0783 | NA |
| ENSG00000089177 | KIF16B | 55614 | 803,113 | -0.377 | 0.105 | -3,575 | 0.0004 | 0.0066 | 84 |
| ENSG00000056972 | TRAF3IP2 | 10758 | 2,033.447 | -0.376 | 0.102 | -3,700 | 0.0002 | 0.0045 | NA |
| ENSG00000162104 | ADCY9 | 115 | 3,168.298 | -0.376 | 0.106 | -3,541 | 0.0004 | 0.0074 | NA |
| ENSG00000145284 | SCD5 | 79966 | 419,915 | -0.376 | 0.128 | -2,950 | 0.0032 | 0.0394 | NA |
| ENSG00000161800 | RACGAP1 | 29127 | 3,207.551 | -0.376 | 0.138 | -2,718 | 0.0066 | 0.0680 | NA |
| ENSG00000101104 | PABPC1L | 80336 | 439,214 | -0.375 | 0.140 | -2,682 | 0.0073 | 0.0742 | NA |
| ENSG00000173226 | IQCB1 | 9657 | 350,895 | -0.375 | 0.129 | -2,898 | 0.0038 | 0.0447 | 84 |
| ENSG00000166272 | WBP1L | 54838 | 3,483.561 | -0.373 | 0.086 | -4,354 | 0.0000 | 0.0004 | NA |
| ENSG00000169925 | BRD3 | 8019 | 871,020 | -0.373 | 0.109 | -3,432 | 0.0006 | 0.0103 | NA |
| ENSG00000115840 | SLC25A12 | 8604 | 876,686 | -0.373 | 0.124 | -3,016 | 0.0026 | 0.0331 | NA |
| ENSG00000143641 | GALNT2 | 2590 | 11,484.111 | -0.373 | 0.094 | -3,982 | 0.0001 | 0.0017 | 88 |
| ENSG00000146966 | DENND2A | 27147 | 1,617.159 | -0.372 | 0.112 | -3,336 | 0.0009 | 0.0139 | NA |
| ENSG00000164506 | STXBP5 | 134957 | 1,605.934 | -0.372 | 0.135 | -2,766 | 0.0057 | 0.0610 | 98 |
| ENSG00000149557 | FEZ1 | 9638 | 1,596.170 | -0.370 | 0.095 | -3,899 | 0.0001 | 0.0023 | NA |
| ENSG00000069275 | NUCKS1 | 64710 | 6,786.345 | -0.369 | 0.131 | -2,808 | 0.0050 | 0.0557 | 87 |
| ENSG00000108797 | CNTNAP1 | 8506 | 2,742.402 | -0.368 | 0.109 | -3,371 | 0.0007 | 0.0125 | NA |
| ENSG00000196976 | LAGE3 | 8270 | 432,756 | -0.368 | 0.141 | -2,606 | 0.0092 | 0.0860 | NA |
| ENSG00000171608 | PIK3CD | 5293 | 1,878.421 | -0.367 | 0.105 | -3,507 | 0.0005 | 0.0082 | 93 |
| ENSG00000124641 | MED20 | 9477 | 671,813 | -0.366 | 0.114 | -3,206 | 0.0013 | 0.0201 | NA |
| ENSG00000196850 | PPTC7 | 160760 | 1,247.821 | -0.366 | 0.098 | -3,727 | 0.0002 | 0.0041 | 87 |
| ENSG00000197375 | SLC22A5 | 6584 | 312,391 | -0.366 | 0.139 | -2,631 | 0.0085 | 0.0822 | 78 |
| ENSG00000072864 | NDE1 | 54820 | 735,663 | -0.365 | 0.111 | -3,290 | 0.0010 | 0.0157 | NA |
| ENSG00000100099 | HPS4 | 89781 | 1,324.353 | -0.365 | 0.116 | -3,143 | 0.0017 | 0.0239 | NA |
| ENSG00000197535 | MYO5A | 4644 | 3,900.382 | -0.364 | 0.103 | -3,534 | 0.0004 | 0.0076 | 94 |
| ENSG00000205060 | SLC35B4 | 84912 | 1,607.825 | -0.364 | 0.113 | -3,217 | 0.0013 | 0.0195 | 84 |
| ENSG00000163964 | PIGX | 54965 | 559,126 | -0.363 | 0.128 | -2,832 | 0.0046 | 0.0525 | NA |
| ENSG00000139926 | FRMD6 | 122786 | 12,844.315 | -0.363 | 0.107 | -3,386 | 0.0007 | 0.0119 | 94 |
| ENSG00000188486 | H2AX | 3014 | 4,334.967 | -0.361 | 0.139 | -2,599 | 0.0093 | 0.0871 | NA |
| ENSG00000010017 | RANBP9 | 10048 | 1,977.825 | -0.360 | 0.109 | -3,290 | 0.0010 | 0.0157 | NA |
| ENSG00000011523 | CEP68 | 23177 | 465,809 | -0.360 | 0.118 | -3,047 | 0.0023 | 0.0306 | NA |
| ENSG00000167325 | RRM1 | 6240 | 3,950.561 | -0.358 | 0.123 | -2,918 | 0.0035 | 0.0425 | NA |
| ENSG00000164402 | SEPTIN8 | 23176 | 2,783.678 | -0.356 | 0.099 | -3,612 | 0.0003 | 0.0059 | NA |
| ENSG00000161692 | DBF4B | 80174 | 714,675 | -0.356 | 0.117 | -3,042 | 0.0024 | 0.0310 | NA |
| ENSG00000064961 | HMG20B | 10362 | 1,777.969 | -0.356 | 0.121 | -2,946 | 0.0032 | 0.0398 | NA |
| ENSG00000151500 | THYN1 | 29087 | 485,198 | -0.356 | 0.134 | -2,652 | 0.0080 | 0.0786 | NA |
| ENSG00000090686 | USP48 | 84196 | 1,703.381 | -0.355 | 0.110 | -3,229 | 0.0012 | 0.0188 | 88 |
| ENSG00000106546 | AHR | 196 | 2,489.836 | -0.354 | 0.116 | -3,048 | 0.0023 | 0.0305 | NA |
| ENSG00000176619 | LMNB2 | 84823 | 7,461.127 | -0.353 | 0.096 | -3,677 | 0.0002 | 0.0049 | NA |
| ENSG00000105270 | CLIP3 | 25999 | 2,360.722 | -0.353 | 0.107 | -3,289 | 0.0010 | 0.0157 | NA |
| ENSG00000100150 | DEPDC5 | 9681 | 422,367 | -0.353 | 0.119 | -2,961 | 0.0031 | 0.0384 | NA |
| ENSG00000107130 | NCS1 | 23413 | 5,821.118 | -0.352 | 0.097 | -3,618 | 0.0003 | 0.0058 | NA |
| ENSG00000035681 | NSMAF | 8439 | 2,428.030 | -0.352 | 0.127 | -2,781 | 0.0054 | 0.0591 | NA |
| ENSG00000172830 | SSH3 | 54961 | 1,093.229 | -0.352 | 0.139 | -2,535 | 0.0113 | 0.0989 | NA |
| ENSG00000205221 | VIT | 5212 | 1,213.301 | -0.351 | 0.128 | -2,738 | 0.0062 | 0.0651 | NA |
| ENSG00000170855 | TRIAP1 | 51499 | 927,673 | -0.351 | 0.138 | -2,537 | 0.0112 | 0.0988 | NA |
| ENSG00000087253 | LPCAT2 | 54947 | 698,187 | -0.351 | 0.111 | -3,175 | 0.0015 | 0.0220 | 72 |
| ENSG00000153786 | ZDHHC7 | 55625 | 5,329.401 | -0.350 | 0.100 | -3,506 | 0.0005 | 0.0083 | NA |
| ENSG00000115129 | TP53I3 | 9540 | 1,557.854 | -0.350 | 0.123 | -2,845 | 0.0044 | 0.0508 | NA |
| ENSG00000180346 | TIGD2 | 166815 | 359,594 | -0.350 | 0.136 | -2,563 | 0.0104 | 0.0935 | NA |
| ENSG00000171877 | FRMD5 | 84978 | 544,391 | -0.349 | 0.138 | -2,535 | 0.0112 | 0.0989 | NA |
| ENSG00000156026 | MCU | 90550 | 1,467.834 | -0.348 | 0.102 | -3,399 | 0.0007 | 0.0114 | NA |
| ENSG00000131725 | WDR44 | 54521 | 869,672 | -0.346 | 0.117 | -2,960 | 0.0031 | 0.0385 | 86 |
| ENSG00000179981 | TSHZ1 | 10194 | 436,037 | -0.345 | 0.127 | -2,722 | 0.0065 | 0.0673 | NA |
| ENSG00000172728 | FUT10 | 84750 | 516,412 | -0.344 | 0.134 | -2,567 | 0.0103 | 0.0926 | NA |
| ENSG00000137492 | THAP12 | 5612 | 1,259.212 | -0.344 | 0.128 | -2,695 | 0.0070 | 0.0719 | 82 |
| ENSG00000153774 | CFDP1 | 10428 | 465,488 | -0.343 | 0.132 | -2,591 | 0.0096 | 0.0884 | 71 |
| ENSG00000111206 | FOXM1 | 2305 | 3,048.358 | -0.342 | 0.098 | -3,501 | 0.0005 | 0.0084 | NA |
| ENSG00000078804 | TP53INP2 | 58476 | 3,251.234 | -0.342 | 0.105 | -3,242 | 0.0012 | 0.0181 | NA |
| ENSG00000137500 | CCDC90B | 60492 | 1,084.476 | -0.342 | 0.114 | -2,997 | 0.0027 | 0.0349 | NA |
| ENSG00000160953 | PWWP3A | 84939 | 877,476 | -0.341 | 0.107 | -3,182 | 0.0015 | 0.0215 | NA |
| ENSG00000155755 | TMEM237 | 65062 | 827,552 | -0.341 | 0.109 | -3,125 | 0.0018 | 0.0250 | NA |
| ENSG00000151553 | FAM160B1 | 57700 | 1,416.248 | -0.341 | 0.103 | -3,304 | 0.0010 | 0.0151 | 97 |
| ENSG00000087077 | TRIP6 | 7205 | 6,848.518 | -0.340 | 0.128 | -2,656 | 0.0079 | 0.0781 | NA |
| ENSG00000115875 | SRSF7 | 6432 | 2,620.650 | -0.340 | 0.101 | -3,377 | 0.0007 | 0.0122 | 96 |
| ENSG00000133059 | DSTYK | 25778 | 1,521.478 | -0.340 | 0.113 | -3,010 | 0.0026 | 0.0336 | 88 |
| ENSG00000177239 | MAN1B1 | 11253 | 5,451.449 | -0.340 | 0.115 | -2,946 | 0.0032 | 0.0398 | 64 |
| ENSG00000128656 | CHN1 | 1123 | 1,783.971 | -0.339 | 0.099 | -3,419 | 0.0006 | 0.0108 | NA |
| ENSG00000108599 | AKAP10 | 11216 | 987,563 | -0.339 | 0.110 | -3,077 | 0.0021 | 0.0283 | 62 |
| ENSG00000104889 | RNASEH2A | 10535 | 951,396 | -0.338 | 0.121 | -2,803 | 0.0051 | 0.0562 | NA |
| ENSG00000130338 | TULP4 | 56995 | 911,216 | -0.338 | 0.098 | -3,457 | 0.0005 | 0.0096 | 87 |
| ENSG00000110619 | CARS1 | 833 | 2,093.621 | -0.337 | 0.101 | -3,332 | 0.0009 | 0.0140 | NA |
| ENSG00000135924 | DNAJB2 | 3300 | 2,556.538 | -0.337 | 0.115 | -2,931 | 0.0034 | 0.0412 | NA |
| ENSG00000172943 | PHF8 | 23133 | 798,722 | -0.337 | 0.117 | -2,870 | 0.0041 | 0.0477 | NA |
| ENSG00000241839 | PLEKHO2 | 80301 | 3,305.322 | -0.337 | 0.116 | -2,894 | 0.0038 | 0.0453 | 96 |
| ENSG00000140382 | HMG20A | 10363 | 813,716 | -0.335 | 0.110 | -3,056 | 0.0022 | 0.0299 | NA |
| ENSG00000170633 | RNF34 | 80196 | 1,064.816 | -0.334 | 0.096 | -3,484 | 0.0005 | 0.0088 | NA |
| ENSG00000155760 | FZD7 | 8324 | 4,903.765 | -0.334 | 0.114 | -2,924 | 0.0035 | 0.0418 | NA |
| ENSG00000196083 | IL1RAP | 3556 | 500,753 | -0.334 | 0.125 | -2,672 | 0.0076 | 0.0758 | NA |
| ENSG00000082898 | XPO1 | 7514 | 5,166.191 | -0.334 | 0.116 | -2,872 | 0.0041 | 0.0477 | 99 |
| ENSG00000085721 | RRN3 | 54700 | 1,596.872 | -0.332 | 0.110 | -3,022 | 0.0025 | 0.0326 | 78 |
| ENSG00000074266 | EED | 8726 | 541,268 | -0.331 | 0.126 | -2,628 | 0.0086 | 0.0825 | 99 |
| ENSG00000146830 | GIGYF1 | 64599 | 2,180.973 | -0.331 | 0.113 | -2,929 | 0.0034 | 0.0414 | 78 |
| ENSG00000172840 | PDP2 | 57546 | 635,358 | -0.330 | 0.119 | -2,785 | 0.0054 | 0.0586 | NA |
| ENSG00000135424 | ITGA7 | 3679 | 1,317.628 | -0.330 | 0.130 | -2,535 | 0.0112 | 0.0989 | NA |
| ENSG00000008130 | NADK | 65220 | 2,869.924 | -0.330 | 0.124 | -2,664 | 0.0077 | 0.0769 | 93 |
| ENSG00000137845 | ADAM10 | 102 | 6,030.945 | -0.330 | 0.105 | -3,132 | 0.0017 | 0.0246 | 56 |
| ENSG00000137404 | NRM | 11270 | 893,674 | -0.329 | 0.105 | -3,130 | 0.0017 | 0.0246 | NA |
| ENSG00000136854 | STXBP1 | 6812 | 2,764.483 | -0.328 | 0.095 | -3,461 | 0.0005 | 0.0095 | NA |
| ENSG00000112249 | ASCC3 | 10973 | 3,148.047 | -0.327 | 0.125 | -2,616 | 0.0089 | 0.0845 | 79 |
| ENSG00000114416 | FXR1 | 8087 | 3,234.959 | -0.327 | 0.103 | -3,174 | 0.0015 | 0.0220 | 77 |
| ENSG00000187837 | H1-2 | 3006 | 2,020.974 | -0.326 | 0.102 | -3,186 | 0.0014 | 0.0213 | NA |
| ENSG00000196455 | PIK3R4 | 30849 | 1,023.693 | -0.326 | 0.124 | -2,629 | 0.0086 | 0.0824 | NA |
| ENSG00000114331 | ACAP2 | 23527 | 1,906.190 | -0.325 | 0.119 | -2,738 | 0.0062 | 0.0651 | 58 |
| ENSG00000011114 | BTBD7 | 55727 | 1,108.642 | -0.323 | 0.125 | -2,592 | 0.0096 | 0.0884 | NA |
| ENSG00000198648 | STK39 | 27347 | 1,616.402 | -0.323 | 0.120 | -2,696 | 0.0070 | 0.0719 | 97 |
| ENSG00000064393 | HIPK2 | 28996 | 6,830.503 | -0.323 | 0.105 | -3,080 | 0.0021 | 0.0281 | 89 |
| ENSG00000006042 | TMEM98 | 26022 | 1,578.507 | -0.320 | 0.092 | -3,483 | 0.0005 | 0.0088 | NA |
| ENSG00000100439 | ABHD4 | 63874 | 1,880.716 | -0.320 | 0.108 | -2,965 | 0.0030 | 0.0380 | NA |
| ENSG00000176974 | SHMT1 | 6470 | 776,901 | -0.320 | 0.115 | -2,787 | 0.0053 | 0.0583 | NA |
| ENSG00000204392 | LSM2 | 57819 | 696,755 | -0.320 | 0.115 | -2,787 | 0.0053 | 0.0584 | NA |
| ENSG00000275835 | TUBGCP5 | 114791 | 926,272 | -0.319 | 0.124 | -2,580 | 0.0099 | 0.0899 | NA |
| ENSG00000102763 | VWA8 | 23078 | 812,781 | -0.319 | 0.125 | -2,541 | 0.0110 | 0.0980 | NA |
| ENSG00000196504 | PRPF40A | 55660 | 3,149.955 | -0.318 | 0.119 | -2,663 | 0.0077 | 0.0770 | 82 |
| ENSG00000102753 | KPNA3 | 3839 | 2,237.055 | -0.318 | 0.123 | -2,590 | 0.0096 | 0.0884 | 75 |
| ENSG00000175866 | BAIAP2 | 10458 | 1,728.840 | -0.317 | 0.108 | -2,953 | 0.0031 | 0.0391 | NA |
| ENSG00000198807 | PAX9 | 5083 | 3,436.296 | -0.317 | 0.105 | -3,014 | 0.0026 | 0.0333 | 80 |
| ENSG00000130956 | HABP4 | 22927 | 1,926.085 | -0.316 | 0.118 | -2,680 | 0.0074 | 0.0744 | NA |
| ENSG00000081721 | DUSP12 | 11266 | 675,059 | -0.314 | 0.107 | -2,941 | 0.0033 | 0.0402 | NA |
| ENSG00000108523 | RNF167 | 26001 | 3,958.615 | -0.313 | 0.086 | -3,650 | 0.0003 | 0.0053 | NA |
| ENSG00000134283 | PPHLN1 | 51535 | 1,634.293 | -0.313 | 0.109 | -2,874 | 0.0041 | 0.0475 | NA |
| ENSG00000166387 | PPFIBP2 | 8495 | 817,784 | -0.313 | 0.117 | -2,667 | 0.0077 | 0.0765 | NA |
| ENSG00000096092 | TMEM14A | 28978 | 595,633 | -0.313 | 0.123 | -2,547 | 0.0109 | 0.0968 | NA |
| ENSG00000126778 | SIX1 | 6495 | 2,063.252 | -0.312 | 0.116 | -2,689 | 0.0072 | 0.0729 | 95 |
| ENSG00000196715 | VKORC1L1 | 154807 | 1,813.581 | -0.310 | 0.116 | -2,660 | 0.0078 | 0.0775 | 84 |
| ENSG00000182667 | NTM | 50863 | 2,356.373 | -0.310 | 0.085 | -3,659 | 0.0003 | 0.0051 | 51 |
| ENSG00000115935 | WIPF1 | 7456 | 1,432.934 | -0.309 | 0.103 | -2,993 | 0.0028 | 0.0352 | 85 |
| ENSG00000105355 | PLIN3 | 10226 | 10,734.773 | -0.309 | 0.118 | -2,621 | 0.0088 | 0.0838 | 83 |
| ENSG00000168393 | DTYMK | 1841 | 1,803.754 | -0.308 | 0.100 | -3,070 | 0.0021 | 0.0287 | NA |
| ENSG00000090097 | PCBP4 | 57060 | 1,521.806 | -0.306 | 0.119 | -2,584 | 0.0098 | 0.0894 | NA |
| ENSG00000151176 | PLBD2 | 196463 | 12,662.883 | -0.304 | 0.088 | -3,434 | 0.0006 | 0.0103 | NA |
| ENSG00000139405 | RITA1 | 84934 | 889,799 | -0.304 | 0.114 | -2,671 | 0.0076 | 0.0758 | NA |
| ENSG00000120963 | ZNF706 | 51123 | 1,542.622 | -0.303 | 0.093 | -3,257 | 0.0011 | 0.0173 | NA |
| ENSG00000121940 | CLCC1 | 23155 | 1,017.150 | -0.303 | 0.100 | -3,025 | 0.0025 | 0.0325 | NA |
| ENSG00000165934 | CPSF2 | 53981 | 2,515.140 | -0.303 | 0.111 | -2,723 | 0.0065 | 0.0672 | NA |
| ENSG00000157036 | EXOG | 9941 | 643,721 | -0.303 | 0.113 | -2,691 | 0.0071 | 0.0725 | NA |
| ENSG00000127241 | MASP1 | 5648 | 4,960.449 | -0.301 | 0.115 | -2,618 | 0.0089 | 0.0843 | NA |
| ENSG00000196562 | SULF2 | 55959 | 1,069.788 | -0.301 | 0.115 | -2,615 | 0.0089 | 0.0846 | NA |
| ENSG00000162976 | SLC66A3 | 130814 | 772,454 | -0.300 | 0.111 | -2,710 | 0.0067 | 0.0694 | NA |
| ENSG00000196411 | EPHB4 | 2050 | 1,734.115 | -0.300 | 0.118 | -2,539 | 0.0111 | 0.0986 | NA |
| ENSG00000093167 | LRRFIP2 | 9209 | 5,227.496 | -0.300 | 0.092 | -3,247 | 0.0012 | 0.0178 | 62 |
| ENSG00000115170 | ACVR1 | 90 | 2,257.510 | -0.298 | 0.115 | -2,593 | 0.0095 | 0.0882 | 89 |
| ENSG00000148248 | SURF4 | 6836 | 17,245.962 | -0.297 | 0.091 | -3,276 | 0.0011 | 0.0164 | NA |
| ENSG00000139168 | ZCRB1 | 85437 | 1,308.436 | -0.297 | 0.112 | -2,663 | 0.0077 | 0.0770 | 78 |
| ENSG00000076513 | ANKRD13A | 88455 | 4,853.922 | -0.296 | 0.113 | -2,630 | 0.0086 | 0.0824 | NA |
| ENSG00000117899 | MESD | 23184 | 3,196.257 | -0.295 | 0.093 | -3,173 | 0.0015 | 0.0220 | NA |
| ENSG00000154889 | MPPE1 | 65258 | 534,472 | -0.295 | 0.114 | -2,594 | 0.0095 | 0.0881 | NA |
| ENSG00000130830 | MPP1 | 4354 | 737,598 | -0.295 | 0.114 | -2,591 | 0.0096 | 0.0884 | NA |
| ENSG00000143344 | RGL1 | 23179 | 2,532.591 | -0.295 | 0.097 | -3,022 | 0.0025 | 0.0326 | 52 |
| ENSG00000113194 | FAF2 | 23197 | 3,203.611 | -0.294 | 0.088 | -3,326 | 0.0009 | 0.0142 | NA |
| ENSG00000254087 | LYN | 4067 | 1,499.685 | -0.294 | 0.113 | -2,591 | 0.0096 | 0.0884 | 87 |
| ENSG00000140443 | IGF1R | 3480 | 1,914.882 | -0.293 | 0.091 | -3,222 | 0.0013 | 0.0192 | NA |
| ENSG00000004975 | DVL2 | 1856 | 2,066.484 | -0.293 | 0.106 | -2,769 | 0.0056 | 0.0607 | NA |
| ENSG00000097007 | ABL1 | 25 | 6,630.750 | -0.293 | 0.097 | -3,025 | 0.0025 | 0.0325 | 91 |
| ENSG00000196704 | AMZ2 | 51321 | 1,759.952 | -0.292 | 0.105 | -2,786 | 0.0053 | 0.0584 | NA |
| ENSG00000081923 | ATP8B1 | 5205 | 9,496.883 | -0.292 | 0.114 | -2,556 | 0.0106 | 0.0949 | NA |
| ENSG00000111361 | EIF2B1 | 1967 | 1,762.765 | -0.291 | 0.093 | -3,126 | 0.0018 | 0.0249 | NA |
| ENSG00000135617 | PRADC1 | 84279 | 683,806 | -0.291 | 0.111 | -2,627 | 0.0086 | 0.0826 | NA |
| ENSG00000085449 | WDFY1 | 57590 | 3,657.365 | -0.289 | 0.088 | -3,290 | 0.0010 | 0.0157 | NA |
| ENSG00000075426 | FOSL2 | 2355 | 5,673.858 | -0.289 | 0.094 | -3,091 | 0.0020 | 0.0274 | NA |
| ENSG00000178974 | FBXO34 | 55030 | 678,069 | -0.287 | 0.111 | -2,581 | 0.0098 | 0.0897 | 84 |
| ENSG00000160271 | RALGDS | 5900 | 1,297.704 | -0.287 | 0.108 | -2,654 | 0.0079 | 0.0783 | 83 |
| ENSG00000112983 | BRD8 | 10902 | 1,945.434 | -0.286 | 0.100 | -2,860 | 0.0042 | 0.0491 | NA |
| ENSG00000170037 | CNTROB | 116840 | 1,703.811 | -0.285 | 0.098 | -2,892 | 0.0038 | 0.0454 | NA |
| ENSG00000167461 | RAB8A | 4218 | 1,071.980 | -0.284 | 0.104 | -2,744 | 0.0061 | 0.0644 | 94 |
| ENSG00000172500 | FIBP | 9158 | 2,317.762 | -0.282 | 0.092 | -3,056 | 0.0022 | 0.0299 | NA |
| ENSG00000167693 | NXN | 64359 | 4,499.776 | -0.280 | 0.082 | -3,415 | 0.0006 | 0.0109 | NA |
| ENSG00000124788 | ATXN1 | 6310 | 1,366.145 | -0.280 | 0.095 | -2,945 | 0.0032 | 0.0399 | 88 |
| ENSG00000141504 | SAT2 | 112483 | 1,640.619 | -0.279 | 0.106 | -2,633 | 0.0085 | 0.0820 | NA |
| ENSG00000074201 | CLNS1A | 1207 | 1,543.764 | -0.278 | 0.100 | -2,780 | 0.0054 | 0.0592 | NA |
| ENSG00000163069 | SGCB | 6443 | 4,526.068 | -0.277 | 0.105 | -2,629 | 0.0086 | 0.0824 | 71 |
| ENSG00000010810 | FYN | 2534 | 1,659.234 | -0.272 | 0.100 | -2,711 | 0.0067 | 0.0693 | NA |
| ENSG00000100364 | KIAA0930 | 23313 | 5,936.772 | -0.270 | 0.102 | -2,636 | 0.0084 | 0.0815 | NA |
| ENSG00000008283 | CYB561 | 1534 | 3,082.966 | -0.270 | 0.103 | -2,620 | 0.0088 | 0.0840 | 88 |
| ENSG00000136935 | GOLGA1 | 2800 | 844,740 | -0.268 | 0.104 | -2,578 | 0.0099 | 0.0903 | 96 |
| ENSG00000103035 | PSMD7 | 5713 | 1,491.961 | -0.263 | 0.101 | -2,611 | 0.0090 | 0.0852 | 94 |
| ENSG00000171793 | CTPS1 | 1503 | 3,195.083 | -0.261 | 0.101 | -2,591 | 0.0096 | 0.0884 | NA |
| ENSG00000184752 | NDUFA12 | 55967 | 1,164.099 | -0.253 | 0.099 | -2,542 | 0.0110 | 0.0978 | NA |
| ENSG00000025800 | KPNA6 | 23633 | 3,603.131 | -0.251 | 0.097 | -2,595 | 0.0094 | 0.0878 | 86 |
| ENSG00000025293 | PHF20 | 51230 | 2,324.525 | -0.251 | 0.095 | -2,633 | 0.0085 | 0.0819 | 61 |
| ENSG00000124422 | USP22 | 23326 | 7,367.424 | -0.249 | 0.088 | -2,831 | 0.0046 | 0.0526 | 64 |
| ENSG00000013297 | CLDN11 | 5010 | 4,714.607 | -0.248 | 0.097 | -2,563 | 0.0104 | 0.0934 | NA |
| ENSG00000124222 | STX16 | 8675 | 1,656.999 | -0.247 | 0.088 | -2,824 | 0.0047 | 0.0536 | 92 |
| ENSG00000080845 | DLGAP4 | 22839 | 4,112.567 | -0.247 | 0.093 | -2,646 | 0.0081 | 0.0796 | 89 |
| ENSG00000182022 | CHST15 | 51363 | 1,701.662 | -0.245 | 0.093 | -2,619 | 0.0088 | 0.0840 | NA |
| ENSG00000113083 | LOX | 4015 | 22,592.928 | -0.245 | 0.094 | -2,614 | 0.0090 | 0.0847 | 91 |
| ENSG00000172301 | COPRS | 55352 | 3,157.838 | -0.242 | 0.094 | -2,589 | 0.0096 | 0.0884 | NA |
| ENSG00000112640 | PPP2R5D | 5528 | 1,970.995 | -0.242 | 0.095 | -2,562 | 0.0104 | 0.0935 | NA |
| ENSG00000186184 | POLR1D | 51082 | 1,696.847 | -0.240 | 0.091 | -2,631 | 0.0085 | 0.0822 | NA |
| ENSG00000131508 | UBE2D2 | 7322 | 2,538.197 | -0.237 | 0.089 | -2,660 | 0.0078 | 0.0775 | NA |
| ENSG00000099956 | SMARCB1 | 6598 | 2,053.503 | -0.235 | 0.091 | -2,569 | 0.0102 | 0.0922 | NA |
| ENSG00000092964 | DPYSL2 | 1808 | 3,641.592 | -0.231 | 0.088 | -2,634 | 0.0084 | 0.0818 | 86 |
| ENSG00000204381 | LAYN | 143903 | 3,803.517 | -0.216 | 0.081 | -2,673 | 0.0075 | 0.0756 | NA |
| ENSG00000180304 | OAZ2 | 4947 | 3,804.381 | -0.202 | 0.078 | -2,582 | 0.0098 | 0.0896 | NA |
| ENSG00000044574 | HSPA5 | 3309 | 31,594.211 | 0.198 | 0.077 | 2,582 | 0.0098 | 0.0897 | NA |
| ENSG00000166250 | CLMP | 79827 | 16,146.786 | 0.205 | 0.081 | 2,535 | 0.0112 | 0.0989 | NA |
| ENSG00000188706 | ZDHHC9 | 51114 | 3,400.883 | 0.215 | 0.080 | 2,699 | 0.0070 | 0.0714 | NA |
| ENSG00000112531 | QKI | 9444 | 3,163.051 | 0.218 | 0.084 | 2,612 | 0.0090 | 0.0851 | 78 |
| ENSG00000103429 | BFAR | 51283 | 3,015.273 | 0.219 | 0.086 | 2,547 | 0.0109 | 0.0968 | NA |
| ENSG00000156113 | KCNMA1 | 3778 | 10,240.284 | 0.223 | 0.083 | 2,684 | 0.0073 | 0.0739 | NA |
| ENSG00000135930 | EIF4E2 | 9470 | 2,380.363 | 0.223 | 0.087 | 2,575 | 0.0100 | 0.0909 | NA |
| ENSG00000067082 | KLF6 | 1316 | 8,401.276 | 0.226 | 0.083 | 2,713 | 0.0067 | 0.0688 | NA |
| ENSG00000082701 | GSK3B | 2932 | 3,233.843 | 0.229 | 0.082 | 2,771 | 0.0056 | 0.0606 | NA |
| ENSG00000166747 | AP1G1 | 164 | 3,983.334 | 0.233 | 0.088 | 2,639 | 0.0083 | 0.0809 | NA |
| ENSG00000196655 | TRAPPC4 | 51399 | 1,677.745 | 0.234 | 0.091 | 2,590 | 0.0096 | 0.0884 | NA |
| ENSG00000001167 | NFYA | 4800 | 1,152.376 | 0.236 | 0.093 | 2,534 | 0.0113 | 0.0991 | NA |
| ENSG00000103381 | CPPED1 | 55313 | 2,310.296 | 0.237 | 0.092 | 2,582 | 0.0098 | 0.0897 | NA |
| ENSG00000135842 | NIBAN1 | 116496 | 3,999.614 | 0.238 | 0.089 | 2,660 | 0.0078 | 0.0775 | NA |
| ENSG00000174705 | SH3PXD2B | 285590 | 4,735.662 | 0.242 | 0.092 | 2,629 | 0.0086 | 0.0824 | NA |
| ENSG00000135047 | CTSL | 1514 | 21,173.065 | 0.243 | 0.093 | 2,609 | 0.0091 | 0.0855 | NA |
| ENSG00000176871 | WSB2 | 55884 | 7,395.456 | 0.244 | 0.082 | 2,967 | 0.0030 | 0.0378 | NA |
| ENSG00000132388 | UBE2G1 | 7326 | 1,541.256 | 0.244 | 0.093 | 2,635 | 0.0084 | 0.0815 | NA |
| ENSG00000147872 | PLIN2 | 123 | 4,275.091 | 0.246 | 0.088 | 2,785 | 0.0054 | 0.0586 | NA |
| ENSG00000127870 | RNF6 | 6049 | 2,460.310 | 0.246 | 0.097 | 2,537 | 0.0112 | 0.0988 | NA |
| ENSG00000136770 | DNAJC1 | 64215 | 1,532.152 | 0.247 | 0.094 | 2,616 | 0.0089 | 0.0844 | NA |
| ENSG00000156642 | NPTN | 27020 | 6,996.085 | 0.251 | 0.088 | 2,840 | 0.0045 | 0.0514 | NA |
| ENSG00000197496 | SLC2A10 | 81031 | 1,983.107 | 0.252 | 0.092 | 2,729 | 0.0064 | 0.0664 | NA |
| ENSG00000071054 | MAP4K4 | 9448 | 13,727.536 | 0.252 | 0.097 | 2,600 | 0.0093 | 0.0870 | 80 |
| ENSG00000197321 | SVIL | 6840 | 7,957.668 | 0.253 | 0.098 | 2,577 | 0.0100 | 0.0906 | NA |
| ENSG00000134531 | EMP1 | 2012 | 47,759.949 | 0.254 | 0.081 | 3,129 | 0.0018 | 0.0247 | NA |
| ENSG00000142687 | KIAA0319L | 79932 | 3,027.295 | 0.254 | 0.091 | 2,790 | 0.0053 | 0.0579 | NA |
| ENSG00000100196 | KDELR3 | 11015 | 4,413.097 | 0.254 | 0.095 | 2,676 | 0.0074 | 0.0750 | NA |
| ENSG00000058668 | ATP2B4 | 493 | 29,587.111 | 0.256 | 0.096 | 2,671 | 0.0076 | 0.0758 | NA |
| ENSG00000144043 | TEX261 | 113419 | 4,360.981 | 0.257 | 0.081 | 3,162 | 0.0016 | 0.0227 | NA |
| ENSG00000112308 | C6orf62 | 81688 | 5,862.779 | 0.258 | 0.096 | 2,698 | 0.0070 | 0.0714 | NA |
| ENSG00000196821 | ILRUN | 64771 | 4,459.033 | 0.259 | 0.090 | 2,874 | 0.0041 | 0.0475 | NA |
| ENSG00000105127 | AKAP8 | 10270 | 1,364.122 | 0.259 | 0.093 | 2,786 | 0.0053 | 0.0584 | NA |
| ENSG00000140992 | PDPK1 | 5170 | 1,454.050 | 0.260 | 0.092 | 2,823 | 0.0048 | 0.0536 | NA |
| ENSG00000156639 | ZFAND3 | 60685 | 3,655.313 | 0.261 | 0.101 | 2,586 | 0.0097 | 0.0892 | NA |
| ENSG00000188522 | FAM83G | 644815 | 1,482.069 | 0.261 | 0.102 | 2,552 | 0.0107 | 0.0958 | NA |
| ENSG00000168610 | STAT3 | 6774 | 5,783.418 | 0.262 | 0.095 | 2,769 | 0.0056 | 0.0607 | NA |
| ENSG00000050393 | MCUR1 | 63933 | 1,328.432 | 0.262 | 0.097 | 2,695 | 0.0070 | 0.0719 | NA |
| ENSG00000058085 | LAMC2 | 3918 | 3,543.862 | 0.264 | 0.097 | 2,723 | 0.0065 | 0.0672 | NA |
| ENSG00000115594 | IL1R1 | 3554 | 4,400.455 | 0.264 | 0.102 | 2,603 | 0.0092 | 0.0864 | NA |
| ENSG00000058673 | ZC3H11A | 9877 | 3,927.658 | 0.264 | 0.103 | 2,564 | 0.0103 | 0.0932 | NA |
| ENSG00000136813 | ECPAS | 23392 | 5,052.991 | 0.266 | 0.083 | 3,213 | 0.0013 | 0.0198 | NA |
| ENSG00000110931 | CAMKK2 | 10645 | 2,494.247 | 0.266 | 0.103 | 2,577 | 0.0100 | 0.0906 | 92 |
| ENSG00000143079 | CTTNBP2NL | 55917 | 1,745.191 | 0.266 | 0.103 | 2,590 | 0.0096 | 0.0884 | 53 |
| ENSG00000136888 | ATP6V1G1 | 9550 | 4,246.316 | 0.267 | 0.101 | 2,637 | 0.0084 | 0.0813 | NA |
| ENSG00000123983 | ACSL3 | 2181 | 3,103.106 | 0.269 | 0.091 | 2,950 | 0.0032 | 0.0394 | NA |
| ENSG00000077238 | IL4R | 3566 | 2,014.326 | 0.269 | 0.099 | 2,728 | 0.0064 | 0.0664 | NA |
| ENSG00000143499 | SMYD2 | 56950 | 739,611 | 0.269 | 0.106 | 2,543 | 0.0110 | 0.0978 | NA |
| ENSG00000103353 | UBFD1 | 56061 | 3,513.379 | 0.270 | 0.081 | 3,317 | 0.0009 | 0.0145 | NA |
| ENSG00000072786 | STK10 | 6793 | 3,242.396 | 0.270 | 0.105 | 2,574 | 0.0100 | 0.0910 | NA |
| ENSG00000187742 | SECISBP2 | 79048 | 1,050.042 | 0.270 | 0.107 | 2,533 | 0.0113 | 0.0992 | NA |
| ENSG00000111412 | SPRING1 | 79794 | 2,905.137 | 0.275 | 0.083 | 3,301 | 0.0010 | 0.0152 | NA |
| ENSG00000070831 | CDC42 | 998 | 8,617.566 | 0.275 | 0.100 | 2,750 | 0.0060 | 0.0635 | NA |
| ENSG00000156928 | MALSU1 | 115416 | 718,614 | 0.276 | 0.106 | 2,596 | 0.0094 | 0.0878 | NA |
| ENSG00000115159 | GPD2 | 2820 | 2,192.912 | 0.276 | 0.108 | 2,561 | 0.0104 | 0.0937 | NA |
| ENSG00000152818 | UTRN | 7402 | 3,840.819 | 0.278 | 0.095 | 2,929 | 0.0034 | 0.0414 | NA |
| ENSG00000071994 | PDCD2 | 5134 | 1,559.274 | 0.278 | 0.096 | 2,892 | 0.0038 | 0.0454 | NA |
| ENSG00000198380 | GFPT1 | 2673 | 5,381.657 | 0.279 | 0.097 | 2,871 | 0.0041 | 0.0477 | NA |
| ENSG00000134086 | VHL | 7428 | 992,015 | 0.280 | 0.102 | 2,749 | 0.0060 | 0.0637 | NA |
| ENSG00000134294 | SLC38A2 | 54407 | 12,921.261 | 0.281 | 0.100 | 2,819 | 0.0048 | 0.0542 | 89 |
| ENSG00000168256 | NKIRAS2 | 28511 | 2,367.482 | 0.282 | 0.104 | 2,727 | 0.0064 | 0.0666 | NA |
| ENSG00000124164 | VAPB | 9217 | 2,525.213 | 0.283 | 0.088 | 3,202 | 0.0014 | 0.0204 | NA |
| ENSG00000005810 | MYCBP2 | 23077 | 2,358.658 | 0.283 | 0.102 | 2,777 | 0.0055 | 0.0597 | NA |
| ENSG00000171310 | CHST11 | 50515 | 1,038.726 | 0.284 | 0.106 | 2,680 | 0.0074 | 0.0744 | NA |
| ENSG00000135829 | DHX9 | 1660 | 6,689.576 | 0.286 | 0.102 | 2,801 | 0.0051 | 0.0564 | NA |
| ENSG00000130348 | QRSL1 | 55278 | 599,531 | 0.287 | 0.111 | 2,583 | 0.0098 | 0.0895 | NA |
| ENSG00000155380 | SLC16A1 | 6566 | 2,134.539 | 0.288 | 0.102 | 2,818 | 0.0048 | 0.0543 | NA |
| ENSG00000006831 | ADIPOR2 | 79602 | 2,547.266 | 0.289 | 0.085 | 3,391 | 0.0007 | 0.0117 | NA |
| ENSG00000169398 | PTK2 | 5747 | 3,222.058 | 0.289 | 0.091 | 3,187 | 0.0014 | 0.0213 | NA |
| ENSG00000180357 | ZNF609 | 23060 | 1,826.684 | 0.289 | 0.095 | 3,040 | 0.0024 | 0.0312 | NA |
| ENSG00000138593 | SECISBP2L | 9728 | 2,085.934 | 0.289 | 0.100 | 2,877 | 0.0040 | 0.0472 | NA |
| ENSG00000150776 | NKAPD1 | 55216 | 860,384 | 0.289 | 0.110 | 2,627 | 0.0086 | 0.0826 | NA |
| ENSG00000138434 | ITPRID2 | 6744 | 9,868.725 | 0.289 | 0.113 | 2,560 | 0.0105 | 0.0940 | NA |
| ENSG00000080815 | PSEN1 | 5663 | 2,822.033 | 0.290 | 0.097 | 2,985 | 0.0028 | 0.0359 | NA |
| ENSG00000154122 | ANKH | 56172 | 1,148.461 | 0.290 | 0.097 | 2,983 | 0.0029 | 0.0360 | NA |
| ENSG00000197894 | ADH5 | 128 | 7,602.589 | 0.292 | 0.083 | 3,533 | 0.0004 | 0.0076 | NA |
| ENSG00000122741 | DCAF10 | 79269 | 831,543 | 0.292 | 0.103 | 2,841 | 0.0045 | 0.0514 | NA |
| ENSG00000111911 | HINT3 | 135114 | 989,629 | 0.293 | 0.115 | 2,553 | 0.0107 | 0.0957 | NA |
| ENSG00000159346 | ADIPOR1 | 51094 | 4,501.936 | 0.294 | 0.095 | 3,102 | 0.0019 | 0.0265 | NA |
| ENSG00000115310 | RTN4 | 57142 | 47,592.791 | 0.294 | 0.100 | 2,934 | 0.0033 | 0.0409 | NA |
| ENSG00000198856 | OSTC | 58505 | 2,999.976 | 0.296 | 0.115 | 2,576 | 0.0100 | 0.0907 | NA |
| ENSG00000100994 | PYGB | 5834 | 6,879.407 | 0.296 | 0.117 | 2,537 | 0.0112 | 0.0988 | NA |
| ENSG00000101577 | LPIN2 | 9663 | 2,365.279 | 0.299 | 0.102 | 2,927 | 0.0034 | 0.0415 | NA |
| ENSG00000072609 | CHFR | 55743 | 1,455.741 | 0.299 | 0.113 | 2,646 | 0.0081 | 0.0796 | NA |
| ENSG00000140632 | GLYR1 | 84656 | 4,305.598 | 0.300 | 0.078 | 3,864 | 0.0001 | 0.0026 | NA |
| ENSG00000134287 | ARF3 | 377 | 7,205.573 | 0.300 | 0.093 | 3,228 | 0.0012 | 0.0189 | NA |
| ENSG00000071127 | WDR1 | 9948 | 23,911.313 | 0.300 | 0.096 | 3,116 | 0.0018 | 0.0255 | NA |
| ENSG00000129315 | CCNT1 | 904 | 1,101.833 | 0.303 | 0.113 | 2,692 | 0.0071 | 0.0724 | NA |
| ENSG00000159140 | SON | 6651 | 8,039.471 | 0.304 | 0.084 | 3,630 | 0.0003 | 0.0056 | NA |
| ENSG00000157827 | FMNL2 | 114793 | 1,847.140 | 0.304 | 0.111 | 2,736 | 0.0062 | 0.0655 | NA |
| ENSG00000168818 | STX18 | 53407 | 1,244.472 | 0.305 | 0.092 | 3,320 | 0.0009 | 0.0145 | NA |
| ENSG00000171132 | PRKCE | 5581 | 1,299.088 | 0.305 | 0.105 | 2,903 | 0.0037 | 0.0443 | NA |
| ENSG00000102172 | SMS | 6611 | 4,959.430 | 0.305 | 0.105 | 2,891 | 0.0038 | 0.0454 | NA |
| ENSG00000110841 | PPFIBP1 | 8496 | 2,286.638 | 0.305 | 0.108 | 2,822 | 0.0048 | 0.0538 | NA |
| ENSG00000118495 | PLAGL1 | 5325 | 1,280.255 | 0.305 | 0.117 | 2,606 | 0.0092 | 0.0860 | NA |
| ENSG00000088854 | C20orf194 | 25943 | 1,465.096 | 0.306 | 0.095 | 3,234 | 0.0012 | 0.0186 | NA |
| ENSG00000106105 | GARS1 | 2617 | 13,318.137 | 0.307 | 0.077 | 3,958 | 0.0001 | 0.0019 | NA |
| ENSG00000142949 | PTPRF | 5792 | 7,768.905 | 0.307 | 0.093 | 3,312 | 0.0009 | 0.0148 | NA |
| ENSG00000109079 | TNFAIP1 | 7126 | 3,974.920 | 0.307 | 0.103 | 2,984 | 0.0028 | 0.0360 | NA |
| ENSG00000058063 | ATP11B | 23200 | 1,486.202 | 0.307 | 0.107 | 2,873 | 0.0041 | 0.0476 | NA |
| ENSG00000040531 | CTNS | 1497 | 1,465.108 | 0.308 | 0.095 | 3,232 | 0.0012 | 0.0186 | NA |
| ENSG00000114978 | MOB1A | 55233 | 3,108.303 | 0.309 | 0.111 | 2,783 | 0.0054 | 0.0588 | NA |
| ENSG00000119408 | NEK6 | 10783 | 4,680.783 | 0.310 | 0.082 | 3,758 | 0.0002 | 0.0037 | NA |
| ENSG00000118579 | MED28 | 80306 | 1,481.273 | 0.310 | 0.100 | 3,094 | 0.0020 | 0.0272 | NA |
| ENSG00000105058 | FAM32A | 26017 | 3,437.141 | 0.310 | 0.117 | 2,651 | 0.0080 | 0.0789 | NA |
| ENSG00000122376 | SHLD2 | 54537 | 1,220.905 | 0.310 | 0.118 | 2,623 | 0.0087 | 0.0834 | NA |
| ENSG00000102401 | ARMCX3 | 51566 | 2,517.395 | 0.311 | 0.092 | 3,384 | 0.0007 | 0.0119 | NA |
| ENSG00000179820 | MYADM | 91663 | 7,502.910 | 0.311 | 0.108 | 2,890 | 0.0039 | 0.0456 | NA |
| ENSG00000164951 | PDP1 | 54704 | 1,013.173 | 0.312 | 0.102 | 3,046 | 0.0023 | 0.0307 | NA |
| ENSG00000073712 | FERMT2 | 10979 | 4,011.805 | 0.312 | 0.111 | 2,798 | 0.0051 | 0.0568 | NA |
| ENSG00000164741 | DLC1 | 10395 | 8,073.871 | 0.313 | 0.100 | 3,131 | 0.0017 | 0.0246 | NA |
| ENSG00000166147 | FBN1 | 2200 | 40,936.604 | 0.313 | 0.107 | 2,937 | 0.0033 | 0.0405 | NA |
| ENSG00000198900 | TOP1 | 7150 | 4,606.185 | 0.314 | 0.096 | 3,268 | 0.0011 | 0.0167 | NA |
| ENSG00000082512 | TRAF5 | 7188 | 514,021 | 0.314 | 0.119 | 2,647 | 0.0081 | 0.0796 | NA |
| ENSG00000102081 | FMR1 | 2332 | 1,171.948 | 0.314 | 0.123 | 2,542 | 0.0110 | 0.0978 | NA |
| ENSG00000128512 | DOCK4 | 9732 | 1,125.199 | 0.315 | 0.121 | 2,591 | 0.0096 | 0.0884 | NA |
| ENSG00000101310 | SEC23B | 10483 | 2,723.104 | 0.316 | 0.107 | 2,944 | 0.0032 | 0.0400 | NA |
| ENSG00000172466 | ZNF24 | 7572 | 1,580.575 | 0.317 | 0.109 | 2,906 | 0.0037 | 0.0440 | NA |
| ENSG00000173065 | FAM222B | 55731 | 898,353 | 0.318 | 0.114 | 2,788 | 0.0053 | 0.0583 | NA |
| ENSG00000181704 | YIPF6 | 286451 | 1,802.601 | 0.318 | 0.119 | 2,667 | 0.0076 | 0.0764 | NA |
| ENSG00000127947 | PTPN12 | 5782 | 2,305.371 | 0.320 | 0.121 | 2,649 | 0.0081 | 0.0792 | NA |
| ENSG00000214548 | MEG3 | 55384 | 10,829.117 | 0.320 | 0.126 | 2,535 | 0.0112 | 0.0989 | NA |
| ENSG00000176994 | SMCR8 | 140775 | 2,553.192 | 0.321 | 0.091 | 3,538 | 0.0004 | 0.0075 | NA |
| ENSG00000173950 | XXYLT1 | 152002 | 1,352.285 | 0.321 | 0.091 | 3,521 | 0.0004 | 0.0079 | NA |
| ENSG00000110583 | NAA40 | 79829 | 1,043.560 | 0.321 | 0.096 | 3,325 | 0.0009 | 0.0142 | NA |
| ENSG00000164715 | LMTK2 | 22853 | 1,139.566 | 0.321 | 0.102 | 3,151 | 0.0016 | 0.0233 | NA |
| ENSG00000186951 | PPARA | 5465 | 1,033.572 | 0.321 | 0.106 | 3,033 | 0.0024 | 0.0317 | NA |
| ENSG00000075643 | MOCOS | 55034 | 1,716.076 | 0.321 | 0.108 | 2,958 | 0.0031 | 0.0386 | NA |
| ENSG00000086061 | DNAJA1 | 3301 | 9,288.733 | 0.321 | 0.111 | 2,878 | 0.0040 | 0.0471 | NA |
| ENSG00000171940 | ZNF217 | 7764 | 2,130.954 | 0.322 | 0.095 | 3,378 | 0.0007 | 0.0122 | NA |
| ENSG00000159399 | HK2 | 3099 | 4,522.483 | 0.324 | 0.121 | 2,671 | 0.0076 | 0.0758 | NA |
| ENSG00000164244 | PRRC1 | 133619 | 3,410.553 | 0.325 | 0.100 | 3,240 | 0.0012 | 0.0182 | NA |
| ENSG00000067066 | SP100 | 6672 | 4,951.598 | 0.325 | 0.108 | 3,008 | 0.0026 | 0.0338 | NA |
| ENSG00000198042 | MAK16 | 84549 | 710,930 | 0.325 | 0.114 | 2,859 | 0.0042 | 0.0493 | NA |
| ENSG00000144747 | TMF1 | 7110 | 1,649.910 | 0.325 | 0.115 | 2,821 | 0.0048 | 0.0538 | NA |
| ENSG00000167470 | MIDN | 90007 | 3,514.714 | 0.325 | 0.124 | 2,620 | 0.0088 | 0.0840 | NA |
| ENSG00000173193 | PARP14 | 54625 | 8,615.114 | 0.326 | 0.121 | 2,689 | 0.0072 | 0.0730 | NA |
| ENSG00000091436 | MAP3K20 | 51776 | 3,417.463 | 0.326 | 0.124 | 2,632 | 0.0085 | 0.0821 | NA |
| ENSG00000133103 | COG6 | 57511 | 1,210.692 | 0.326 | 0.125 | 2,614 | 0.0090 | 0.0847 | NA |
| ENSG00000154319 | FAM167A | 83648 | 1,019.449 | 0.327 | 0.126 | 2,595 | 0.0094 | 0.0878 | NA |
| ENSG00000107290 | SETX | 23064 | 2,161.055 | 0.328 | 0.108 | 3,022 | 0.0025 | 0.0326 | NA |
| ENSG00000103160 | HSDL1 | 83693 | 770,775 | 0.328 | 0.124 | 2,637 | 0.0084 | 0.0813 | NA |
| ENSG00000134748 | PRPF38A | 84950 | 783,897 | 0.329 | 0.110 | 2,996 | 0.0027 | 0.0350 | NA |
| ENSG00000171466 | ZNF562 | 54811 | 988,312 | 0.329 | 0.113 | 2,912 | 0.0036 | 0.0433 | NA |
| ENSG00000166471 | TMEM41B | 440026 | 720,794 | 0.329 | 0.120 | 2,740 | 0.0061 | 0.0650 | NA |
| ENSG00000197586 | ENTPD6 | 955 | 3,688.771 | 0.329 | 0.120 | 2,734 | 0.0063 | 0.0658 | NA |
| ENSG00000132840 | BHMT2 | 23743 | 629,828 | 0.329 | 0.125 | 2,627 | 0.0086 | 0.0825 | NA |
| ENSG00000082805 | ERC1 | 23085 | 3,617.167 | 0.332 | 0.099 | 3,334 | 0.0009 | 0.0139 | NA |
| ENSG00000131844 | MCCC2 | 64087 | 1,964.126 | 0.333 | 0.102 | 3,271 | 0.0011 | 0.0166 | NA |
| ENSG00000173848 | NET1 | 10276 | 1,424.861 | 0.333 | 0.111 | 2,990 | 0.0028 | 0.0355 | NA |
| ENSG00000135919 | SERPINE2 | 5270 | 76,890.365 | 0.334 | 0.080 | 4,160 | 0.0000 | 0.0009 | NA |
| ENSG00000096070 | BRPF3 | 27154 | 1,878.986 | 0.334 | 0.094 | 3,566 | 0.0004 | 0.0068 | NA |
| ENSG00000260032 | NORAD | 647979 | 15,594.900 | 0.334 | 0.100 | 3,328 | 0.0009 | 0.0141 | NA |
| ENSG00000153714 | LURAP1L | 286343 | 754,278 | 0.334 | 0.126 | 2,657 | 0.0079 | 0.0780 | NA |
| ENSG00000076067 | RBMS2 | 5939 | 4,053.189 | 0.334 | 0.128 | 2,617 | 0.0089 | 0.0843 | NA |
| ENSG00000119487 | MAPKAP1 | 79109 | 6,430.138 | 0.335 | 0.093 | 3,586 | 0.0003 | 0.0064 | NA |
| ENSG00000008952 | SEC62 | 7095 | 3,573.388 | 0.335 | 0.117 | 2,852 | 0.0044 | 0.0502 | NA |
| ENSG00000143842 | SOX13 | 9580 | 471,423 | 0.335 | 0.124 | 2,698 | 0.0070 | 0.0714 | NA |
| ENSG00000040199 | PHLPP2 | 23035 | 1,073.483 | 0.335 | 0.132 | 2,542 | 0.0110 | 0.0978 | NA |
| ENSG00000231074 | HCG18 | 414777 | 852,749 | 0.336 | 0.112 | 2,994 | 0.0028 | 0.0351 | NA |
| ENSG00000186174 | BCL9L | 283149 | 6,371.643 | 0.337 | 0.130 | 2,583 | 0.0098 | 0.0895 | NA |
| ENSG00000154813 | DPH3 | 285381 | 1,208.067 | 0.338 | 0.112 | 3,033 | 0.0024 | 0.0317 | NA |
| ENSG00000153071 | DAB2 | 1601 | 11,506.124 | 0.339 | 0.090 | 3,774 | 0.0002 | 0.0035 | NA |
| ENSG00000225830 | ERCC6 | 2074 | 1,423.596 | 0.339 | 0.107 | 3,160 | 0.0016 | 0.0228 | NA |
| ENSG00000166068 | SPRED1 | 161742 | 1,775.060 | 0.341 | 0.096 | 3,532 | 0.0004 | 0.0076 | NA |
| ENSG00000110395 | CBL | 867 | 1,819.536 | 0.341 | 0.124 | 2,736 | 0.0062 | 0.0655 | NA |
| ENSG00000175592 | FOSL1 | 8061 | 13,393.394 | 0.342 | 0.122 | 2,802 | 0.0051 | 0.0564 | NA |
| ENSG00000157214 | STEAP2 | 261729 | 987,256 | 0.342 | 0.123 | 2,793 | 0.0052 | 0.0575 | NA |
| ENSG00000196526 | AFAP1 | 60312 | 2,199.547 | 0.343 | 0.103 | 3,320 | 0.0009 | 0.0145 | NA |
| ENSG00000158711 | ELK4 | 2005 | 784,455 | 0.343 | 0.124 | 2,759 | 0.0058 | 0.0621 | NA |
| ENSG00000138035 | PNPT1 | 87178 | 2,684.133 | 0.343 | 0.132 | 2,605 | 0.0092 | 0.0860 | NA |
| ENSG00000142627 | EPHA2 | 1969 | 2,581.045 | 0.346 | 0.130 | 2,655 | 0.0079 | 0.0783 | NA |
| ENSG00000068305 | MEF2A | 4205 | 1,624.239 | 0.347 | 0.101 | 3,435 | 0.0006 | 0.0103 | NA |
| ENSG00000139514 | SLC7A1 | 6541 | 7,125.805 | 0.348 | 0.085 | 4,094 | 0.0000 | 0.0012 | NA |
| ENSG00000051108 | HERPUD1 | 9709 | 2,576.027 | 0.348 | 0.097 | 3,578 | 0.0003 | 0.0066 | NA |
| ENSG00000076685 | NT5C2 | 22978 | 1,549.924 | 0.348 | 0.099 | 3,501 | 0.0005 | 0.0084 | NA |
| ENSG00000100580 | TMED8 | 283578 | 907,919 | 0.349 | 0.105 | 3,317 | 0.0009 | 0.0145 | NA |
| ENSG00000196204 | RNF216P1 | 441191 | 698,669 | 0.349 | 0.120 | 2,900 | 0.0037 | 0.0446 | NA |
| ENSG00000136868 | SLC31A1 | 1317 | 3,504.175 | 0.350 | 0.099 | 3,534 | 0.0004 | 0.0076 | NA |
| ENSG00000177426 | TGIF1 | 7050 | 1,935.675 | 0.350 | 0.102 | 3,444 | 0.0006 | 0.0100 | NA |
| ENSG00000145623 | OSMR | 9180 | 4,108.523 | 0.351 | 0.108 | 3,257 | 0.0011 | 0.0173 | NA |
| ENSG00000159479 | MED8 | 112950 | 1,296.308 | 0.351 | 0.111 | 3,156 | 0.0016 | 0.0230 | NA |
| ENSG00000171621 | SPSB1 | 80176 | 1,698.978 | 0.351 | 0.124 | 2,828 | 0.0047 | 0.0531 | NA |
| ENSG00000198542 | ITGBL1 | 9358 | 6,616.888 | 0.352 | 0.114 | 3,083 | 0.0020 | 0.0279 | NA |
| ENSG00000057019 | DCBLD2 | 131566 | 10,365.536 | 0.352 | 0.125 | 2,823 | 0.0048 | 0.0537 | NA |
| ENSG00000196116 | TDRD7 | 23424 | 1,887.854 | 0.353 | 0.125 | 2,824 | 0.0047 | 0.0536 | NA |
| ENSG00000135968 | GCC2 | 9648 | 1,134.305 | 0.353 | 0.129 | 2,737 | 0.0062 | 0.0654 | NA |
| ENSG00000169247 | SH3TC2 | 79628 | 554,630 | 0.353 | 0.135 | 2,618 | 0.0088 | 0.0842 | NA |
| ENSG00000166750 | SLFN5 | 162394 | 12,016.971 | 0.355 | 0.111 | 3,199 | 0.0014 | 0.0205 | NA |
| ENSG00000126947 | ARMCX1 | 51309 | 1,984.700 | 0.355 | 0.120 | 2,967 | 0.0030 | 0.0378 | NA |
| ENSG00000115756 | HPCAL1 | 3241 | 10,124.140 | 0.355 | 0.122 | 2,909 | 0.0036 | 0.0436 | NA |
| ENSG00000176438 | SYNE3 | 161176 | 1,976.834 | 0.356 | 0.100 | 3,547 | 0.0004 | 0.0073 | NA |
| ENSG00000144535 | DIS3L2 | 129563 | 630,334 | 0.356 | 0.129 | 2,754 | 0.0059 | 0.0629 | NA |
| ENSG00000094975 | SUCO | 51430 | 1,536.592 | 0.356 | 0.134 | 2,666 | 0.0077 | 0.0767 | NA |
| ENSG00000153179 | RASSF3 | 283349 | 1,602.121 | 0.357 | 0.101 | 3,535 | 0.0004 | 0.0075 | NA |
| ENSG00000110092 | CCND1 | 595 | 64,818.497 | 0.357 | 0.113 | 3,150 | 0.0016 | 0.0234 | NA |
| ENSG00000163017 | ACTG2 | 72 | 1,810.433 | 0.358 | 0.124 | 2,878 | 0.0040 | 0.0471 | NA |
| ENSG00000164332 | UBLCP1 | 134510 | 1,038.728 | 0.358 | 0.135 | 2,642 | 0.0082 | 0.0805 | NA |
| ENSG00000170873 | MTSS1 | 9788 | 435,679 | 0.358 | 0.136 | 2,630 | 0.0085 | 0.0823 | NA |
| ENSG00000165322 | ARHGAP12 | 94134 | 2,330.840 | 0.359 | 0.112 | 3,207 | 0.0013 | 0.0201 | NA |
| ENSG00000255248 | MIR100HG | 399959 | 2,229.895 | 0.359 | 0.123 | 2,923 | 0.0035 | 0.0419 | NA |
| ENSG00000060982 | BCAT1 | 586 | 2,952.243 | 0.360 | 0.126 | 2,846 | 0.0044 | 0.0508 | NA |
| ENSG00000180530 | NRIP1 | 8204 | 957,836 | 0.360 | 0.128 | 2,816 | 0.0049 | 0.0546 | NA |
| ENSG00000120805 | ARL1 | 400 | 3,119.610 | 0.362 | 0.119 | 3,034 | 0.0024 | 0.0317 | NA |
| ENSG00000105738 | SIPA1L3 | 23094 | 1,388.923 | 0.362 | 0.133 | 2,713 | 0.0067 | 0.0688 | NA |
| ENSG00000151552 | QDPR | 5860 | 1,621.864 | 0.363 | 0.092 | 3,946 | 0.0001 | 0.0020 | NA |
| ENSG00000106785 | TRIM14 | 9830 | 3,815.483 | 0.363 | 0.117 | 3,090 | 0.0020 | 0.0274 | NA |
| ENSG00000237172 | B3GNT9 | 84752 | 1,473.358 | 0.363 | 0.123 | 2,941 | 0.0033 | 0.0402 | NA |
| ENSG00000174151 | CYB561D1 | 284613 | 898,282 | 0.364 | 0.106 | 3,428 | 0.0006 | 0.0105 | NA |
| ENSG00000123066 | MED13L | 23389 | 2,178.623 | 0.364 | 0.110 | 3,308 | 0.0009 | 0.0149 | NA |
| ENSG00000100523 | DDHD1 | 80821 | 926,529 | 0.364 | 0.117 | 3,120 | 0.0018 | 0.0253 | NA |
| ENSG00000132640 | BTBD3 | 22903 | 721,177 | 0.365 | 0.143 | 2,551 | 0.0107 | 0.0959 | NA |
| ENSG00000166326 | TRIM44 | 54765 | 4,649.656 | 0.366 | 0.090 | 4,063 | 0.0000 | 0.0013 | NA |
| ENSG00000141084 | RANBP10 | 57610 | 1,152.163 | 0.366 | 0.107 | 3,436 | 0.0006 | 0.0103 | NA |
| ENSG00000144824 | PHLDB2 | 90102 | 666,695 | 0.367 | 0.141 | 2,600 | 0.0093 | 0.0870 | NA |
| ENSG00000173442 | EHBP1L1 | 254102 | 4,970.633 | 0.367 | 0.141 | 2,592 | 0.0096 | 0.0884 | NA |
| ENSG00000152952 | PLOD2 | 5352 | 12,351.733 | 0.369 | 0.109 | 3,392 | 0.0007 | 0.0117 | NA |
| ENSG00000121864 | ZNF639 | 51193 | 652,995 | 0.369 | 0.111 | 3,335 | 0.0009 | 0.0139 | NA |
| ENSG00000135953 | MFSD9 | 84804 | 332,340 | 0.369 | 0.145 | 2,540 | 0.0111 | 0.0984 | NA |
| ENSG00000134352 | IL6ST | 3572 | 20,315.874 | 0.370 | 0.131 | 2,837 | 0.0046 | 0.0519 | NA |
| ENSG00000232533 |  |  | 548,645 | 0.370 | 0.132 | 2,798 | 0.0051 | 0.0568 | NA |
| ENSG00000142961 | MOB3C | 148932 | 984,203 | 0.370 | 0.139 | 2,655 | 0.0079 | 0.0783 | NA |
| ENSG00000146242 | TPBG | 7162 | 5,644.924 | 0.372 | 0.096 | 3,892 | 0.0001 | 0.0024 | NA |
| ENSG00000029364 | SLC39A9 | 55334 | 2,733.238 | 0.372 | 0.110 | 3,365 | 0.0008 | 0.0127 | NA |
| ENSG00000163597 | SNHG16 | 1E+08 | 1,965.496 | 0.372 | 0.119 | 3,122 | 0.0018 | 0.0252 | NA |
| ENSG00000088367 | EPB41L1 | 2036 | 1,521.856 | 0.372 | 0.136 | 2,731 | 0.0063 | 0.0661 | NA |
| ENSG00000204442 | FAM155A | 728215 | 454,308 | 0.372 | 0.140 | 2,651 | 0.0080 | 0.0789 | NA |
| ENSG00000162086 | ZNF75A | 7627 | 445,715 | 0.373 | 0.123 | 3,027 | 0.0025 | 0.0323 | NA |
| ENSG00000146676 | PURB | 5814 | 1,382.423 | 0.375 | 0.137 | 2,744 | 0.0061 | 0.0644 | NA |
| ENSG00000168495 | POLR3D | 661 | 1,698.697 | 0.376 | 0.109 | 3,458 | 0.0005 | 0.0096 | NA |
| ENSG00000114861 | FOXP1 | 27086 | 1,191.431 | 0.376 | 0.112 | 3,361 | 0.0008 | 0.0129 | NA |
| ENSG00000117475 | BLZF1 | 8548 | 2,201.970 | 0.376 | 0.122 | 3,072 | 0.0021 | 0.0286 | NA |
| ENSG00000157193 | LRP8 | 7804 | 1,655.660 | 0.377 | 0.101 | 3,741 | 0.0002 | 0.0039 | NA |
| ENSG00000175376 | EIF1AD | 84285 | 908,263 | 0.377 | 0.103 | 3,652 | 0.0003 | 0.0052 | NA |
| ENSG00000070159 | PTPN3 | 5774 | 607,376 | 0.378 | 0.111 | 3,399 | 0.0007 | 0.0114 | NA |
| ENSG00000087510 | TFAP2C | 7022 | 2,916.790 | 0.380 | 0.093 | 4,096 | 0.0000 | 0.0012 | NA |
| ENSG00000144791 | LIMD1 | 8994 | 1,221.736 | 0.380 | 0.118 | 3,215 | 0.0013 | 0.0197 | NA |
| ENSG00000163788 | SNRK | 54861 | 606,929 | 0.380 | 0.134 | 2,845 | 0.0044 | 0.0508 | NA |
| ENSG00000223959 | AFG3L1P | 172 | 446,525 | 0.380 | 0.139 | 2,729 | 0.0063 | 0.0663 | NA |
| ENSG00000100219 | XBP1 | 7494 | 4,691.226 | 0.381 | 0.101 | 3,773 | 0.0002 | 0.0035 | NA |
| ENSG00000198455 | ZXDB | 158586 | 331,329 | 0.382 | 0.139 | 2,752 | 0.0059 | 0.0633 | NA |
| ENSG00000196935 | SRGAP1 | 57522 | 2,085.481 | 0.383 | 0.116 | 3,309 | 0.0009 | 0.0149 | NA |
| ENSG00000187498 | COL4A1 | 1282 | 7,672.613 | 0.383 | 0.145 | 2,636 | 0.0084 | 0.0814 | NA |
| ENSG00000197343 | ZNF655 | 79027 | 1,383.743 | 0.384 | 0.103 | 3,734 | 0.0002 | 0.0040 | NA |
| ENSG00000157168 | NRG1 | 3084 | 2,526.096 | 0.384 | 0.110 | 3,500 | 0.0005 | 0.0084 | NA |
| ENSG00000142197 | DOP1B | 9980 | 1,090.580 | 0.386 | 0.106 | 3,641 | 0.0003 | 0.0054 | NA |
| ENSG00000172493 | AFF1 | 4299 | 3,258.887 | 0.387 | 0.095 | 4,062 | 0.0000 | 0.0013 | NA |
| ENSG00000112305 | SMAP1 | 60682 | 249,401 | 0.387 | 0.150 | 2,589 | 0.0096 | 0.0884 | NA |
| ENSG00000198142 | SOWAHC | 65124 | 430,390 | 0.388 | 0.139 | 2,794 | 0.0052 | 0.0574 | NA |
| ENSG00000134070 | IRAK2 | 3656 | 1,240.184 | 0.391 | 0.130 | 3,009 | 0.0026 | 0.0337 | NA |
| ENSG00000139364 | TMEM132B | 114795 | 305,919 | 0.391 | 0.143 | 2,726 | 0.0064 | 0.0667 | NA |
| ENSG00000165246 | NLGN4Y | 22829 | 874,700 | 0.394 | 0.103 | 3,823 | 0.0001 | 0.0030 | NA |
| ENSG00000157657 | ZNF618 | 114991 | 1,372.214 | 0.394 | 0.118 | 3,333 | 0.0009 | 0.0140 | NA |
| ENSG00000136381 | IREB2 | 3658 | 1,926.753 | 0.394 | 0.120 | 3,273 | 0.0011 | 0.0165 | NA |
| ENSG00000100335 | MIEF1 | 54471 | 2,887.583 | 0.395 | 0.085 | 4,673 | 0.0000 | 0.0001 | NA |
| ENSG00000176095 | IP6K1 | 9807 | 2,501.440 | 0.395 | 0.090 | 4,387 | 0.0000 | 0.0004 | NA |
| ENSG00000121060 | TRIM25 | 7706 | 9,036.886 | 0.395 | 0.091 | 4,324 | 0.0000 | 0.0005 | NA |
| ENSG00000163814 | CDCP1 | 64866 | 566,408 | 0.395 | 0.142 | 2,771 | 0.0056 | 0.0605 | NA |
| ENSG00000221988 | PPT2 | 9374 | 617,754 | 0.395 | 0.149 | 2,654 | 0.0080 | 0.0783 | NA |
| ENSG00000062194 | GPBP1 | 65056 | 1,456.431 | 0.396 | 0.112 | 3,523 | 0.0004 | 0.0078 | NA |
| ENSG00000175764 | TTLL11 | 158135 | 268,213 | 0.396 | 0.156 | 2,533 | 0.0113 | 0.0992 | NA |
| ENSG00000137309 | HMGA1 | 3159 | 28,021.277 | 0.397 | 0.127 | 3,121 | 0.0018 | 0.0252 | NA |
| ENSG00000198466 | ZNF587 | 84914 | 456,907 | 0.397 | 0.145 | 2,740 | 0.0062 | 0.0650 | NA |
| ENSG00000170234 | PWWP2A | 114825 | 546,866 | 0.398 | 0.121 | 3,300 | 0.0010 | 0.0152 | NA |
| ENSG00000133961 | NUMB | 8650 | 3,542.411 | 0.399 | 0.080 | 4,998 | 0.0000 | 0.0000 | NA |
| ENSG00000198363 | ASPH | 444 | 7,204.979 | 0.399 | 0.123 | 3,257 | 0.0011 | 0.0173 | NA |
| ENSG00000158417 | EIF5B | 9669 | 5,661.560 | 0.400 | 0.111 | 3,596 | 0.0003 | 0.0062 | NA |
| ENSG00000105971 | CAV2 | 858 | 7,081.223 | 0.400 | 0.117 | 3,436 | 0.0006 | 0.0103 | NA |
| ENSG00000196187 | TMEM63A | 9725 | 2,242.645 | 0.400 | 0.122 | 3,279 | 0.0010 | 0.0162 | NA |
| ENSG00000178695 | KCTD12 | 115207 | 7,103.798 | 0.401 | 0.112 | 3,573 | 0.0004 | 0.0067 | NA |
| ENSG00000124532 | MRS2 | 57380 | 933,397 | 0.401 | 0.117 | 3,436 | 0.0006 | 0.0103 | NA |
| ENSG00000134897 | BIVM | 54841 | 350,307 | 0.401 | 0.158 | 2,537 | 0.0112 | 0.0988 | NA |
| ENSG00000134324 | LPIN1 | 23175 | 2,935.076 | 0.402 | 0.089 | 4,502 | 0.0000 | 0.0002 | NA |
| ENSG00000186522 | SEPTIN10 | 151011 | 2,028.131 | 0.403 | 0.128 | 3,156 | 0.0016 | 0.0230 | NA |
| ENSG00000177432 | NAP1L5 | 266812 | 357,375 | 0.403 | 0.150 | 2,680 | 0.0074 | 0.0744 | NA |
| ENSG00000175066 | GK5 | 256356 | 497,437 | 0.405 | 0.132 | 3,071 | 0.0021 | 0.0286 | NA |
| ENSG00000105810 | CDK6 | 1021 | 6,248.280 | 0.405 | 0.142 | 2,846 | 0.0044 | 0.0508 | NA |
| ENSG00000148730 | EIF4EBP2 | 1979 | 4,749.493 | 0.406 | 0.106 | 3,819 | 0.0001 | 0.0030 | NA |
| ENSG00000124702 | KLHDC3 | 116138 | 2,291.337 | 0.408 | 0.111 | 3,685 | 0.0002 | 0.0047 | NA |
| ENSG00000145545 | SRD5A1 | 6715 | 773,961 | 0.410 | 0.129 | 3,172 | 0.0015 | 0.0220 | NA |
| ENSG00000135269 | TES | 26136 | 958,465 | 0.410 | 0.131 | 3,138 | 0.0017 | 0.0241 | NA |
| ENSG00000153936 | HS2ST1 | 9653 | 786,979 | 0.410 | 0.144 | 2,845 | 0.0044 | 0.0508 | NA |
| ENSG00000198805 | PNP | 4860 | 1,728.056 | 0.411 | 0.146 | 2,826 | 0.0047 | 0.0534 | NA |
| ENSG00000108179 | PPIF | 10105 | 11,122.691 | 0.412 | 0.111 | 3,726 | 0.0002 | 0.0041 | NA |
| ENSG00000169504 | CLIC4 | 25932 | 14,879.995 | 0.413 | 0.105 | 3,929 | 0.0001 | 0.0021 | NA |
| ENSG00000136152 | COG3 | 83548 | 866,807 | 0.414 | 0.106 | 3,899 | 0.0001 | 0.0023 | NA |
| ENSG00000175643 | RMI2 | 116028 | 325,698 | 0.414 | 0.148 | 2,803 | 0.0051 | 0.0562 | NA |
| ENSG00000136997 | MYC | 4609 | 2,096.300 | 0.415 | 0.116 | 3,580 | 0.0003 | 0.0066 | NA |
| ENSG00000132170 | PPARG | 5468 | 943,590 | 0.415 | 0.126 | 3,294 | 0.0010 | 0.0155 | NA |
| ENSG00000140853 | NLRC5 | 84166 | 3,038.534 | 0.416 | 0.143 | 2,904 | 0.0037 | 0.0442 | NA |
| ENSG00000023171 | GRAMD1B | 57476 | 1,017.134 | 0.417 | 0.111 | 3,754 | 0.0002 | 0.0038 | NA |
| ENSG00000178105 | DDX10 | 1662 | 585,688 | 0.417 | 0.148 | 2,812 | 0.0049 | 0.0552 | NA |
| ENSG00000106346 | USP42 | 84132 | 479,245 | 0.417 | 0.149 | 2,805 | 0.0050 | 0.0559 | NA |
| ENSG00000132256 | TRIM5 | 85363 | 2,190.146 | 0.417 | 0.162 | 2,575 | 0.0100 | 0.0909 | NA |
| ENSG00000113319 | RASGRF2 | 5924 | 1,451.906 | 0.418 | 0.109 | 3,848 | 0.0001 | 0.0027 | NA |
| ENSG00000082153 | BZW1 | 9689 | 7,847.432 | 0.418 | 0.117 | 3,583 | 0.0003 | 0.0065 | NA |
| ENSG00000197608 | ZNF841 | 284371 | 456,417 | 0.418 | 0.124 | 3,363 | 0.0008 | 0.0128 | NA |
| ENSG00000188786 | MTF1 | 4520 | 1,050.529 | 0.419 | 0.101 | 4,153 | 0.0000 | 0.0009 | NA |
| ENSG00000141664 | ZCCHC2 | 54877 | 968,049 | 0.419 | 0.132 | 3,178 | 0.0015 | 0.0217 | NA |
| ENSG00000197646 | PDCD1LG2 | 80380 | 1,677.916 | 0.419 | 0.146 | 2,881 | 0.0040 | 0.0467 | NA |
| ENSG00000065911 | MTHFD2 | 10797 | 1,356.601 | 0.420 | 0.133 | 3,165 | 0.0016 | 0.0226 | NA |
| ENSG00000118473 | SGIP1 | 84251 | 1,365.414 | 0.420 | 0.133 | 3,155 | 0.0016 | 0.0231 | NA |
| ENSG00000086712 | TXLNG | 55787 | 508,180 | 0.421 | 0.142 | 2,970 | 0.0030 | 0.0375 | NA |
| ENSG00000151229 | SLC2A13 | 114134 | 369,616 | 0.422 | 0.139 | 3,047 | 0.0023 | 0.0306 | NA |
| ENSG00000168785 | TSPAN5 | 10098 | 3,214.707 | 0.423 | 0.096 | 4,401 | 0.0000 | 0.0004 | NA |
| ENSG00000145780 | FEM1C | 56929 | 1,175.438 | 0.424 | 0.128 | 3,301 | 0.0010 | 0.0152 | NA |
| ENSG00000153250 | RBMS1 | 5937 | 4,908.318 | 0.426 | 0.102 | 4,166 | 0.0000 | 0.0009 | NA |
| ENSG00000118503 | TNFAIP3 | 7128 | 5,567.005 | 0.427 | 0.158 | 2,700 | 0.0069 | 0.0713 | NA |
| ENSG00000267774 |  |  | 886,900 | 0.427 | 0.168 | 2,545 | 0.0109 | 0.0974 | NA |
| ENSG00000214114 | MYCBP | 26292 | 282,974 | 0.428 | 0.148 | 2,884 | 0.0039 | 0.0464 | NA |
| ENSG00000172716 | SLFN11 | 91607 | 867,592 | 0.429 | 0.109 | 3,924 | 0.0001 | 0.0021 | NA |
| ENSG00000146373 | RNF217 | 154214 | 400,169 | 0.430 | 0.140 | 3,061 | 0.0022 | 0.0295 | NA |
| ENSG00000247315 | ZCCHC3 | 85364 | 724,550 | 0.432 | 0.126 | 3,432 | 0.0006 | 0.0103 | NA |
| ENSG00000077063 | CTTNBP2 | 83992 | 203,993 | 0.433 | 0.159 | 2,721 | 0.0065 | 0.0675 | NA |
| ENSG00000087116 | ADAMTS2 | 9509 | 7,680.951 | 0.434 | 0.116 | 3,754 | 0.0002 | 0.0038 | NA |
| ENSG00000052802 | MSMO1 | 6307 | 1,509.097 | 0.434 | 0.140 | 3,109 | 0.0019 | 0.0260 | NA |
| ENSG00000081803 | CADPS2 | 93664 | 325,661 | 0.436 | 0.142 | 3,074 | 0.0021 | 0.0285 | NA |
| ENSG00000133657 | ATP13A3 | 79572 | 9,657.383 | 0.437 | 0.117 | 3,734 | 0.0002 | 0.0040 | NA |
| ENSG00000082482 | KCNK2 | 3776 | 2,757.802 | 0.437 | 0.121 | 3,598 | 0.0003 | 0.0062 | NA |
| ENSG00000198874 | TYW1 | 55253 | 651,004 | 0.438 | 0.105 | 4,160 | 0.0000 | 0.0009 | NA |
| ENSG00000100697 | DICER1 | 23405 | 1,732.319 | 0.438 | 0.152 | 2,870 | 0.0041 | 0.0477 | NA |
| ENSG00000006432 | MAP3K9 | 4293 | 309,913 | 0.438 | 0.158 | 2,767 | 0.0057 | 0.0609 | NA |
| ENSG00000172795 | DCP2 | 167227 | 537,159 | 0.439 | 0.143 | 3,063 | 0.0022 | 0.0293 | NA |
| ENSG00000075618 | FSCN1 | 6624 | 12,225.557 | 0.441 | 0.123 | 3,568 | 0.0004 | 0.0068 | NA |
| ENSG00000108846 | ABCC3 | 8714 | 638,742 | 0.441 | 0.159 | 2,773 | 0.0055 | 0.0602 | NA |
| ENSG00000120526 | NUDCD1 | 84955 | 1,349.154 | 0.442 | 0.104 | 4,249 | 0.0000 | 0.0007 | NA |
| ENSG00000162702 | ZNF281 | 23528 | 910,161 | 0.442 | 0.114 | 3,890 | 0.0001 | 0.0024 | NA |
| ENSG00000067064 | IDI1 | 3422 | 1,844.460 | 0.442 | 0.129 | 3,430 | 0.0006 | 0.0104 | NA |
| ENSG00000102755 | FLT1 | 2321 | 450,564 | 0.442 | 0.157 | 2,808 | 0.0050 | 0.0557 | NA |
| ENSG00000134970 | TMED7 | 51014 | 1,120.004 | 0.442 | 0.160 | 2,752 | 0.0059 | 0.0633 | NA |
| ENSG00000059378 | PARP12 | 64761 | 2,707.652 | 0.443 | 0.118 | 3,753 | 0.0002 | 0.0038 | NA |
| ENSG00000157600 | TMEM164 | 84187 | 1,166.691 | 0.444 | 0.101 | 4,397 | 0.0000 | 0.0004 | NA |
| ENSG00000150630 | VEGFC | 7424 | 5,513.316 | 0.445 | 0.128 | 3,462 | 0.0005 | 0.0095 | NA |
| ENSG00000132357 | CARD6 | 84674 | 1,028.613 | 0.445 | 0.142 | 3,135 | 0.0017 | 0.0243 | NA |
| ENSG00000171365 | CLCN5 | 1184 | 856,183 | 0.445 | 0.143 | 3,123 | 0.0018 | 0.0251 | NA |
| ENSG00000124151 | NCOA3 | 8202 | 1,998.481 | 0.446 | 0.108 | 4,134 | 0.0000 | 0.0010 | NA |
| ENSG00000227051 | C14orf132 | 56967 | 3,238.262 | 0.447 | 0.103 | 4,342 | 0.0000 | 0.0004 | NA |
| ENSG00000008513 | ST3GAL1 | 6482 | 3,484.056 | 0.447 | 0.119 | 3,753 | 0.0002 | 0.0038 | NA |
| ENSG00000154124 | OTULIN | 90268 | 861,210 | 0.448 | 0.114 | 3,919 | 0.0001 | 0.0021 | NA |
| ENSG00000160613 | PCSK7 | 9159 | 1,754.941 | 0.449 | 0.118 | 3,807 | 0.0001 | 0.0032 | NA |
| ENSG00000185507 | IRF7 | 3665 | 1,582.658 | 0.449 | 0.170 | 2,636 | 0.0084 | 0.0815 | NA |
| ENSG00000183287 | CCBE1 | 147372 | 12,997.560 | 0.450 | 0.114 | 3,940 | 0.0001 | 0.0020 | NA |
| ENSG00000104549 | SQLE | 6713 | 3,589.744 | 0.455 | 0.119 | 3,826 | 0.0001 | 0.0030 | NA |
| ENSG00000152256 | PDK1 | 5163 | 415,230 | 0.455 | 0.121 | 3,742 | 0.0002 | 0.0039 | NA |
| ENSG00000196449 | YRDC | 79693 | 696,718 | 0.456 | 0.131 | 3,483 | 0.0005 | 0.0088 | NA |
| ENSG00000117500 | TMED5 | 50999 | 1,188.768 | 0.456 | 0.136 | 3,357 | 0.0008 | 0.0130 | NA |
| ENSG00000170340 | B3GNT2 | 10678 | 743,133 | 0.457 | 0.132 | 3,459 | 0.0005 | 0.0096 | NA |
| ENSG00000198121 | LPAR1 | 1902 | 10,470.788 | 0.458 | 0.091 | 5,006 | 0.0000 | 0.0000 | NA |
| ENSG00000105829 | BET1 | 10282 | 1,009.497 | 0.458 | 0.117 | 3,924 | 0.0001 | 0.0021 | NA |
| ENSG00000181381 | DDX60L | 91351 | 3,662.985 | 0.458 | 0.146 | 3,131 | 0.0017 | 0.0246 | NA |
| ENSG00000114999 | TTL | 150465 | 3,313.416 | 0.459 | 0.122 | 3,776 | 0.0002 | 0.0035 | NA |
| ENSG00000110422 | HIPK3 | 10114 | 1,845.665 | 0.459 | 0.123 | 3,724 | 0.0002 | 0.0041 | NA |
| ENSG00000179314 | WSCD1 | 23302 | 786,940 | 0.459 | 0.161 | 2,861 | 0.0042 | 0.0491 | NA |
| ENSG00000119899 | SLC17A5 | 26503 | 4,473.484 | 0.461 | 0.102 | 4,498 | 0.0000 | 0.0002 | NA |
| ENSG00000231924 | PSG1 | 5669 | 492,586 | 0.462 | 0.140 | 3,295 | 0.0010 | 0.0155 | NA |
| ENSG00000126391 | FRMD8 | 83786 | 3,923.380 | 0.463 | 0.128 | 3,604 | 0.0003 | 0.0061 | NA |
| ENSG00000163348 | PYGO2 | 90780 | 987,353 | 0.464 | 0.094 | 4,910 | 0.0000 | 0.0000 | NA |
| ENSG00000133313 | CNDP2 | 55748 | 4,817.207 | 0.464 | 0.099 | 4,709 | 0.0000 | 0.0001 | NA |
| ENSG00000146858 | ZC3HAV1L | 92092 | 293,146 | 0.464 | 0.144 | 3,224 | 0.0013 | 0.0191 | NA |
| ENSG00000119661 | DNAL1 | 83544 | 420,134 | 0.466 | 0.151 | 3,091 | 0.0020 | 0.0274 | NA |
| ENSG00000215301 | DDX3X | 1654 | 9,926.282 | 0.467 | 0.118 | 3,972 | 0.0001 | 0.0018 | NA |
| ENSG00000074527 | NTN4 | 59277 | 3,628.196 | 0.467 | 0.124 | 3,757 | 0.0002 | 0.0037 | NA |
| ENSG00000117228 | GBP1 | 2633 | 4,956.354 | 0.467 | 0.155 | 3,006 | 0.0026 | 0.0340 | NA |
| ENSG00000139668 | WDFY2 | 115825 | 1,645.302 | 0.469 | 0.095 | 4,957 | 0.0000 | 0.0000 | NA |
| ENSG00000090530 | P3H2 | 55214 | 1,395.968 | 0.472 | 0.113 | 4,183 | 0.0000 | 0.0008 | NA |
| ENSG00000008086 | CDKL5 | 6792 | 356,034 | 0.472 | 0.139 | 3,396 | 0.0007 | 0.0115 | NA |
| ENSG00000163644 | PPM1K | 152926 | 838,956 | 0.472 | 0.160 | 2,957 | 0.0031 | 0.0386 | NA |
| ENSG00000283041 | LOC729998 | 729998 | 127,616 | 0.473 | 0.187 | 2,533 | 0.0113 | 0.0992 | NA |
| ENSG00000100320 | RBFOX2 | 23543 | 7,336.282 | 0.474 | 0.089 | 5,352 | 0.0000 | 0.0000 | NA |
| ENSG00000171246 | NPTX1 | 4884 | 12,138.271 | 0.474 | 0.109 | 4,331 | 0.0000 | 0.0005 | NA |
| ENSG00000008083 | JARID2 | 3720 | 600,717 | 0.476 | 0.133 | 3,579 | 0.0003 | 0.0066 | NA |
| ENSG00000106392 | C1GALT1 | 56913 | 1,346.032 | 0.476 | 0.135 | 3,536 | 0.0004 | 0.0075 | NA |
| ENSG00000127452 | FBXL12 | 54850 | 1,031.352 | 0.476 | 0.136 | 3,503 | 0.0005 | 0.0083 | NA |
| ENSG00000076108 | BAZ2A | 11176 | 5,225.133 | 0.477 | 0.114 | 4,172 | 0.0000 | 0.0009 | NA |
| ENSG00000107443 | CCNJ | 54619 | 403,114 | 0.477 | 0.134 | 3,560 | 0.0004 | 0.0070 | NA |
| ENSG00000096968 | JAK2 | 3717 | 593,417 | 0.477 | 0.142 | 3,347 | 0.0008 | 0.0134 | NA |
| ENSG00000144655 | CSRNP1 | 64651 | 568,268 | 0.477 | 0.158 | 3,024 | 0.0025 | 0.0325 | NA |
| ENSG00000260196 |  |  | 273,636 | 0.479 | 0.148 | 3,246 | 0.0012 | 0.0178 | NA |
| ENSG00000179431 | FJX1 | 24147 | 3,642.919 | 0.479 | 0.161 | 2,974 | 0.0029 | 0.0370 | NA |
| ENSG00000111266 | DUSP16 | 80824 | 471,263 | 0.480 | 0.145 | 3,301 | 0.0010 | 0.0152 | NA |
| ENSG00000223960 | CHROMR | 1E+08 | 268,566 | 0.480 | 0.174 | 2,760 | 0.0058 | 0.0619 | NA |
| ENSG00000004864 | SLC25A13 | 10165 | 1,064.478 | 0.482 | 0.122 | 3,937 | 0.0001 | 0.0020 | NA |
| ENSG00000139218 | SCAF11 | 9169 | 2,462.130 | 0.483 | 0.113 | 4,291 | 0.0000 | 0.0006 | NA |
| ENSG00000143126 | CELSR2 | 1952 | 232,630 | 0.483 | 0.165 | 2,923 | 0.0035 | 0.0419 | NA |
| ENSG00000114127 | XRN1 | 54464 | 1,737.582 | 0.485 | 0.112 | 4,322 | 0.0000 | 0.0005 | NA |
| ENSG00000257219 | LNCOG | 1.1E+08 | 354,965 | 0.485 | 0.167 | 2,899 | 0.0037 | 0.0446 | NA |
| ENSG00000106070 | GRB10 | 2887 | 1,148.774 | 0.486 | 0.124 | 3,918 | 0.0001 | 0.0022 | NA |
| ENSG00000089127 | OAS1 | 4938 | 5,695.647 | 0.486 | 0.167 | 2,912 | 0.0036 | 0.0432 | NA |
| ENSG00000148426 | PROSER2 | 254427 | 238,437 | 0.486 | 0.177 | 2,751 | 0.0059 | 0.0634 | NA |
| ENSG00000176018 | LYSMD3 | 116068 | 518,550 | 0.487 | 0.180 | 2,699 | 0.0070 | 0.0714 | NA |
| ENSG00000139697 | SBNO1 | 55206 | 1,821.042 | 0.488 | 0.111 | 4,396 | 0.0000 | 0.0004 | NA |
| ENSG00000087448 | KLHL42 | 57542 | 861,283 | 0.490 | 0.111 | 4,409 | 0.0000 | 0.0003 | NA |
| ENSG00000112237 | CCNC | 892 | 868,285 | 0.490 | 0.129 | 3,794 | 0.0001 | 0.0033 | NA |
| ENSG00000198959 | TGM2 | 7052 | 3,297.167 | 0.490 | 0.147 | 3,342 | 0.0008 | 0.0136 | NA |
| ENSG00000136146 | MED4 | 29079 | 444,377 | 0.492 | 0.151 | 3,269 | 0.0011 | 0.0167 | NA |
| ENSG00000155744 | FAM126B | 285172 | 405,377 | 0.492 | 0.151 | 3,255 | 0.0011 | 0.0174 | NA |
| ENSG00000121769 | FABP3 | 2170 | 1,255.184 | 0.492 | 0.181 | 2,727 | 0.0064 | 0.0666 | NA |
| ENSG00000054793 | ATP9A | 10079 | 1,868.937 | 0.495 | 0.105 | 4,733 | 0.0000 | 0.0001 | NA |
| ENSG00000065559 | MAP2K4 | 6416 | 1,229.087 | 0.498 | 0.127 | 3,906 | 0.0001 | 0.0022 | NA |
| ENSG00000184402 | SS18L1 | 26039 | 371,808 | 0.499 | 0.131 | 3,794 | 0.0001 | 0.0033 | NA |
| ENSG00000133466 | C1QTNF6 | 114904 | 517,657 | 0.499 | 0.164 | 3,042 | 0.0024 | 0.0310 | NA |
| ENSG00000137075 | RNF38 | 152006 | 810,286 | 0.500 | 0.125 | 4,010 | 0.0001 | 0.0016 | NA |
| ENSG00000117525 | F3 | 2152 | 780,563 | 0.501 | 0.158 | 3,174 | 0.0015 | 0.0220 | NA |
| ENSG00000100426 | ZBED4 | 9889 | 1,044.765 | 0.503 | 0.109 | 4,604 | 0.0000 | 0.0002 | NA |
| ENSG00000006625 | GGCT | 79017 | 306,392 | 0.503 | 0.146 | 3,433 | 0.0006 | 0.0103 | NA |
| ENSG00000106100 | NOD1 | 10392 | 251,442 | 0.503 | 0.158 | 3,190 | 0.0014 | 0.0211 | NA |
| ENSG00000187164 | SHTN1 | 57698 | 116,842 | 0.503 | 0.196 | 2,570 | 0.0102 | 0.0921 | NA |
| ENSG00000158480 | SPATA2 | 9825 | 812,848 | 0.505 | 0.114 | 4,419 | 0.0000 | 0.0003 | NA |
| ENSG00000174574 | AKIRIN1 | 79647 | 2,673.772 | 0.506 | 0.086 | 5,884 | 0.0000 | 0.0000 | NA |
| ENSG00000143507 | DUSP10 | 11221 | 1,569.781 | 0.507 | 0.142 | 3,569 | 0.0004 | 0.0068 | NA |
| ENSG00000147852 | VLDLR | 7436 | 331,762 | 0.508 | 0.147 | 3,442 | 0.0006 | 0.0101 | NA |
| ENSG00000136379 | ABHD17C | 58489 | 427,806 | 0.509 | 0.145 | 3,504 | 0.0005 | 0.0083 | NA |
| ENSG00000105447 | GRWD1 | 83743 | 2,135.444 | 0.511 | 0.120 | 4,272 | 0.0000 | 0.0006 | NA |
| ENSG00000130775 | THEMIS2 | 9473 | 1,202.355 | 0.511 | 0.198 | 2,585 | 0.0097 | 0.0893 | NA |
| ENSG00000080345 | RIF1 | 55183 | 1,324.746 | 0.513 | 0.140 | 3,673 | 0.0002 | 0.0049 | NA |
| ENSG00000143028 | SYPL2 | 284612 | 177,502 | 0.514 | 0.172 | 2,992 | 0.0028 | 0.0353 | NA |
| ENSG00000136158 | SPRY2 | 10253 | 3,500.720 | 0.515 | 0.124 | 4,153 | 0.0000 | 0.0009 | NA |
| ENSG00000138757 | G3BP2 | 9908 | 4,524.706 | 0.516 | 0.115 | 4,490 | 0.0000 | 0.0003 | NA |
| ENSG00000049192 | ADAMTS6 | 11174 | 482,835 | 0.519 | 0.177 | 2,928 | 0.0034 | 0.0415 | NA |
| ENSG00000135503 | ACVR1B | 91 | 753,173 | 0.520 | 0.118 | 4,392 | 0.0000 | 0.0004 | NA |
| ENSG00000164327 | RICTOR | 253260 | 1,007.646 | 0.521 | 0.131 | 3,983 | 0.0001 | 0.0017 | NA |
| ENSG00000125430 | HS3ST3B1 | 9953 | 823,507 | 0.521 | 0.153 | 3,408 | 0.0007 | 0.0111 | NA |
| ENSG00000168769 | TET2 | 54790 | 459,629 | 0.522 | 0.143 | 3,647 | 0.0003 | 0.0053 | NA |
| ENSG00000158161 | EYA3 | 2140 | 954,578 | 0.524 | 0.127 | 4,121 | 0.0000 | 0.0010 | NA |
| ENSG00000125266 | EFNB2 | 1948 | 534,909 | 0.524 | 0.196 | 2,677 | 0.0074 | 0.0750 | NA |
| ENSG00000166900 | STX3 | 6809 | 2,627.950 | 0.525 | 0.094 | 5,604 | 0.0000 | 0.0000 | NA |
| ENSG00000134954 | ETS1 | 2113 | 7,903.753 | 0.526 | 0.091 | 5,775 | 0.0000 | 0.0000 | NA |
| ENSG00000187605 | TET3 | 200424 | 544,779 | 0.526 | 0.148 | 3,554 | 0.0004 | 0.0071 | NA |
| ENSG00000169946 | ZFPM2 | 23414 | 229,229 | 0.526 | 0.174 | 3,023 | 0.0025 | 0.0326 | NA |
| ENSG00000117523 | PRRC2C | 23215 | 7,122.394 | 0.527 | 0.130 | 4,052 | 0.0001 | 0.0013 | NA |
| ENSG00000155660 | PDIA4 | 9601 | 7,959.986 | 0.528 | 0.104 | 5,087 | 0.0000 | 0.0000 | NA |
| ENSG00000130589 | HELZ2 | 85441 | 9,030.613 | 0.528 | 0.169 | 3,117 | 0.0018 | 0.0255 | NA |
| ENSG00000171658 | NMRAL2P | 344887 | 341,541 | 0.528 | 0.171 | 3,083 | 0.0021 | 0.0280 | NA |
| ENSG00000174839 | DENND6A | 201627 | 317,734 | 0.530 | 0.147 | 3,616 | 0.0003 | 0.0058 | NA |
| ENSG00000140943 | MBTPS1 | 8720 | 4,982.017 | 0.531 | 0.081 | 6,550 | 0.0000 | 0.0000 | NA |
| ENSG00000260910 |  |  | 217,998 | 0.533 | 0.183 | 2,920 | 0.0035 | 0.0422 | NA |
| ENSG00000113657 | DPYSL3 | 1809 | 8,479.197 | 0.534 | 0.107 | 4,991 | 0.0000 | 0.0000 | NA |
| ENSG00000135828 | RNASEL | 6041 | 431,461 | 0.535 | 0.139 | 3,854 | 0.0001 | 0.0027 | NA |
| ENSG00000162407 | PLPP3 | 8613 | 8,443.619 | 0.536 | 0.088 | 6,115 | 0.0000 | 0.0000 | NA |
| ENSG00000205730 | ITPRIPL2 | 162073 | 2,287.508 | 0.536 | 0.099 | 5,439 | 0.0000 | 0.0000 | NA |
| ENSG00000102024 | PLS3 | 5358 | 6,499.648 | 0.536 | 0.120 | 4,466 | 0.0000 | 0.0003 | NA |
| ENSG00000261115 | TMEM178B | 1E+08 | 1,338.851 | 0.536 | 0.127 | 4,234 | 0.0000 | 0.0007 | NA |
| ENSG00000152104 | PTPN14 | 5784 | 4,056.588 | 0.537 | 0.140 | 3,837 | 0.0001 | 0.0029 | NA |
| ENSG00000140280 | LYSMD2 | 256586 | 344,310 | 0.537 | 0.153 | 3,509 | 0.0004 | 0.0082 | NA |
| ENSG00000143147 | GPR161 | 23432 | 872,571 | 0.540 | 0.122 | 4,441 | 0.0000 | 0.0003 | NA |
| ENSG00000135905 | DOCK10 | 55619 | 2,875.642 | 0.540 | 0.124 | 4,362 | 0.0000 | 0.0004 | NA |
| ENSG00000143033 | MTF2 | 22823 | 405,994 | 0.540 | 0.169 | 3,206 | 0.0013 | 0.0201 | NA |
| ENSG00000261512 |  |  | 185,530 | 0.540 | 0.208 | 2,600 | 0.0093 | 0.0870 | NA |
| ENSG00000168398 | BDKRB2 | 624 | 3,256.363 | 0.541 | 0.180 | 2,999 | 0.0027 | 0.0347 | NA |
| ENSG00000164056 | SPRY1 | 10252 | 110,435 | 0.542 | 0.196 | 2,767 | 0.0057 | 0.0609 | NA |
| ENSG00000164080 | RAD54L2 | 23132 | 970,374 | 0.543 | 0.113 | 4,819 | 0.0000 | 0.0001 | NA |
| ENSG00000226380 |  |  | 847,348 | 0.543 | 0.134 | 4,053 | 0.0001 | 0.0013 | NA |
| ENSG00000165806 | CASP7 | 840 | 1,984.609 | 0.544 | 0.132 | 4,134 | 0.0000 | 0.0010 | NA |
| ENSG00000005884 | ITGA3 | 3675 | 13,447.000 | 0.544 | 0.157 | 3,458 | 0.0005 | 0.0096 | NA |
| ENSG00000164045 | CDC25A | 993 | 861,666 | 0.544 | 0.191 | 2,857 | 0.0043 | 0.0496 | NA |
| ENSG00000145685 | LHFPL2 | 10184 | 5,379.253 | 0.545 | 0.097 | 5,602 | 0.0000 | 0.0000 | NA |
| ENSG00000184988 | TMEM106A | 113277 | 714,908 | 0.545 | 0.149 | 3,669 | 0.0002 | 0.0050 | NA |
| ENSG00000164631 | ZNF12 | 7559 | 826,147 | 0.548 | 0.125 | 4,389 | 0.0000 | 0.0004 | NA |
| ENSG00000143469 | SYT14 | 255928 | 175,821 | 0.549 | 0.194 | 2,837 | 0.0045 | 0.0518 | NA |
| ENSG00000185129 | PURA | 5813 | 1,229.983 | 0.550 | 0.115 | 4,800 | 0.0000 | 0.0001 | NA |
| ENSG00000010030 | ETV7 | 51513 | 857,739 | 0.550 | 0.193 | 2,856 | 0.0043 | 0.0497 | NA |
| ENSG00000115271 | GCA | 25801 | 174,957 | 0.551 | 0.169 | 3,252 | 0.0011 | 0.0175 | NA |
| ENSG00000168876 | ANKRD49 | 54851 | 197,562 | 0.552 | 0.172 | 3,209 | 0.0013 | 0.0200 | NA |
| ENSG00000096696 | DSP | 1832 | 758,639 | 0.553 | 0.140 | 3,963 | 0.0001 | 0.0018 | NA |
| ENSG00000167772 | ANGPTL4 | 51129 | 505,428 | 0.555 | 0.189 | 2,938 | 0.0033 | 0.0405 | NA |
| ENSG00000157404 | KIT | 3815 | 488,363 | 0.555 | 0.190 | 2,928 | 0.0034 | 0.0415 | NA |
| ENSG00000113441 | LNPEP | 4012 | 1,795.390 | 0.557 | 0.117 | 4,744 | 0.0000 | 0.0001 | NA |
| ENSG00000145358 | DDIT4L | 115265 | 146,358 | 0.560 | 0.204 | 2,741 | 0.0061 | 0.0649 | NA |
| ENSG00000059758 | CDK17 | 5128 | 1,144.719 | 0.561 | 0.111 | 5,039 | 0.0000 | 0.0000 | NA |
| ENSG00000198113 | TOR4A | 54863 | 3,192.341 | 0.561 | 0.157 | 3,569 | 0.0004 | 0.0068 | NA |
| ENSG00000185989 | RASA3 | 22821 | 3,301.779 | 0.565 | 0.100 | 5,655 | 0.0000 | 0.0000 | NA |
| ENSG00000101972 | STAG2 | 10735 | 2,037.397 | 0.567 | 0.127 | 4,473 | 0.0000 | 0.0003 | NA |
| ENSG00000132664 | POLR3F | 10621 | 542,884 | 0.570 | 0.140 | 4,067 | 0.0000 | 0.0013 | NA |
| ENSG00000197008 | ZNF138 | 7697 | 143,433 | 0.571 | 0.195 | 2,926 | 0.0034 | 0.0416 | NA |
| ENSG00000196182 | STK40 | 83931 | 3,825.947 | 0.572 | 0.130 | 4,393 | 0.0000 | 0.0004 | NA |
| ENSG00000071537 | SEL1L | 6400 | 5,248.776 | 0.576 | 0.099 | 5,845 | 0.0000 | 0.0000 | NA |
| ENSG00000155858 | LSM11 | 134353 | 250,750 | 0.577 | 0.204 | 2,831 | 0.0046 | 0.0526 | NA |
| ENSG00000143190 | POU2F1 | 5451 | 240,711 | 0.580 | 0.172 | 3,380 | 0.0007 | 0.0121 | NA |
| ENSG00000128590 | DNAJB9 | 4189 | 684,594 | 0.582 | 0.125 | 4,661 | 0.0000 | 0.0001 | NA |
| ENSG00000101187 | SLCO4A1 | 28231 | 161,167 | 0.582 | 0.184 | 3,158 | 0.0016 | 0.0229 | NA |
| ENSG00000182768 | NGRN | 51335 | 1,386.501 | 0.583 | 0.113 | 5,145 | 0.0000 | 0.0000 | NA |
| ENSG00000139645 | ANKRD52 | 283373 | 8,627.679 | 0.583 | 0.109 | 5,340 | 0.0000 | 0.0000 | NA |
| ENSG00000151414 | NEK7 | 140609 | 4,493.687 | 0.585 | 0.132 | 4,430 | 0.0000 | 0.0003 | NA |
| ENSG00000128965 | CHAC1 | 79094 | 531,266 | 0.585 | 0.199 | 2,943 | 0.0033 | 0.0401 | NA |
| ENSG00000162892 | IL24 | 11009 | 1,405.642 | 0.585 | 0.205 | 2,853 | 0.0043 | 0.0500 | NA |
| ENSG00000163110 | PDLIM5 | 10611 | 4,771.478 | 0.586 | 0.144 | 4,079 | 0.0000 | 0.0012 | NA |
| ENSG00000175334 | BANF1 | 8815 | 3,277.417 | 0.589 | 0.094 | 6,299 | 0.0000 | 0.0000 | NA |
| ENSG00000234072 |  |  | 155,159 | 0.590 | 0.198 | 2,978 | 0.0029 | 0.0366 | NA |
| ENSG00000006459 | KDM7A | 80853 | 121,224 | 0.590 | 0.227 | 2,597 | 0.0094 | 0.0876 | NA |
| ENSG00000189266 | PNRC2 | 55629 | 2,591.142 | 0.591 | 0.107 | 5,548 | 0.0000 | 0.0000 | NA |
| ENSG00000030419 | IKZF2 | 22807 | 125,934 | 0.591 | 0.216 | 2,741 | 0.0061 | 0.0649 | NA |
| ENSG00000164211 | STARD4 | 134429 | 926,145 | 0.592 | 0.129 | 4,587 | 0.0000 | 0.0002 | NA |
| ENSG00000164983 | TMEM65 | 157378 | 513,321 | 0.592 | 0.158 | 3,752 | 0.0002 | 0.0038 | NA |
| ENSG00000240694 | PNMA2 | 10687 | 468,432 | 0.593 | 0.143 | 4,160 | 0.0000 | 0.0009 | NA |
| ENSG00000249915 | PDCD6 | 10016 | 2,304.234 | 0.595 | 0.092 | 6,446 | 0.0000 | 0.0000 | NA |
| ENSG00000189410 | SH2D5 | 400745 | 2,132.126 | 0.596 | 0.154 | 3,875 | 0.0001 | 0.0025 | NA |
| ENSG00000002745 | WNT16 | 51384 | 677,097 | 0.599 | 0.170 | 3,520 | 0.0004 | 0.0079 | NA |
| ENSG00000173011 | TADA2B | 93624 | 1,087.348 | 0.600 | 0.113 | 5,298 | 0.0000 | 0.0000 | NA |
| ENSG00000173334 | TRIB1 | 10221 | 687,035 | 0.600 | 0.129 | 4,664 | 0.0000 | 0.0001 | NA |
| ENSG00000197457 | STMN3 | 50861 | 741,495 | 0.600 | 0.137 | 4,394 | 0.0000 | 0.0004 | NA |
| ENSG00000181938 | GINS3 | 64785 | 320,136 | 0.600 | 0.183 | 3,274 | 0.0011 | 0.0165 | NA |
| ENSG00000143067 | ZNF697 | 90874 | 663,972 | 0.601 | 0.149 | 4,047 | 0.0001 | 0.0014 | NA |
| ENSG00000175906 | ARL4D | 379 | 1,304.132 | 0.603 | 0.124 | 4,851 | 0.0000 | 0.0001 | NA |
| ENSG00000171150 | SOCS5 | 9655 | 1,421.343 | 0.609 | 0.111 | 5,461 | 0.0000 | 0.0000 | NA |
| ENSG00000165312 | OTUD1 | 220213 | 161,942 | 0.609 | 0.191 | 3,185 | 0.0014 | 0.0213 | NA |
| ENSG00000228649 | SNHG26 | 1.1E+08 | 156,070 | 0.612 | 0.199 | 3,077 | 0.0021 | 0.0283 | NA |
| ENSG00000159314 | ARHGAP27 | 201176 | 536,635 | 0.612 | 0.218 | 2,806 | 0.0050 | 0.0559 | NA |
| ENSG00000136231 | IGF2BP3 | 10643 | 1,818.047 | 0.614 | 0.101 | 6,087 | 0.0000 | 0.0000 | NA |
| ENSG00000131127 | ZNF141 | 7700 | 141,074 | 0.616 | 0.200 | 3,082 | 0.0021 | 0.0280 | NA |
| ENSG00000163545 | NUAK2 | 81788 | 349,302 | 0.620 | 0.158 | 3,917 | 0.0001 | 0.0022 | NA |
| ENSG00000181751 | MACIR | 90355 | 455,317 | 0.620 | 0.161 | 3,840 | 0.0001 | 0.0028 | NA |
| ENSG00000166446 | CDYL2 | 124359 | 613,696 | 0.621 | 0.126 | 4,915 | 0.0000 | 0.0000 | NA |
| ENSG00000108578 | BLMH | 642 | 1,052.930 | 0.621 | 0.101 | 6,147 | 0.0000 | 0.0000 | NA |
| ENSG00000176692 | FOXC2 | 2303 | 346,366 | 0.626 | 0.209 | 2,988 | 0.0028 | 0.0356 | NA |
| ENSG00000123685 | BATF3 | 55509 | 138,632 | 0.626 | 0.245 | 2,553 | 0.0107 | 0.0956 | NA |
| ENSG00000071575 | TRIB2 | 28951 | 641,070 | 0.627 | 0.124 | 5,051 | 0.0000 | 0.0000 | NA |
| ENSG00000106123 | EPHB6 | 2051 | 117,040 | 0.630 | 0.220 | 2,872 | 0.0041 | 0.0477 | NA |
| ENSG00000100596 | SPTLC2 | 9517 | 1,932.427 | 0.632 | 0.112 | 5,646 | 0.0000 | 0.0000 | NA |
| ENSG00000203727 | SAMD5 | 389432 | 237,199 | 0.632 | 0.173 | 3,643 | 0.0003 | 0.0054 | NA |
| ENSG00000117758 | STX12 | 23673 | 2,130.892 | 0.633 | 0.120 | 5,269 | 0.0000 | 0.0000 | NA |
| ENSG00000165997 | ARL5B | 221079 | 736,075 | 0.633 | 0.142 | 4,442 | 0.0000 | 0.0003 | NA |
| ENSG00000179051 | RCC2 | 55920 | 2,910.543 | 0.636 | 0.094 | 6,791 | 0.0000 | 0.0000 | NA |
| ENSG00000165105 | RASEF | 158158 | 361,623 | 0.637 | 0.156 | 4,091 | 0.0000 | 0.0012 | NA |
| ENSG00000167193 | CRK | 1398 | 4,618.447 | 0.638 | 0.092 | 6,972 | 0.0000 | 0.0000 | NA |
| ENSG00000188211 | NCR3LG1 | 374383 | 1,425.957 | 0.638 | 0.110 | 5,786 | 0.0000 | 0.0000 | NA |
| ENSG00000137936 | BCAR3 | 8412 | 2,944.940 | 0.639 | 0.126 | 5,063 | 0.0000 | 0.0000 | NA |
| ENSG00000136514 | RTP4 | 64108 | 617,646 | 0.640 | 0.203 | 3,150 | 0.0016 | 0.0234 | NA |
| ENSG00000223551 | TMSB4XP4 | 7118 | 107,958 | 0.640 | 0.224 | 2,864 | 0.0042 | 0.0486 | NA |
| ENSG00000189134 | NKAPL | 222698 | 133,005 | 0.641 | 0.218 | 2,942 | 0.0033 | 0.0402 | NA |
| ENSG00000250312 | ZNF718 | 255403 | 155,804 | 0.644 | 0.216 | 2,983 | 0.0029 | 0.0360 | NA |
| ENSG00000138166 | DUSP5 | 1847 | 2,276.144 | 0.647 | 0.115 | 5,634 | 0.0000 | 0.0000 | NA |
| ENSG00000240583 | AQP1 | 358 | 468,862 | 0.649 | 0.200 | 3,248 | 0.0012 | 0.0177 | NA |
| ENSG00000113161 | HMGCR | 3156 | 2,711.773 | 0.657 | 0.131 | 5,023 | 0.0000 | 0.0000 | NA |
| ENSG00000156171 | DRAM2 | 128338 | 422,813 | 0.657 | 0.147 | 4,463 | 0.0000 | 0.0003 | NA |
| ENSG00000188070 | C11orf95 | 65998 | 1,816.047 | 0.658 | 0.103 | 6,388 | 0.0000 | 0.0000 | NA |
| ENSG00000169715 | MT1E | 4493 | 2,667.923 | 0.661 | 0.179 | 3,689 | 0.0002 | 0.0047 | NA |
| ENSG00000137449 | CPEB2 | 132864 | 2,180.884 | 0.662 | 0.101 | 6,529 | 0.0000 | 0.0000 | NA |
| ENSG00000246763 | RGMB-AS1 | 503569 | 874,823 | 0.664 | 0.144 | 4,628 | 0.0000 | 0.0001 | NA |
| ENSG00000028277 | POU2F2 | 5452 | 3,802.658 | 0.666 | 0.150 | 4,440 | 0.0000 | 0.0003 | NA |
| ENSG00000109046 | WSB1 | 26118 | 2,381.743 | 0.668 | 0.125 | 5,356 | 0.0000 | 0.0000 | NA |
| ENSG00000117152 | RGS4 | 5999 | 769,061 | 0.668 | 0.165 | 4,040 | 0.0001 | 0.0014 | NA |
| ENSG00000135048 | CEMIP2 | 23670 | 3,905.950 | 0.670 | 0.130 | 5,162 | 0.0000 | 0.0000 | NA |
| ENSG00000099337 | KCNK6 | 9424 | 1,779.463 | 0.670 | 0.130 | 5,152 | 0.0000 | 0.0000 | NA |
| ENSG00000099810 | MTAP | 4507 | 780,702 | 0.672 | 0.129 | 5,216 | 0.0000 | 0.0000 | NA |
| ENSG00000109220 | CHIC2 | 26511 | 702,889 | 0.681 | 0.119 | 5,749 | 0.0000 | 0.0000 | NA |
| ENSG00000136802 | LRRC8A | 56262 | 3,994.064 | 0.682 | 0.098 | 6,988 | 0.0000 | 0.0000 | NA |
| ENSG00000197223 | C1D | 10438 | 408,419 | 0.683 | 0.145 | 4,700 | 0.0000 | 0.0001 | NA |
| ENSG00000168404 | MLKL | 197259 | 1,102.134 | 0.685 | 0.135 | 5,061 | 0.0000 | 0.0000 | NA |
| ENSG00000006607 | FARP2 | 9855 | 1,498.876 | 0.688 | 0.141 | 4,869 | 0.0000 | 0.0000 | NA |
| ENSG00000013588 | GPRC5A | 9052 | 1,606.492 | 0.689 | 0.148 | 4,661 | 0.0000 | 0.0001 | NA |
| ENSG00000123700 | KCNJ2 | 3759 | 1,782.104 | 0.690 | 0.161 | 4,290 | 0.0000 | 0.0006 | NA |
| ENSG00000198642 | KLHL9 | 55958 | 1,752.445 | 0.692 | 0.101 | 6,855 | 0.0000 | 0.0000 | NA |
| ENSG00000153234 | NR4A2 | 4929 | 157,125 | 0.693 | 0.259 | 2,680 | 0.0074 | 0.0744 | NA |
| ENSG00000171345 | KRT19 | 3880 | 914,636 | 0.694 | 0.209 | 3,320 | 0.0009 | 0.0145 | NA |
| ENSG00000145919 | BOD1 | 91272 | 1,687.417 | 0.695 | 0.096 | 7,221 | 0.0000 | 0.0000 | NA |
| ENSG00000196459 | TRAPPC2 | 6399 | 251,298 | 0.697 | 0.144 | 4,831 | 0.0000 | 0.0001 | NA |
| ENSG00000152804 | HHEX | 3087 | 90,943 | 0.697 | 0.255 | 2,730 | 0.0063 | 0.0663 | NA |
| ENSG00000176697 | BDNF | 627 | 901,646 | 0.699 | 0.142 | 4,908 | 0.0000 | 0.0000 | NA |
| ENSG00000181274 | FRAT2 | 23401 | 185,620 | 0.703 | 0.186 | 3,789 | 0.0002 | 0.0033 | NA |
| ENSG00000162981 | LRATD1 | 151354 | 211,248 | 0.703 | 0.196 | 3,587 | 0.0003 | 0.0064 | NA |
| ENSG00000135899 | SP110 | 3431 | 238,294 | 0.703 | 0.212 | 3,310 | 0.0009 | 0.0149 | NA |
| ENSG00000113369 | ARRDC3 | 57561 | 1,978.348 | 0.706 | 0.096 | 7,347 | 0.0000 | 0.0000 | NA |
| ENSG00000176928 | GCNT4 | 51301 | 106,071 | 0.709 | 0.231 | 3,074 | 0.0021 | 0.0285 | NA |
| ENSG00000108854 | SMURF2 | 64750 | 9,420.221 | 0.712 | 0.127 | 5,596 | 0.0000 | 0.0000 | NA |
| ENSG00000139318 | DUSP6 | 1848 | 2,786.195 | 0.713 | 0.161 | 4,431 | 0.0000 | 0.0003 | NA |
| ENSG00000124882 | EREG | 2069 | 276,927 | 0.713 | 0.224 | 3,188 | 0.0014 | 0.0213 | NA |
| ENSG00000213073 | CHP1P2 | 729603 | 56,637 | 0.714 | 0.268 | 2,665 | 0.0077 | 0.0768 | NA |
| ENSG00000160712 | IL6R | 3570 | 839,345 | 0.720 | 0.140 | 5,140 | 0.0000 | 0.0000 | NA |
| ENSG00000132003 | ZSWIM4 | 65249 | 577,330 | 0.721 | 0.149 | 4,829 | 0.0000 | 0.0001 | NA |
| ENSG00000157823 | AP3S2 | 10239 | 451,100 | 0.722 | 0.133 | 5,420 | 0.0000 | 0.0000 | NA |
| ENSG00000182934 | SRPRA | 6734 | 9,707.821 | 0.725 | 0.091 | 7,955 | 0.0000 | 0.0000 | NA |
| ENSG00000107201 | DDX58 | 23586 | 6,999.519 | 0.727 | 0.276 | 2,640 | 0.0083 | 0.0809 | NA |
| ENSG00000173638 | SLC19A1 | 6573 | 474,911 | 0.728 | 0.167 | 4,362 | 0.0000 | 0.0004 | NA |
| ENSG00000173221 | GLRX | 2745 | 2,173.368 | 0.732 | 0.129 | 5,672 | 0.0000 | 0.0000 | NA |
| ENSG00000011007 | ELOA | 6924 | 3,921.960 | 0.733 | 0.109 | 6,747 | 0.0000 | 0.0000 | NA |
| ENSG00000185127 | C6orf120 | 387263 | 1,289.789 | 0.740 | 0.098 | 7,549 | 0.0000 | 0.0000 | NA |
| ENSG00000154727 | GABPA | 2551 | 522,766 | 0.741 | 0.138 | 5,376 | 0.0000 | 0.0000 | NA |
| ENSG00000113070 | HBEGF | 1839 | 962,491 | 0.742 | 0.143 | 5,206 | 0.0000 | 0.0000 | NA |
| ENSG00000059728 | MXD1 | 4084 | 397,668 | 0.743 | 0.152 | 4,893 | 0.0000 | 0.0000 | NA |
| ENSG00000104321 | TRPA1 | 8989 | 1,339.217 | 0.747 | 0.196 | 3,810 | 0.0001 | 0.0031 | NA |
| ENSG00000174136 | RGMB | 285704 | 6,511.717 | 0.749 | 0.108 | 6,935 | 0.0000 | 0.0000 | NA |
| ENSG00000174804 | FZD4 | 8322 | 628,894 | 0.750 | 0.154 | 4,854 | 0.0000 | 0.0001 | NA |
| ENSG00000134955 | SLC37A2 | 219855 | 301,480 | 0.752 | 0.183 | 4,108 | 0.0000 | 0.0011 | NA |
| ENSG00000087095 | NLK | 51701 | 476,257 | 0.754 | 0.117 | 6,432 | 0.0000 | 0.0000 | NA |
| ENSG00000073756 | PTGS2 | 5743 | 5,426.477 | 0.755 | 0.178 | 4,232 | 0.0000 | 0.0007 | NA |
| ENSG00000134899 | ERCC5 | 2073 | 418,816 | 0.757 | 0.133 | 5,710 | 0.0000 | 0.0000 | NA |
| ENSG00000163428 | LRRC58 | 116064 | 1,488.566 | 0.757 | 0.166 | 4,573 | 0.0000 | 0.0002 | NA |
| ENSG00000148572 | NRBF2 | 29982 | 935,898 | 0.758 | 0.119 | 6,379 | 0.0000 | 0.0000 | NA |
| ENSG00000022567 | SLC45A4 | 57210 | 238,113 | 0.759 | 0.155 | 4,896 | 0.0000 | 0.0000 | NA |
| ENSG00000082497 | SERTAD4 | 56256 | 85,345 | 0.759 | 0.282 | 2,695 | 0.0070 | 0.0719 | NA |
| ENSG00000151967 | SCHIP1 | 29970 | 59,544 | 0.769 | 0.277 | 2,775 | 0.0055 | 0.0600 | NA |
| ENSG00000272821 |  |  | 94,233 | 0.770 | 0.262 | 2,939 | 0.0033 | 0.0404 | NA |
| ENSG00000131149 | GSE1 | 23199 | 1,586.879 | 0.773 | 0.127 | 6,104 | 0.0000 | 0.0000 | NA |
| ENSG00000277534 |  |  | 56,537 | 0.777 | 0.273 | 2,845 | 0.0044 | 0.0508 | NA |
| ENSG00000163041 | H3-3A | 3020 | 1,298.295 | 0.783 | 0.109 | 7,208 | 0.0000 | 0.0000 | NA |
| ENSG00000180398 | MCFD2 | 90411 | 9,118.064 | 0.788 | 0.110 | 7,174 | 0.0000 | 0.0000 | NA |
| ENSG00000114698 | PLSCR4 | 57088 | 758,573 | 0.790 | 0.132 | 5,971 | 0.0000 | 0.0000 | NA |
| ENSG00000123094 | RASSF8 | 11228 | 3,084.084 | 0.792 | 0.138 | 5,751 | 0.0000 | 0.0000 | NA |
| ENSG00000112972 | HMGCS1 | 3157 | 2,114.036 | 0.792 | 0.145 | 5,475 | 0.0000 | 0.0000 | NA |
| ENSG00000198768 | APCDD1L | 164284 | 321,250 | 0.792 | 0.200 | 3,954 | 0.0001 | 0.0019 | NA |
| ENSG00000047932 | GOPC | 57120 | 906,017 | 0.794 | 0.122 | 6,506 | 0.0000 | 0.0000 | NA |
| ENSG00000143384 | MCL1 | 4170 | 10,658.824 | 0.795 | 0.089 | 8,945 | 0.0000 | 0.0000 | NA |
| ENSG00000174099 | MSRB3 | 253827 | 3,328.861 | 0.797 | 0.107 | 7,438 | 0.0000 | 0.0000 | NA |
| ENSG00000177283 | FZD8 | 8325 | 1,042.474 | 0.801 | 0.191 | 4,185 | 0.0000 | 0.0008 | NA |
| ENSG00000134532 | SOX5 | 6660 | 64,852 | 0.802 | 0.276 | 2,905 | 0.0037 | 0.0440 | NA |
| ENSG00000065613 | SLK | 9748 | 2,844.378 | 0.809 | 0.117 | 6,936 | 0.0000 | 0.0000 | NA |
| ENSG00000274211 | SOCS7 | 30837 | 587,580 | 0.812 | 0.124 | 6,572 | 0.0000 | 0.0000 | NA |
| ENSG00000088205 | DDX18 | 8886 | 2,072.504 | 0.812 | 0.121 | 6,706 | 0.0000 | 0.0000 | NA |
| ENSG00000133111 | RFXAP | 5994 | 75,296 | 0.812 | 0.272 | 2,989 | 0.0028 | 0.0356 | NA |
| ENSG00000198018 | ENTPD7 | 57089 | 2,354.541 | 0.813 | 0.113 | 7,204 | 0.0000 | 0.0000 | NA |
| ENSG00000163743 | RCHY1 | 25898 | 300,517 | 0.816 | 0.170 | 4,792 | 0.0000 | 0.0001 | NA |
| ENSG00000179862 | CITED4 | 163732 | 338,613 | 0.816 | 0.223 | 3,660 | 0.0003 | 0.0051 | NA |
| ENSG00000196227 | FAM217B | 63939 | 507,396 | 0.818 | 0.147 | 5,549 | 0.0000 | 0.0000 | NA |
| ENSG00000257218 | GATC | 283459 | 546,705 | 0.821 | 0.116 | 7,077 | 0.0000 | 0.0000 | NA |
| ENSG00000124466 | LYPD3 | 27076 | 405,446 | 0.822 | 0.175 | 4,705 | 0.0000 | 0.0001 | NA |
| ENSG00000120875 | DUSP4 | 1846 | 2,613.687 | 0.825 | 0.107 | 7,687 | 0.0000 | 0.0000 | NA |
| ENSG00000163818 | LZTFL1 | 54585 | 380,348 | 0.836 | 0.154 | 5,418 | 0.0000 | 0.0000 | NA |
| ENSG00000139174 | PRICKLE1 | 144165 | 92,714 | 0.837 | 0.241 | 3,469 | 0.0005 | 0.0093 | NA |
| ENSG00000163568 | AIM2 | 9447 | 335,638 | 0.842 | 0.266 | 3,160 | 0.0016 | 0.0228 | NA |
| ENSG00000144560 | VGLL4 | 9686 | 876,384 | 0.843 | 0.123 | 6,856 | 0.0000 | 0.0000 | NA |
| ENSG00000213281 | NRAS | 4893 | 2,948.261 | 0.844 | 0.130 | 6,488 | 0.0000 | 0.0000 | NA |
| ENSG00000103888 | CEMIP | 57214 | 2,559.172 | 0.844 | 0.330 | 2,556 | 0.0106 | 0.0950 | NA |
| ENSG00000134490 | TMEM241 | 85019 | 245,634 | 0.846 | 0.164 | 5,171 | 0.0000 | 0.0000 | NA |
| ENSG00000160233 | LRRC3 | 81543 | 293,613 | 0.855 | 0.204 | 4,183 | 0.0000 | 0.0008 | NA |
| ENSG00000187173 | LCE2A | 353139 | 164,301 | 0.855 | 0.246 | 3,470 | 0.0005 | 0.0093 | NA |
| ENSG00000189143 | CLDN4 | 1364 | 249,460 | 0.860 | 0.274 | 3,142 | 0.0017 | 0.0239 | NA |
| ENSG00000160013 | PTGIR | 5739 | 391,205 | 0.865 | 0.233 | 3,706 | 0.0002 | 0.0044 | NA |
| ENSG00000117020 | AKT3 | 10000 | 1,161.552 | 0.875 | 0.120 | 7,318 | 0.0000 | 0.0000 | NA |
| ENSG00000187678 | SPRY4 | 81848 | 1,057.007 | 0.898 | 0.148 | 6,089 | 0.0000 | 0.0000 | NA |
| ENSG00000172159 | FRMD3 | 257019 | 48,551 | 0.899 | 0.349 | 2,577 | 0.0100 | 0.0905 | NA |
| ENSG00000135002 | RFK | 55312 | 896,719 | 0.900 | 0.128 | 7,021 | 0.0000 | 0.0000 | NA |
| ENSG00000066583 | ISOC1 | 51015 | 306,497 | 0.901 | 0.170 | 5,289 | 0.0000 | 0.0000 | NA |
| ENSG00000117174 | ZNHIT6 | 54680 | 1,059.745 | 0.911 | 0.129 | 7,072 | 0.0000 | 0.0000 | NA |
| ENSG00000140406 | TLNRD1 | 59274 | 1,609.002 | 0.914 | 0.166 | 5,521 | 0.0000 | 0.0000 | NA |
| ENSG00000065320 | NTN1 | 9423 | 1,090.156 | 0.915 | 0.165 | 5,536 | 0.0000 | 0.0000 | NA |
| ENSG00000147421 | HMBOX1 | 79618 | 246,825 | 0.920 | 0.162 | 5,694 | 0.0000 | 0.0000 | NA |
| ENSG00000131737 | KRT34 | 3885 | 488,019 | 0.923 | 0.329 | 2,803 | 0.0051 | 0.0562 | NA |
| ENSG00000184564 | SLITRK6 | 84189 | 51,505 | 0.928 | 0.347 | 2,675 | 0.0075 | 0.0752 | NA |
| ENSG00000128617 | OPN1SW | 611 | 135,020 | 0.929 | 0.191 | 4,853 | 0.0000 | 0.0001 | NA |
| ENSG00000176641 | RNF152 | 220441 | 507,229 | 0.942 | 0.161 | 5,840 | 0.0000 | 0.0000 | NA |
| ENSG00000101384 | JAG1 | 182 | 1,025.948 | 0.942 | 0.130 | 7,234 | 0.0000 | 0.0000 | NA |
| ENSG00000128595 | CALU | 813 | 57,677.454 | 0.945 | 0.131 | 7,226 | 0.0000 | 0.0000 | NA |
| ENSG00000134326 | CMPK2 | 129607 | 5,004.227 | 0.952 | 0.312 | 3,057 | 0.0022 | 0.0298 | NA |
| ENSG00000196396 | PTPN1 | 5770 | 3,047.243 | 0.962 | 0.118 | 8,165 | 0.0000 | 0.0000 | NA |
| ENSG00000135378 | PRRG4 | 79056 | 58,113 | 0.981 | 0.367 | 2,672 | 0.0075 | 0.0758 | NA |
| ENSG00000173762 | CD7 | 924 | 83,825 | 0.984 | 0.320 | 3,078 | 0.0021 | 0.0283 | NA |
| ENSG00000106484 | MEST | 4232 | 113,477 | 1,002 | 0.313 | 3,199 | 0.0014 | 0.0205 | NA |
| ENSG00000018408 | WWTR1 | 25937 | 2,876.649 | 1,009 | 0.104 | 9,707 | 0.0000 | 0.0000 | NA |
| ENSG00000134716 | CYP2J2 | 1573 | 83,182 | 1,013 | 0.397 | 2,551 | 0.0108 | 0.0960 | NA |
| ENSG00000235655 |  |  | 309,153 | 1,016 | 0.157 | 6,456 | 0.0000 | 0.0000 | NA |
| ENSG00000077522 | ACTN2 | 88 | 57,720 | 1,022 | 0.341 | 2,996 | 0.0027 | 0.0350 | NA |
| ENSG00000163376 | KBTBD8 | 84541 | 94,316 | 1,027 | 0.268 | 3,833 | 0.0001 | 0.0029 | NA |
| ENSG00000117868 | ESYT2 | 57488 | 3,499.457 | 1,035 | 0.089 | 11,571 | 0.0000 | 0.0000 | NA |
| ENSG00000225492 | GBP1P1 | 400759 | 293,460 | 1,046 | 0.263 | 3,977 | 0.0001 | 0.0017 | NA |
| ENSG00000152056 | AP1S3 | 130340 | 180,845 | 1,046 | 0.228 | 4,589 | 0.0000 | 0.0002 | 70 |
| ENSG00000123643 | SLC36A1 | 206358 | 1,707.838 | 1,051 | 0.112 | 9,355 | 0.0000 | 0.0000 | 77 |
| ENSG00000173726 | TOMM20 | 9804 | 4,628.413 | 1,056 | 0.114 | 9,238 | 0.0000 | 0.0000 | NA |
| ENSG00000164761 | TNFRSF11B | 4982 | 14,214.761 | 1,067 | 0.128 | 8,343 | 0.0000 | 0.0000 | NA |
| ENSG00000109321 | AREG | 374 | 51,998 | 1,072 | 0.349 | 3,075 | 0.0021 | 0.0285 | NA |
| ENSG00000131979 | GCH1 | 2643 | 300,118 | 1,102 | 0.242 | 4,548 | 0.0000 | 0.0002 | NA |
| ENSG00000081277 | PKP1 | 5317 | 64,478 | 1,111 | 0.366 | 3,035 | 0.0024 | 0.0315 | NA |
| ENSG00000163347 | CLDN1 | 9076 | 1,263.074 | 1,114 | 0.176 | 6,326 | 0.0000 | 0.0000 | NA |
| ENSG00000120217 | CD274 | 29126 | 471,881 | 1,124 | 0.217 | 5,181 | 0.0000 | 0.0000 | NA |
| ENSG00000162654 | GBP4 | 115361 | 984,610 | 1,143 | 0.217 | 5,258 | 0.0000 | 0.0000 | NA |
| ENSG00000168214 | RBPJ | 3516 | 4,398.667 | 1,147 | 0.123 | 9,330 | 0.0000 | 0.0000 | NA |
| ENSG00000153310 | CYRIB | 51571 | 830,829 | 1,151 | 0.134 | 8,578 | 0.0000 | 0.0000 | NA |
| ENSG00000186106 | ANKRD46 | 157567 | 250,697 | 1,156 | 0.160 | 7,245 | 0.0000 | 0.0000 | NA |
| ENSG00000082074 | FYB1 | 2533 | 86,353 | 1,176 | 0.269 | 4,372 | 0.0000 | 0.0004 | NA |
| ENSG00000203786 | KPRP | 448834 | 120,741 | 1,179 | 0.292 | 4,042 | 0.0001 | 0.0014 | NA |
| ENSG00000112576 | CCND3 | 896 | 3,364.886 | 1,185 | 0.139 | 8,528 | 0.0000 | 0.0000 | NA |
| ENSG00000141682 | PMAIP1 | 5366 | 2,098.688 | 1,200 | 0.162 | 7,415 | 0.0000 | 0.0000 | NA |
| ENSG00000121858 | TNFSF10 | 8743 | 535,955 | 1,318 | 0.474 | 2,780 | 0.0054 | 0.0592 | NA |
| ENSG00000128567 | PODXL | 5420 | 810,399 | 1,364 | 0.407 | 3,350 | 0.0008 | 0.0133 | NA |
| ENSG00000169762 | TAPT1 | 202018 | 413,116 | 1,367 | 0.154 | 8,892 | 0.0000 | 0.0000 | NA |
| ENSG00000149948 | HMGA2 | 8091 | 2,813.038 | 1,415 | 0.186 | 7,622 | 0.0000 | 0.0000 | NA |
| ENSG00000067048 | DDX3Y | 8653 | 2,176.594 | 1,453 | 0.121 | 11,979 | 0.0000 | 0.0000 | NA |
| ENSG00000163207 | IVL | 3713 | 111,493 | 1,476 | 0.356 | 4,141 | 0.0000 | 0.0010 | NA |
| ENSG00000101188 | NTSR1 | 4923 | 82,885 | 1,542 | 0.326 | 4,729 | 0.0000 | 0.0001 | NA |
| ENSG00000235750 | KIAA0040 | 9674 | 46,713 | 1,550 | 0.374 | 4,143 | 0.0000 | 0.0010 | NA |
| ENSG00000120337 | TNFSF18 | 8995 | 49,633 | 1,589 | 0.421 | 3,775 | 0.0002 | 0.0035 | NA |
| ENSG00000007968 | E2F2 | 1870 | 196,831 | 1,623 | 0.197 | 8,253 | 0.0000 | 0.0000 | NA |
| ENSG00000105976 | MET | 4233 | 6,142.139 | 2,089 | 0.122 | 17,057 | 0.0000 | 0.0000 | NA |
| ENSG00000236824 | BCYRN1 | 618 | 151,040 | 2,289 | 0.678 | 3,378 | 0.0007 | 0.0122 | NA |

| Supplementary Table 7. DEGs of miR-223-3P with target score | | | | | | | | | |
| --- | --- | --- | --- | --- | --- | --- | --- | --- | --- |
|  | symbol | entrez | baseMean | log2Fold Change | lfcSE | stat | pvalue | padj | Target sore |
| ENSG00000170540 | ARL6IP1 | 23204 | 2,594.97 | -1.938 | 0.168 | -11.56 | 6.60E-31 | 1.50E-27 | NA |
| ENSG00000101363 | MANBAL | 63905 | 1,537.49 | -1.87 | 0.117 | -16.006 | 1.20E-57 | 5.10E-54 | 60 |
| ENSG00000143799 | PARP1 | 142 | 3,798.33 | -1.64 | 0.1 | -16.337 | 5.40E-60 | 7.10E-56 | 89 |
| ENSG00000166347 | CYB5A | 1528 | 1,438.02 | -1.591 | 0.103 | -15.505 | 3.20E-54 | 1.10E-50 | 71 |
| ENSG00000166106 | ADAMTS15 | 170689 | 72.481 | -1.492 | 0.418 | -3.571 | 0.00036 | 0.0095 | 58 |
| ENSG00000143061 | IGSF3 | 3321 | 55.186 | -1.481 | 0.382 | -3.874 | 0.00011 | 0.0036 | NA |
| ENSG00000152076 | CCDC74B | 91409 | 52.742 | -1.346 | 0.325 | -4.146 | 3.4E-05 | 0.0014 | 65 |
| ENSG00000104946 | TBC1D17 | 79735 | 1,471.18 | -1.299 | 0.16 | -8.133 | 4.20E-16 | 1.70E-13 | 85 |
| ENSG00000150054 | MPP7 | 143098 | 42.113 | -1.293 | 0.371 | -3.489 | 0.000500 | 0.012100 | NA |
| ENSG00000168917 | SLC35G2 | 80723 | 798.83 | -1.275 | 0.141 | -9.06 | 1.30E-19 | 7.80E-17 | 93 |
| ENSG00000092068 | SLC7A8 | 23428 | 1,389.14 | -1.259 | 0.14 | -8.997 | 2.30E-19 | 1.30E-16 | 91 |
| ENSG00000230615 |  |  | 46.617 | -1.252 | 0.364 | -3.44 | 0.000600 | 0.014100 | NA |
| ENSG00000136908 | DPM2 | 8818 | 1,289.60 | -1.246 | 0.152 | -8.22 | 2.00E-16 | 9.30E-14 | NA |
| ENSG00000080493 | SLC4A4 | 8671 | 881 | -1.242 | 0.133 | -9.317 | 1.20E-20 | 9.30E-18 | 97 |
| ENSG00000187634 | SAMD11 | 148398 | 101.585 | -1.236 | 0.421 | -2.934 | 0.0033 | 0.051 | NA |
| ENSG00000163040 | CCDC74A | 90557 | 184.539 | -1.235 | 0.199 | -6.22 | 5.00E-10 | 7.80E-08 | 65 |
| ENSG00000066117 | SMARCD1 | 6602 | 2,116.00 | -1.211 | 0.117 | -10.341 | 4.60E-25 | 5.50E-22 | 75 |
| ENSG00000169715 | MT1E | 4493 | 2,667.92 | -1.202 | 0.181 | -6.632 | 3.30E-11 | 6.50E-09 | NA |
| ENSG00000075702 | WDR62 | 284403 | 1,152.40 | -1.201 | 0.132 | -9.1 | 9.00E-20 | 5.70E-17 | NA |
| ENSG00000110031 | LPXN | 9404 | 4,092.44 | -1.194 | 0.106 | -11.238 | 2.60E-29 | 4.40E-26 | 59 |
| ENSG00000094916 | CBX5 | 23468 | 2,809.31 | -1.18 | 0.127 | -9.266 | 1.90E-20 | 1.30E-17 | 95 |
| ENSG00000164117 | FBXO8 | 26269 | 329.871 | -1.169 | 0.141 | -8.269 | 1.30E-16 | 6.40E-14 | 94 |
| ENSG00000126947 | ARMCX1 | 51309 | 1,984.70 | -1.163 | 0.121 | -9.589 | 8.90E-22 | 7.40E-19 | 94 |
| ENSG00000180694 | TMEM64 | 169200 | 474.162 | -1.115 | 0.143 | -7.808 | 5.80E-15 | 2.10E-12 | 54 |
| ENSG00000272899 | ATP6V1FNB | 1E+08 | 41.385 | -1.08 | 0.345 | -3.131 | 0.0017 | 0.032 | NA |
| ENSG00000174871 | CNIH2 | 254263 | 38.897 | -1.08 | 0.354 | -3.054 | 0.002300 | 0.038700 | NA |
| ENSG00000168078 | PBK | 55872 | 998.957 | -1.064 | 0.155 | -6.845 | 0.000000 | 0.000000 | NA |
| ENSG00000072952 | IRAG1 | 10335 | 185.199 | -1.062 | 0.277 | -3.84 | 0.000100 | 0.004100 | NA |
| ENSG00000143153 | ATP1B1 | 481 | 423.699 | -1.058 | 0.194 | -5.449 | 0.000000 | 0.000000 | 74 |
| ENSG00000117906 | RCN2 | 5955 | 1,985.38 | -1.028 | 0.166 | -6.173 | 6.70E-10 | 1.00E-07 | 80 |
| ENSG00000168309 | FAM107A | 11170 | 1,033.02 | -1.02 | 0.338 | -3.013 | 0.002600 | 0.042300 | NA |
| ENSG00000163431 | LMOD1 | 25802 | 254.224 | -1.016 | 0.196 | -5.189 | 0.000000 | 0.000000 | NA |
| ENSG00000110911 | SLC11A2 | 4891 | 2,759.58 | -1.007 | 0.104 | -9.644 | 0.000000 | 0.000000 | 59 |
| ENSG00000203706 |  |  | 41.2 | -1.007 | 0.359 | -2.805 | 0.005000 | 0.065400 | NA |
| ENSG00000132182 | NUP210 | 23225 | 47.679 | -1.004 | 0.335 | -2.993 | 0.002800 | 0.044700 | 86 |
| ENSG00000138606 | SHF | 90525 | 76.211 | -1.002 | 0.265 | -3.774 | 0.000200 | 0.005000 | NA |
| ENSG00000118898 | PPL | 5493 | 306.248 | -0.993 | 0.201 | -4.936 | 0.000000 | 0.000100 | NA |
| ENSG00000113356 | POLR3G | 10622 | 207.68 | -0.989 | 0.18 | -5.491 | 0.000000 | 0.000000 | 67 |
| ENSG00000104635 | SLC39A14 | 23516 | 6,652.46 | -0.976 | 0.091 | -10.68 | 0.000000 | 0.000000 | 56 |
| ENSG00000089057 | SLC23A2 | 9962 | 1,568.72 | -0.976 | 0.114 | -8.545 | 0.000000 | 0.000000 | 75 |
| ENSG00000207561 | MIR635 | 693220 | 38.343 | -0.975 | 0.322 | -3.03 | 0.002400 | 0.040800 | NA |
| ENSG00000101166 | PRELID3B | 51012 | 1,478.77 | -0.971 | 0.126 | -7.691 | 0.000000 | 0.000000 | NA |
| ENSG00000161847 | RAVER1 | 125950 | 1,074.30 | -0.964 | 0.153 | -6.287 | 0.000000 | 0.000000 | NA |
| ENSG00000137942 | FNBP1L | 54874 | 118.797 | -0.964 | 0.238 | -4.048 | 0.000100 | 0.002000 | NA |
| ENSG00000157368 | IL34 | 146433 | 107.851 | -0.963 | 0.281 | -3.429 | 0.000600 | 0.014400 | NA |
| ENSG00000138821 | SLC39A8 | 64116 | 1,241.79 | -0.96 | 0.138 | -6.942 | 0.000000 | 0.000000 | 76 |
| ENSG00000161920 | MED11 | 400569 | 177.19 | -0.953 | 0.188 | -5.069 | 0.000000 | 0.000000 | NA |
| ENSG00000167767 | KRT80 | 144501 | 183.35 | -0.953 | 0.193 | -4.945 | 0.000000 | 0.000100 | NA |
| ENSG00000112208 | BAG2 | 9532 | 1,872.52 | -0.947 | 0.093 | -10.153 | 0.000000 | 0.000000 | NA |
| ENSG00000198876 | DCAF12 | 25853 | 1,087.71 | -0.945 | 0.115 | -8.189 | 0.000000 | 0.000000 | 76 |
| ENSG00000143850 | PLEKHA6 | 22874 | 147.752 | -0.939 | 0.264 | -3.558 | 0.000400 | 0.009900 | NA |
| ENSG00000143878 | RHOB | 388 | 2,334.78 | -0.936 | 0.156 | -5.988 | 0.000000 | 0.000000 | 93 |
| ENSG00000090339 | ICAM1 | 3383 | 793.446 | -0.927 | 0.156 | -5.961 | 0.000000 | 0.000000 | 75 |
| ENSG00000065717 | TLE2 | 7089 | 48.409 | -0.925 | 0.342 | -2.704 | 0.006800 | 0.080100 | NA |
| ENSG00000259319 |  |  | 37.242 | -0.922 | 0.324 | -2.844 | 0.004500 | 0.060800 | NA |
| ENSG00000137992 | DBT | 1629 | 552.311 | -0.921 | 0.142 | -6.475 | 0.000000 | 0.000000 | 81 |
| ENSG00000109670 | FBXW7 | 55294 | 524.884 | -0.92 | 0.125 | -7.38 | 0.000000 | 0.000000 | 100 |
| ENSG00000249669 |  |  | 55.197 | -0.915 | 0.333 | -2.748 | 0.006000 | 0.074100 | NA |
| ENSG00000099953 | MMP11 | 4320 | 46.258 | -0.912 | 0.348 | -2.62 | 0.008800 | 0.095400 | NA |
| ENSG00000006327 | TNFRSF12A | 51330 | 8,514.55 | -0.9 | 0.153 | -5.891 | 0.000000 | 0.000000 | NA |
| ENSG00000101412 | E2F1 | 1869 | 871.607 | -0.899 | 0.165 | -5.453 | 0.000000 | 0.000000 | NA |
| ENSG00000184524 | CEND1 | 51286 | 99.461 | -0.89 | 0.27 | -3.299 | 0.001000 | 0.020900 | NA |
| ENSG00000102879 | CORO1A | 11151 | 41.275 | -0.887 | 0.338 | -2.621 | 0.008800 | 0.095400 | NA |
| ENSG00000066654 | THUMPD1 | 55623 | 1,007.98 | -0.885 | 0.119 | -7.462 | 0.000000 | 0.000000 | NA |
| ENSG00000177076 | ACER2 | 340485 | 74.448 | -0.884 | 0.253 | -3.497 | 0.000500 | 0.011800 | NA |
| ENSG00000123689 | G0S2 | 50486 | 231.045 | -0.882 | 0.287 | -3.069 | 0.002200 | 0.037400 | 68 |
| ENSG00000064652 | SNX24 | 28966 | 560.035 | -0.881 | 0.147 | -5.985 | 0.000000 | 0.000000 | NA |
| ENSG00000130270 | ATP8B3 | 148229 | 209.215 | -0.879 | 0.186 | -4.728 | 0.000000 | 0.000100 | NA |
| ENSG00000170412 | GPRC5C | 55890 | 59.791 | -0.877 | 0.316 | -2.775 | 0.005500 | 0.069800 | NA |
| ENSG00000108840 | HDAC5 | 10014 | 1,258.98 | -0.874 | 0.195 | -4.485 | 0.000000 | 0.000400 | NA |
| ENSG00000074219 | TEAD2 | 8463 | 575.637 | -0.873 | 0.171 | -5.1 | 0.000000 | 0.000000 | NA |
| ENSG00000261061 |  |  | 166.694 | -0.869 | 0.176 | -4.95 | 0.000000 | 0.000100 | NA |
| ENSG00000129195 | PIMREG | 54478 | 1,078.35 | -0.861 | 0.128 | -6.702 | 0.000000 | 0.000000 | NA |
| ENSG00000048392 | RRM2B | 50484 | 1,905.73 | -0.853 | 0.15 | -5.674 | 0.000000 | 0.000000 | NA |
| ENSG00000185112 | FAM43A | 131583 | 1,646.78 | -0.851 | 0.167 | -5.088 | 0.000000 | 0.000000 | NA |
| ENSG00000164850 | GPER1 | 2852 | 237.687 | -0.85 | 0.192 | -4.42 | 0.000000 | 0.000500 | NA |
| ENSG00000064692 | SNCAIP | 9627 | 272.643 | -0.847 | 0.194 | -4.377 | 0.000000 | 0.000600 | NA |
| ENSG00000119403 | PHF19 | 26147 | 1,909.88 | -0.842 | 0.095 | -8.831 | 0.000000 | 0.000000 | NA |
| ENSG00000171388 | APLN | 8862 | 84.242 | -0.836 | 0.264 | -3.165 | 0.001600 | 0.029300 | NA |
| ENSG00000204386 | NEU1 | 4758 | 6,683.64 | -0.833 | 0.106 | -7.853 | 0.000000 | 0.000000 | NA |
| ENSG00000213347 | MXD3 | 83463 | 370.311 | -0.832 | 0.195 | -4.26 | 0.000000 | 0.000900 | NA |
| ENSG00000183111 | ARHGEF37 | 389337 | 94.072 | -0.825 | 0.271 | -3.043 | 0.002300 | 0.039700 | NA |
| ENSG00000170345 | FOS | 2353 | 95.743 | -0.824 | 0.231 | -3.566 | 0.000400 | 0.009700 | NA |
| ENSG00000004777 | ARHGAP33 | 115703 | 321.41 | -0.823 | 0.155 | -5.303 | 0.000000 | 0.000000 | NA |
| ENSG00000253669 |  |  | 67.488 | -0.821 | 0.266 | -3.091 | 0.002000 | 0.035400 | NA |
| ENSG00000149596 | JPH2 | 57158 | 355.007 | -0.818 | 0.188 | -4.357 | 0.000000 | 0.000600 | NA |
| ENSG00000125354 | SEPTIN6 | 23157 | 558.214 | -0.815 | 0.137 | -5.931 | 0.000000 | 0.000000 | NA |
| ENSG00000233901 | LINC01503 | 1.01E+08 | 60.903 | -0.814 | 0.271 | -3 | 0.002700 | 0.043900 | NA |
| ENSG00000171603 | CLSTN1 | 22883 | 8,659.55 | -0.811 | 0.108 | -7.509 | 0.000000 | 0.000000 | 56 |
| ENSG00000204682 | MIR1915HG | 399726 | 105.037 | -0.811 | 0.233 | -3.486 | 0.000500 | 0.012200 | NA |
| ENSG00000162191 | UBXN1 | 51035 | 1,668.91 | -0.81 | 0.154 | -5.257 | 0.000000 | 0.000000 | 74 |
| ENSG00000104936 | DMPK | 1760 | 2,429.52 | -0.809 | 0.154 | -5.264 | 0.000000 | 0.000000 | NA |
| ENSG00000165240 | ATP7A | 538 | 670.871 | -0.808 | 0.112 | -7.206 | 0.000000 | 0.000000 | 97 |
| ENSG00000174059 | CD34 | 947 | 82.837 | -0.808 | 0.278 | -2.91 | 0.003600 | 0.053600 | NA |
| ENSG00000185090 | MANEAL | 149175 | 94.144 | -0.806 | 0.234 | -3.451 | 0.000600 | 0.013600 | NA |
| ENSG00000185697 | MYBL1 | 4603 | 1,339.13 | -0.802 | 0.157 | -5.098 | 0.000000 | 0.000000 | 85 |
| ENSG00000132932 | ATP8A2 | 51761 | 96.631 | -0.793 | 0.302 | -2.626 | 0.008600 | 0.094100 | NA |
| ENSG00000242294 | STAG3L5P | 1.02E+08 | 62.718 | -0.791 | 0.296 | -2.67 | 0.007600 | 0.085900 | NA |
| ENSG00000120693 | SMAD9 | 4093 | 178.209 | -0.79 | 0.181 | -4.362 | 0.000000 | 0.000600 | NA |
| ENSG00000138311 | ZNF365 | 22891 | 54.629 | -0.786 | 0.303 | -2.599 | 0.009400 | 0.099700 | 77 |
| ENSG00000163900 | TMEM41A | 90407 | 692.384 | -0.784 | 0.103 | -7.644 | 0.000000 | 0.000000 | 51 |
| ENSG00000102554 | KLF5 | 688 | 204.234 | -0.784 | 0.199 | -3.93 | 0.000100 | 0.003000 | NA |
| ENSG00000165915 | SLC39A13 | 91252 | 6,375.93 | -0.782 | 0.14 | -5.597 | 0.000000 | 0.000000 | NA |
| ENSG00000133026 | MYH10 | 4628 | 1,870.18 | -0.779 | 0.106 | -7.383 | 0.000000 | 0.000000 | 70 |
| ENSG00000117632 | STMN1 | 3925 | 5,270.67 | -0.778 | 0.126 | -6.174 | 0.000000 | 0.000000 | 53 |
| ENSG00000272092 |  |  | 58.779 | -0.778 | 0.272 | -2.863 | 0.004200 | 0.058800 | NA |
| ENSG00000188026 | RILPL1 | 353116 | 553.965 | -0.775 | 0.159 | -4.875 | 0.000000 | 0.000100 | NA |
| ENSG00000124613 | ZNF391 | 346157 | 112.096 | -0.773 | 0.227 | -3.4 | 0.000700 | 0.015600 | 78 |
| ENSG00000081913 | PHLPP1 | 23239 | 475.658 | -0.772 | 0.128 | -6.023 | 0.000000 | 0.000000 | 78 |
| ENSG00000096070 | BRPF3 | 27154 | 1,878.99 | -0.767 | 0.095 | -8.077 | 0.000000 | 0.000000 | 59 |
| ENSG00000124593 |  |  | 63.172 | -0.767 | 0.273 | -2.81 | 0.005000 | 0.064900 | NA |
| ENSG00000096433 | ITPR3 | 3710 | 12,299.32 | -0.764 | 0.114 | -6.682 | 0.000000 | 0.000000 | 76 |
| ENSG00000143570 | SLC39A1 | 27173 | 5,336.73 | -0.76 | 0.115 | -6.635 | 0.000000 | 0.000000 | 78 |
| ENSG00000137193 | PIM1 | 5292 | 241.721 | -0.759 | 0.218 | -3.487 | 0.000500 | 0.012100 | NA |
| ENSG00000164929 | BAALC | 79870 | 174.784 | -0.757 | 0.209 | -3.624 | 0.000300 | 0.008000 | NA |
| ENSG00000144476 | ACKR3 | 57007 | 238.475 | -0.754 | 0.202 | -3.737 | 0.000200 | 0.005600 | NA |
| ENSG00000121039 | RDH10 | 157506 | 3,960.39 | -0.752 | 0.161 | -4.687 | 0.000000 | 0.000200 | NA |
| ENSG00000105855 | ITGB8 | 3696 | 187.881 | -0.752 | 0.266 | -2.83 | 0.004600 | 0.062400 | NA |
| ENSG00000169439 | SDC2 | 6383 | 1,360.07 | -0.75 | 0.134 | -5.598 | 0.000000 | 0.000000 | 89 |
| ENSG00000110900 | TSPAN11 | 441631 | 343.323 | -0.75 | 0.189 | -3.958 | 0.000100 | 0.002700 | NA |
| ENSG00000158246 | TENT5B | 115572 | 79.322 | -0.748 | 0.276 | -2.709 | 0.006700 | 0.079500 | NA |
| ENSG00000116675 | DNAJC6 | 9829 | 1,478.40 | -0.747 | 0.105 | -7.087 | 0.000000 | 0.000000 | NA |
| ENSG00000101447 | FAM83D | 81610 | 1,148.10 | -0.743 | 0.166 | -4.49 | 0.000000 | 0.000400 | NA |
| ENSG00000235863 | B3GALT4 | 8705 | 92.242 | -0.739 | 0.254 | -2.912 | 0.003600 | 0.053300 | NA |
| ENSG00000178878 | APOLD1 | 81575 | 246.624 | -0.737 | 0.189 | -3.901 | 0.000100 | 0.003300 | NA |
| ENSG00000204084 | INPP5B | 3633 | 500.13 | -0.727 | 0.115 | -6.308 | 0.000000 | 0.000000 | 95 |
| ENSG00000167191 | GPRC5B | 51704 | 472.503 | -0.717 | 0.143 | -5.005 | 0.000000 | 0.000000 | NA |
| ENSG00000145911 | N4BP3 | 23138 | 149.842 | -0.717 | 0.259 | -2.772 | 0.005600 | 0.070200 | NA |
| ENSG00000147324 | MFHAS1 | 9258 | 780.597 | -0.715 | 0.109 | -6.552 | 0.000000 | 0.000000 | NA |
| ENSG00000220205 | VAMP2 | 6844 | 969.75 | -0.714 | 0.105 | -6.799 | 0.000000 | 0.000000 | NA |
| ENSG00000198732 | SMOC1 | 64093 | 280.358 | -0.713 | 0.254 | -2.812 | 0.004900 | 0.064800 | NA |
| ENSG00000151117 | TMEM86A | 144110 | 88.04 | -0.707 | 0.24 | -2.947 | 0.003200 | 0.049900 | NA |
| ENSG00000105655 | ISYNA1 | 51477 | 191.313 | -0.706 | 0.224 | -3.16 | 0.001600 | 0.029700 | NA |
| ENSG00000168874 | ATOH8 | 84913 | 1,044.90 | -0.698 | 0.163 | -4.283 | 0.000000 | 0.000800 | NA |
| ENSG00000136856 | SLC2A8 | 29988 | 558.519 | -0.697 | 0.133 | -5.23 | 0.000000 | 0.000000 | NA |
| ENSG00000164400 | CSF2 | 1437 | 335.832 | -0.696 | 0.254 | -2.736 | 0.006200 | 0.075500 | 51 |
| ENSG00000186522 | SEPTIN10 | 151011 | 2,028.13 | -0.695 | 0.129 | -5.402 | 0.000000 | 0.000000 | NA |
| ENSG00000197380 | DACT3 | 147906 | 243.528 | -0.694 | 0.212 | -3.272 | 0.001100 | 0.022300 | NA |
| ENSG00000198954 | KIFBP | 26128 | 1,571.20 | -0.688 | 0.107 | -6.457 | 0.000000 | 0.000000 | NA |
| ENSG00000107551 | RASSF4 | 83937 | 335.237 | -0.688 | 0.15 | -4.587 | 0.000000 | 0.000300 | NA |
| ENSG00000121281 | ADCY7 | 113 | 522.021 | -0.686 | 0.191 | -3.59 | 0.000300 | 0.009000 | 92 |
| ENSG00000010626 | LRRC23 | 10233 | 169.277 | -0.686 | 0.176 | -3.905 | 0.000100 | 0.003300 | NA |
| ENSG00000166575 | TMEM135 | 65084 | 396.648 | -0.685 | 0.143 | -4.793 | 0.000000 | 0.000100 | NA |
| ENSG00000133106 | EPSTI1 | 94240 | 2,414.91 | -0.685 | 0.173 | -3.953 | 0.000100 | 0.002800 | NA |
| ENSG00000161558 | TMEM143 | 55260 | 259.358 | -0.68 | 0.149 | -4.552 | 0.000000 | 0.000300 | 91 |
| ENSG00000117318 | ID3 | 3399 | 2,611.49 | -0.678 | 0.176 | -3.854 | 0.000100 | 0.003900 | NA |
| ENSG00000176909 | MAMSTR | 284358 | 107.297 | -0.677 | 0.237 | -2.858 | 0.004300 | 0.059100 | NA |
| ENSG00000154040 | CABYR | 26256 | 121.018 | -0.675 | 0.226 | -2.986 | 0.002800 | 0.045400 | NA |
| ENSG00000164604 | GPR85 | 54329 | 139.558 | -0.671 | 0.201 | -3.335 | 0.000900 | 0.018800 | NA |
| ENSG00000022267 | FHL1 | 2273 | 2,649.19 | -0.67 | 0.138 | -4.861 | 0.000000 | 0.000100 | 71 |
| ENSG00000008513 | ST3GAL1 | 6482 | 3,484.06 | -0.669 | 0.12 | -5.585 | 0.000000 | 0.000000 | NA |
| ENSG00000111305 | GSG1 | 83445 | 171.137 | -0.669 | 0.196 | -3.409 | 0.000700 | 0.015200 | NA |
| ENSG00000172380 | GNG12 | 55970 | 7,666.93 | -0.665 | 0.115 | -5.796 | 0.000000 | 0.000000 | NA |
| ENSG00000132000 | PODNL1 | 79883 | 130.827 | -0.663 | 0.209 | -3.174 | 0.001500 | 0.028600 | NA |
| ENSG00000174600 | CMKLR1 | 1240 | 376.955 | -0.659 | 0.187 | -3.535 | 0.000400 | 0.010600 | NA |
| ENSG00000131381 | RBSN | 64145 | 975.096 | -0.658 | 0.101 | -6.505 | 0.000000 | 0.000000 | 89 |
| ENSG00000099889 | ARVCF | 421 | 441.453 | -0.658 | 0.184 | -3.573 | 0.000400 | 0.009400 | NA |
| ENSG00000103034 | NDRG4 | 65009 | 170.261 | -0.658 | 0.23 | -2.863 | 0.004200 | 0.058800 | NA |
| ENSG00000281490 |  |  | 222.635 | -0.657 | 0.186 | -3.532 | 0.000400 | 0.010700 | NA |
| ENSG00000141510 | TP53 | 7157 | 1,626.21 | -0.656 | 0.126 | -5.221 | 0.000000 | 0.000000 | 71 |
| ENSG00000198807 | PAX9 | 5083 | 3,436.30 | -0.655 | 0.105 | -6.211 | 0.000000 | 0.000000 | 74 |
| ENSG00000072415 | MPP5 | 64398 | 1,628.84 | -0.652 | 0.131 | -4.972 | 0.000000 | 0.000100 | 75 |
| ENSG00000007237 | GAS7 | 8522 | 168.257 | -0.652 | 0.194 | -3.36 | 0.000800 | 0.017600 | NA |
| ENSG00000100968 | NFATC4 | 4776 | 4,619.82 | -0.651 | 0.144 | -4.515 | 0.000000 | 0.000300 | NA |
| ENSG00000144749 | LRIG1 | 26018 | 1,200.46 | -0.65 | 0.137 | -4.762 | 0.000000 | 0.000100 | NA |
| ENSG00000212747 | RTL8B | 441518 | 1,207.24 | -0.648 | 0.126 | -5.154 | 0.000000 | 0.000000 | NA |
| ENSG00000146950 | SHROOM2 | 357 | 87.528 | -0.646 | 0.248 | -2.606 | 0.009200 | 0.098500 | NA |
| ENSG00000073060 | SCARB1 | 949 | 1,120.19 | -0.645 | 0.125 | -5.144 | 0.000000 | 0.000000 | 56 |
| ENSG00000123836 | PFKFB2 | 5208 | 343.924 | -0.645 | 0.185 | -3.492 | 0.000500 | 0.012000 | NA |
| ENSG00000158402 | CDC25C | 995 | 238.766 | -0.645 | 0.22 | -2.937 | 0.003300 | 0.050900 | NA |
| ENSG00000166451 | CENPN | 55839 | 731.037 | -0.644 | 0.14 | -4.603 | 0.000000 | 0.000300 | 59 |
| ENSG00000085998 | POMGNT1 | 55624 | 4,961.69 | -0.643 | 0.106 | -6.077 | 0.000000 | 0.000000 | NA |
| ENSG00000204130 | RUFY2 | 55680 | 545.478 | -0.643 | 0.15 | -4.302 | 0.000000 | 0.000800 | NA |
| ENSG00000184363 | PKP3 | 11187 | 97.888 | -0.643 | 0.225 | -2.862 | 0.004200 | 0.058900 | NA |
| ENSG00000116299 | ELAPOR1 | 57535 | 232.463 | -0.642 | 0.191 | -3.359 | 0.000800 | 0.017600 | NA |
| ENSG00000186063 | AIDA | 64853 | 2,161.80 | -0.641 | 0.122 | -5.236 | 0.000000 | 0.000000 | NA |
| ENSG00000079308 | TNS1 | 7145 | 3,326.09 | -0.636 | 0.131 | -4.865 | 0.000000 | 0.000100 | NA |
| ENSG00000134508 | CABLES1 | 91768 | 1,220.01 | -0.636 | 0.129 | -4.92 | 0.000000 | 0.000100 | NA |
| ENSG00000161682 | FAM171A2 | 284069 | 129.926 | -0.633 | 0.232 | -2.723 | 0.006500 | 0.077300 | NA |
| ENSG00000112759 | SLC29A1 | 2030 | 1,059.83 | -0.631 | 0.13 | -4.849 | 0.000000 | 0.000100 | 81 |
| ENSG00000136274 | NACAD | 23148 | 299.69 | -0.629 | 0.165 | -3.807 | 0.000100 | 0.004500 | NA |
| ENSG00000151239 | TWF1 | 5756 | 2,748.73 | -0.628 | 0.161 | -3.898 | 0.000100 | 0.003300 | 90 |
| ENSG00000143013 | LMO4 | 8543 | 756.653 | -0.628 | 0.119 | -5.288 | 0.000000 | 0.000000 | NA |
| ENSG00000128872 | TMOD2 | 29767 | 606.689 | -0.628 | 0.137 | -4.596 | 0.000000 | 0.000300 | NA |
| ENSG00000227063 |  |  | 194.352 | -0.628 | 0.229 | -2.745 | 0.006100 | 0.074300 | NA |
| ENSG00000163468 | CCT3 | 7203 | 8,766.58 | -0.626 | 0.082 | -7.602 | 0.000000 | 0.000000 | NA |
| ENSG00000105514 | RAB3D | 9545 | 271.397 | -0.625 | 0.141 | -4.425 | 0.000000 | 0.000500 | NA |
| ENSG00000079435 | LIPE | 3991 | 125.38 | -0.625 | 0.206 | -3.032 | 0.002400 | 0.040700 | NA |
| ENSG00000112972 | HMGCS1 | 3157 | 2,114.04 | -0.622 | 0.146 | -4.267 | 0.000000 | 0.000900 | 91 |
| ENSG00000173801 | JUP | 3728 | 310.465 | -0.621 | 0.162 | -3.836 | 0.000100 | 0.004100 | NA |
| ENSG00000187800 | PEAR1 | 375033 | 1,406.81 | -0.621 | 0.181 | -3.426 | 0.000600 | 0.014500 | NA |
| ENSG00000129465 | RIPK3 | 11035 | 354.833 | -0.621 | 0.195 | -3.175 | 0.001500 | 0.028600 | NA |
| ENSG00000125740 | FOSB | 2354 | 265.094 | -0.619 | 0.195 | -3.173 | 0.001500 | 0.028600 | NA |
| ENSG00000164985 | PSIP1 | 11168 | 1,340.73 | -0.617 | 0.103 | -5.964 | 0.000000 | 0.000000 | 76 |
| ENSG00000198483 | ANKRD35 | 148741 | 126.872 | -0.616 | 0.23 | -2.68 | 0.007400 | 0.084000 | NA |
| ENSG00000130653 | PNPLA7 | 375775 | 144.55 | -0.612 | 0.217 | -2.82 | 0.004800 | 0.063900 | NA |
| ENSG00000118985 | ELL2 | 22936 | 5,248.94 | -0.609 | 0.127 | -4.813 | 0.000000 | 0.000100 | NA |
| ENSG00000173705 | SUSD5 | 26032 | 924.898 | -0.607 | 0.129 | -4.721 | 0.000000 | 0.000200 | NA |
| ENSG00000179094 | PER1 | 5187 | 278.561 | -0.607 | 0.153 | -3.977 | 0.000100 | 0.002600 | NA |
| ENSG00000175938 | ORAI3 | 93129 | 391.925 | -0.607 | 0.168 | -3.603 | 0.000300 | 0.008600 | NA |
| ENSG00000102096 | PIM2 | 11040 | 359.393 | -0.602 | 0.149 | -4.05 | 0.000100 | 0.002000 | NA |
| ENSG00000119946 | CNNM1 | 26507 | 382.953 | -0.602 | 0.162 | -3.721 | 0.000200 | 0.005800 | NA |
| ENSG00000008517 | IL32 | 9235 | 263.151 | -0.599 | 0.178 | -3.369 | 0.000800 | 0.017000 | NA |
| ENSG00000102225 | CDK16 | 5127 | 4,375.24 | -0.596 | 0.111 | -5.363 | 0.000000 | 0.000000 | NA |
| ENSG00000204580 | DDR1 | 780 | 1,117.94 | -0.596 | 0.183 | -3.266 | 0.001100 | 0.022600 | NA |
| ENSG00000100564 | PIGH | 5283 | 212.286 | -0.595 | 0.152 | -3.913 | 0.000100 | 0.003200 | NA |
| ENSG00000183856 | IQGAP3 | 128239 | 1,926.71 | -0.594 | 0.155 | -3.835 | 0.000100 | 0.004100 | NA |
| ENSG00000164294 | GPX8 | 493869 | 5,232.12 | -0.593 | 0.112 | -5.305 | 0.000000 | 0.000000 | 75 |
| ENSG00000139977 | NAA30 | 122830 | 775.087 | -0.592 | 0.133 | -4.447 | 0.000000 | 0.000400 | 65 |
| ENSG00000004700 | RECQL | 5965 | 1,652.08 | -0.591 | 0.14 | -4.223 | 0.000000 | 0.001000 | NA |
| ENSG00000198816 | ZNF358 | 140467 | 507.548 | -0.591 | 0.175 | -3.379 | 0.000700 | 0.016600 | NA |
| ENSG00000214357 | NEURL1B | 54492 | 347.397 | -0.591 | 0.182 | -3.258 | 0.001100 | 0.023200 | NA |
| ENSG00000089685 | BIRC5 | 332 | 2,606.11 | -0.589 | 0.132 | -4.45 | 0.000000 | 0.000400 | NA |
| ENSG00000108639 | SYNGR2 | 9144 | 3,138.02 | -0.589 | 0.145 | -4.07 | 0.000000 | 0.001800 | NA |
| ENSG00000105341 | DMAC2 | 55101 | 1,396.50 | -0.588 | 0.116 | -5.052 | 0.000000 | 0.000000 | NA |
| ENSG00000143416 | SELENBP1 | 8991 | 569.341 | -0.588 | 0.133 | -4.422 | 0.000000 | 0.000500 | NA |
| ENSG00000052802 | MSMO1 | 6307 | 1,509.10 | -0.587 | 0.141 | -4.179 | 0.000000 | 0.001200 | NA |
| ENSG00000158859 | ADAMTS4 | 9507 | 443.29 | -0.586 | 0.133 | -4.407 | 0.000000 | 0.000500 | NA |
| ENSG00000101974 | ATP11C | 286410 | 860.319 | -0.585 | 0.12 | -4.881 | 0.000000 | 0.000100 | NA |
| ENSG00000166592 | RRAD | 6236 | 1,005.42 | -0.584 | 0.179 | -3.257 | 0.001100 | 0.023200 | NA |
| ENSG00000221990 | EXOC3-AS1 | 116349 | 151.924 | -0.584 | 0.186 | -3.145 | 0.001700 | 0.030900 | NA |
| ENSG00000100162 | CENPM | 79019 | 439.335 | -0.583 | 0.153 | -3.817 | 0.000100 | 0.004400 | NA |
| ENSG00000161835 | TAMALIN | 160622 | 143.725 | -0.583 | 0.217 | -2.686 | 0.007200 | 0.082800 | NA |
| ENSG00000105519 | CAPS | 828 | 172.374 | -0.583 | 0.225 | -2.596 | 0.009400 | 0.100000 | NA |
| ENSG00000185904 | LINC00839 | 84856 | 634.198 | -0.578 | 0.111 | -5.227 | 0.000000 | 0.000000 | NA |
| ENSG00000169895 | SYAP1 | 94056 | 1,783.53 | -0.577 | 0.11 | -5.259 | 0.000000 | 0.000000 | 63 |
| ENSG00000147408 | CSGALNACT1 | 55790 | 651.983 | -0.576 | 0.128 | -4.504 | 0.000000 | 0.000400 | NA |
| ENSG00000152784 | PRDM8 | 56978 | 972.095 | -0.576 | 0.136 | -4.223 | 0.000000 | 0.001000 | NA |
| ENSG00000164976 | MYORG | 57462 | 281.054 | -0.575 | 0.18 | -3.202 | 0.001400 | 0.026600 | NA |
| ENSG00000166128 | RAB8B | 51762 | 1,103.55 | -0.574 | 0.149 | -3.844 | 0.000100 | 0.004000 | 82 |
| ENSG00000005884 | ITGA3 | 3675 | 13,447.00 | -0.572 | 0.158 | -3.629 | 0.000300 | 0.007900 | NA |
| ENSG00000173402 | DAG1 | 1605 | 10,590.45 | -0.57 | 0.099 | -5.782 | 0.000000 | 0.000000 | 62 |
| ENSG00000127837 | AAMP | 14 | 3,784.68 | -0.564 | 0.115 | -4.895 | 0.000000 | 0.000100 | NA |
| ENSG00000085982 | USP40 | 55230 | 1,555.07 | -0.563 | 0.087 | -6.469 | 0.000000 | 0.000000 | 88 |
| ENSG00000173641 | HSPB7 | 27129 | 2,196.48 | -0.561 | 0.133 | -4.215 | 0.000000 | 0.001100 | NA |
| ENSG00000132879 | FBXO44 | 93611 | 641.687 | -0.561 | 0.184 | -3.044 | 0.002300 | 0.039700 | NA |
| ENSG00000105227 | PRX | 57716 | 156.553 | -0.561 | 0.203 | -2.771 | 0.005600 | 0.070400 | NA |
| ENSG00000143924 | EML4 | 27436 | 1,408.37 | -0.56 | 0.117 | -4.792 | 0.000000 | 0.000100 | NA |
| ENSG00000167992 | VWCE | 220001 | 506.345 | -0.56 | 0.125 | -4.467 | 0.000000 | 0.000400 | NA |
| ENSG00000129472 | RAB2B | 84932 | 501.828 | -0.56 | 0.132 | -4.253 | 0.000000 | 0.000900 | NA |
| ENSG00000136044 | APPL2 | 55198 | 2,202.01 | -0.558 | 0.158 | -3.536 | 0.000400 | 0.010600 | NA |
| ENSG00000112984 | KIF20A | 10112 | 2,563.83 | -0.556 | 0.126 | -4.416 | 0.000000 | 0.000500 | NA |
| ENSG00000123080 | CDKN2C | 1031 | 1,461.90 | -0.556 | 0.125 | -4.427 | 0.000000 | 0.000500 | NA |
| ENSG00000205978 | NYNRIN | 57523 | 1,372.28 | -0.556 | 0.144 | -3.861 | 0.000100 | 0.003800 | NA |
| ENSG00000103257 | SLC7A5 | 8140 | 17,341.85 | -0.554 | 0.131 | -4.217 | 0.000000 | 0.001100 | NA |
| ENSG00000072163 | LIMS2 | 55679 | 545.597 | -0.554 | 0.183 | -3.029 | 0.002500 | 0.040900 | NA |
| ENSG00000150457 | LATS2 | 26524 | 2,124.91 | -0.553 | 0.121 | -4.549 | 0.000000 | 0.000300 | NA |
| ENSG00000167600 | CYP2S1 | 29785 | 212.123 | -0.551 | 0.188 | -2.924 | 0.003500 | 0.052200 | NA |
| ENSG00000163701 | IL17RE | 132014 | 246.587 | -0.551 | 0.196 | -2.804 | 0.005000 | 0.065600 | NA |
| ENSG00000104976 | SNAPC2 | 6618 | 1,342.44 | -0.55 | 0.147 | -3.733 | 0.000200 | 0.005600 | NA |
| ENSG00000108602 | ALDH3A1 | 218 | 476.118 | -0.55 | 0.196 | -2.81 | 0.005000 | 0.064900 | NA |
| ENSG00000163602 | RYBP | 23429 | 1,241.24 | -0.548 | 0.13 | -4.222 | 0.000000 | 0.001000 | NA |
| ENSG00000164933 | SLC25A32 | 81034 | 806.976 | -0.547 | 0.145 | -3.765 | 0.000200 | 0.005100 | 51 |
| ENSG00000177595 | PIDD1 | 55367 | 897.794 | -0.547 | 0.16 | -3.409 | 0.000700 | 0.015200 | NA |
| ENSG00000221890 | NPTXR | 23467 | 529.155 | -0.546 | 0.137 | -3.998 | 0.000100 | 0.002400 | NA |
| ENSG00000161513 | FDXR | 2232 | 1,248.05 | -0.546 | 0.141 | -3.863 | 0.000100 | 0.003800 | NA |
| ENSG00000006576 | PHTF2 | 57157 | 952.922 | -0.546 | 0.166 | -3.288 | 0.001000 | 0.021400 | NA |
| ENSG00000004139 | SARM1 | 23098 | 158.283 | -0.546 | 0.2 | -2.731 | 0.006300 | 0.076400 | NA |
| ENSG00000165572 | KBTBD6 | 89890 | 355.047 | -0.545 | 0.138 | -3.959 | 0.000100 | 0.002700 | NA |
| ENSG00000099875 | MKNK2 | 2872 | 1,968.08 | -0.544 | 0.16 | -3.405 | 0.000700 | 0.015400 | NA |
| ENSG00000162624 | LHX8 | 431707 | 830.046 | -0.543 | 0.12 | -4.505 | 0.000000 | 0.000400 | NA |
| ENSG00000138162 | TACC2 | 10579 | 1,189.57 | -0.543 | 0.143 | -3.806 | 0.000100 | 0.004500 | NA |
| ENSG00000106628 | POLD2 | 5425 | 3,548.59 | -0.541 | 0.108 | -5.024 | 0.000000 | 0.000000 | NA |
| ENSG00000173786 | CNP | 1267 | 8,284.89 | -0.541 | 0.16 | -3.393 | 0.000700 | 0.015900 | NA |
| ENSG00000135362 | PRR5L | 79899 | 266.884 | -0.541 | 0.18 | -3.015 | 0.002600 | 0.042300 | NA |
| ENSG00000186575 | NF2 | 4771 | 3,841.02 | -0.538 | 0.124 | -4.354 | 0.000000 | 0.000600 | NA |
| ENSG00000069275 | NUCKS1 | 64710 | 6,786.35 | -0.537 | 0.131 | -4.087 | 0.000000 | 0.001700 | NA |
| ENSG00000271122 | LOC101930085 | 1.02E+08 | 265.505 | -0.537 | 0.149 | -3.616 | 0.000300 | 0.008300 | NA |
| ENSG00000088280 | ASAP3 | 55616 | 944.17 | -0.536 | 0.142 | -3.789 | 0.000200 | 0.004700 | NA |
| ENSG00000197093 | GAL3ST4 | 79690 | 422.182 | -0.536 | 0.194 | -2.762 | 0.005700 | 0.071800 | NA |
| ENSG00000167157 | PRRX2 | 51450 | 2,098.31 | -0.535 | 0.175 | -3.048 | 0.002300 | 0.039300 | NA |
| ENSG00000157800 | SLC37A3 | 84255 | 1,450.75 | -0.534 | 0.163 | -3.27 | 0.001100 | 0.022400 | 88 |
| ENSG00000143816 | WNT9A | 7483 | 1,621.01 | -0.533 | 0.185 | -2.873 | 0.004100 | 0.057700 | NA |
| ENSG00000137364 | TPMT | 7172 | 1,149.32 | -0.532 | 0.123 | -4.315 | 0.000000 | 0.000700 | NA |
| ENSG00000111276 | CDKN1B | 1027 | 828.499 | -0.531 | 0.125 | -4.248 | 0.000000 | 0.000900 | NA |
| ENSG00000106785 | TRIM14 | 9830 | 3,815.48 | -0.53 | 0.118 | -4.471 | 0.000000 | 0.000400 | 55 |
| ENSG00000122873 | CISD1 | 55847 | 636.508 | -0.53 | 0.12 | -4.436 | 0.000000 | 0.000500 | NA |
| ENSG00000126351 | THRA | 7067 | 1,400.58 | -0.53 | 0.125 | -4.25 | 0.000000 | 0.000900 | NA |
| ENSG00000135451 | TROAP | 10024 | 825.737 | -0.53 | 0.132 | -4.022 | 0.000100 | 0.002200 | NA |
| ENSG00000171812 | COL8A2 | 1296 | 361.951 | -0.53 | 0.158 | -3.359 | 0.000800 | 0.017600 | NA |
| ENSG00000165424 | ZCCHC24 | 219654 | 2,750.68 | -0.529 | 0.117 | -4.517 | 0.000000 | 0.000300 | NA |
| ENSG00000205746 | PKD1P4 | 353512 | 258.345 | -0.529 | 0.178 | -2.968 | 0.003000 | 0.047500 | NA |
| ENSG00000065243 | PKN2 | 5586 | 1,295.43 | -0.528 | 0.116 | -4.568 | 0.000000 | 0.000300 | 67 |
| ENSG00000160447 | PKN3 | 29941 | 500.23 | -0.528 | 0.129 | -4.08 | 0.000000 | 0.001800 | NA |
| ENSG00000077713 | SLC25A43 | 203427 | 1,143.23 | -0.527 | 0.127 | -4.131 | 0.000000 | 0.001500 | NA |
| ENSG00000107796 | ACTA2 | 59 | 2,543.94 | -0.527 | 0.138 | -3.815 | 0.000100 | 0.004400 | NA |
| ENSG00000164627 | KIF6 | 221458 | 301.839 | -0.527 | 0.194 | -2.716 | 0.006600 | 0.078300 | NA |
| ENSG00000173599 | PC | 5091 | 607.239 | -0.526 | 0.127 | -4.13 | 0.000000 | 0.001500 | NA |
| ENSG00000128283 | CDC42EP1 | 11135 | 3,538.74 | -0.526 | 0.147 | -3.588 | 0.000300 | 0.009000 | NA |
| ENSG00000149218 | ENDOD1 | 23052 | 2,858.23 | -0.526 | 0.156 | -3.379 | 0.000700 | 0.016600 | NA |
| ENSG00000064687 | ABCA7 | 10347 | 192.443 | -0.526 | 0.168 | -3.137 | 0.001700 | 0.031500 | NA |
| ENSG00000272886 | DCP1A | 55802 | 1,125.82 | -0.525 | 0.13 | -4.04 | 0.000100 | 0.002000 | NA |
| ENSG00000113721 | PDGFRB | 5159 | 4,102.12 | -0.525 | 0.152 | -3.462 | 0.000500 | 0.013100 | NA |
| ENSG00000023445 | BIRC3 | 330 | 2,438.80 | -0.525 | 0.154 | -3.416 | 0.000600 | 0.014900 | NA |
| ENSG00000138018 | SELENOI | 85465 | 1,136.68 | -0.524 | 0.11 | -4.772 | 0.000000 | 0.000100 | 64 |
| ENSG00000175832 | ETV4 | 2118 | 315.108 | -0.523 | 0.169 | -3.089 | 0.002000 | 0.035500 | NA |
| ENSG00000138134 | STAMBPL1 | 57559 | 1,719.77 | -0.522 | 0.109 | -4.781 | 0.000000 | 0.000100 | NA |
| ENSG00000163170 | BOLA3 | 388962 | 1,077.45 | -0.522 | 0.115 | -4.549 | 0.000000 | 0.000300 | NA |
| ENSG00000171823 | FBXL14 | 144699 | 250.013 | -0.522 | 0.145 | -3.601 | 0.000300 | 0.008600 | NA |
| ENSG00000134222 | PSRC1 | 84722 | 872.699 | -0.522 | 0.164 | -3.181 | 0.001500 | 0.028000 | NA |
| ENSG00000185596 |  |  | 206.256 | -0.521 | 0.182 | -2.858 | 0.004300 | 0.059200 | NA |
| ENSG00000116521 | SCAMP3 | 10067 | 5,712.34 | -0.518 | 0.105 | -4.931 | 0.000000 | 0.000100 | NA |
| ENSG00000108797 | CNTNAP1 | 8506 | 2,742.40 | -0.518 | 0.109 | -4.744 | 0.000000 | 0.000100 | NA |
| ENSG00000108106 | UBE2S | 27338 | 5,408.56 | -0.517 | 0.141 | -3.668 | 0.000200 | 0.007000 | NA |
| ENSG00000083290 | ULK2 | 9706 | 516.186 | -0.516 | 0.123 | -4.209 | 0.000000 | 0.001100 | 65 |
| ENSG00000112033 | PPARD | 5467 | 1,994.25 | -0.515 | 0.102 | -5.023 | 0.000000 | 0.000000 | NA |
| ENSG00000280798 | LINC00294 | 283267 | 202.506 | -0.515 | 0.166 | -3.109 | 0.001900 | 0.034000 | NA |
| ENSG00000150938 | CRIM1 | 51232 | 17,257.88 | -0.514 | 0.117 | -4.393 | 0.000000 | 0.000500 | 97 |
| ENSG00000049540 | ELN | 2006 | 36,597.96 | -0.514 | 0.144 | -3.568 | 0.000400 | 0.009600 | NA |
| ENSG00000167900 | TK1 | 7083 | 2,994.61 | -0.514 | 0.15 | -3.439 | 0.000600 | 0.014100 | NA |
| ENSG00000111665 | CDCA3 | 83461 | 1,238.49 | -0.512 | 0.153 | -3.339 | 0.000800 | 0.018600 | NA |
| ENSG00000119688 | ABCD4 | 5826 | 745.335 | -0.511 | 0.114 | -4.49 | 0.000000 | 0.000400 | 68 |
| ENSG00000164877 | MICALL2 | 79778 | 1,195.29 | -0.51 | 0.166 | -3.082 | 0.002100 | 0.036200 | NA |
| ENSG00000187244 | BCAM | 4059 | 332.404 | -0.51 | 0.196 | -2.602 | 0.009300 | 0.099100 | NA |
| ENSG00000135424 | ITGA7 | 3679 | 1,317.63 | -0.509 | 0.13 | -3.922 | 0.000100 | 0.003100 | NA |
| ENSG00000150593 | PDCD4 | 27250 | 1,344.41 | -0.509 | 0.144 | -3.542 | 0.000400 | 0.010400 | NA |
| ENSG00000112149 | CD83 | 9308 | 190.234 | -0.508 | 0.177 | -2.876 | 0.004000 | 0.057400 | 68 |
| ENSG00000196730 | DAPK1 | 1612 | 596.35 | -0.507 | 0.133 | -3.803 | 0.000100 | 0.004500 | NA |
| ENSG00000149451 | ADAM33 | 80332 | 3,643.03 | -0.507 | 0.138 | -3.68 | 0.000200 | 0.006700 | NA |
| ENSG00000184702 | SEPTIN5 | 5413 | 336.301 | -0.507 | 0.165 | -3.068 | 0.002200 | 0.037400 | NA |
| ENSG00000100307 | CBX7 | 23492 | 396.375 | -0.506 | 0.148 | -3.422 | 0.000600 | 0.014700 | NA |
| ENSG00000167702 | KIFC2 | 90990 | 824.141 | -0.506 | 0.171 | -2.95 | 0.003200 | 0.049500 | NA |
| ENSG00000012171 | SEMA3B | 7869 | 895.871 | -0.506 | 0.174 | -2.906 | 0.003700 | 0.054100 | NA |
| ENSG00000137393 | RNF144B | 255488 | 476.975 | -0.505 | 0.167 | -3.03 | 0.002400 | 0.040900 | NA |
| ENSG00000175305 | CCNE2 | 9134 | 175.036 | -0.505 | 0.192 | -2.626 | 0.008600 | 0.094200 | NA |
| ENSG00000170456 | DENND5B | 160518 | 362.429 | -0.503 | 0.155 | -3.238 | 0.001200 | 0.024400 | 84 |
| ENSG00000168385 | SEPTIN2 | 4735 | 14,555.60 | -0.503 | 0.102 | -4.933 | 0.000000 | 0.000100 | NA |
| ENSG00000136161 | RCBTB2 | 1102 | 284.831 | -0.503 | 0.156 | -3.221 | 0.001300 | 0.025500 | NA |
| ENSG00000175711 | B3GNTL1 | 146712 | 175.106 | -0.503 | 0.161 | -3.122 | 0.001800 | 0.032800 | NA |
| ENSG00000121753 | ADGRB2 | 576 | 486.492 | -0.502 | 0.138 | -3.642 | 0.000300 | 0.007600 | NA |
| ENSG00000086544 | ITPKC | 80271 | 637.46 | -0.502 | 0.15 | -3.34 | 0.000800 | 0.018600 | NA |
| ENSG00000077454 | LRCH4 | 4034 | 472.113 | -0.5 | 0.182 | -2.745 | 0.006100 | 0.074300 | NA |
| ENSG00000139625 | MAP3K12 | 7786 | 1,463.14 | -0.498 | 0.14 | -3.555 | 0.000400 | 0.010000 | NA |
| ENSG00000204219 | TCEA3 | 6920 | 249.204 | -0.497 | 0.143 | -3.483 | 0.000500 | 0.012300 | NA |
| ENSG00000164087 | POC1A | 25886 | 841.692 | -0.496 | 0.113 | -4.393 | 0.000000 | 0.000500 | NA |
| ENSG00000166444 | DENND2B | 6764 | 1,195.34 | -0.496 | 0.116 | -4.259 | 0.000000 | 0.000900 | NA |
| ENSG00000102287 | GABRE | 2564 | 262.691 | -0.495 | 0.165 | -2.996 | 0.002700 | 0.044400 | NA |
| ENSG00000091136 | LAMB1 | 3912 | 9,035.96 | -0.494 | 0.093 | -5.295 | 0.000000 | 0.000000 | 90 |
| ENSG00000131943 | C19orf12 | 83636 | 1,139.93 | -0.494 | 0.119 | -4.14 | 0.000000 | 0.001400 | NA |
| ENSG00000135480 | KRT7 | 3855 | 249.655 | -0.494 | 0.18 | -2.747 | 0.006000 | 0.074100 | NA |
| ENSG00000198380 | GFPT1 | 2673 | 5,381.66 | -0.493 | 0.098 | -5.048 | 0.000000 | 0.000000 | NA |
| ENSG00000110492 | MDK | 4192 | 2,444.38 | -0.492 | 0.187 | -2.635 | 0.008400 | 0.092700 | 74 |
| ENSG00000164054 | SHISA5 | 51246 | 10,824.47 | -0.492 | 0.098 | -5.018 | 0.000000 | 0.000000 | NA |
| ENSG00000075945 | KIFAP3 | 22920 | 819.649 | -0.492 | 0.107 | -4.593 | 0.000000 | 0.000300 | NA |
| ENSG00000110328 | GALNT18 | 374378 | 423.614 | -0.491 | 0.131 | -3.755 | 0.000200 | 0.005200 | 96 |
| ENSG00000167323 | STIM1 | 6786 | 2,979.31 | -0.49 | 0.102 | -4.817 | 0.000000 | 0.000100 | 75 |
| ENSG00000139880 | CDH24 | 64403 | 319.987 | -0.488 | 0.17 | -2.871 | 0.004100 | 0.057900 | NA |
| ENSG00000185361 | TNFAIP8L1 | 126282 | 231.714 | -0.486 | 0.173 | -2.815 | 0.004900 | 0.064400 | NA |
| ENSG00000127824 | TUBA4A | 7277 | 1,819.09 | -0.485 | 0.115 | -4.211 | 0.000000 | 0.001100 | NA |
| ENSG00000168306 | ACOX2 | 8309 | 423.794 | -0.485 | 0.131 | -3.711 | 0.000200 | 0.006000 | NA |
| ENSG00000117650 | NEK2 | 4751 | 603.34 | -0.485 | 0.183 | -2.654 | 0.008000 | 0.088700 | NA |
| ENSG00000064393 | HIPK2 | 28996 | 6,830.50 | -0.484 | 0.105 | -4.621 | 0.000000 | 0.000200 | 63 |
| ENSG00000011332 | DPF1 | 8193 | 147.318 | -0.484 | 0.179 | -2.701 | 0.006900 | 0.080600 | NA |
| ENSG00000127564 | PKMYT1 | 9088 | 1,572.52 | -0.483 | 0.177 | -2.723 | 0.006500 | 0.077300 | NA |
| ENSG00000068137 | PLEKHH3 | 79990 | 731.791 | -0.482 | 0.134 | -3.599 | 0.000300 | 0.008700 | NA |
| ENSG00000175634 | RPS6KB2 | 6199 | 1,362.70 | -0.481 | 0.138 | -3.492 | 0.000500 | 0.012000 | NA |
| ENSG00000179151 | EDC3 | 80153 | 1,058.84 | -0.48 | 0.1 | -4.797 | 0.000000 | 0.000100 | NA |
| ENSG00000176974 | SHMT1 | 6470 | 776.901 | -0.48 | 0.115 | -4.18 | 0.000000 | 0.001200 | NA |
| ENSG00000169744 | LDB2 | 9079 | 341.276 | -0.48 | 0.181 | -2.657 | 0.007900 | 0.088300 | NA |
| ENSG00000229320 | KRT8P12 | 90133 | 249.548 | -0.48 | 0.182 | -2.639 | 0.008300 | 0.091900 | NA |
| ENSG00000099860 | GADD45B | 4616 | 864.948 | -0.478 | 0.163 | -2.941 | 0.003300 | 0.050600 | NA |
| ENSG00000204381 | LAYN | 143903 | 3,803.52 | -0.477 | 0.081 | -5.894 | 0.000000 | 0.000000 | 63 |
| ENSG00000033867 | SLC4A7 | 9497 | 2,615.59 | -0.477 | 0.136 | -3.516 | 0.000400 | 0.011200 | NA |
| ENSG00000170153 | RNF150 | 57484 | 495.946 | -0.477 | 0.155 | -3.083 | 0.002100 | 0.036200 | NA |
| ENSG00000139946 | PELI2 | 57161 | 249.687 | -0.477 | 0.172 | -2.774 | 0.005500 | 0.069900 | NA |
| ENSG00000174839 | DENND6A | 201627 | 317.734 | -0.476 | 0.151 | -3.15 | 0.001600 | 0.030400 | 52 |
| ENSG00000135617 | PRADC1 | 84279 | 683.806 | -0.476 | 0.111 | -4.303 | 0.000000 | 0.000800 | NA |
| ENSG00000184371 | CSF1 | 1435 | 11,682.21 | -0.475 | 0.159 | -2.985 | 0.002800 | 0.045500 | NA |
| ENSG00000163346 | PBXIP1 | 57326 | 2,088.75 | -0.474 | 0.145 | -3.277 | 0.001000 | 0.022000 | NA |
| ENSG00000213722 | DDAH2 | 23564 | 2,146.85 | -0.473 | 0.133 | -3.549 | 0.000400 | 0.010100 | NA |
| ENSG00000185909 | KLHDC8B | 200942 | 834.587 | -0.473 | 0.135 | -3.519 | 0.000400 | 0.011100 | NA |
| ENSG00000169169 | CPT1C | 126129 | 613.205 | -0.473 | 0.16 | -2.96 | 0.003100 | 0.048300 | NA |
| ENSG00000181826 | RELL1 | 768211 | 183.078 | -0.473 | 0.177 | -2.671 | 0.007600 | 0.085800 | NA |
| ENSG00000131370 | SH3BP5 | 9467 | 297.83 | -0.472 | 0.162 | -2.919 | 0.003500 | 0.052500 | NA |
| ENSG00000116016 | EPAS1 | 2034 | 29,169.52 | -0.47 | 0.09 | -5.234 | 0.000000 | 0.000000 | NA |
| ENSG00000092445 | TYRO3 | 7301 | 1,300.35 | -0.469 | 0.107 | -4.383 | 0.000000 | 0.000600 | NA |
| ENSG00000121644 | DESI2 | 51029 | 1,526.35 | -0.468 | 0.123 | -3.818 | 0.000100 | 0.004300 | 79 |
| ENSG00000181982 | CCDC149 | 91050 | 755.559 | -0.468 | 0.116 | -4.048 | 0.000100 | 0.002000 | NA |
| ENSG00000125730 | C3 | 718 | 7,829.09 | -0.467 | 0.121 | -3.855 | 0.000100 | 0.003900 | NA |
| ENSG00000135074 | ADAM19 | 8728 | 665.985 | -0.467 | 0.122 | -3.831 | 0.000100 | 0.004200 | NA |
| ENSG00000198796 | ALPK2 | 115701 | 594.924 | -0.466 | 0.118 | -3.96 | 0.000100 | 0.002700 | NA |
| ENSG00000153048 | CARHSP1 | 23589 | 738.451 | -0.466 | 0.122 | -3.836 | 0.000100 | 0.004100 | NA |
| ENSG00000160199 | PKNOX1 | 5316 | 485.664 | -0.465 | 0.126 | -3.705 | 0.000200 | 0.006200 | 79 |
| ENSG00000171307 | ZDHHC16 | 84287 | 2,279.02 | -0.465 | 0.092 | -5.041 | 0.000000 | 0.000000 | NA |
| ENSG00000161692 | DBF4B | 80174 | 714.675 | -0.465 | 0.117 | -3.979 | 0.000100 | 0.002500 | NA |
| ENSG00000135045 | C9orf40 | 55071 | 515.623 | -0.465 | 0.127 | -3.666 | 0.000200 | 0.007000 | NA |
| ENSG00000181218 | H2AW | 92815 | 356.608 | -0.465 | 0.179 | -2.604 | 0.009200 | 0.098800 | NA |
| ENSG00000214530 | STARD10 | 10809 | 322.661 | -0.463 | 0.153 | -3.025 | 0.002500 | 0.041200 | NA |
| ENSG00000186166 | CENATAC | 338657 | 241.794 | -0.463 | 0.158 | -2.921 | 0.003500 | 0.052400 | NA |
| ENSG00000132481 | TRIM47 | 91107 | 860.816 | -0.462 | 0.15 | -3.08 | 0.002100 | 0.036300 | NA |
| ENSG00000134057 | CCNB1 | 891 | 3,413.50 | -0.461 | 0.15 | -3.06 | 0.002200 | 0.038100 | NA |
| ENSG00000163517 | HDAC11 | 79885 | 632.571 | -0.461 | 0.159 | -2.896 | 0.003800 | 0.055200 | NA |
| ENSG00000164099 | PRSS12 | 8492 | 6,650.12 | -0.46 | 0.083 | -5.516 | 0.000000 | 0.000000 | 56 |
| ENSG00000099992 | TBC1D10A | 83874 | 485.481 | -0.46 | 0.158 | -2.906 | 0.003700 | 0.054100 | 57 |
| ENSG00000204291 | COL15A1 | 1306 | 675.114 | -0.46 | 0.15 | -3.056 | 0.002200 | 0.038500 | NA |
| ENSG00000164050 | PLXNB1 | 5364 | 2,039.69 | -0.459 | 0.147 | -3.127 | 0.001800 | 0.032300 | 67 |
| ENSG00000114030 | KPNA1 | 3836 | 1,924.36 | -0.459 | 0.108 | -4.256 | 0.000000 | 0.000900 | 86 |
| ENSG00000166851 | PLK1 | 5347 | 2,208.49 | -0.459 | 0.143 | -3.208 | 0.001300 | 0.026300 | NA |
| ENSG00000162496 | DHRS3 | 9249 | 1,098.59 | -0.458 | 0.143 | -3.211 | 0.001300 | 0.026100 | NA |
| ENSG00000172059 | KLF11 | 8462 | 276.093 | -0.456 | 0.14 | -3.248 | 0.001200 | 0.023700 | NA |
| ENSG00000220785 |  |  | 244.831 | -0.455 | 0.158 | -2.879 | 0.004000 | 0.057000 | NA |
| ENSG00000164611 | PTTG1 | 9232 | 1,911.36 | -0.454 | 0.127 | -3.59 | 0.000300 | 0.009000 | NA |
| ENSG00000120509 | PDZD11 | 51248 | 1,479.18 | -0.451 | 0.099 | -4.542 | 0.000000 | 0.000300 | NA |
| ENSG00000148481 | MINDY3 | 80013 | 1,110.06 | -0.45 | 0.112 | -4 | 0.000100 | 0.002400 | 72 |
| ENSG00000110237 | ARHGEF17 | 9828 | 2,808.50 | -0.45 | 0.12 | -3.746 | 0.000200 | 0.005400 | NA |
| ENSG00000126561 | STAT5A | 6776 | 697.502 | -0.45 | 0.135 | -3.328 | 0.000900 | 0.019100 | NA |
| ENSG00000029725 | RABEP1 | 9135 | 2,295.02 | -0.448 | 0.104 | -4.295 | 0.000000 | 0.000800 | NA |
| ENSG00000100908 | EMC9 | 51016 | 323.706 | -0.448 | 0.152 | -2.94 | 0.003300 | 0.050600 | NA |
| ENSG00000135679 | MDM2 | 4193 | 3,630.79 | -0.447 | 0.12 | -3.735 | 0.000200 | 0.005600 | 67 |
| ENSG00000223865 | HLA-DPB1 | 3115 | 173.977 | -0.447 | 0.168 | -2.654 | 0.007900 | 0.088700 | NA |
| ENSG00000170390 | DCLK2 | 166614 | 446.926 | -0.446 | 0.145 | -3.078 | 0.002100 | 0.036400 | NA |
| ENSG00000135736 | CCDC102A | 92922 | 291.352 | -0.446 | 0.162 | -2.753 | 0.005900 | 0.073200 | NA |
| ENSG00000100065 | CARD10 | 29775 | 703.818 | -0.445 | 0.121 | -3.683 | 0.000200 | 0.006700 | NA |
| ENSG00000111206 | FOXM1 | 2305 | 3,048.36 | -0.444 | 0.098 | -4.536 | 0.000000 | 0.000300 | NA |
| ENSG00000110906 | KCTD10 | 83892 | 5,403.33 | -0.442 | 0.109 | -4.06 | 0.000000 | 0.001900 | NA |
| ENSG00000143819 | EPHX1 | 2052 | 3,540.50 | -0.442 | 0.111 | -3.971 | 0.000100 | 0.002600 | NA |
| ENSG00000196739 | COL27A1 | 85301 | 1,047.11 | -0.441 | 0.121 | -3.64 | 0.000300 | 0.007600 | NA |
| ENSG00000068912 | ERLEC1 | 27248 | 3,075.51 | -0.44 | 0.105 | -4.168 | 0.000000 | 0.001300 | NA |
| ENSG00000172840 | PDP2 | 57546 | 635.358 | -0.44 | 0.118 | -3.721 | 0.000200 | 0.005800 | NA |
| ENSG00000166794 | PPIB | 5479 | 5,860.38 | -0.439 | 0.112 | -3.907 | 0.000100 | 0.003300 | NA |
| ENSG00000105290 | APLP1 | 333 | 577.497 | -0.439 | 0.126 | -3.478 | 0.000500 | 0.012500 | NA |
| ENSG00000100116 | GCAT | 23464 | 436.551 | -0.439 | 0.133 | -3.306 | 0.000900 | 0.020400 | NA |
| ENSG00000186918 | ZNF395 | 55893 | 888.168 | -0.438 | 0.106 | -4.12 | 0.000000 | 0.001500 | NA |
| ENSG00000146426 | TIAM2 | 26230 | 245.007 | -0.438 | 0.159 | -2.75 | 0.006000 | 0.073700 | NA |
| ENSG00000113645 | WWC1 | 23286 | 785.361 | -0.438 | 0.165 | -2.655 | 0.007900 | 0.088700 | NA |
| ENSG00000174238 | PITPNA | 5306 | 3,654.70 | -0.437 | 0.084 | -5.214 | 0.000000 | 0.000000 | 59 |
| ENSG00000100626 | GALNT16 | 57452 | 608.9 | -0.437 | 0.146 | -2.991 | 0.002800 | 0.044800 | NA |
| ENSG00000185813 | PCYT2 | 5833 | 3,221.21 | -0.436 | 0.127 | -3.441 | 0.000600 | 0.014100 | NA |
| ENSG00000181019 | NQO1 | 1728 | 19,035.39 | -0.435 | 0.111 | -3.922 | 0.000100 | 0.003100 | NA |
| ENSG00000011523 | CEP68 | 23177 | 465.809 | -0.435 | 0.117 | -3.706 | 0.000200 | 0.006100 | NA |
| ENSG00000162627 | SNX7 | 51375 | 1,502.88 | -0.435 | 0.123 | -3.526 | 0.000400 | 0.010800 | NA |
| ENSG00000110697 | PITPNM1 | 9600 | 1,471.97 | -0.435 | 0.14 | -3.103 | 0.001900 | 0.034500 | NA |
| ENSG00000175274 | TP53I11 | 9537 | 6,556.31 | -0.435 | 0.14 | -3.096 | 0.002000 | 0.035100 | NA |
| ENSG00000077721 | UBE2A | 7319 | 3,559.17 | -0.434 | 0.098 | -4.425 | 0.000000 | 0.000500 | 81 |
| ENSG00000141577 | CEP131 | 22994 | 459.843 | -0.434 | 0.148 | -2.932 | 0.003400 | 0.051400 | NA |
| ENSG00000124145 | SDC4 | 6385 | 10,014.92 | -0.433 | 0.091 | -4.741 | 0.000000 | 0.000100 | NA |
| ENSG00000113758 | DBN1 | 1627 | 4,894.07 | -0.433 | 0.136 | -3.182 | 0.001500 | 0.028000 | NA |
| ENSG00000171169 | NAIF1 | 203245 | 333.147 | -0.432 | 0.16 | -2.693 | 0.007100 | 0.081700 | 59 |
| ENSG00000070366 | SMG6 | 23293 | 1,159.95 | -0.432 | 0.104 | -4.135 | 0.000000 | 0.001400 | 60 |
| ENSG00000118454 | ANKRD13C | 81573 | 1,235.80 | -0.432 | 0.113 | -3.814 | 0.000100 | 0.004400 | NA |
| ENSG00000140451 | PIF1 | 80119 | 377.14 | -0.431 | 0.151 | -2.86 | 0.004200 | 0.059000 | NA |
| ENSG00000263528 | IKBKE | 9641 | 1,060.31 | -0.43 | 0.155 | -2.766 | 0.005700 | 0.071100 | NA |
| ENSG00000110171 | TRIM3 | 10612 | 929.729 | -0.429 | 0.106 | -4.032 | 0.000100 | 0.002100 | NA |
| ENSG00000168067 | MAP4K2 | 5871 | 588.462 | -0.429 | 0.134 | -3.201 | 0.001400 | 0.026600 | NA |
| ENSG00000075218 | GTSE1 | 51512 | 1,521.23 | -0.429 | 0.147 | -2.925 | 0.003400 | 0.052200 | NA |
| ENSG00000107738 | VSIR | 64115 | 2,537.21 | -0.429 | 0.15 | -2.86 | 0.004200 | 0.059000 | NA |
| ENSG00000006118 | TMEM132A | 54972 | 7,256.02 | -0.429 | 0.151 | -2.851 | 0.004400 | 0.059800 | NA |
| ENSG00000151849 | CENPJ | 55835 | 548.377 | -0.429 | 0.158 | -2.715 | 0.006600 | 0.078400 | NA |
| ENSG00000063322 | MED29 | 55588 | 2,048.36 | -0.428 | 0.113 | -3.784 | 0.000200 | 0.004800 | NA |
| ENSG00000129355 | CDKN2D | 1032 | 548.641 | -0.428 | 0.16 | -2.673 | 0.007500 | 0.085400 | NA |
| ENSG00000067955 | CBFB | 865 | 1,110.56 | -0.427 | 0.098 | -4.345 | 0.000000 | 0.000700 | 58 |
| ENSG00000204371 | EHMT2 | 10919 | 1,938.01 | -0.427 | 0.122 | -3.498 | 0.000500 | 0.011800 | NA |
| ENSG00000076382 | SPAG5 | 10615 | 1,659.36 | -0.427 | 0.139 | -3.082 | 0.002100 | 0.036200 | NA |
| ENSG00000154237 | LRRK1 | 79705 | 1,076.67 | -0.426 | 0.098 | -4.343 | 0.000000 | 0.000700 | NA |
| ENSG00000176170 | SPHK1 | 8877 | 3,857.75 | -0.426 | 0.14 | -3.032 | 0.002400 | 0.040700 | NA |
| ENSG00000211445 | GPX3 | 2878 | 4,154.28 | -0.425 | 0.092 | -4.592 | 0.000000 | 0.000300 | NA |
| ENSG00000235106 | BRD3OS | 266655 | 1,118.70 | -0.425 | 0.12 | -3.542 | 0.000400 | 0.010400 | NA |
| ENSG00000060656 | PTPRU | 10076 | 1,042.82 | -0.425 | 0.158 | -2.693 | 0.007100 | 0.081700 | NA |
| ENSG00000154945 | ANKRD40 | 91369 | 1,491.67 | -0.424 | 0.096 | -4.418 | 0.000000 | 0.000500 | 81 |
| ENSG00000168234 | TTC39C | 125488 | 1,013.34 | -0.424 | 0.133 | -3.197 | 0.001400 | 0.026900 | NA |
| ENSG00000145907 | G3BP1 | 10146 | 6,333.70 | -0.423 | 0.099 | -4.271 | 0.000000 | 0.000900 | 83 |
| ENSG00000175592 | FOSL1 | 8061 | 13,393.39 | -0.423 | 0.122 | -3.467 | 0.000500 | 0.013000 | NA |
| ENSG00000002822 | MAD1L1 | 8379 | 1,902.97 | -0.422 | 0.153 | -2.767 | 0.005700 | 0.071000 | NA |
| ENSG00000138443 | ABI2 | 10152 | 1,171.18 | -0.421 | 0.123 | -3.431 | 0.000600 | 0.014400 | 78 |
| ENSG00000047579 | DTNBP1 | 84062 | 572.592 | -0.421 | 0.118 | -3.562 | 0.000400 | 0.009800 | NA |
| ENSG00000136205 | TNS3 | 64759 | 7,463.20 | -0.421 | 0.123 | -3.434 | 0.000600 | 0.014200 | NA |
| ENSG00000163050 | COQ8A | 56997 | 437.475 | -0.421 | 0.14 | -2.996 | 0.002700 | 0.044400 | NA |
| ENSG00000215861 |  |  | 396.727 | -0.421 | 0.144 | -2.924 | 0.003500 | 0.052200 | NA |
| ENSG00000187257 | RSBN1L | 222194 | 342.407 | -0.42 | 0.147 | -2.855 | 0.004300 | 0.059500 | 59 |
| ENSG00000213015 | ZNF580 | 51157 | 852.973 | -0.42 | 0.15 | -2.806 | 0.005000 | 0.065400 | NA |
| ENSG00000114019 | AMOTL2 | 51421 | 5,672.49 | -0.419 | 0.149 | -2.813 | 0.004900 | 0.064700 | NA |
| ENSG00000065989 | PDE4A | 5141 | 576.402 | -0.419 | 0.155 | -2.706 | 0.006800 | 0.080100 | NA |
| ENSG00000119689 | DLST | 1743 | 6,430.84 | -0.418 | 0.095 | -4.397 | 0.000000 | 0.000500 | NA |
| ENSG00000157510 | AFAP1L1 | 134265 | 1,247.51 | -0.417 | 0.12 | -3.463 | 0.000500 | 0.013100 | NA |
| ENSG00000122515 | ZMIZ2 | 83637 | 4,622.97 | -0.417 | 0.128 | -3.263 | 0.001100 | 0.022800 | NA |
| ENSG00000090889 | KIF4A | 24137 | 1,395.33 | -0.417 | 0.132 | -3.171 | 0.001500 | 0.028700 | NA |
| ENSG00000063241 | ISOC2 | 79763 | 2,028.61 | -0.417 | 0.147 | -2.85 | 0.004400 | 0.060000 | NA |
| ENSG00000198612 | COPS8 | 10920 | 2,422.50 | -0.416 | 0.113 | -3.685 | 0.000200 | 0.006600 | NA |
| ENSG00000183023 | SLC8A1 | 6546 | 813.065 | -0.415 | 0.139 | -2.99 | 0.002800 | 0.044900 | 91 |
| ENSG00000092621 | PHGDH | 26227 | 1,326.99 | -0.415 | 0.126 | -3.284 | 0.001000 | 0.021700 | NA |
| ENSG00000151806 | GUF1 | 60558 | 864.326 | -0.415 | 0.135 | -3.075 | 0.002100 | 0.036800 | NA |
| ENSG00000171241 | SHCBP1 | 79801 | 2,251.56 | -0.415 | 0.141 | -2.943 | 0.003200 | 0.050300 | NA |
| ENSG00000135924 | DNAJB2 | 3300 | 2,556.54 | -0.414 | 0.115 | -3.604 | 0.000300 | 0.008600 | NA |
| ENSG00000131584 | ACAP3 | 116983 | 2,727.59 | -0.413 | 0.132 | -3.127 | 0.001800 | 0.032300 | NA |
| ENSG00000177666 | PNPLA2 | 57104 | 3,057.73 | -0.413 | 0.148 | -2.798 | 0.005100 | 0.066500 | NA |
| ENSG00000222009 | BTBD19 | 149478 | 354.698 | -0.413 | 0.157 | -2.629 | 0.008600 | 0.094000 | NA |
| ENSG00000153904 | DDAH1 | 23576 | 3,989.53 | -0.412 | 0.121 | -3.414 | 0.000600 | 0.015000 | NA |
| ENSG00000072135 | PTPN18 | 26469 | 1,255.31 | -0.412 | 0.132 | -3.127 | 0.001800 | 0.032300 | NA |
| ENSG00000213079 | SCAF8 | 22828 | 1,550.20 | -0.411 | 0.099 | -4.144 | 0.000000 | 0.001400 | 61 |
| ENSG00000136732 | GYPC | 2995 | 3,185.82 | -0.411 | 0.124 | -3.316 | 0.000900 | 0.019800 | NA |
| ENSG00000145861 | C1QTNF2 | 114898 | 504.771 | -0.411 | 0.132 | -3.106 | 0.001900 | 0.034300 | NA |
| ENSG00000166387 | PPFIBP2 | 8495 | 817.784 | -0.41 | 0.117 | -3.504 | 0.000500 | 0.011600 | NA |
| ENSG00000148411 | NACC2 | 138151 | 2,453.22 | -0.409 | 0.12 | -3.419 | 0.000600 | 0.014800 | NA |
| ENSG00000189337 | KAZN | 23254 | 1,060.33 | -0.409 | 0.127 | -3.228 | 0.001200 | 0.025000 | NA |
| ENSG00000170745 | KCNS3 | 3790 | 870.929 | -0.407 | 0.116 | -3.498 | 0.000500 | 0.011800 | 53 |
| ENSG00000140022 | STON2 | 85439 | 429.49 | -0.407 | 0.143 | -2.838 | 0.004500 | 0.061700 | NA |
| ENSG00000104856 | RELB | 5971 | 1,104.07 | -0.407 | 0.146 | -2.786 | 0.005300 | 0.068000 | NA |
| ENSG00000147027 | TMEM47 | 83604 | 4,022.42 | -0.406 | 0.125 | -3.236 | 0.001200 | 0.024600 | NA |
| ENSG00000137814 | HAUS2 | 55142 | 721.626 | -0.406 | 0.143 | -2.831 | 0.004600 | 0.062400 | NA |
| ENSG00000198892 | SHISA4 | 149345 | 1,227.54 | -0.406 | 0.146 | -2.783 | 0.005400 | 0.068400 | NA |
| ENSG00000205181 | LINC00654 | 149837 | 293.335 | -0.405 | 0.138 | -2.945 | 0.003200 | 0.050100 | NA |
| ENSG00000276023 | DUSP14 | 11072 | 2,009.32 | -0.404 | 0.087 | -4.641 | 0.000000 | 0.000200 | NA |
| ENSG00000162745 | OLFML2B | 25903 | 3,276.82 | -0.404 | 0.121 | -3.333 | 0.000900 | 0.018900 | NA |
| ENSG00000136045 | PWP1 | 11137 | 2,730.02 | -0.403 | 0.088 | -4.591 | 0.000000 | 0.000300 | 52 |
| ENSG00000145016 | RUBCN | 9711 | 1,604.68 | -0.403 | 0.148 | -2.729 | 0.006400 | 0.076600 | 72 |
| ENSG00000186185 | KIF18B | 146909 | 1,222.44 | -0.403 | 0.146 | -2.764 | 0.005700 | 0.071500 | NA |
| ENSG00000158711 | ELK4 | 2005 | 784.455 | -0.402 | 0.126 | -3.205 | 0.001400 | 0.026500 | 81 |
| ENSG00000137070 | IL11RA | 3590 | 519.483 | -0.402 | 0.139 | -2.898 | 0.003800 | 0.055200 | NA |
| ENSG00000176485 | PLAAT3 | 11145 | 730.527 | -0.401 | 0.126 | -3.186 | 0.001400 | 0.027800 | NA |
| ENSG00000128989 | ARPP19 | 10776 | 3,338.44 | -0.401 | 0.131 | -3.055 | 0.002200 | 0.038500 | NA |
| ENSG00000120896 | SORBS3 | 10174 | 4,243.98 | -0.401 | 0.132 | -3.031 | 0.002400 | 0.040700 | NA |
| ENSG00000100364 | KIAA0930 | 23313 | 5,936.77 | -0.4 | 0.102 | -3.902 | 0.000100 | 0.003300 | NA |
| ENSG00000123416 | TUBA1B | 10376 | 14,274.19 | -0.399 | 0.095 | -4.187 | 0.000000 | 0.001200 | 64 |
| ENSG00000177427 | MIEF2 | 125170 | 644.35 | -0.399 | 0.138 | -2.889 | 0.003900 | 0.056300 | NA |
| ENSG00000160446 | ZDHHC12 | 84885 | 1,692.54 | -0.399 | 0.14 | -2.854 | 0.004300 | 0.059600 | NA |
| ENSG00000142327 | RNPEPL1 | 57140 | 3,096.54 | -0.398 | 0.148 | -2.698 | 0.007000 | 0.081100 | NA |
| ENSG00000250251 | PKD1P6 | 353511 | 379.549 | -0.397 | 0.153 | -2.596 | 0.009400 | 0.100000 | NA |
| ENSG00000155368 | DBI | 1622 | 4,422.73 | -0.396 | 0.091 | -4.369 | 0.000000 | 0.000600 | NA |
| ENSG00000077943 | ITGA8 | 8516 | 1,985.26 | -0.396 | 0.15 | -2.647 | 0.008100 | 0.090200 | NA |
| ENSG00000038358 | EDC4 | 23644 | 1,848.24 | -0.394 | 0.106 | -3.735 | 0.000200 | 0.005600 | NA |
| ENSG00000148153 | INIP | 58493 | 920.427 | -0.394 | 0.138 | -2.851 | 0.004400 | 0.059800 | NA |
| ENSG00000102753 | KPNA3 | 3839 | 2,237.06 | -0.393 | 0.123 | -3.205 | 0.001400 | 0.026500 | 97 |
| ENSG00000230733 |  |  | 506.301 | -0.393 | 0.128 | -3.08 | 0.002100 | 0.036300 | NA |
| ENSG00000105968 | H2AZ2 | 94239 | 2,861.42 | -0.392 | 0.104 | -3.765 | 0.000200 | 0.005100 | NA |
| ENSG00000168734 | PKIG | 11142 | 2,039.47 | -0.392 | 0.122 | -3.222 | 0.001300 | 0.025500 | NA |
| ENSG00000136859 | ANGPTL2 | 23452 | 4,902.15 | -0.392 | 0.129 | -3.044 | 0.002300 | 0.039700 | NA |
| ENSG00000167468 | GPX4 | 2879 | 9,817.55 | -0.39 | 0.119 | -3.28 | 0.001000 | 0.021900 | NA |
| ENSG00000129474 | AJUBA | 84962 | 2,348.98 | -0.389 | 0.127 | -3.071 | 0.002100 | 0.037200 | NA |
| ENSG00000188186 | LAMTOR4 | 389541 | 2,016.43 | -0.389 | 0.131 | -2.962 | 0.003100 | 0.048100 | NA |
| ENSG00000206190 | ATP10A | 57194 | 2,109.89 | -0.389 | 0.138 | -2.814 | 0.004900 | 0.064500 | NA |
| ENSG00000139182 | CLSTN3 | 9746 | 720.527 | -0.389 | 0.143 | -2.724 | 0.006400 | 0.077300 | NA |
| ENSG00000145284 | SCD5 | 79966 | 419.915 | -0.388 | 0.127 | -3.067 | 0.002200 | 0.037500 | NA |
| ENSG00000012232 | EXTL3 | 2137 | 3,048.62 | -0.387 | 0.085 | -4.531 | 0.000000 | 0.000300 | NA |
| ENSG00000213672 | NCKIPSD | 51517 | 1,105.37 | -0.387 | 0.113 | -3.428 | 0.000600 | 0.014500 | NA |
| ENSG00000143390 | RFX5 | 5993 | 1,441.39 | -0.387 | 0.138 | -2.812 | 0.004900 | 0.064800 | NA |
| ENSG00000032444 | PNPLA6 | 10908 | 3,410.17 | -0.387 | 0.139 | -2.788 | 0.005300 | 0.067800 | NA |
| ENSG00000128274 | A4GALT | 53947 | 1,185.61 | -0.387 | 0.147 | -2.628 | 0.008600 | 0.094100 | NA |
| ENSG00000138760 | SCARB2 | 950 | 16,309.17 | -0.386 | 0.103 | -3.749 | 0.000200 | 0.005300 | NA |
| ENSG00000196182 | STK40 | 83931 | 3,825.95 | -0.386 | 0.131 | -2.951 | 0.003200 | 0.049500 | NA |
| ENSG00000161800 | RACGAP1 | 29127 | 3,207.55 | -0.386 | 0.138 | -2.789 | 0.005300 | 0.067700 | NA |
| ENSG00000198952 | SMG5 | 23381 | 5,315.14 | -0.385 | 0.107 | -3.581 | 0.000300 | 0.009200 | NA |
| ENSG00000119326 | CTNNAL1 | 8727 | 1,787.40 | -0.385 | 0.119 | -3.22 | 0.001300 | 0.025600 | NA |
| ENSG00000161011 | SQSTM1 | 8878 | 60,167.25 | -0.385 | 0.144 | -2.676 | 0.007500 | 0.084900 | NA |
| ENSG00000111077 | TNS2 | 23371 | 1,979.64 | -0.382 | 0.115 | -3.327 | 0.000900 | 0.019200 | NA |
| ENSG00000088387 | DOCK9 | 23348 | 720.921 | -0.382 | 0.116 | -3.295 | 0.001000 | 0.021100 | NA |
| ENSG00000165912 | PACSIN3 | 29763 | 504.229 | -0.382 | 0.145 | -2.627 | 0.008600 | 0.094100 | NA |
| ENSG00000119280 | C1orf198 | 84886 | 3,585.75 | -0.381 | 0.133 | -2.861 | 0.004200 | 0.059000 | NA |
| ENSG00000166260 | COX11 | 1353 | 683.626 | -0.38 | 0.131 | -2.897 | 0.003800 | 0.055200 | NA |
| ENSG00000166341 | DCHS1 | 8642 | 670.558 | -0.38 | 0.145 | -2.627 | 0.008600 | 0.094100 | NA |
| ENSG00000119333 | DYNC2I2 | 89891 | 2,125.26 | -0.379 | 0.13 | -2.921 | 0.003500 | 0.052400 | NA |
| ENSG00000107175 | CREB3 | 10488 | 4,516.58 | -0.378 | 0.117 | -3.225 | 0.001300 | 0.025300 | NA |
| ENSG00000113328 | CCNG1 | 900 | 2,359.48 | -0.378 | 0.13 | -2.921 | 0.003500 | 0.052400 | NA |
| ENSG00000204564 | C6orf136 | 221545 | 465.32 | -0.377 | 0.135 | -2.797 | 0.005200 | 0.066600 | 67 |
| ENSG00000145246 | ATP10D | 57205 | 1,023.47 | -0.377 | 0.118 | -3.208 | 0.001300 | 0.026300 | 69 |
| ENSG00000163961 | RNF168 | 165918 | 488.611 | -0.377 | 0.127 | -2.978 | 0.002900 | 0.046400 | 79 |
| ENSG00000090238 | YPEL3 | 83719 | 452.663 | -0.376 | 0.126 | -2.983 | 0.002900 | 0.045700 | NA |
| ENSG00000153560 | UBP1 | 7342 | 2,954.65 | -0.375 | 0.089 | -4.197 | 0.000000 | 0.001100 | NA |
| ENSG00000198909 | MAP3K3 | 4215 | 1,625.33 | -0.375 | 0.095 | -3.958 | 0.000100 | 0.002700 | NA |
| ENSG00000130707 | ASS1 | 445 | 4,058.99 | -0.375 | 0.134 | -2.787 | 0.005300 | 0.067900 | NA |
| ENSG00000135821 | GLUL | 2752 | 9,712.30 | -0.374 | 0.119 | -3.143 | 0.001700 | 0.030900 | NA |
| ENSG00000188486 | H2AX | 3014 | 4,334.97 | -0.374 | 0.139 | -2.695 | 0.007000 | 0.081400 | NA |
| ENSG00000100577 | GSTZ1 | 2954 | 501.775 | -0.373 | 0.129 | -2.891 | 0.003800 | 0.055800 | NA |
| ENSG00000162413 | KLHL21 | 9903 | 5,856.29 | -0.373 | 0.13 | -2.86 | 0.004200 | 0.059000 | NA |
| ENSG00000085788 | DDHD2 | 23259 | 1,192.16 | -0.371 | 0.113 | -3.283 | 0.001000 | 0.021700 | 69 |
| ENSG00000102390 | PBDC1 | 51260 | 798.434 | -0.371 | 0.107 | -3.459 | 0.000500 | 0.013200 | NA |
| ENSG00000221955 | SLC12A8 | 84561 | 1,438.54 | -0.371 | 0.131 | -2.837 | 0.004600 | 0.061700 | NA |
| ENSG00000133460 | SLC2A11 | 66035 | 293.979 | -0.37 | 0.137 | -2.692 | 0.007100 | 0.081800 | 73 |
| ENSG00000077684 | JADE1 | 79960 | 742.437 | -0.37 | 0.135 | -2.736 | 0.006200 | 0.075600 | NA |
| ENSG00000186111 | PIP5K1C | 23396 | 5,487.97 | -0.369 | 0.123 | -2.99 | 0.002800 | 0.044900 | NA |
| ENSG00000149179 | C11orf49 | 79096 | 628.477 | -0.369 | 0.136 | -2.716 | 0.006600 | 0.078300 | NA |
| ENSG00000171100 | MTM1 | 4534 | 456.453 | -0.368 | 0.13 | -2.819 | 0.004800 | 0.064100 | 73 |
| ENSG00000162542 | TMCO4 | 255104 | 1,134.54 | -0.368 | 0.134 | -2.748 | 0.006000 | 0.074100 | NA |
| ENSG00000116120 | FARSB | 10056 | 2,138.58 | -0.367 | 0.115 | -3.193 | 0.001400 | 0.027200 | NA |
| ENSG00000108448 | TRIM16L | 147166 | 660.826 | -0.367 | 0.118 | -3.111 | 0.001900 | 0.033900 | NA |
| ENSG00000117399 | CDC20 | 991 | 3,420.39 | -0.367 | 0.133 | -2.761 | 0.005800 | 0.071900 | NA |
| ENSG00000013810 | TACC3 | 10460 | 3,646.26 | -0.365 | 0.127 | -2.886 | 0.003900 | 0.056500 | NA |
| ENSG00000101400 | SNTA1 | 6640 | 1,248.22 | -0.365 | 0.129 | -2.815 | 0.004900 | 0.064400 | NA |
| ENSG00000109113 | RAB34 | 83871 | 5,320.73 | -0.364 | 0.102 | -3.558 | 0.000400 | 0.009900 | NA |
| ENSG00000149260 | CAPN5 | 726 | 1,899.69 | -0.364 | 0.125 | -2.923 | 0.003500 | 0.052400 | NA |
| ENSG00000138172 | CALHM2 | 51063 | 1,388.25 | -0.364 | 0.137 | -2.66 | 0.007800 | 0.087700 | NA |
| ENSG00000079805 | DNM2 | 1785 | 2,851.87 | -0.363 | 0.115 | -3.158 | 0.001600 | 0.029800 | NA |
| ENSG00000166741 | NNMT | 4837 | 1,705.17 | -0.361 | 0.128 | -2.828 | 0.004700 | 0.062700 | NA |
| ENSG00000139641 | ESYT1 | 23344 | 13,558.13 | -0.36 | 0.079 | -4.537 | 0.000000 | 0.000300 | NA |
| ENSG00000072062 | PRKACA | 5566 | 4,243.65 | -0.36 | 0.088 | -4.073 | 0.000000 | 0.001800 | NA |
| ENSG00000117298 | ECE1 | 1889 | 8,836.36 | -0.36 | 0.121 | -2.989 | 0.002800 | 0.045000 | NA |
| ENSG00000141562 | NARF | 26502 | 715.945 | -0.36 | 0.122 | -2.952 | 0.003200 | 0.049300 | NA |
| ENSG00000144791 | LIMD1 | 8994 | 1,221.74 | -0.359 | 0.119 | -3.015 | 0.002600 | 0.042300 | NA |
| ENSG00000104823 | ECH1 | 1891 | 555.331 | -0.358 | 0.126 | -2.833 | 0.004600 | 0.062200 | NA |
| ENSG00000085117 | CD82 | 3732 | 3,630.38 | -0.358 | 0.131 | -2.73 | 0.006300 | 0.076500 | NA |
| ENSG00000145860 | RNF145 | 153830 | 2,691.61 | -0.357 | 0.099 | -3.605 | 0.000300 | 0.008600 | 75 |
| ENSG00000158850 | B4GALT3 | 8703 | 1,106.40 | -0.356 | 0.099 | -3.594 | 0.000300 | 0.008900 | NA |
| ENSG00000148343 | MIGA2 | 84895 | 1,157.55 | -0.356 | 0.126 | -2.826 | 0.004700 | 0.063100 | NA |
| ENSG00000158552 | ZFAND2B | 130617 | 656.201 | -0.356 | 0.131 | -2.708 | 0.006800 | 0.079700 | NA |
| ENSG00000132535 | DLG4 | 1742 | 864.926 | -0.355 | 0.13 | -2.736 | 0.006200 | 0.075500 | NA |
| ENSG00000174437 | ATP2A2 | 488 | 14,361.35 | -0.353 | 0.088 | -4.014 | 0.000100 | 0.002200 | NA |
| ENSG00000179134 | SAMD4B | 55095 | 4,791.44 | -0.353 | 0.119 | -2.965 | 0.003000 | 0.047800 | NA |
| ENSG00000144283 | PKP4 | 8502 | 2,023.99 | -0.352 | 0.104 | -3.373 | 0.000700 | 0.016900 | NA |
| ENSG00000106299 | WASL | 8976 | 1,512.06 | -0.351 | 0.12 | -2.926 | 0.003400 | 0.052200 | 57 |
| ENSG00000116991 | SIPA1L2 | 57568 | 1,515.59 | -0.351 | 0.115 | -3.053 | 0.002300 | 0.038700 | 74 |
| ENSG00000168528 | SERINC2 | 347735 | 7,609.62 | -0.351 | 0.121 | -2.913 | 0.003600 | 0.053300 | NA |
| ENSG00000130558 | OLFM1 | 10439 | 316.139 | -0.35 | 0.133 | -2.625 | 0.008700 | 0.094400 | 88 |
| ENSG00000154096 | THY1 | 7070 | 40,148.38 | -0.35 | 0.101 | -3.462 | 0.000500 | 0.013100 | NA |
| ENSG00000183207 | RUVBL2 | 10856 | 3,268.89 | -0.35 | 0.118 | -2.955 | 0.003100 | 0.049000 | NA |
| ENSG00000178057 | NDUFAF3 | 25915 | 1,344.49 | -0.35 | 0.124 | -2.815 | 0.004900 | 0.064400 | NA |
| ENSG00000159164 | SV2A | 9900 | 778.93 | -0.35 | 0.125 | -2.807 | 0.005000 | 0.065300 | NA |
| ENSG00000162520 | SYNC | 81493 | 1,286.47 | -0.35 | 0.13 | -2.694 | 0.007100 | 0.081500 | NA |
| ENSG00000140948 | ZCCHC14 | 23174 | 1,021.50 | -0.349 | 0.108 | -3.246 | 0.001200 | 0.023900 | 72 |
| ENSG00000111843 | TMEM14C | 51522 | 2,814.00 | -0.349 | 0.09 | -3.893 | 0.000100 | 0.003400 | NA |
| ENSG00000110717 | NDUFS8 | 4728 | 2,593.97 | -0.349 | 0.125 | -2.791 | 0.005300 | 0.067500 | NA |
| ENSG00000102898 | NUTF2 | 10204 | 3,167.91 | -0.347 | 0.092 | -3.767 | 0.000200 | 0.005100 | 67 |
| ENSG00000170017 | ALCAM | 214 | 9,177.71 | -0.347 | 0.095 | -3.639 | 0.000300 | 0.007600 | 80 |
| ENSG00000137411 | VARS2 | 57176 | 958.895 | -0.347 | 0.128 | -2.709 | 0.006800 | 0.079600 | NA |
| ENSG00000029534 | ANK1 | 286 | 792.561 | -0.346 | 0.132 | -2.614 | 0.008900 | 0.096700 | NA |
| ENSG00000047849 | MAP4 | 4134 | 22,085.37 | -0.345 | 0.092 | -3.769 | 0.000200 | 0.005100 | NA |
| ENSG00000196155 | PLEKHG4 | 25894 | 2,229.36 | -0.345 | 0.133 | -2.597 | 0.009400 | 0.100000 | NA |
| ENSG00000176834 | VSIG10 | 54621 | 656.907 | -0.344 | 0.105 | -3.264 | 0.001100 | 0.022700 | NA |
| ENSG00000162104 | ADCY9 | 115 | 3,168.30 | -0.343 | 0.106 | -3.239 | 0.001200 | 0.024400 | NA |
| ENSG00000221968 | FADS3 | 3995 | 4,683.65 | -0.343 | 0.115 | -2.981 | 0.002900 | 0.046000 | NA |
| ENSG00000240849 | PEDS1 | 387521 | 1,368.46 | -0.342 | 0.091 | -3.78 | 0.000200 | 0.004900 | NA |
| ENSG00000064961 | HMG20B | 10362 | 1,777.97 | -0.342 | 0.121 | -2.833 | 0.004600 | 0.062200 | NA |
| ENSG00000128487 | SPECC1 | 92521 | 424.904 | -0.341 | 0.131 | -2.606 | 0.009200 | 0.098500 | NA |
| ENSG00000004897 | CDC27 | 996 | 3,169.95 | -0.34 | 0.118 | -2.882 | 0.003900 | 0.056800 | 57 |
| ENSG00000165669 | FAM204A | 63877 | 962.849 | -0.34 | 0.106 | -3.222 | 0.001300 | 0.025500 | NA |
| ENSG00000165801 | ARHGEF40 | 55701 | 5,296.11 | -0.34 | 0.113 | -3.013 | 0.002600 | 0.042300 | NA |
| ENSG00000148331 | ASB6 | 140459 | 1,851.48 | -0.34 | 0.119 | -2.865 | 0.004200 | 0.058600 | NA |
| ENSG00000010295 | IFFO1 | 25900 | 2,052.79 | -0.34 | 0.12 | -2.832 | 0.004600 | 0.062300 | NA |
| ENSG00000146122 | DAAM2 | 23500 | 1,650.66 | -0.34 | 0.129 | -2.633 | 0.008500 | 0.093000 | NA |
| ENSG00000128708 | HAT1 | 8520 | 1,504.71 | -0.34 | 0.13 | -2.607 | 0.009100 | 0.098300 | NA |
| ENSG00000182054 | IDH2 | 3418 | 2,246.27 | -0.339 | 0.099 | -3.427 | 0.000600 | 0.014500 | NA |
| ENSG00000004975 | DVL2 | 1856 | 2,066.48 | -0.339 | 0.106 | -3.207 | 0.001300 | 0.026300 | NA |
| ENSG00000001461 | NIPAL3 | 57185 | 8,647.26 | -0.338 | 0.104 | -3.267 | 0.001100 | 0.022600 | NA |
| ENSG00000121440 | PDZRN3 | 23024 | 1,554.54 | -0.337 | 0.102 | -3.288 | 0.001000 | 0.021400 | NA |
| ENSG00000048140 | TSPAN17 | 26262 | 3,413.29 | -0.337 | 0.118 | -2.859 | 0.004300 | 0.059100 | NA |
| ENSG00000128641 | MYO1B | 4430 | 10,698.64 | -0.336 | 0.108 | -3.122 | 0.001800 | 0.032800 | 57 |
| ENSG00000005194 | CIAPIN1 | 57019 | 1,853.84 | -0.336 | 0.088 | -3.803 | 0.000100 | 0.004500 | NA |
| ENSG00000123992 | DNPEP | 23549 | 2,540.18 | -0.335 | 0.111 | -3.022 | 0.002500 | 0.041400 | NA |
| ENSG00000160326 | SLC2A6 | 11182 | 1,730.17 | -0.335 | 0.128 | -2.625 | 0.008700 | 0.094400 | NA |
| ENSG00000108861 | DUSP3 | 1845 | 7,093.86 | -0.334 | 0.078 | -4.3 | 0.000000 | 0.000800 | NA |
| ENSG00000204843 | DCTN1 | 1639 | 10,662.00 | -0.333 | 0.098 | -3.411 | 0.000600 | 0.015200 | NA |
| ENSG00000119139 | TJP2 | 9414 | 1,069.36 | -0.333 | 0.102 | -3.253 | 0.001100 | 0.023400 | NA |
| ENSG00000148842 | CNNM2 | 54805 | 556.865 | -0.333 | 0.117 | -2.834 | 0.004600 | 0.062100 | NA |
| ENSG00000176463 | SLCO3A1 | 28232 | 1,312.41 | -0.333 | 0.124 | -2.687 | 0.007200 | 0.082700 | NA |
| ENSG00000135387 | CAPRIN1 | 4076 | 12,467.79 | -0.332 | 0.087 | -3.825 | 0.000100 | 0.004200 | 76 |
| ENSG00000117122 | MFAP2 | 4237 | 1,996.33 | -0.332 | 0.114 | -2.905 | 0.003700 | 0.054200 | NA |
| ENSG00000197355 | UAP1L1 | 91373 | 4,835.71 | -0.331 | 0.112 | -2.963 | 0.003000 | 0.048000 | NA |
| ENSG00000156873 | PHKG2 | 5261 | 628.62 | -0.331 | 0.12 | -2.757 | 0.005800 | 0.072400 | NA |
| ENSG00000205221 | VIT | 5212 | 1,213.30 | -0.331 | 0.127 | -2.617 | 0.008900 | 0.096300 | NA |
| ENSG00000188229 | TUBB4B | 10383 | 22,723.24 | -0.33 | 0.1 | -3.291 | 0.001000 | 0.021200 | NA |
| ENSG00000115318 | LOXL3 | 84695 | 2,730.30 | -0.33 | 0.123 | -2.672 | 0.007500 | 0.085500 | NA |
| ENSG00000140836 | ZFHX3 | 463 | 685.999 | -0.33 | 0.123 | -2.67 | 0.007600 | 0.085900 | NA |
| ENSG00000154832 | CXXC1 | 30827 | 1,592.66 | -0.329 | 0.106 | -3.099 | 0.001900 | 0.034800 | NA |
| ENSG00000100280 | AP1B1 | 162 | 6,989.55 | -0.329 | 0.117 | -2.801 | 0.005100 | 0.066200 | NA |
| ENSG00000197798 | FAM118B | 79607 | 650.553 | -0.328 | 0.114 | -2.873 | 0.004100 | 0.057700 | NA |
| ENSG00000126453 | BCL2L12 | 83596 | 840.877 | -0.328 | 0.119 | -2.747 | 0.006000 | 0.074200 | NA |
| ENSG00000186716 | BCR | 613 | 2,202.95 | -0.328 | 0.121 | -2.723 | 0.006500 | 0.077300 | NA |
| ENSG00000132589 | FLOT2 | 2319 | 2,690.32 | -0.326 | 0.097 | -3.37 | 0.000800 | 0.017000 | NA |
| ENSG00000139405 | RITA1 | 84934 | 889.799 | -0.326 | 0.113 | -2.87 | 0.004100 | 0.058100 | NA |
| ENSG00000168807 | SNTB2 | 6645 | 1,490.88 | -0.326 | 0.124 | -2.641 | 0.008300 | 0.091500 | NA |
| ENSG00000173511 | VEGFB | 7423 | 5,484.31 | -0.326 | 0.124 | -2.637 | 0.008400 | 0.092200 | NA |
| ENSG00000143324 | XPR1 | 9213 | 1,176.95 | -0.325 | 0.124 | -2.616 | 0.008900 | 0.096400 | 68 |
| ENSG00000127418 | FGFRL1 | 53834 | 3,180.62 | -0.324 | 0.111 | -2.919 | 0.003500 | 0.052500 | NA |
| ENSG00000155090 | KLF10 | 7071 | 2,174.15 | -0.323 | 0.086 | -3.766 | 0.000200 | 0.005100 | NA |
| ENSG00000068903 | SIRT2 | 22933 | 2,436.34 | -0.323 | 0.101 | -3.184 | 0.001500 | 0.028000 | NA |
| ENSG00000008256 | CYTH3 | 9265 | 3,326.48 | -0.323 | 0.123 | -2.628 | 0.008600 | 0.094100 | NA |
| ENSG00000135446 | CDK4 | 1019 | 3,513.16 | -0.322 | 0.101 | -3.187 | 0.001400 | 0.027800 | NA |
| ENSG00000181090 | EHMT1 | 79813 | 1,438.28 | -0.322 | 0.103 | -3.139 | 0.001700 | 0.031300 | NA |
| ENSG00000116793 | PHTF1 | 10745 | 1,209.92 | -0.322 | 0.108 | -2.999 | 0.002700 | 0.044100 | NA |
| ENSG00000171604 | CXXC5 | 51523 | 1,412.44 | -0.322 | 0.115 | -2.801 | 0.005100 | 0.066200 | NA |
| ENSG00000113369 | ARRDC3 | 57561 | 1,978.35 | -0.32 | 0.097 | -3.292 | 0.001000 | 0.021200 | 59 |
| ENSG00000164402 | SEPTIN8 | 23176 | 2,783.68 | -0.32 | 0.099 | -3.251 | 0.001100 | 0.023500 | NA |
| ENSG00000157613 | CREB3L1 | 90993 | 11,042.22 | -0.32 | 0.1 | -3.204 | 0.001400 | 0.026500 | NA |
| ENSG00000126581 | BECN1 | 8678 | 2,010.26 | -0.32 | 0.112 | -2.863 | 0.004200 | 0.058800 | NA |
| ENSG00000214022 | REPIN1 | 29803 | 3,565.99 | -0.32 | 0.118 | -2.715 | 0.006600 | 0.078400 | NA |
| ENSG00000149136 | SSRP1 | 6749 | 4,027.77 | -0.317 | 0.086 | -3.701 | 0.000200 | 0.006200 | NA |
| ENSG00000173517 | PEAK1 | 79834 | 3,523.34 | -0.317 | 0.09 | -3.532 | 0.000400 | 0.010700 | NA |
| ENSG00000162144 | CYB561A3 | 220002 | 2,780.25 | -0.315 | 0.105 | -3.01 | 0.002600 | 0.042800 | NA |
| ENSG00000011105 | TSPAN9 | 10867 | 1,343.27 | -0.314 | 0.109 | -2.885 | 0.003900 | 0.056500 | NA |
| ENSG00000090924 | PLEKHG2 | 64857 | 1,366.39 | -0.312 | 0.102 | -3.055 | 0.002200 | 0.038500 | NA |
| ENSG00000099797 | TECR | 9524 | 2,131.37 | -0.311 | 0.093 | -3.349 | 0.000800 | 0.018100 | NA |
| ENSG00000159788 | RGS12 | 6002 | 712.43 | -0.311 | 0.106 | -2.931 | 0.003400 | 0.051500 | NA |
| ENSG00000166963 | MAP1A | 4130 | 14,875.09 | -0.31 | 0.088 | -3.518 | 0.000400 | 0.011100 | NA |
| ENSG00000148516 | ZEB1 | 6935 | 2,284.56 | -0.31 | 0.116 | -2.667 | 0.007600 | 0.086200 | NA |
| ENSG00000089060 | SLC8B1 | 80024 | 968.19 | -0.31 | 0.117 | -2.641 | 0.008300 | 0.091500 | NA |
| ENSG00000100439 | ABHD4 | 63874 | 1,880.72 | -0.309 | 0.108 | -2.869 | 0.004100 | 0.058100 | NA |
| ENSG00000172500 | FIBP | 9158 | 2,317.76 | -0.308 | 0.092 | -3.343 | 0.000800 | 0.018400 | NA |
| ENSG00000105270 | CLIP3 | 25999 | 2,360.72 | -0.308 | 0.107 | -2.881 | 0.004000 | 0.056900 | NA |
| ENSG00000171608 | PIK3CD | 5293 | 1,878.42 | -0.306 | 0.105 | -2.929 | 0.003400 | 0.051700 | NA |
| ENSG00000198055 | GRK6 | 2870 | 1,124.07 | -0.306 | 0.105 | -2.917 | 0.003500 | 0.052600 | NA |
| ENSG00000149557 | FEZ1 | 9638 | 1,596.17 | -0.305 | 0.094 | -3.232 | 0.001200 | 0.024800 | NA |
| ENSG00000167815 | PRDX2 | 7001 | 2,853.27 | -0.305 | 0.098 | -3.102 | 0.001900 | 0.034500 | NA |
| ENSG00000132471 | WBP2 | 23558 | 5,124.36 | -0.305 | 0.106 | -2.869 | 0.004100 | 0.058100 | NA |
| ENSG00000103994 | ZNF106 | 64397 | 4,294.59 | -0.305 | 0.113 | -2.696 | 0.007000 | 0.081400 | NA |
| ENSG00000107263 | RAPGEF1 | 2889 | 4,013.74 | -0.304 | 0.089 | -3.423 | 0.000600 | 0.014700 | NA |
| ENSG00000109079 | TNFAIP1 | 7126 | 3,974.92 | -0.303 | 0.103 | -2.933 | 0.003400 | 0.051300 | NA |
| ENSG00000160445 | ZER1 | 10444 | 1,830.00 | -0.302 | 0.108 | -2.784 | 0.005400 | 0.068400 | NA |
| ENSG00000102226 | USP11 | 8237 | 1,929.88 | -0.301 | 0.103 | -2.924 | 0.003500 | 0.052300 | NA |
| ENSG00000198420 | TCAF1 | 9747 | 1,480.49 | -0.301 | 0.104 | -2.885 | 0.003900 | 0.056500 | NA |
| ENSG00000166311 | SMPD1 | 6609 | 10,998.26 | -0.301 | 0.11 | -2.739 | 0.006200 | 0.075200 | NA |
| ENSG00000105443 | CYTH2 | 9266 | 1,366.78 | -0.3 | 0.108 | -2.777 | 0.005500 | 0.069600 | NA |
| ENSG00000103365 | GGA2 | 23062 | 2,460.12 | -0.299 | 0.095 | -3.149 | 0.001600 | 0.030400 | NA |
| ENSG00000127241 | MASP1 | 5648 | 4,960.45 | -0.299 | 0.115 | -2.603 | 0.009200 | 0.098800 | NA |
| ENSG00000107164 | FUBP3 | 8939 | 2,782.47 | -0.297 | 0.091 | -3.268 | 0.001100 | 0.022600 | 78 |
| ENSG00000006282 | SPATA20 | 64847 | 3,941.87 | -0.296 | 0.11 | -2.705 | 0.006800 | 0.080100 | NA |
| ENSG00000047644 | WWC3 | 55841 | 1,318.65 | -0.295 | 0.096 | -3.068 | 0.002200 | 0.037400 | NA |
| ENSG00000177963 | RIC8A | 60626 | 6,797.65 | -0.294 | 0.095 | -3.09 | 0.002000 | 0.035500 | NA |
| ENSG00000163513 | TGFBR2 | 7048 | 8,051.58 | -0.294 | 0.101 | -2.897 | 0.003800 | 0.055200 | NA |
| ENSG00000122870 | BICC1 | 80114 | 2,092.60 | -0.292 | 0.101 | -2.897 | 0.003800 | 0.055200 | NA |
| ENSG00000183160 | TMEM119 | 338773 | 12,904.80 | -0.291 | 0.112 | -2.605 | 0.009200 | 0.098600 | NA |
| ENSG00000132591 | ERAL1 | 26284 | 1,609.95 | -0.29 | 0.094 | -3.093 | 0.002000 | 0.035300 | NA |
| ENSG00000105281 | SLC1A5 | 6510 | 6,109.29 | -0.29 | 0.101 | -2.867 | 0.004100 | 0.058200 | NA |
| ENSG00000085662 | AKR1B1 | 231 | 11,717.44 | -0.29 | 0.104 | -2.799 | 0.005100 | 0.066400 | NA |
| ENSG00000183255 | PTTG1IP | 754 | 10,231.72 | -0.288 | 0.089 | -3.255 | 0.001100 | 0.023300 | NA |
| ENSG00000010810 | FYN | 2534 | 1,659.23 | -0.288 | 0.1 | -2.877 | 0.004000 | 0.057300 | NA |
| ENSG00000071462 | BUD23 | 114049 | 1,872.49 | -0.287 | 0.1 | -2.852 | 0.004300 | 0.059800 | NA |
| ENSG00000090316 | MAEA | 10296 | 2,785.78 | -0.287 | 0.103 | -2.782 | 0.005400 | 0.068600 | NA |
| ENSG00000186951 | PPARA | 5465 | 1,033.57 | -0.286 | 0.107 | -2.68 | 0.007400 | 0.084000 | 68 |
| ENSG00000140443 | IGF1R | 3480 | 1,914.88 | -0.284 | 0.09 | -3.144 | 0.001700 | 0.030900 | 88 |
| ENSG00000148120 | AOPEP | 84909 | 3,109.24 | -0.283 | 0.08 | -3.532 | 0.000400 | 0.010700 | NA |
| ENSG00000278259 | MYO19 | 80179 | 1,625.81 | -0.281 | 0.092 | -3.048 | 0.002300 | 0.039300 | NA |
| ENSG00000123136 | DDX39A | 10212 | 3,064.62 | -0.281 | 0.096 | -2.931 | 0.003400 | 0.051400 | NA |
| ENSG00000116062 | MSH6 | 2956 | 1,945.27 | -0.279 | 0.097 | -2.874 | 0.004100 | 0.057700 | NA |
| ENSG00000129116 | PALLD | 23022 | 4,682.63 | -0.274 | 0.1 | -2.723 | 0.006500 | 0.077300 | NA |
| ENSG00000170633 | RNF34 | 80196 | 1,064.82 | -0.273 | 0.095 | -2.863 | 0.004200 | 0.058800 | 76 |
| ENSG00000275066 | SYNRG | 11276 | 2,008.74 | -0.271 | 0.099 | -2.743 | 0.006100 | 0.074600 | 64 |
| ENSG00000102034 | ELF4 | 2000 | 2,801.32 | -0.271 | 0.103 | -2.635 | 0.008400 | 0.092700 | NA |
| ENSG00000110090 | CPT1A | 1374 | 4,292.83 | -0.271 | 0.104 | -2.605 | 0.009200 | 0.098600 | NA |
| ENSG00000054148 | PHPT1 | 29085 | 2,441.25 | -0.27 | 0.104 | -2.607 | 0.009100 | 0.098300 | NA |
| ENSG00000170348 | TMED10 | 10972 | 8,016.13 | -0.269 | 0.092 | -2.929 | 0.003400 | 0.051700 | NA |
| ENSG00000117362 | APH1A | 51107 | 4,423.98 | -0.269 | 0.096 | -2.79 | 0.005300 | 0.067600 | NA |
| ENSG00000129353 | SLC44A2 | 57153 | 9,008.53 | -0.268 | 0.098 | -2.745 | 0.006100 | 0.074300 | NA |
| ENSG00000065000 | AP3D1 | 8943 | 8,769.95 | -0.268 | 0.099 | -2.721 | 0.006500 | 0.077600 | NA |
| ENSG00000083857 | FAT1 | 2195 | 8,698.94 | -0.265 | 0.093 | -2.85 | 0.004400 | 0.059900 | 55 |
| ENSG00000135250 | SRPK2 | 6733 | 1,602.46 | -0.263 | 0.096 | -2.741 | 0.006100 | 0.074900 | NA |
| ENSG00000118689 | FOXO3 | 2309 | 2,214.24 | -0.263 | 0.099 | -2.642 | 0.008200 | 0.091300 | NA |
| ENSG00000123342 | MMP19 | 4327 | 925.224 | -0.263 | 0.101 | -2.611 | 0.009000 | 0.097400 | NA |
| ENSG00000065534 | MYLK | 4638 | 10,626.72 | -0.26 | 0.082 | -3.16 | 0.001600 | 0.029700 | NA |
| ENSG00000113716 | HMGXB3 | 22993 | 3,341.29 | -0.259 | 0.085 | -3.06 | 0.002200 | 0.038100 | NA |
| ENSG00000144677 | CTDSPL | 10217 | 1,533.04 | -0.257 | 0.095 | -2.717 | 0.006600 | 0.078200 | NA |
| ENSG00000120885 | CLU | 1191 | 4,957.22 | -0.257 | 0.097 | -2.655 | 0.007900 | 0.088700 | NA |
| ENSG00000065357 | DGKA | 1606 | 1,951.44 | -0.254 | 0.087 | -2.938 | 0.003300 | 0.050700 | NA |
| ENSG00000154978 | VOPP1 | 81552 | 3,772.30 | -0.253 | 0.089 | -2.829 | 0.004700 | 0.062500 | NA |
| ENSG00000006042 | TMEM98 | 26022 | 1,578.51 | -0.253 | 0.091 | -2.77 | 0.005600 | 0.070500 | NA |
| ENSG00000075426 | FOSL2 | 2355 | 5,673.86 | -0.251 | 0.094 | -2.687 | 0.007200 | 0.082700 | NA |
| ENSG00000167693 | NXN | 64359 | 4,499.78 | -0.25 | 0.082 | -3.057 | 0.002200 | 0.038400 | NA |
| ENSG00000168036 | CTNNB1 | 1499 | 10,530.43 | -0.249 | 0.078 | -3.182 | 0.001500 | 0.028000 | NA |
| ENSG00000166025 | AMOTL1 | 154810 | 3,324.40 | -0.249 | 0.089 | -2.792 | 0.005200 | 0.067400 | NA |
| ENSG00000174705 | SH3PXD2B | 285590 | 4,735.66 | -0.241 | 0.092 | -2.613 | 0.009000 | 0.097000 | NA |
| ENSG00000138944 | SHISAL1 | 85352 | 11,600.09 | -0.239 | 0.083 | -2.894 | 0.003800 | 0.055500 | NA |
| ENSG00000004455 | AK2 | 204 | 3,556.60 | -0.204 | 0.076 | -2.666 | 0.007700 | 0.086500 | NA |
| ENSG00000144043 | TEX261 | 113419 | 4,360.98 | 0.217 | 0.081 | 2.673 | 0.007500 | 0.085400 | NA |
| ENSG00000264364 | DYNLL2 | 140735 | 4,576.10 | 0.225 | 0.085 | 2.66 | 0.007800 | 0.087700 | NA |
| ENSG00000035687 | ADSS2 | 159 | 2,462.79 | 0.228 | 0.087 | 2.615 | 0.008900 | 0.096600 | NA |
| ENSG00000136193 | SCRN1 | 9805 | 9,558.76 | 0.232 | 0.085 | 2.719 | 0.006500 | 0.077800 | NA |
| ENSG00000100216 | TOMM22 | 56993 | 2,483.03 | 0.233 | 0.083 | 2.812 | 0.004900 | 0.064800 | NA |
| ENSG00000140598 | EFL1 | 79631 | 2,073.01 | 0.234 | 0.09 | 2.6 | 0.009300 | 0.099400 | NA |
| ENSG00000162407 | PLPP3 | 8613 | 8,443.62 | 0.241 | 0.088 | 2.745 | 0.006000 | 0.074300 | NA |
| ENSG00000276293 | PIP4K2B | 8396 | 3,873.03 | 0.241 | 0.09 | 2.687 | 0.007200 | 0.082700 | NA |
| ENSG00000160679 | CHTOP | 26097 | 1,645.26 | 0.244 | 0.088 | 2.776 | 0.005500 | 0.069600 | NA |
| ENSG00000175970 | UNC119B | 84747 | 1,373.42 | 0.245 | 0.089 | 2.759 | 0.005800 | 0.072100 | NA |
| ENSG00000067082 | KLF6 | 1316 | 8,401.28 | 0.247 | 0.083 | 2.966 | 0.003000 | 0.047800 | NA |
| ENSG00000135341 | MAP3K7 | 6885 | 2,041.53 | 0.256 | 0.09 | 2.849 | 0.004400 | 0.060000 | NA |
| ENSG00000166326 | TRIM44 | 54765 | 4,649.66 | 0.257 | 0.09 | 2.852 | 0.004400 | 0.059800 | NA |
| ENSG00000205213 | LGR4 | 55366 | 2,355.72 | 0.259 | 0.093 | 2.786 | 0.005300 | 0.068100 | NA |
| ENSG00000058668 | ATP2B4 | 493 | 29,587.11 | 0.26 | 0.096 | 2.714 | 0.006700 | 0.078600 | NA |
| ENSG00000062650 | WAPL | 23063 | 2,008.56 | 0.262 | 0.097 | 2.701 | 0.006900 | 0.080600 | NA |
| ENSG00000071994 | PDCD2 | 5134 | 1,559.27 | 0.264 | 0.096 | 2.751 | 0.005900 | 0.073600 | NA |
| ENSG00000197894 | ADH5 | 128 | 7,602.59 | 0.265 | 0.082 | 3.21 | 0.001300 | 0.026200 | NA |
| ENSG00000156639 | ZFAND3 | 60685 | 3,655.31 | 0.268 | 0.101 | 2.66 | 0.007800 | 0.087700 | NA |
| ENSG00000114503 | NCBP2 | 22916 | 2,539.04 | 0.27 | 0.093 | 2.909 | 0.003600 | 0.053700 | NA |
| ENSG00000131873 | CHSY1 | 22856 | 1,663.29 | 0.273 | 0.094 | 2.894 | 0.003800 | 0.055500 | NA |
| ENSG00000169398 | PTK2 | 5747 | 3,222.06 | 0.274 | 0.091 | 3.025 | 0.002500 | 0.041200 | NA |
| ENSG00000119487 | MAPKAP1 | 79109 | 6,430.14 | 0.276 | 0.093 | 2.96 | 0.003100 | 0.048300 | NA |
| ENSG00000119396 | RAB14 | 51552 | 3,561.44 | 0.276 | 0.099 | 2.788 | 0.005300 | 0.067800 | NA |
| ENSG00000121022 | COPS5 | 10987 | 1,330.70 | 0.277 | 0.09 | 3.092 | 0.002000 | 0.035400 | NA |
| ENSG00000144468 | RHBDD1 | 84236 | 972.2 | 0.278 | 0.098 | 2.825 | 0.004700 | 0.063100 | NA |
| ENSG00000119616 | FCF1 | 51077 | 1,367.23 | 0.278 | 0.102 | 2.736 | 0.006200 | 0.075500 | NA |
| ENSG00000070778 | PTPN21 | 11099 | 1,194.11 | 0.28 | 0.097 | 2.886 | 0.003900 | 0.056500 | NA |
| ENSG00000227051 | C14orf132 | 56967 | 3,238.26 | 0.28 | 0.103 | 2.72 | 0.006500 | 0.077700 | NA |
| ENSG00000133313 | CNDP2 | 55748 | 4,817.21 | 0.281 | 0.099 | 2.848 | 0.004400 | 0.060100 | NA |
| ENSG00000075413 | MARK3 | 4140 | 2,496.07 | 0.281 | 0.103 | 2.728 | 0.006400 | 0.076800 | NA |
| ENSG00000135457 | TFCP2 | 7024 | 1,128.04 | 0.282 | 0.097 | 2.894 | 0.003800 | 0.055500 | NA |
| ENSG00000115145 | STAM2 | 10254 | 1,285.67 | 0.283 | 0.102 | 2.782 | 0.005400 | 0.068600 | NA |
| ENSG00000146648 | EGFR | 1956 | 13,895.28 | 0.284 | 0.104 | 2.737 | 0.006200 | 0.075500 | NA |
| ENSG00000167447 | SMG8 | 55181 | 1,018.21 | 0.285 | 0.101 | 2.811 | 0.004900 | 0.064800 | NA |
| ENSG00000013503 | POLR3B | 55703 | 620.396 | 0.285 | 0.106 | 2.696 | 0.007000 | 0.081400 | NA |
| ENSG00000156928 | MALSU1 | 115416 | 718.614 | 0.285 | 0.106 | 2.694 | 0.007100 | 0.081500 | NA |
| ENSG00000114098 | ARMC8 | 25852 | 1,149.27 | 0.286 | 0.1 | 2.859 | 0.004300 | 0.059100 | NA |
| ENSG00000244462 | RBM12 | 10137 | 2,020.80 | 0.286 | 0.109 | 2.627 | 0.008600 | 0.094100 | NA |
| ENSG00000127870 | RNF6 | 6049 | 2,460.31 | 0.291 | 0.097 | 2.998 | 0.002700 | 0.044100 | NA |
| ENSG00000133961 | NUMB | 8650 | 3,542.41 | 0.292 | 0.08 | 3.655 | 0.000300 | 0.007300 | NA |
| ENSG00000265241 | RBM8A | 9939 | 1,045.30 | 0.292 | 0.107 | 2.723 | 0.006500 | 0.077300 | NA |
| ENSG00000179119 | SPTY2D1 | 144108 | 1,267.34 | 0.292 | 0.107 | 2.723 | 0.006500 | 0.077300 | NA |
| ENSG00000171466 | ZNF562 | 54811 | 988.312 | 0.293 | 0.113 | 2.6 | 0.009300 | 0.099500 | NA |
| ENSG00000139218 | SCAF11 | 9169 | 2,462.13 | 0.294 | 0.113 | 2.614 | 0.008900 | 0.096700 | NA |
| ENSG00000090470 | PDCD7 | 10081 | 869.025 | 0.295 | 0.097 | 3.035 | 0.002400 | 0.040400 | NA |
| ENSG00000260032 | NORAD | 647979 | 15,594.90 | 0.297 | 0.1 | 2.969 | 0.003000 | 0.047500 | NA |
| ENSG00000221914 | PPP2R2A | 5520 | 2,104.23 | 0.297 | 0.101 | 2.95 | 0.003200 | 0.049600 | NA |
| ENSG00000178695 | KCTD12 | 115207 | 7,103.80 | 0.299 | 0.112 | 2.662 | 0.007800 | 0.087300 | NA |
| ENSG00000106105 | GARS1 | 2617 | 13,318.14 | 0.3 | 0.077 | 3.871 | 0.000100 | 0.003700 | NA |
| ENSG00000171109 | MFN1 | 55669 | 1,540.21 | 0.3 | 0.111 | 2.705 | 0.006800 | 0.080100 | NA |
| ENSG00000154124 | OTULIN | 90268 | 861.21 | 0.301 | 0.114 | 2.63 | 0.008500 | 0.093800 | NA |
| ENSG00000210196 |  |  | 1,757.52 | 0.302 | 0.103 | 2.942 | 0.003300 | 0.050500 | NA |
| ENSG00000029364 | SLC39A9 | 55334 | 2,733.24 | 0.303 | 0.11 | 2.741 | 0.006100 | 0.074800 | NA |
| ENSG00000135999 | EPC2 | 26122 | 560.447 | 0.303 | 0.113 | 2.687 | 0.007200 | 0.082700 | NA |
| ENSG00000104332 | SFRP1 | 6422 | 25,912.88 | 0.304 | 0.095 | 3.189 | 0.001400 | 0.027500 | NA |
| ENSG00000008869 | HEATR5B | 54497 | 817.598 | 0.306 | 0.111 | 2.744 | 0.006100 | 0.074300 | NA |
| ENSG00000136802 | LRRC8A | 56262 | 3,994.06 | 0.307 | 0.098 | 3.137 | 0.001700 | 0.031400 | NA |
| ENSG00000135775 | COG2 | 22796 | 658.576 | 0.307 | 0.114 | 2.698 | 0.007000 | 0.081100 | NA |
| ENSG00000110841 | PPFIBP1 | 8496 | 2,286.64 | 0.308 | 0.108 | 2.857 | 0.004300 | 0.059200 | NA |
| ENSG00000120875 | DUSP4 | 1846 | 2,613.69 | 0.308 | 0.108 | 2.854 | 0.004300 | 0.059600 | NA |
| ENSG00000142867 | BCL10 | 8915 | 949.542 | 0.309 | 0.119 | 2.597 | 0.009400 | 0.099900 | NA |
| ENSG00000168818 | STX18 | 53407 | 1,244.47 | 0.311 | 0.092 | 3.399 | 0.000700 | 0.015700 | NA |
| ENSG00000117222 | RBBP5 | 5929 | 844.184 | 0.311 | 0.11 | 2.843 | 0.004500 | 0.061000 | NA |
| ENSG00000150776 | NKAPD1 | 55216 | 860.384 | 0.311 | 0.11 | 2.838 | 0.004500 | 0.061700 | NA |
| ENSG00000139746 | RBM26 | 64062 | 1,411.42 | 0.311 | 0.117 | 2.66 | 0.007800 | 0.087700 | NA |
| ENSG00000105778 | AVL9 | 23080 | 2,421.24 | 0.312 | 0.115 | 2.721 | 0.006500 | 0.077600 | NA |
| ENSG00000166411 | IDH3A | 3419 | 907.672 | 0.312 | 0.115 | 2.707 | 0.006800 | 0.079800 | NA |
| ENSG00000102580 | DNAJC3 | 5611 | 2,623.07 | 0.312 | 0.116 | 2.679 | 0.007400 | 0.084100 | NA |
| ENSG00000103241 | FOXF1 | 2294 | 3,000.94 | 0.314 | 0.115 | 2.729 | 0.006300 | 0.076600 | NA |
| ENSG00000170860 | LSM3 | 27258 | 1,374.96 | 0.314 | 0.116 | 2.704 | 0.006900 | 0.080200 | NA |
| ENSG00000165322 | ARHGAP12 | 94134 | 2,330.84 | 0.315 | 0.112 | 2.818 | 0.004800 | 0.064100 | NA |
| ENSG00000198961 | PJA2 | 9867 | 3,513.67 | 0.316 | 0.121 | 2.611 | 0.009000 | 0.097400 | NA |
| ENSG00000156273 | BACH1 | 571 | 1,370.54 | 0.317 | 0.102 | 3.112 | 0.001900 | 0.033700 | NA |
| ENSG00000115946 | PNO1 | 56902 | 900.469 | 0.317 | 0.122 | 2.6 | 0.009300 | 0.099400 | NA |
| ENSG00000254087 | LYN | 4067 | 1,499.69 | 0.318 | 0.112 | 2.835 | 0.004600 | 0.062100 | NA |
| ENSG00000152256 | PDK1 | 5163 | 415.23 | 0.318 | 0.121 | 2.626 | 0.008600 | 0.094100 | NA |
| ENSG00000154734 | ADAMTS1 | 9510 | 9,645.06 | 0.32 | 0.118 | 2.709 | 0.006700 | 0.079500 | NA |
| ENSG00000138166 | DUSP5 | 1847 | 2,276.14 | 0.321 | 0.115 | 2.795 | 0.005200 | 0.067000 | NA |
| ENSG00000181704 | YIPF6 | 286451 | 1,802.60 | 0.321 | 0.119 | 2.695 | 0.007000 | 0.081500 | NA |
| ENSG00000141564 | RPTOR | 57521 | 2,296.77 | 0.322 | 0.102 | 3.172 | 0.001500 | 0.028700 | NA |
| ENSG00000125676 | THOC2 | 57187 | 1,561.40 | 0.322 | 0.116 | 2.776 | 0.005500 | 0.069600 | NA |
| ENSG00000173559 | NABP1 | 64859 | 3,285.03 | 0.322 | 0.119 | 2.698 | 0.007000 | 0.081100 | NA |
| ENSG00000164466 | SFXN1 | 94081 | 2,265.09 | 0.323 | 0.111 | 2.917 | 0.003500 | 0.052600 | NA |
| ENSG00000198900 | TOP1 | 7150 | 4,606.19 | 0.325 | 0.096 | 3.383 | 0.000700 | 0.016500 | NA |
| ENSG00000130396 | AFDN | 4301 | 1,347.38 | 0.325 | 0.107 | 3.032 | 0.002400 | 0.040700 | NA |
| ENSG00000160613 | PCSK7 | 9159 | 1,754.94 | 0.325 | 0.118 | 2.76 | 0.005800 | 0.072000 | NA |
| ENSG00000152242 | C18orf25 | 147339 | 1,397.29 | 0.325 | 0.119 | 2.73 | 0.006300 | 0.076500 | NA |
| ENSG00000108256 | NUFIP2 | 57532 | 4,180.19 | 0.326 | 0.101 | 3.231 | 0.001200 | 0.024800 | NA |
| ENSG00000171150 | SOCS5 | 9655 | 1,421.34 | 0.326 | 0.112 | 2.922 | 0.003500 | 0.052400 | NA |
| ENSG00000196363 | WDR5 | 11091 | 1,926.73 | 0.327 | 0.102 | 3.197 | 0.001400 | 0.026900 | NA |
| ENSG00000011198 | ABHD5 | 51099 | 1,606.30 | 0.328 | 0.098 | 3.332 | 0.000900 | 0.018900 | NA |
| ENSG00000104660 | LEPROTL1 | 23484 | 1,020.44 | 0.328 | 0.124 | 2.642 | 0.008200 | 0.091300 | NA |
| ENSG00000150316 | CWC15 | 51503 | 1,306.18 | 0.329 | 0.113 | 2.902 | 0.003700 | 0.054500 | NA |
| ENSG00000112031 | MTRF1L | 54516 | 611.028 | 0.33 | 0.107 | 3.089 | 0.002000 | 0.035500 | NA |
| ENSG00000144233 | AMMECR1L | 83607 | 1,508.36 | 0.331 | 0.104 | 3.193 | 0.001400 | 0.027200 | NA |
| ENSG00000085491 | SLC25A24 | 29957 | 1,912.82 | 0.331 | 0.118 | 2.793 | 0.005200 | 0.067100 | NA |
| ENSG00000145216 | FIP1L1 | 81608 | 1,215.58 | 0.333 | 0.102 | 3.278 | 0.001000 | 0.022000 | NA |
| ENSG00000110395 | CBL | 867 | 1,819.54 | 0.333 | 0.124 | 2.681 | 0.007300 | 0.083800 | NA |
| ENSG00000128590 | DNAJB9 | 4189 | 684.594 | 0.333 | 0.125 | 2.668 | 0.007600 | 0.086200 | NA |
| ENSG00000179387 | ELMOD2 | 255520 | 605.172 | 0.334 | 0.128 | 2.598 | 0.009400 | 0.099900 | NA |
| ENSG00000109685 | NSD2 | 7468 | 4,564.54 | 0.335 | 0.116 | 2.899 | 0.003700 | 0.055000 | NA |
| ENSG00000123728 | RAP2C | 57826 | 944.285 | 0.335 | 0.118 | 2.83 | 0.004700 | 0.062500 | NA |
| ENSG00000141425 | RPRD1A | 55197 | 1,162.47 | 0.336 | 0.116 | 2.909 | 0.003600 | 0.053700 | NA |
| ENSG00000136875 | PRPF4 | 9128 | 1,497.37 | 0.336 | 0.12 | 2.811 | 0.004900 | 0.064800 | NA |
| ENSG00000232956 | SNHG15 | 285958 | 462.151 | 0.336 | 0.124 | 2.701 | 0.006900 | 0.080600 | NA |
| ENSG00000159873 | CCDC117 | 150275 | 767.693 | 0.337 | 0.122 | 2.774 | 0.005500 | 0.070000 | NA |
| ENSG00000074755 | ZZEF1 | 23140 | 2,402.55 | 0.338 | 0.11 | 3.069 | 0.002100 | 0.037400 | NA |
| ENSG00000189320 | FAM180A | 389558 | 5,548.49 | 0.338 | 0.117 | 2.88 | 0.004000 | 0.056900 | NA |
| ENSG00000128578 | STRIP2 | 57464 | 763.07 | 0.339 | 0.11 | 3.094 | 0.002000 | 0.035200 | NA |
| ENSG00000138660 | AP1AR | 55435 | 545.84 | 0.339 | 0.118 | 2.873 | 0.004100 | 0.057700 | NA |
| ENSG00000099622 | CIRBP | 1153 | 3,858.87 | 0.34 | 0.094 | 3.605 | 0.000300 | 0.008600 | NA |
| ENSG00000079739 | PGM1 | 5236 | 1,955.68 | 0.34 | 0.101 | 3.354 | 0.000800 | 0.017900 | NA |
| ENSG00000152661 | GJA1 | 2697 | 16,567.19 | 0.34 | 0.128 | 2.663 | 0.007700 | 0.087200 | NA |
| ENSG00000151576 | QTRT2 | 79691 | 901.086 | 0.341 | 0.113 | 3.013 | 0.002600 | 0.042300 | NA |
| ENSG00000178234 | GALNT11 | 63917 | 1,289.21 | 0.341 | 0.118 | 2.883 | 0.003900 | 0.056800 | NA |
| ENSG00000116918 | TSNAX | 7257 | 706.215 | 0.341 | 0.12 | 2.832 | 0.004600 | 0.062300 | NA |
| ENSG00000243646 | IL10RB | 3588 | 754.316 | 0.342 | 0.115 | 2.972 | 0.003000 | 0.047000 | NA |
| ENSG00000198121 | LPAR1 | 1902 | 10,470.79 | 0.343 | 0.091 | 3.756 | 0.000200 | 0.005200 | NA |
| ENSG00000185753 | CXorf38 | 159013 | 844.683 | 0.343 | 0.109 | 3.15 | 0.001600 | 0.030400 | NA |
| ENSG00000198873 | GRK5 | 2869 | 1,762.96 | 0.344 | 0.102 | 3.381 | 0.000700 | 0.016500 | NA |
| ENSG00000187742 | SECISBP2 | 79048 | 1,050.04 | 0.345 | 0.106 | 3.255 | 0.001100 | 0.023300 | NA |
| ENSG00000104691 | UBXN8 | 7993 | 509.504 | 0.346 | 0.119 | 2.909 | 0.003600 | 0.053700 | NA |
| ENSG00000153989 | NUS1 | 116150 | 1,570.95 | 0.346 | 0.122 | 2.83 | 0.004600 | 0.062400 | NA |
| ENSG00000131375 | CAPN7 | 23473 | 933.361 | 0.346 | 0.124 | 2.796 | 0.005200 | 0.066700 | NA |
| ENSG00000198860 | TSEN15 | 116461 | 789.054 | 0.348 | 0.119 | 2.92 | 0.003500 | 0.052400 | NA |
| ENSG00000163945 | UVSSA | 57654 | 523.616 | 0.349 | 0.122 | 2.868 | 0.004100 | 0.058200 | NA |
| ENSG00000159200 | RCAN1 | 1827 | 1,993.08 | 0.349 | 0.127 | 2.744 | 0.006100 | 0.074400 | NA |
| ENSG00000100335 | MIEF1 | 54471 | 2,887.58 | 0.35 | 0.084 | 4.141 | 0.000000 | 0.001400 | NA |
| ENSG00000106524 | ANKMY2 | 57037 | 807.872 | 0.35 | 0.111 | 3.153 | 0.001600 | 0.030200 | NA |
| ENSG00000138434 | ITPRID2 | 6744 | 9,868.73 | 0.35 | 0.113 | 3.105 | 0.001900 | 0.034300 | NA |
| ENSG00000176142 | TMEM39A | 55254 | 1,357.42 | 0.35 | 0.116 | 3.026 | 0.002500 | 0.041200 | NA |
| ENSG00000058063 | ATP11B | 23200 | 1,486.20 | 0.351 | 0.106 | 3.3 | 0.001000 | 0.020800 | NA |
| ENSG00000173418 | NAA20 | 51126 | 2,194.91 | 0.352 | 0.103 | 3.418 | 0.000600 | 0.014900 | NA |
| ENSG00000111145 | ELK3 | 2004 | 2,339.43 | 0.352 | 0.117 | 2.992 | 0.002800 | 0.044700 | NA |
| ENSG00000014123 | UFL1 | 23376 | 1,136.81 | 0.352 | 0.118 | 2.976 | 0.002900 | 0.046600 | NA |
| ENSG00000155729 | KCTD18 | 130535 | 560.052 | 0.352 | 0.124 | 2.835 | 0.004600 | 0.062100 | NA |
| ENSG00000107758 | PPP3CB | 5532 | 1,182.65 | 0.352 | 0.128 | 2.75 | 0.006000 | 0.073700 | NA |
| ENSG00000132485 | ZRANB2 | 9406 | 1,338.78 | 0.352 | 0.13 | 2.717 | 0.006600 | 0.078200 | NA |
| ENSG00000164327 | RICTOR | 253260 | 1,007.65 | 0.353 | 0.131 | 2.695 | 0.007000 | 0.081400 | NA |
| ENSG00000075785 | RAB7A | 7879 | 19,795.21 | 0.355 | 0.09 | 3.95 | 0.000100 | 0.002800 | NA |
| ENSG00000103591 | AAGAB | 79719 | 1,996.77 | 0.355 | 0.095 | 3.719 | 0.000200 | 0.005900 | NA |
| ENSG00000135048 | CEMIP2 | 23670 | 3,905.95 | 0.355 | 0.13 | 2.735 | 0.006200 | 0.075700 | NA |
| ENSG00000148634 | HERC4 | 26091 | 6,398.29 | 0.357 | 0.11 | 3.235 | 0.001200 | 0.024600 | NA |
| ENSG00000110092 | CCND1 | 595 | 64,818.50 | 0.358 | 0.113 | 3.164 | 0.001600 | 0.029400 | NA |
| ENSG00000152223 | EPG5 | 57724 | 4,598.23 | 0.358 | 0.124 | 2.885 | 0.003900 | 0.056500 | NA |
| ENSG00000133773 | CCDC59 | 29080 | 460.088 | 0.358 | 0.133 | 2.682 | 0.007300 | 0.083700 | NA |
| ENSG00000127989 | MTERF1 | 7978 | 319.708 | 0.358 | 0.134 | 2.668 | 0.007600 | 0.086200 | NA |
| ENSG00000102531 | FNDC3A | 22862 | 3,446.82 | 0.359 | 0.136 | 2.637 | 0.008400 | 0.092200 | NA |
| ENSG00000138138 | ATAD1 | 84896 | 1,537.92 | 0.36 | 0.117 | 3.066 | 0.002200 | 0.037500 | NA |
| ENSG00000166147 | FBN1 | 2200 | 40,936.60 | 0.362 | 0.107 | 3.393 | 0.000700 | 0.015900 | NA |
| ENSG00000101310 | SEC23B | 10483 | 2,723.10 | 0.363 | 0.107 | 3.382 | 0.000700 | 0.016500 | NA |
| ENSG00000067533 | RRP15 | 51018 | 945.485 | 0.364 | 0.121 | 3.004 | 0.002700 | 0.043400 | NA |
| ENSG00000080371 | RAB21 | 23011 | 1,956.36 | 0.365 | 0.123 | 2.967 | 0.003000 | 0.047700 | NA |
| ENSG00000103044 | HAS3 | 3038 | 613.917 | 0.365 | 0.124 | 2.942 | 0.003300 | 0.050500 | NA |
| ENSG00000140199 | SLC12A6 | 9990 | 1,240.27 | 0.365 | 0.127 | 2.878 | 0.004000 | 0.057200 | NA |
| ENSG00000130348 | QRSL1 | 55278 | 599.531 | 0.366 | 0.11 | 3.322 | 0.000900 | 0.019500 | NA |
| ENSG00000066697 | MSANTD3 | 91283 | 2,565.83 | 0.366 | 0.118 | 3.094 | 0.002000 | 0.035200 | NA |
| ENSG00000146143 | PRIM2 | 5558 | 1,503.16 | 0.366 | 0.135 | 2.704 | 0.006900 | 0.080100 | NA |
| ENSG00000113319 | RASGRF2 | 5924 | 1,451.91 | 0.367 | 0.108 | 3.385 | 0.000700 | 0.016400 | NA |
| ENSG00000055147 | FAM114A2 | 10827 | 780.316 | 0.367 | 0.111 | 3.293 | 0.001000 | 0.021200 | NA |
| ENSG00000163349 | HIPK1 | 204851 | 1,670.88 | 0.367 | 0.114 | 3.214 | 0.001300 | 0.026000 | NA |
| ENSG00000089195 | TRMT6 | 51605 | 797.867 | 0.367 | 0.125 | 2.947 | 0.003200 | 0.049900 | NA |
| ENSG00000163378 | EOGT | 285203 | 1,151.29 | 0.367 | 0.129 | 2.854 | 0.004300 | 0.059600 | NA |
| ENSG00000112715 | VEGFA | 7422 | 7,911.44 | 0.368 | 0.118 | 3.132 | 0.001700 | 0.031900 | NA |
| ENSG00000158966 | CACHD1 | 57685 | 556.104 | 0.368 | 0.13 | 2.82 | 0.004800 | 0.063900 | NA |
| ENSG00000085382 | HACE1 | 57531 | 345.576 | 0.368 | 0.138 | 2.669 | 0.007600 | 0.085900 | NA |
| ENSG00000168172 | HOOK3 | 84376 | 1,466.98 | 0.369 | 0.14 | 2.639 | 0.008300 | 0.091900 | NA |
| ENSG00000198604 | BAZ1A | 11177 | 1,858.59 | 0.37 | 0.128 | 2.879 | 0.004000 | 0.057000 | NA |
| ENSG00000183283 | DAZAP2 | 9802 | 4,575.38 | 0.37 | 0.14 | 2.648 | 0.008100 | 0.090000 | NA |
| ENSG00000124380 | SNRNP27 | 11017 | 735.516 | 0.372 | 0.102 | 3.646 | 0.000300 | 0.007500 | NA |
| ENSG00000196204 | RNF216P1 | 441191 | 698.669 | 0.372 | 0.12 | 3.1 | 0.001900 | 0.034700 | NA |
| ENSG00000115758 | ODC1 | 4953 | 8,929.34 | 0.373 | 0.116 | 3.204 | 0.001400 | 0.026500 | NA |
| ENSG00000134987 | WDR36 | 134430 | 1,111.25 | 0.373 | 0.126 | 2.971 | 0.003000 | 0.047200 | NA |
| ENSG00000010072 | SPRTN | 83932 | 492.858 | 0.373 | 0.126 | 2.959 | 0.003100 | 0.048400 | NA |
| ENSG00000082153 | BZW1 | 9689 | 7,847.43 | 0.374 | 0.117 | 3.211 | 0.001300 | 0.026100 | NA |
| ENSG00000158985 | CDC42SE2 | 56990 | 1,023.39 | 0.374 | 0.137 | 2.737 | 0.006200 | 0.075500 | NA |
| ENSG00000188342 | GTF2F2 | 2963 | 1,416.94 | 0.375 | 0.112 | 3.331 | 0.000900 | 0.019000 | NA |
| ENSG00000008952 | SEC62 | 7095 | 3,573.39 | 0.377 | 0.117 | 3.216 | 0.001300 | 0.025900 | NA |
| ENSG00000146376 | ARHGAP18 | 93663 | 2,181.29 | 0.377 | 0.131 | 2.882 | 0.004000 | 0.056800 | NA |
| ENSG00000080345 | RIF1 | 55183 | 1,324.75 | 0.382 | 0.14 | 2.734 | 0.006300 | 0.075800 | NA |
| ENSG00000075223 | SEMA3C | 10512 | 7,462.75 | 0.383 | 0.124 | 3.104 | 0.001900 | 0.034400 | NA |
| ENSG00000101109 | STK4 | 6789 | 2,157.18 | 0.384 | 0.1 | 3.831 | 0.000100 | 0.004200 | NA |
| ENSG00000185989 | RASA3 | 22821 | 3,301.78 | 0.386 | 0.1 | 3.865 | 0.000100 | 0.003800 | NA |
| ENSG00000127804 | METTL16 | 79066 | 1,020.08 | 0.386 | 0.106 | 3.638 | 0.000300 | 0.007700 | NA |
| ENSG00000184988 | TMEM106A | 113277 | 714.908 | 0.387 | 0.149 | 2.603 | 0.009200 | 0.098900 | NA |
| ENSG00000140350 | ANP32A | 8125 | 2,086.99 | 0.388 | 0.119 | 3.275 | 0.001100 | 0.022200 | NA |
| ENSG00000197343 | ZNF655 | 79027 | 1,383.74 | 0.389 | 0.103 | 3.795 | 0.000100 | 0.004600 | NA |
| ENSG00000105829 | BET1 | 10282 | 1,009.50 | 0.39 | 0.116 | 3.352 | 0.000800 | 0.018000 | NA |
| ENSG00000064313 | TAF2 | 6873 | 1,368.20 | 0.39 | 0.117 | 3.344 | 0.000800 | 0.018400 | NA |
| ENSG00000215301 | DDX3X | 1654 | 9,926.28 | 0.39 | 0.117 | 3.318 | 0.000900 | 0.019700 | NA |
| ENSG00000100522 | GNPNAT1 | 64841 | 1,443.56 | 0.39 | 0.139 | 2.797 | 0.005200 | 0.066700 | NA |
| ENSG00000169991 | IFFO2 | 126917 | 497.741 | 0.395 | 0.12 | 3.297 | 0.001000 | 0.020900 | NA |
| ENSG00000167186 | COQ7 | 10229 | 331.606 | 0.395 | 0.139 | 2.842 | 0.004500 | 0.061100 | NA |
| ENSG00000086712 | TXLNG | 55787 | 508.18 | 0.397 | 0.141 | 2.81 | 0.005000 | 0.064900 | NA |
| ENSG00000186480 | INSIG1 | 3638 | 4,303.38 | 0.398 | 0.113 | 3.53 | 0.000400 | 0.010700 | NA |
| ENSG00000125871 | MGME1 | 92667 | 1,074.72 | 0.398 | 0.115 | 3.47 | 0.000500 | 0.012900 | NA |
| ENSG00000113161 | HMGCR | 3156 | 2,711.77 | 0.398 | 0.131 | 3.038 | 0.002400 | 0.040200 | NA |
| ENSG00000143067 | ZNF697 | 90874 | 663.972 | 0.398 | 0.149 | 2.675 | 0.007500 | 0.085100 | NA |
| ENSG00000139289 | PHLDA1 | 22822 | 9,372.23 | 0.4 | 0.086 | 4.671 | 0.000000 | 0.000200 | NA |
| ENSG00000075213 | SEMA3A | 10371 | 2,690.21 | 0.4 | 0.101 | 3.953 | 0.000100 | 0.002800 | NA |
| ENSG00000197961 | ZNF121 | 7675 | 511.099 | 0.4 | 0.142 | 2.821 | 0.004800 | 0.063800 | NA |
| ENSG00000143786 | CNIH3 | 149111 | 625.041 | 0.4 | 0.144 | 2.77 | 0.005600 | 0.070500 | NA |
| ENSG00000178904 | DPY19L3 | 147991 | 985.938 | 0.403 | 0.137 | 2.935 | 0.003300 | 0.051100 | NA |
| ENSG00000164687 | FABP5 | 2171 | 281.005 | 0.404 | 0.146 | 2.764 | 0.005700 | 0.071500 | NA |
| ENSG00000173848 | NET1 | 10276 | 1,424.86 | 0.406 | 0.111 | 3.656 | 0.000300 | 0.007300 | NA |
| ENSG00000135503 | ACVR1B | 91 | 753.173 | 0.406 | 0.118 | 3.436 | 0.000600 | 0.014200 | NA |
| ENSG00000106070 | GRB10 | 2887 | 1,148.77 | 0.406 | 0.124 | 3.272 | 0.001100 | 0.022300 | NA |
| ENSG00000080546 | SESN1 | 27244 | 405.228 | 0.406 | 0.143 | 2.838 | 0.004500 | 0.061700 | NA |
| ENSG00000111266 | DUSP16 | 80824 | 471.263 | 0.408 | 0.145 | 2.808 | 0.005000 | 0.065100 | NA |
| ENSG00000059728 | MXD1 | 4084 | 397.668 | 0.408 | 0.152 | 2.681 | 0.007300 | 0.083800 | NA |
| ENSG00000165732 | DDX21 | 9188 | 6,705.31 | 0.409 | 0.134 | 3.061 | 0.002200 | 0.038100 | NA |
| ENSG00000111412 | SPRING1 | 79794 | 2,905.14 | 0.41 | 0.083 | 4.948 | 0.000000 | 0.000100 | NA |
| ENSG00000143751 | SDE2 | 163859 | 871.396 | 0.41 | 0.123 | 3.338 | 0.000800 | 0.018700 | NA |
| ENSG00000173281 | PPP1R3B | 79660 | 702.536 | 0.41 | 0.133 | 3.077 | 0.002100 | 0.036600 | NA |
| ENSG00000109534 | GAR1 | 54433 | 470.864 | 0.41 | 0.134 | 3.064 | 0.002200 | 0.037700 | NA |
| ENSG00000159256 | MORC3 | 23515 | 857.797 | 0.41 | 0.136 | 3.022 | 0.002500 | 0.041400 | NA |
| ENSG00000170191 | NANP | 140838 | 408.357 | 0.41 | 0.155 | 2.65 | 0.008000 | 0.089600 | NA |
| ENSG00000145782 | ATG12 | 9140 | 1,572.09 | 0.411 | 0.112 | 3.665 | 0.000200 | 0.007000 | NA |
| ENSG00000135269 | TES | 26136 | 958.465 | 0.411 | 0.13 | 3.16 | 0.001600 | 0.029700 | NA |
| ENSG00000146858 | ZC3HAV1L | 92092 | 293.146 | 0.411 | 0.143 | 2.871 | 0.004100 | 0.057900 | NA |
| ENSG00000188786 | MTF1 | 4520 | 1,050.53 | 0.412 | 0.101 | 4.092 | 0.000000 | 0.001700 | NA |
| ENSG00000163584 | RPL22L1 | 200916 | 1,685.85 | 0.412 | 0.142 | 2.905 | 0.003700 | 0.054200 | NA |
| ENSG00000233864 |  |  | 484.157 | 0.412 | 0.143 | 2.884 | 0.003900 | 0.056500 | NA |
| ENSG00000151552 | QDPR | 5860 | 1,621.86 | 0.413 | 0.092 | 4.506 | 0.000000 | 0.000400 | NA |
| ENSG00000137075 | RNF38 | 152006 | 810.286 | 0.413 | 0.125 | 3.313 | 0.000900 | 0.020000 | NA |
| ENSG00000112592 | TBP | 6908 | 543.608 | 0.413 | 0.129 | 3.214 | 0.001300 | 0.026000 | NA |
| ENSG00000187678 | SPRY4 | 81848 | 1,057.01 | 0.414 | 0.148 | 2.8 | 0.005100 | 0.066200 | NA |
| ENSG00000131732 | ZCCHC9 | 84240 | 972.921 | 0.415 | 0.133 | 3.113 | 0.001900 | 0.033700 | NA |
| ENSG00000153936 | HS2ST1 | 9653 | 786.979 | 0.415 | 0.144 | 2.888 | 0.003900 | 0.056300 | NA |
| ENSG00000115526 | CHST10 | 9486 | 354.449 | 0.416 | 0.134 | 3.106 | 0.001900 | 0.034300 | NA |
| ENSG00000003436 | TFPI | 7035 | 1,799.09 | 0.416 | 0.137 | 3.042 | 0.002300 | 0.039700 | NA |
| ENSG00000198740 | ZNF652 | 22834 | 393.514 | 0.416 | 0.137 | 3.037 | 0.002400 | 0.040300 | NA |
| ENSG00000136146 | MED4 | 29079 | 444.377 | 0.417 | 0.15 | 2.77 | 0.005600 | 0.070400 | NA |
| ENSG00000197763 | TXNRD3 | 114112 | 279.774 | 0.418 | 0.139 | 3.015 | 0.002600 | 0.042300 | NA |
| ENSG00000134970 | TMED7 | 51014 | 1,120.00 | 0.419 | 0.16 | 2.613 | 0.009000 | 0.097000 | NA |
| ENSG00000134597 | RBMX2 | 51634 | 583.681 | 0.42 | 0.115 | 3.65 | 0.000300 | 0.007400 | NA |
| ENSG00000175066 | GK5 | 256356 | 497.437 | 0.42 | 0.131 | 3.202 | 0.001400 | 0.026600 | NA |
| ENSG00000109689 | STIM2 | 57620 | 795.237 | 0.42 | 0.132 | 3.18 | 0.001500 | 0.028100 | NA |
| ENSG00000091039 | OSBPL8 | 114882 | 3,506.13 | 0.422 | 0.129 | 3.278 | 0.001000 | 0.022000 | NA |
| ENSG00000176697 | BDNF | 627 | 901.646 | 0.423 | 0.143 | 2.963 | 0.003000 | 0.048100 | NA |
| ENSG00000247626 | MARS2 | 92935 | 335.176 | 0.424 | 0.151 | 2.805 | 0.005000 | 0.065400 | NA |
| ENSG00000181467 | RAP2B | 5912 | 1,123.48 | 0.427 | 0.101 | 4.245 | 0.000000 | 0.001000 | NA |
| ENSG00000165169 | DYNLT3 | 6990 | 839.188 | 0.428 | 0.13 | 3.297 | 0.001000 | 0.021000 | NA |
| ENSG00000163348 | PYGO2 | 90780 | 987.353 | 0.429 | 0.094 | 4.556 | 0.000000 | 0.000300 | NA |
| ENSG00000132357 | CARD6 | 84674 | 1,028.61 | 0.429 | 0.142 | 3.029 | 0.002500 | 0.040900 | NA |
| ENSG00000157168 | NRG1 | 3084 | 2,526.10 | 0.43 | 0.11 | 3.922 | 0.000100 | 0.003100 | NA |
| ENSG00000120656 | TAF12 | 6883 | 670.091 | 0.43 | 0.115 | 3.741 | 0.000200 | 0.005500 | NA |
| ENSG00000134901 | POGLUT2 | 79070 | 370.63 | 0.43 | 0.146 | 2.939 | 0.003300 | 0.050700 | NA |
| ENSG00000108578 | BLMH | 642 | 1,052.93 | 0.434 | 0.101 | 4.291 | 0.000000 | 0.000800 | NA |
| ENSG00000144136 | SLC20A1 | 6574 | 13,564.87 | 0.438 | 0.114 | 3.855 | 0.000100 | 0.003900 | NA |
| ENSG00000168769 | TET2 | 54790 | 459.629 | 0.44 | 0.143 | 3.08 | 0.002100 | 0.036300 | NA |
| ENSG00000173334 | TRIB1 | 10221 | 687.035 | 0.443 | 0.129 | 3.448 | 0.000600 | 0.013700 | NA |
| ENSG00000162441 | LZIC | 84328 | 765.756 | 0.444 | 0.124 | 3.593 | 0.000300 | 0.008900 | NA |
| ENSG00000162702 | ZNF281 | 23528 | 910.161 | 0.445 | 0.113 | 3.929 | 0.000100 | 0.003000 | NA |
| ENSG00000129128 | SPCS3 | 60559 | 3,529.56 | 0.445 | 0.14 | 3.179 | 0.001500 | 0.028200 | NA |
| ENSG00000145335 | SNCA | 6622 | 342.523 | 0.445 | 0.159 | 2.8 | 0.005100 | 0.066300 | NA |
| ENSG00000158417 | EIF5B | 9669 | 5,661.56 | 0.446 | 0.111 | 4.017 | 0.000100 | 0.002200 | NA |
| ENSG00000116406 | EDEM3 | 80267 | 1,840.97 | 0.446 | 0.132 | 3.371 | 0.000700 | 0.017000 | NA |
| ENSG00000255529 | POLR2M | 81488 | 397.351 | 0.448 | 0.164 | 2.724 | 0.006400 | 0.077300 | NA |
| ENSG00000100426 | ZBED4 | 9889 | 1,044.77 | 0.451 | 0.109 | 4.13 | 0.000000 | 0.001500 | NA |
| ENSG00000119661 | DNAL1 | 83544 | 420.134 | 0.452 | 0.15 | 3.008 | 0.002600 | 0.042900 | NA |
| ENSG00000248905 | FMN1 | 342184 | 1,248.32 | 0.453 | 0.161 | 2.821 | 0.004800 | 0.063800 | NA |
| ENSG00000106537 | TSPAN13 | 27075 | 413.297 | 0.453 | 0.168 | 2.693 | 0.007100 | 0.081700 | NA |
| ENSG00000164080 | RAD54L2 | 23132 | 970.374 | 0.454 | 0.112 | 4.031 | 0.000100 | 0.002100 | NA |
| ENSG00000153714 | LURAP1L | 286343 | 754.278 | 0.455 | 0.125 | 3.645 | 0.000300 | 0.007500 | NA |
| ENSG00000134897 | BIVM | 54841 | 350.307 | 0.459 | 0.157 | 2.92 | 0.003500 | 0.052400 | NA |
| ENSG00000122966 | CIT | 11113 | 2,449.34 | 0.461 | 0.147 | 3.142 | 0.001700 | 0.031100 | NA |
| ENSG00000145439 | CBR4 | 84869 | 333.155 | 0.461 | 0.151 | 3.044 | 0.002300 | 0.039700 | NA |
| ENSG00000164647 | STEAP1 | 26872 | 1,434.82 | 0.461 | 0.157 | 2.936 | 0.003300 | 0.050900 | NA |
| ENSG00000153574 | RPIA | 22934 | 352.199 | 0.463 | 0.131 | 3.525 | 0.000400 | 0.010900 | NA |
| ENSG00000205268 | PDE7A | 5150 | 248.924 | 0.464 | 0.161 | 2.876 | 0.004000 | 0.057400 | NA |
| ENSG00000106366 | SERPINE1 | 5054 | 42,872.22 | 0.465 | 0.105 | 4.425 | 0.000000 | 0.000500 | NA |
| ENSG00000179051 | RCC2 | 55920 | 2,910.54 | 0.466 | 0.094 | 4.968 | 0.000000 | 0.000100 | NA |
| ENSG00000198840 | ND3 | 4537 | 9,042.42 | 0.468 | 0.126 | 3.709 | 0.000200 | 0.006100 | NA |
| ENSG00000153767 | GTF2E1 | 2960 | 309.891 | 0.468 | 0.134 | 3.485 | 0.000500 | 0.012200 | NA |
| ENSG00000159055 | MIS18A | 54069 | 388.834 | 0.469 | 0.17 | 2.759 | 0.005800 | 0.072100 | NA |
| ENSG00000151876 | FBXO4 | 26272 | 214.449 | 0.471 | 0.16 | 2.945 | 0.003200 | 0.050100 | NA |
| ENSG00000071127 | WDR1 | 9948 | 23,911.31 | 0.473 | 0.096 | 4.915 | 0.000000 | 0.000100 | NA |
| ENSG00000091436 | MAP3K20 | 51776 | 3,417.46 | 0.473 | 0.124 | 3.829 | 0.000100 | 0.004200 | NA |
| ENSG00000019991 | HGF | 3082 | 1,380.71 | 0.473 | 0.152 | 3.116 | 0.001800 | 0.033400 | NA |
| ENSG00000184897 | H1-10 | 8971 | 1,000.01 | 0.473 | 0.166 | 2.85 | 0.004400 | 0.059900 | NA |
| ENSG00000102024 | PLS3 | 5358 | 6,499.65 | 0.474 | 0.12 | 3.949 | 0.000100 | 0.002800 | NA |
| ENSG00000163661 | PTX3 | 5806 | 12,484.24 | 0.475 | 0.143 | 3.333 | 0.000900 | 0.018900 | NA |
| ENSG00000132640 | BTBD3 | 22903 | 721.177 | 0.475 | 0.143 | 3.328 | 0.000900 | 0.019100 | NA |
| ENSG00000180667 | YOD1 | 55432 | 237.212 | 0.475 | 0.156 | 3.039 | 0.002400 | 0.040100 | NA |
| ENSG00000182768 | NGRN | 51335 | 1,386.50 | 0.476 | 0.113 | 4.201 | 0.000000 | 0.001100 | NA |
| ENSG00000186665 | C17orf58 | 284018 | 181.595 | 0.476 | 0.172 | 2.772 | 0.005600 | 0.070200 | NA |
| ENSG00000158161 | EYA3 | 2140 | 954.578 | 0.477 | 0.127 | 3.752 | 0.000200 | 0.005300 | NA |
| ENSG00000180530 | NRIP1 | 8204 | 957.836 | 0.478 | 0.127 | 3.757 | 0.000200 | 0.005200 | NA |
| ENSG00000117533 | VAMP4 | 8674 | 482.277 | 0.479 | 0.158 | 3.02 | 0.002500 | 0.041600 | NA |
| ENSG00000121897 | LIAS | 11019 | 234.845 | 0.479 | 0.164 | 2.921 | 0.003500 | 0.052400 | NA |
| ENSG00000136152 | COG3 | 83548 | 866.807 | 0.48 | 0.106 | 4.546 | 0.000000 | 0.000300 | NA |
| ENSG00000221823 | PPP3R1 | 5534 | 984.627 | 0.48 | 0.121 | 3.976 | 0.000100 | 0.002600 | NA |
| ENSG00000160712 | IL6R | 3570 | 839.345 | 0.481 | 0.14 | 3.433 | 0.000600 | 0.014300 | NA |
| ENSG00000140943 | MBTPS1 | 8720 | 4,982.02 | 0.482 | 0.081 | 5.953 | 0.000000 | 0.000000 | NA |
| ENSG00000141446 | ESCO1 | 114799 | 309.237 | 0.483 | 0.16 | 3.024 | 0.002500 | 0.041300 | NA |
| ENSG00000163597 | SNHG16 | 1.01E+08 | 1,965.50 | 0.484 | 0.119 | 4.069 | 0.000000 | 0.001800 | NA |
| ENSG00000180488 | MIGA1 | 374986 | 1,073.30 | 0.484 | 0.153 | 3.153 | 0.001600 | 0.030200 | NA |
| ENSG00000197885 | NKIRAS1 | 28512 | 555.946 | 0.485 | 0.143 | 3.398 | 0.000700 | 0.015700 | NA |
| ENSG00000117569 | PTBP2 | 58155 | 230.432 | 0.486 | 0.183 | 2.654 | 0.008000 | 0.088700 | NA |
| ENSG00000164761 | TNFRSF11B | 4982 | 14,214.76 | 0.487 | 0.128 | 3.809 | 0.000100 | 0.004400 | NA |
| ENSG00000165029 | ABCA1 | 19 | 732.721 | 0.487 | 0.163 | 2.993 | 0.002800 | 0.044700 | NA |
| ENSG00000136868 | SLC31A1 | 1317 | 3,504.18 | 0.488 | 0.099 | 4.934 | 0.000000 | 0.000100 | NA |
| ENSG00000129317 | PUS7L | 83448 | 592.524 | 0.488 | 0.161 | 3.038 | 0.002400 | 0.040200 | NA |
| ENSG00000125398 | SOX9 | 6662 | 375.862 | 0.488 | 0.164 | 2.973 | 0.003000 | 0.047000 | NA |
| ENSG00000105447 | GRWD1 | 83743 | 2,135.44 | 0.489 | 0.12 | 4.089 | 0.000000 | 0.001700 | NA |
| ENSG00000117143 | UAP1 | 6675 | 5,150.02 | 0.49 | 0.113 | 4.323 | 0.000000 | 0.000700 | NA |
| ENSG00000135913 | USP37 | 57695 | 253.404 | 0.493 | 0.171 | 2.888 | 0.003900 | 0.056300 | NA |
| ENSG00000174136 | RGMB | 285704 | 6,511.72 | 0.495 | 0.108 | 4.587 | 0.000000 | 0.000300 | NA |
| ENSG00000265972 | TXNIP | 10628 | 1,937.25 | 0.496 | 0.127 | 3.89 | 0.000100 | 0.003400 | NA |
| ENSG00000174206 | C12orf66 | 144577 | 199.56 | 0.499 | 0.17 | 2.939 | 0.003300 | 0.050600 | NA |
| ENSG00000205726 | ITSN1 | 6453 | 1,464.02 | 0.503 | 0.117 | 4.302 | 0.000000 | 0.000800 | NA |
| ENSG00000114698 | PLSCR4 | 57088 | 758.573 | 0.503 | 0.133 | 3.794 | 0.000100 | 0.004600 | NA |
| ENSG00000110060 | PUS3 | 83480 | 277.482 | 0.503 | 0.154 | 3.266 | 0.001100 | 0.022600 | NA |
| ENSG00000247556 | OIP5-AS1 | 729082 | 2,333.02 | 0.504 | 0.154 | 3.277 | 0.001000 | 0.022000 | NA |
| ENSG00000164305 | CASP3 | 836 | 2,705.04 | 0.505 | 0.106 | 4.763 | 0.000000 | 0.000100 | NA |
| ENSG00000171951 | SCG2 | 7857 | 167.359 | 0.508 | 0.192 | 2.649 | 0.008100 | 0.089800 | NA |
| ENSG00000249992 | TMEM158 | 25907 | 3,854.14 | 0.51 | 0.145 | 3.531 | 0.000400 | 0.010700 | NA |
| ENSG00000182986 | ZNF320 | 162967 | 319.262 | 0.514 | 0.147 | 3.502 | 0.000500 | 0.011700 | NA |
| ENSG00000072609 | CHFR | 55743 | 1,455.74 | 0.519 | 0.112 | 4.612 | 0.000000 | 0.000200 | NA |
| ENSG00000117020 | AKT3 | 10000 | 1,161.55 | 0.519 | 0.12 | 4.328 | 0.000000 | 0.000700 | NA |
| ENSG00000179041 | RRS1 | 23212 | 962.739 | 0.519 | 0.136 | 3.813 | 0.000100 | 0.004400 | NA |
| ENSG00000174574 | AKIRIN1 | 79647 | 2,673.77 | 0.521 | 0.086 | 6.07 | 0.000000 | 0.000000 | NA |
| ENSG00000177283 | FZD8 | 8325 | 1,042.47 | 0.522 | 0.192 | 2.723 | 0.006500 | 0.077300 | NA |
| ENSG00000104381 | GDAP1 | 54332 | 420.087 | 0.523 | 0.125 | 4.2 | 0.000000 | 0.001100 | NA |
| ENSG00000112237 | CCNC | 892 | 868.285 | 0.524 | 0.129 | 4.067 | 0.000000 | 0.001900 | NA |
| ENSG00000113070 | HBEGF | 1839 | 962.491 | 0.525 | 0.143 | 3.678 | 0.000200 | 0.006700 | NA |
| ENSG00000198642 | KLHL9 | 55958 | 1,752.45 | 0.526 | 0.101 | 5.205 | 0.000000 | 0.000000 | NA |
| ENSG00000117523 | PRRC2C | 23215 | 7,122.39 | 0.527 | 0.13 | 4.05 | 0.000100 | 0.002000 | NA |
| ENSG00000185728 | YTHDF3 | 253943 | 2,798.69 | 0.53 | 0.102 | 5.203 | 0.000000 | 0.000000 | NA |
| ENSG00000177058 | SLC38A9 | 153129 | 405.115 | 0.53 | 0.16 | 3.312 | 0.000900 | 0.020000 | NA |
| ENSG00000118971 | CCND2 | 894 | 206.416 | 0.534 | 0.158 | 3.382 | 0.000700 | 0.016500 | NA |
| ENSG00000163291 | PAQR3 | 152559 | 860.017 | 0.535 | 0.161 | 3.312 | 0.000900 | 0.020000 | NA |
| ENSG00000173011 | TADA2B | 93624 | 1,087.35 | 0.536 | 0.113 | 4.74 | 0.000000 | 0.000100 | NA |
| ENSG00000107949 | BCCIP | 56647 | 1,613.33 | 0.536 | 0.121 | 4.426 | 0.000000 | 0.000500 | NA |
| ENSG00000131149 | GSE1 | 23199 | 1,586.88 | 0.536 | 0.127 | 4.237 | 0.000000 | 0.001000 | NA |
| ENSG00000139324 | TMTC3 | 160418 | 1,116.51 | 0.537 | 0.143 | 3.768 | 0.000200 | 0.005100 | NA |
| ENSG00000122912 | SLC25A16 | 8034 | 613.267 | 0.541 | 0.137 | 3.939 | 0.000100 | 0.002900 | NA |
| ENSG00000178726 | THBD | 7056 | 3,121.07 | 0.543 | 0.122 | 4.448 | 0.000000 | 0.000400 | NA |
| ENSG00000054793 | ATP9A | 10079 | 1,868.94 | 0.544 | 0.104 | 5.226 | 0.000000 | 0.000000 | NA |
| ENSG00000145390 | USP53 | 54532 | 2,037.33 | 0.544 | 0.142 | 3.828 | 0.000100 | 0.004200 | NA |
| ENSG00000137876 | RSL24D1 | 51187 | 1,389.23 | 0.545 | 0.133 | 4.097 | 0.000000 | 0.001700 | NA |
| ENSG00000153814 | JAZF1 | 221895 | 318.87 | 0.546 | 0.15 | 3.637 | 0.000300 | 0.007700 | NA |
| ENSG00000143033 | MTF2 | 22823 | 405.994 | 0.547 | 0.168 | 3.254 | 0.001100 | 0.023300 | NA |
| ENSG00000204442 | FAM155A | 728215 | 454.308 | 0.548 | 0.139 | 3.952 | 0.000100 | 0.002800 | NA |
| ENSG00000243742 | RPLP0P2 | 113157 | 166.095 | 0.548 | 0.181 | 3.026 | 0.002500 | 0.041200 | NA |
| ENSG00000120519 | SLC10A7 | 84068 | 185.099 | 0.548 | 0.208 | 2.629 | 0.008600 | 0.094000 | NA |
| ENSG00000165434 | PGM2L1 | 283209 | 300.004 | 0.549 | 0.159 | 3.447 | 0.000600 | 0.013800 | NA |
| ENSG00000101844 | ATG4A | 115201 | 502.757 | 0.55 | 0.155 | 3.549 | 0.000400 | 0.010100 | NA |
| ENSG00000155660 | PDIA4 | 9601 | 7,959.99 | 0.551 | 0.104 | 5.311 | 0.000000 | 0.000000 | NA |
| ENSG00000170802 | FOXN2 | 3344 | 459.728 | 0.553 | 0.161 | 3.443 | 0.000600 | 0.014000 | NA |
| ENSG00000198018 | ENTPD7 | 57089 | 2,354.54 | 0.554 | 0.113 | 4.906 | 0.000000 | 0.000100 | NA |
| ENSG00000148680 | HTR7 | 3363 | 152.15 | 0.554 | 0.209 | 2.647 | 0.008100 | 0.090200 | NA |
| ENSG00000164414 | SLC35A1 | 10559 | 115.93 | 0.555 | 0.212 | 2.62 | 0.008800 | 0.095600 | NA |
| ENSG00000177432 | NAP1L5 | 266812 | 357.375 | 0.557 | 0.149 | 3.742 | 0.000200 | 0.005500 | NA |
| ENSG00000257219 | LNCOG | 1.05E+08 | 354.965 | 0.558 | 0.166 | 3.37 | 0.000800 | 0.017000 | NA |
| ENSG00000189266 | PNRC2 | 55629 | 2,591.14 | 0.565 | 0.106 | 5.31 | 0.000000 | 0.000000 | NA |
| ENSG00000226380 |  |  | 847.348 | 0.568 | 0.134 | 4.251 | 0.000000 | 0.000900 | NA |
| ENSG00000110987 | BCL7A | 605 | 388.436 | 0.568 | 0.179 | 3.183 | 0.001500 | 0.028000 | NA |
| ENSG00000120149 | MSX2 | 4488 | 459.747 | 0.569 | 0.152 | 3.756 | 0.000200 | 0.005200 | NA |
| ENSG00000136158 | SPRY2 | 10253 | 3,500.72 | 0.571 | 0.124 | 4.608 | 0.000000 | 0.000200 | NA |
| ENSG00000141664 | ZCCHC2 | 54877 | 968.049 | 0.571 | 0.131 | 4.354 | 0.000000 | 0.000600 | NA |
| ENSG00000152104 | PTPN14 | 5784 | 4,056.59 | 0.572 | 0.14 | 4.095 | 0.000000 | 0.001700 | NA |
| ENSG00000103449 | SALL1 | 6299 | 146.379 | 0.572 | 0.208 | 2.747 | 0.006000 | 0.074200 | NA |
| ENSG00000150867 | PIP4K2A | 5305 | 1,442.17 | 0.574 | 0.109 | 5.265 | 0.000000 | 0.000000 | NA |
| ENSG00000116489 | CAPZA1 | 829 | 6,382.40 | 0.576 | 0.119 | 4.857 | 0.000000 | 0.000100 | NA |
| ENSG00000165288 | BRWD3 | 254065 | 525.347 | 0.578 | 0.159 | 3.627 | 0.000300 | 0.007900 | NA |
| ENSG00000139697 | SBNO1 | 55206 | 1,821.04 | 0.579 | 0.111 | 5.223 | 0.000000 | 0.000000 | NA |
| ENSG00000135905 | DOCK10 | 55619 | 2,875.64 | 0.579 | 0.124 | 4.688 | 0.000000 | 0.000200 | NA |
| ENSG00000102189 | EEA1 | 8411 | 1,828.66 | 0.579 | 0.144 | 4.019 | 0.000100 | 0.002200 | NA |
| ENSG00000135597 | REPS1 | 85021 | 921.14 | 0.581 | 0.126 | 4.595 | 0.000000 | 0.000300 | NA |
| ENSG00000002745 | WNT16 | 51384 | 677.097 | 0.581 | 0.169 | 3.436 | 0.000600 | 0.014200 | NA |
| ENSG00000181690 | PLAG1 | 5324 | 114.621 | 0.583 | 0.209 | 2.79 | 0.005300 | 0.067600 | NA |
| ENSG00000174099 | MSRB3 | 253827 | 3,328.86 | 0.584 | 0.107 | 5.447 | 0.000000 | 0.000000 | NA |
| ENSG00000163818 | LZTFL1 | 54585 | 380.348 | 0.584 | 0.154 | 3.781 | 0.000200 | 0.004900 | NA |
| ENSG00000205133 | TRIQK | 286144 | 326.481 | 0.588 | 0.168 | 3.51 | 0.000400 | 0.011400 | NA |
| ENSG00000134955 | SLC37A2 | 219855 | 301.48 | 0.589 | 0.183 | 3.212 | 0.001300 | 0.026100 | NA |
| ENSG00000099810 | MTAP | 4507 | 780.702 | 0.592 | 0.129 | 4.6 | 0.000000 | 0.000300 | NA |
| ENSG00000114999 | TTL | 150465 | 3,313.42 | 0.593 | 0.122 | 4.883 | 0.000000 | 0.000100 | NA |
| ENSG00000163428 | LRRC58 | 116064 | 1,488.57 | 0.594 | 0.166 | 3.586 | 0.000300 | 0.009000 | NA |
| ENSG00000150630 | VEGFC | 7424 | 5,513.32 | 0.595 | 0.128 | 4.634 | 0.000000 | 0.000200 | NA |
| ENSG00000154188 | ANGPT1 | 284 | 683.563 | 0.595 | 0.162 | 3.68 | 0.000200 | 0.006700 | NA |
| ENSG00000198874 | TYW1 | 55253 | 651.004 | 0.601 | 0.104 | 5.759 | 0.000000 | 0.000000 | NA |
| ENSG00000135828 | RNASEL | 6041 | 431.461 | 0.602 | 0.138 | 4.365 | 0.000000 | 0.000600 | NA |
| ENSG00000164124 | TMEM144 | 55314 | 146.151 | 0.602 | 0.18 | 3.349 | 0.000800 | 0.018100 | NA |
| ENSG00000140450 | ARRDC4 | 91947 | 679.056 | 0.603 | 0.148 | 4.081 | 0.000000 | 0.001800 | NA |
| ENSG00000178105 | DDX10 | 1662 | 585.688 | 0.604 | 0.148 | 4.095 | 0.000000 | 0.001700 | NA |
| ENSG00000138678 | GPAT3 | 84803 | 300.559 | 0.607 | 0.187 | 3.239 | 0.001200 | 0.024400 | NA |
| ENSG00000203727 | SAMD5 | 389432 | 237.199 | 0.608 | 0.172 | 3.53 | 0.000400 | 0.010700 | NA |
| ENSG00000163629 | PTPN13 | 5783 | 1,887.50 | 0.609 | 0.162 | 3.772 | 0.000200 | 0.005000 | NA |
| ENSG00000185818 | NAT8L | 339983 | 136.396 | 0.61 | 0.224 | 2.725 | 0.006400 | 0.077300 | NA |
| ENSG00000249915 | PDCD6 | 10016 | 2,304.23 | 0.617 | 0.092 | 6.705 | 0.000000 | 0.000000 | NA |
| ENSG00000071537 | SEL1L | 6400 | 5,248.78 | 0.619 | 0.099 | 6.286 | 0.000000 | 0.000000 | NA |
| ENSG00000087095 | NLK | 51701 | 476.257 | 0.619 | 0.117 | 5.293 | 0.000000 | 0.000000 | NA |
| ENSG00000170537 | TMC7 | 79905 | 138.007 | 0.619 | 0.209 | 2.954 | 0.003100 | 0.049000 | NA |
| ENSG00000168811 | IL12A | 3592 | 364.95 | 0.621 | 0.175 | 3.554 | 0.000400 | 0.010000 | NA |
| ENSG00000115339 | GALNT3 | 2591 | 376.332 | 0.623 | 0.159 | 3.928 | 0.000100 | 0.003000 | NA |
| ENSG00000167193 | CRK | 1398 | 4,618.45 | 0.627 | 0.091 | 6.853 | 0.000000 | 0.000000 | NA |
| ENSG00000145685 | LHFPL2 | 10184 | 5,379.25 | 0.632 | 0.097 | 6.501 | 0.000000 | 0.000000 | NA |
| ENSG00000139372 | TDG | 6996 | 717.489 | 0.648 | 0.121 | 5.352 | 0.000000 | 0.000000 | NA |
| ENSG00000008083 | JARID2 | 3720 | 600.717 | 0.651 | 0.132 | 4.933 | 0.000000 | 0.000100 | NA |
| ENSG00000155903 | RASA2 | 5922 | 715.443 | 0.651 | 0.167 | 3.9 | 0.000100 | 0.003300 | NA |
| ENSG00000138757 | G3BP2 | 9908 | 4,524.71 | 0.652 | 0.115 | 5.674 | 0.000000 | 0.000000 | NA |
| ENSG00000153044 | CENPH | 64946 | 288.996 | 0.653 | 0.192 | 3.397 | 0.000700 | 0.015700 | NA |
| ENSG00000143384 | MCL1 | 4170 | 10,658.82 | 0.655 | 0.089 | 7.377 | 0.000000 | 0.000000 | NA |
| ENSG00000134146 | DPH6 | 89978 | 121.862 | 0.665 | 0.246 | 2.702 | 0.006900 | 0.080600 | NA |
| ENSG00000175334 | BANF1 | 8815 | 3,277.42 | 0.667 | 0.093 | 7.149 | 0.000000 | 0.000000 | NA |
| ENSG00000213281 | NRAS | 4893 | 2,948.26 | 0.667 | 0.13 | 5.122 | 0.000000 | 0.000000 | NA |
| ENSG00000151611 | MMAA | 166785 | 247.468 | 0.67 | 0.173 | 3.862 | 0.000100 | 0.003800 | NA |
| ENSG00000177570 | SAMD12 | 401474 | 137.354 | 0.671 | 0.191 | 3.509 | 0.000400 | 0.011400 | NA |
| ENSG00000065613 | SLK | 9748 | 2,844.38 | 0.673 | 0.117 | 5.767 | 0.000000 | 0.000000 | NA |
| ENSG00000136098 | NEK3 | 4752 | 245.399 | 0.673 | 0.168 | 4.002 | 0.000100 | 0.002300 | NA |
| ENSG00000143702 | CEP170 | 9859 | 1,973.70 | 0.675 | 0.146 | 4.618 | 0.000000 | 0.000200 | NA |
| ENSG00000138669 | PRKG2 | 5593 | 208.99 | 0.675 | 0.181 | 3.731 | 0.000200 | 0.005600 | NA |
| ENSG00000069431 | ABCC9 | 10060 | 187.996 | 0.679 | 0.256 | 2.655 | 0.007900 | 0.088700 | NA |
| ENSG00000197566 | ZNF624 | 57547 | 97.492 | 0.683 | 0.211 | 3.231 | 0.001200 | 0.024900 | NA |
| ENSG00000185127 | C6orf120 | 387263 | 1,289.79 | 0.684 | 0.098 | 6.986 | 0.000000 | 0.000000 | NA |
| ENSG00000163743 | RCHY1 | 25898 | 300.517 | 0.684 | 0.17 | 4.018 | 0.000100 | 0.002200 | NA |
| ENSG00000157823 | AP3S2 | 10239 | 451.1 | 0.692 | 0.133 | 5.214 | 0.000000 | 0.000000 | NA |
| ENSG00000225339 |  |  | 89.393 | 0.692 | 0.219 | 3.158 | 0.001600 | 0.029800 | NA |
| ENSG00000181938 | GINS3 | 64785 | 320.136 | 0.695 | 0.182 | 3.809 | 0.000100 | 0.004400 | NA |
| ENSG00000091844 | RGS17 | 26575 | 208.329 | 0.701 | 0.178 | 3.938 | 0.000100 | 0.002900 | NA |
| ENSG00000101384 | JAG1 | 182 | 1,025.95 | 0.703 | 0.13 | 5.402 | 0.000000 | 0.000000 | NA |
| ENSG00000125772 | GPCPD1 | 56261 | 1,109.78 | 0.704 | 0.127 | 5.559 | 0.000000 | 0.000000 | NA |
| ENSG00000164983 | TMEM65 | 157378 | 513.321 | 0.706 | 0.157 | 4.498 | 0.000000 | 0.000400 | NA |
| ENSG00000101972 | STAG2 | 10735 | 2,037.40 | 0.707 | 0.126 | 5.592 | 0.000000 | 0.000000 | NA |
| ENSG00000065559 | MAP2K4 | 6416 | 1,229.09 | 0.708 | 0.127 | 5.576 | 0.000000 | 0.000000 | NA |
| ENSG00000180398 | MCFD2 | 90411 | 9,118.06 | 0.71 | 0.11 | 6.458 | 0.000000 | 0.000000 | NA |
| ENSG00000011007 | ELOA | 6924 | 3,921.96 | 0.715 | 0.109 | 6.588 | 0.000000 | 0.000000 | NA |
| ENSG00000147421 | HMBOX1 | 79618 | 246.825 | 0.723 | 0.162 | 4.473 | 0.000000 | 0.000400 | NA |
| ENSG00000175906 | ARL4D | 379 | 1,304.13 | 0.725 | 0.124 | 5.854 | 0.000000 | 0.000000 | NA |
| ENSG00000141682 | PMAIP1 | 5366 | 2,098.69 | 0.725 | 0.162 | 4.472 | 0.000000 | 0.000400 | NA |
| ENSG00000134864 | GGACT | 87769 | 117.078 | 0.727 | 0.198 | 3.68 | 0.000200 | 0.006700 | NA |
| ENSG00000132664 | POLR3F | 10621 | 542.884 | 0.736 | 0.139 | 5.286 | 0.000000 | 0.000000 | NA |
| ENSG00000112305 | SMAP1 | 60682 | 249.401 | 0.737 | 0.146 | 5.042 | 0.000000 | 0.000000 | NA |
| ENSG00000065320 | NTN1 | 9423 | 1,090.16 | 0.742 | 0.166 | 4.481 | 0.000000 | 0.000400 | NA |
| ENSG00000134490 | TMEM241 | 85019 | 245.634 | 0.744 | 0.163 | 4.565 | 0.000000 | 0.000300 | NA |
| ENSG00000133111 | RFXAP | 5994 | 75.296 | 0.746 | 0.27 | 2.76 | 0.005800 | 0.072100 | NA |
| ENSG00000274211 | SOCS7 | 30837 | 587.58 | 0.75 | 0.123 | 6.092 | 0.000000 | 0.000000 | NA |
| ENSG00000197223 | C1D | 10438 | 408.419 | 0.751 | 0.145 | 5.197 | 0.000000 | 0.000000 | NA |
| ENSG00000137713 | PPP2R1B | 5519 | 1,672.05 | 0.757 | 0.143 | 5.303 | 0.000000 | 0.000000 | NA |
| ENSG00000117758 | STX12 | 23673 | 2,130.89 | 0.759 | 0.12 | 6.33 | 0.000000 | 0.000000 | NA |
| ENSG00000196459 | TRAPPC2 | 6399 | 251.298 | 0.772 | 0.143 | 5.403 | 0.000000 | 0.000000 | NA |
| ENSG00000112576 | CCND3 | 896 | 3,364.89 | 0.773 | 0.139 | 5.554 | 0.000000 | 0.000000 | NA |
| ENSG00000182934 | SRPRA | 6734 | 9,707.82 | 0.774 | 0.091 | 8.5 | 0.000000 | 0.000000 | NA |
| ENSG00000176018 | LYSMD3 | 116068 | 518.55 | 0.774 | 0.179 | 4.317 | 0.000000 | 0.000700 | NA |
| ENSG00000171033 | PKIA | 5569 | 192.632 | 0.775 | 0.227 | 3.417 | 0.000600 | 0.014900 | NA |
| ENSG00000247315 | ZCCHC3 | 85364 | 724.55 | 0.776 | 0.125 | 6.221 | 0.000000 | 0.000000 | NA |
| ENSG00000022567 | SLC45A4 | 57210 | 238.113 | 0.777 | 0.154 | 5.059 | 0.000000 | 0.000000 | NA |
| ENSG00000187325 | TAF9B | 51616 | 588.622 | 0.778 | 0.145 | 5.361 | 0.000000 | 0.000000 | NA |
| ENSG00000145919 | BOD1 | 91272 | 1,687.42 | 0.782 | 0.096 | 8.152 | 0.000000 | 0.000000 | NA |
| ENSG00000140043 | PTGR2 | 145482 | 69.129 | 0.782 | 0.278 | 2.817 | 0.004900 | 0.064300 | NA |
| ENSG00000203786 | KPRP | 448834 | 120.741 | 0.796 | 0.293 | 2.718 | 0.006600 | 0.078100 | NA |
| ENSG00000171345 | KRT19 | 3880 | 914.636 | 0.802 | 0.206 | 3.888 | 0.000100 | 0.003400 | NA |
| ENSG00000067048 | DDX3Y | 8653 | 2,176.59 | 0.806 | 0.122 | 6.617 | 0.000000 | 0.000000 | NA |
| ENSG00000049192 | ADAMTS6 | 11174 | 482.835 | 0.81 | 0.176 | 4.6 | 0.000000 | 0.000300 | NA |
| ENSG00000088205 | DDX18 | 8886 | 2,072.50 | 0.812 | 0.121 | 6.718 | 0.000000 | 0.000000 | NA |
| ENSG00000107614 | TRDMT1 | 1787 | 165.848 | 0.831 | 0.204 | 4.066 | 0.000000 | 0.001900 | NA |
| ENSG00000125848 | FLRT3 | 23767 | 146.687 | 0.844 | 0.219 | 3.854 | 0.000100 | 0.003900 | NA |
| ENSG00000154727 | GABPA | 2551 | 522.766 | 0.856 | 0.137 | 6.245 | 0.000000 | 0.000000 | NA |
| ENSG00000163041 | H3-3A | 3020 | 1,298.30 | 0.867 | 0.108 | 8.004 | 0.000000 | 0.000000 | NA |
| ENSG00000128617 | OPN1SW | 611 | 135.02 | 0.881 | 0.19 | 4.633 | 0.000000 | 0.000200 | NA |
| ENSG00000117868 | ESYT2 | 57488 | 3,499.46 | 0.882 | 0.089 | 9.873 | 0.000000 | 0.000000 | NA |
| ENSG00000257218 | GATC | 283459 | 546.705 | 0.894 | 0.115 | 7.753 | 0.000000 | 0.000000 | NA |
| ENSG00000196396 | PTPN1 | 5770 | 3,047.24 | 0.902 | 0.118 | 7.659 | 0.000000 | 0.000000 | NA |
[truncated: 118,027 more chars]
